# Autophagy regulates PVALB (parvalbumin) interneuron excitability and memory

**DOI:** 10.1080/15548627.2025.2597463

**Published:** 2025-12-14

**Authors:** Theodora Chalatsi, Erin Wosnitzka, Angeliki Kolaxi, Laura M.J. Fernandez, Jules Scholler, Laura Batti, Leonardo Restivo, Graham Knott, Anita Lüthi, Manuel Mameli, Vassiliki Nikoletopoulou

**Affiliations:** a Department of Fundamental Neurosciences, University of Lausanne, Lausanne, Switzerland; b Wyss Center, Geneva, Switzerland; c EPFL, Bioelectron Microscopy Core Facility, Lausanne, Switzerland

**Keywords:** Cerebellum, hippocampus, inhibitory neurotransmission, memory, neuronal death, purkinje cells

## Abstract

Macroautophagy/autophagy was previously shown to play a critical role in the hippocampus for memory formation, with age-related autophagy deficits being further linked to cognitive decline. However, the neuronal subtypes where autophagy is required to form new memories remain unknown. Given the well-established role of PVALB (parvalbumin) interneurons in hippocampus-dependent memory formation and consolidation, we examined whether autophagy in these cells is required for such complex behaviors. We show that contrary to other neuronal subtypes, the vast majority of PVALB neurons, with the exception of cerebellar Purkinje cells, survive and are maintained long-term independently of autophagy. However, autophagy controls the homeostasis of mitochondria, endoplasmic reticulum, and synaptic proteins within PVALB interneurons, ultimately regulating their synaptic excitation, neuronal excitability and excitation-inhibition balance in the hippocampus. Consequently, mice with conditional impairment of autophagy in PVALB-expressing neurons exhibit impaired inhibitory neurotransmission and deficits in hippocampus-dependent memory. Taken together, these findings identify PVALB interneurons as key cellular substrates of autophagy in the context of learning and memory.

**Abbreviation**: ATG5: autophagy related 5; BNIP3: BCL2/adenovirus E1B interacting protein 3; BNIP3L: BCL2/adenovirus E1B interacting protein 3-like; CA1: cornu ammonis 1; CALCOCO1: calcium binding and coiled coil domain 1; ER: endoplasmic reticulum; GABA: gamma-aminobutyric acid; GRIA/AMPAR: glutamate receptor, ionotropic, AMPA; GRIN2A/NR2A/GluN2A: glutamate receptor, ionotropic, NMDA2A (epsilon 1); PRKN: parkin RBR E3 ubiquitin protein ligase; PC: pyramidal cells; PJ: Purkinje; PVALB: parvalbumin; RTN3: reticulon 3; SQSTM1/p62: sequestosome 1.

## Introduction

In humans, genetic mutations resulting in diminished autophagic activity manifest as complex neurodevelopmental disorders, entailing epilepsy and impaired cognitive performance [1] or late-onset neurodegenerative diseases [2]. However, the role of autophagy in the maintenance and synaptic transmission of diverse neurons remains insufficiently characterized.

In mammals, the developmental ablation of core autophagy genes, such as *Atg5* [3,4], *Atg7* [5] or *Wdr45* [6] in the neural lineage, leads to progressive and widespread neuronal death. In these studies, autophagy genes were ablated in multiple brain cell types, including progenitors and their derived neurons, astrocytes, and oligodendrocytes, making it difficult to ascertain whether autophagy is required by post-mitotic neurons in a cell-autonomous manner for their survival or sustenance.

Beyond its role in neuronal survival, accumulating evidence suggests that autophagy also regulates several aspects of glutamatergic synaptic transmission. This includes the development and maturation of excitatory synapses, by facilitating dendritic spine pruning [7], long-term synaptic depression (LTD) through the degradation of post-synaptic proteins [8,9], and basal excitatory neurotransmission, through the regulation of presynaptic ER-homeostasis and thus, calcium stores [4].

In agreement with its emergent synaptic functions, autophagy was also shown to be an essential component of hippocampus-dependent learning and memory [10,11]. However, whether autophagy is required by the specific cellular substrates for these behaviors remains elusive. This is largely due to lack of knowledge on whether autophagy also regulates the sustenance and synaptic properties of other neuronal populations, such as interneurons, which provide inhibition to the neuronal networks they are embedded in.

We hypothesized that the memory deficits associated with impaired brain autophagy may involve the dysfunction of PVALB (parvalbumin) expressing neurons. In the cortex and hippocampus, PVALB is mostly expressed by post-mitotic fast spiking interneurons [12]. Despite comprising a small fraction of the brain’s neuronal network, these cells are characterized by unique morphological and molecular properties that make them key players of excitation-inhibition balance and GABAergic disinhibition between circuitries [13]. As well as being heavily implicated in neuro-psychiatric diseases [14–19], PVALB-expressing neurons have high metabolic demands [20–22], rendering them potentially vulnerable to different types of stress [22] including autophagy impairment.

To address this hypothesis, we generated and characterized mice with conditional ablation of *Atg5* in PVALB-expressing neurons. While confirming that cerebellar Purkinje cells, which are GABAergic projection neurons, are selectively eliminated in the absence of functional autophagy as previously described [23,24], we demonstrate that the survival of PVALB-expressing GABAergic interneurons remains unaffected. We also reveal a pivotal role of autophagy in regulating the organellostasis as well as neurotransmission and excitability of PVALB interneurons, with direct repercussions on the hippocampal network and on memory.

## Results

### Generation of mice with conditional impairment of autophagy in PVALB-expressing neurons

To study the functions of autophagy in PVALB-expressing neurons, we crossed *Pvalb-Cre* deleter mice [25] with mice carrying floxed alleles of *Atg5* [3]. The resulting *Pvalb-cre;atg5*^*f/f*^ progeny (referred to hereafter as *Pvalb-atg5*) were born at the expected mendelian ratios. PVALB expression within brain is known to start in adolescence, at postnatal day 14 [26,27]. Upon reaching three-months-of-age, *Pvalb-atg5* mice presented with a significant reduction in body weight when compared to their *Atg5*^*f/f*^ littermates (referred to here as control) (Figures S1A, B). As levels of food consumption were comparable across genotypes, differences in weight were not ascribed to reduced food consumption in *Pvalb-atg5* mice (Figure S1C). We crossed *Pvalb-Cre* and *Pvalb-atg5* animals with the Ai9 Cre-dependent Td-Tomato (*TdT*) reporter strain [28] and confirmed that across multiple brain areas, > 90% of TdT-positive cells were co-labeled using antibodies against PVALB, as illustrated by representative images from the hippocampus and somatosensory cortex (Figure S1D, E). We also observed the expression of TdT in fast twitching (type II fibers), oxidative muscles, such as the gastrocnemius and tibialis anterior, but not in glycolytic muscles such as the soleus (type I fibers) (Figure S1F). Despite not observing any changes in the mass of the PVALB-expressing muscles (Figure S1G), changes in their physiology may account for the reduced body weight, as it was previously described in mice with conditional deletion of *Atg5* or *Atg7* in skeletal muscles [29].

To confirm that *Atg5* ablation indeed results in autophagy impairment, we FAC-sorted TdT-positive cells from the brains of 3-month-old *Pvalb-TdT* and *Pvalb-atg5-TdT* mice and performed Jess Simple Western™ analysis using antibodies against the autophagic receptor and substrate SQSTM1/p62. As shown in Figure S1H, SQSTM1/p62 protein levels were significantly increased in sorted neuronal lysates from *Pvalb-atg5-TdT* mice compared to *Pvalb-TdT* controls, indicative of impaired autophagic degradation due to the absence of *Atg5* within these cells.

Using mice of the same age, we then prepared protein lysates from cerebellar cortices of control and *Pvalb-atg5* littermates, a brain area containing a high proportion of PVALB-expressing cells. Western blot analyses indicated a significant accumulation of SQSTM1/p62 (Figure S1I), as well as another autophagy receptor, WDFY3/ALFY (Figure S1J) in the *Pvalb-atg5* cerebellum, both of which are known to accumulate when autophagic degradation is impaired. Moreover, analysis with an antibody against ATG5, recognizing the ATG12–ATG5 complex at ~ 60 kDa, confirmed a significant reduction of the ATG5 protein in *Pvalb-atg5* cerebellum when compared to controls (Figure S1K).

### Autophagy is dispensable for PVALB-interneuron survival and maintenance

**Figure 1.**
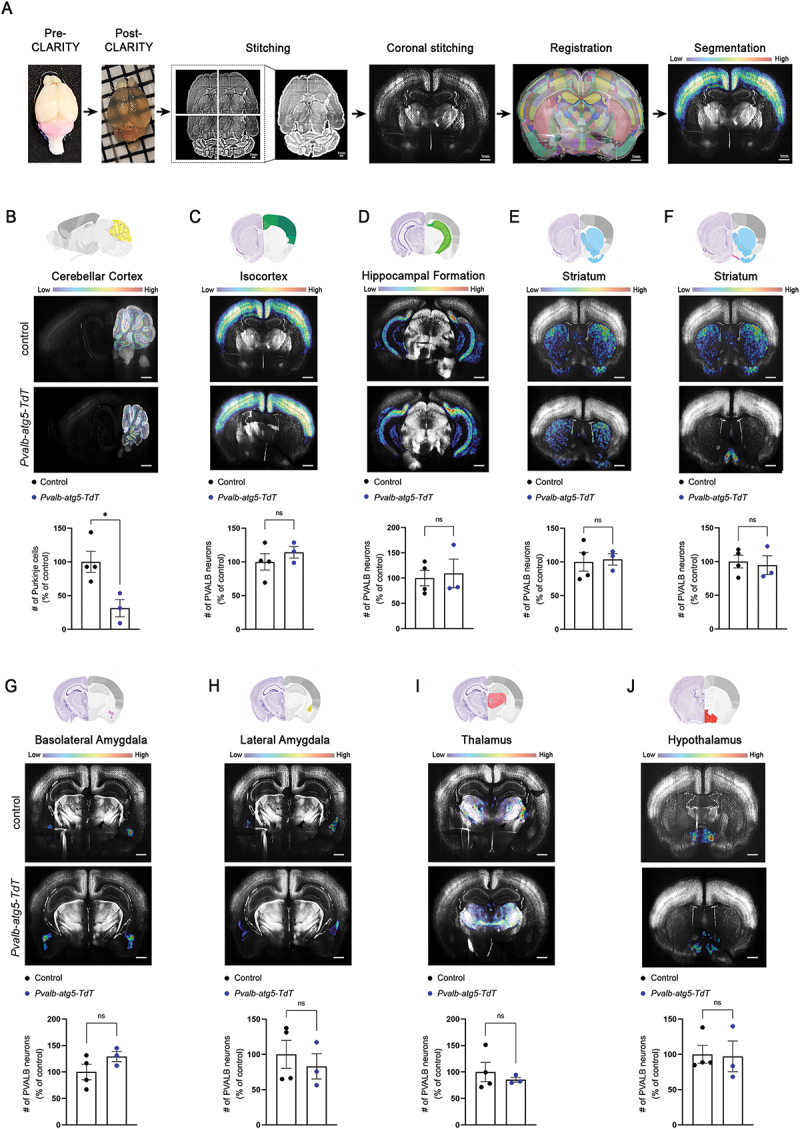
Selective elimination of Purkinje cells and not of interneurons upon genetic autophagy impairment. (A) Overview of the experimental pipeline for whole brain clearance, imaging, and analysis of three-month-old control (*Pvalb-TdT*) and mutant *Pvalb-atg5-TdT* littermates. Scale bars: 1 mm. (B-J) Top rows: Schematic representation and representative heatmaps from the brain segmented masks of control (*Pvalb-TdT*) and *Pvalb-atg5-TdT animals* from the cerebellar cortex (yellow) (B), isocortex (green) (C), hippocampal formation (green) (D), striatum (blue) (E), medial and lateral septum (pink) (F), basolateral amygdala (purple) (G), lateral amygdala (yellow) (H), thalamus (pink) (I) and hypothalamus (red) (J). Scale bar represents 1 mm. Bottom rows: graphs showing the number of TdT^+^ counted cells in the specific masked areas, expressed as percentage of the average of the control. Statistical analyses were performed using unpaired, two-tailed Student’s t tests (*p = 0.0232, *t* = 3.229, df = 5 (B) and ^ns^*p* = 0.4177, *t* = 0.8828, df = 5 (C), ^ns^*p* = 0.7674, *t* = 0.3123, df = 5 (D), ^ns^*p* = 0.8389, *t* = 0.2128, df = 5 (E), ^ns^*p* = 0.7549, *t* = 0.3298, df = 5 (F), ^ns^*p* = 0.1840, *t* = 1.541, df = 5 (G), ^ns^*p* = 0.5714, *t* = 0.6053, df = 5 (H), ^ns^*p* = 0.5332, *t* = 0.6690, df = 5 (I) and ^ns^*p* = 0.9087, *t* = 0.1206, df = 5 (J). Bars represent mean values ± SEM. *N* = 4 and 3 animals for control and *PVALB-atg5-TdT* counts, respectively.

To assess the requirement of autophagy for PVALB neuron maintenance across the brain in an unbiased manner, we used CLARITY combined with light sheet microscopy and machine learning (outlined in Figure 1A) to compare the number of PVALB-positive neurons between three-month-old control (*Pvalb-TdT)* and *Pvalb-atg5-TdT* mice. We started by analyzing the Purkinje cell layer of the cerebellum, which contains the GABAergic projection Purkinje neurons that express both CALB (calbindin) and PVALB and which degenerate upon loss of autophagy [30]. Consistent with this previous work, we found a significant reduction in the number of TdT-positive Purkinje cells in *Pvalb-atg5-TdT* brains when compared to controls (Figure 1B). In line with the major role of Purkinje cells in motor coordination, three-month-old *Pvalb-atg5* mice exhibited impaired motor coordination in a rotarod test when compared to control littermates, including a significant reduction in their mean latency to fall (Figure S2A) that was consistent across trials (Figure S2B). The loss of Purkinje cells was also observed by immunoreactivity for active CASP3 (caspase 3) in the cerebellar Purkinje cell layer of *Pvalb-atg5* animals (Figures S2C, D, arrows). However, no active CASP3 staining could be observed in the molecular cell layer of the cerebellum, which contains PVALB-positive GABAergic interneurons (Figure S2C, E, arrowheads). Even at six-months of age, while the numbers of Purkinje cells were significantly reduced (Figure S2F,G; arrows), no differences could be detected in the number of PVALB-positive interneurons in the molecular cell layer of the cerebellum of *Pvalb-atg5* animals compared to controls (Figure S2F, H; arrowheads), suggesting that interneurons may not require autophagy to survive. In line with this idea, analysis of eight major brain regions known to be rich in PVALB-interneurons [31] indicated no significant differences in the number of TdT-positive cells in *Pvalb-atg5* mice when compared to age-matched controls (Figures 1C-J; total cell counts in Table S1). These areas include the isocortex, hippocampus, striatum, septum, basolateral amygdala, lateral amygdala, thalamus and hypothalamus. Notably, the volume of these brain structures was comparable between genotypes (Table S1). In line with this, a TUNEL assay did not reveal any differences in the number of apoptotic PVALB-positive interneurons in the forebrain of control and *Pvalb-atg5* animals, as illustrated in representative images of the somatosensory cortex and of the hippocampus (Figure S2I-K). Taken together, these results demonstrate that autophagy is not required for the survival of PVALB interneurons across the brain, while confirming that it is essential for cerebellar Purkinje cells. More generally, these observations suggest that distinct PVALB-expressing cell populations exhibit differential vulnerability to autophagy perturbation.

### Autophagy is required for organelle and synaptic protein homeostasis in PVALB interneurons

**Figure 2.**
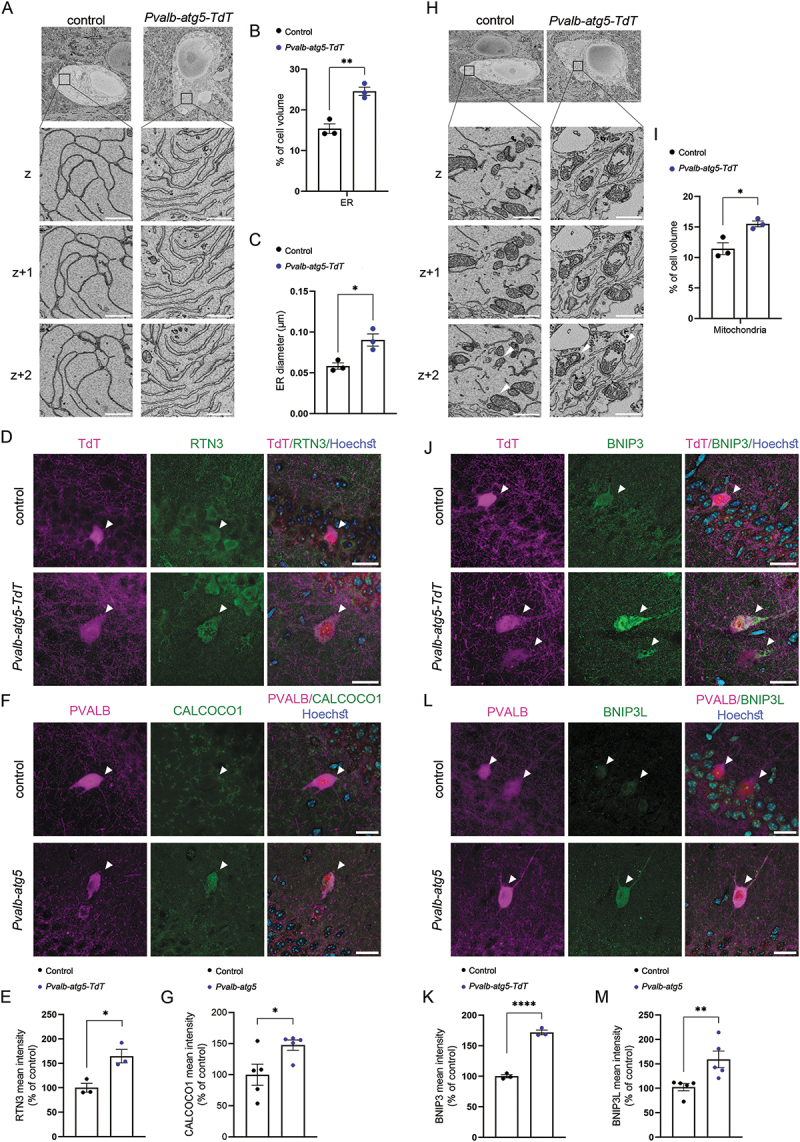
Autophagy regulates the proteostasis of PVALB interneurons. (A) Representative CLEM images of the endoplasmic reticulum of control (*Pvalb-TdT)* and *Pvalb-atg5-TdT* hippocampal PVALB interneurons. Scale bar: 5 μm. Insets show a higher magnification of the boxed areas. Inset scale bars: 1 µm. (B-C) Quantification of differences in cellular er volume (B) and diameter (C) in control and *Pvalb-atg5-TdT* PVALB cells. Statistical analysis was performed using unpaired, two-tailed Student’s t test (for er volume: **p = 0.0043, t = 5.849 and df = 4; for diameter: *p = 0.0189, t = 3.813 and df = 4). Bars represent mean values ± SEM. N = 3 animals per genotype. (D) Representative confocal images of TdT-positive PVALB cells (magenta) in the hippocampal CA1 area of control (*Pvalb-TdT*) and *Pvalb-atg5-TdT* animals, immunolabelled with antibodies against RTN3 (green) and stained with the nuclear dye Hoechst (blue). Scale bar: 20 µm. (E) Quantification of the mean RTN3 signal intensity in control versus *Pvalb-atg5-TdT* PVALB cells in hippocampal CA1. Results are expressed as a percentage of control values. Statistical analysis was performed using unpaired, two-tailed Student’s t test (*p = 0.0172, t = 3.926, df = 4). Bars represent mean values ± SEM. N = 3 animals per genotype. (F) Representative confocal images of PVALB cells in the hippocampal CA1 area of control (*Pvalb-Cre*) and *Pvalb-atg5* animals, immunolabelled with antibodies against PVALB (magenta) CALCOCO1 (green) and stained with the nuclear dye Hoechst (blue). Scale bar: 20 μm. (G) Quantification of the mean CALCOCO1 signal intensity in control versus *Pvalb-atg5* PVALB cells in hippocampal CA1. Results are expressed as a percentage of control values. Statistical analysis was performed using Mann-whitney U test (Mann-Whitney U = 2, n = 5 per group, *p = 0.0317). Bars represent mean values ± SEM. N = 5 animals per genotype. (H) representative CLEM images of mitochondria of control (*Pvalb-TdT)* and *Pvalb-atg5-TdT* hippocampal PVALB interneurons. Arrowheads indicate differences in mitochondrial morphology between controls (left) and *Pvalb-atg5-TdT* (right) cells. Scale bars represent 5 μm. Insets show a higher magnification of the boxed areas. Inset scale bars: 1 µm. (I) Quantification of differences in cellular mitochondrial volume in control and *Pvalb-atg5-TdT* PVALB cells. Statistical analysis was performed using unpaired, two-tailed Student’s t test (*p = 0.0193, t = 3.790, df = 4). Bars represent mean values ± SEM. N = 3 animals per genotype. (J) Representative confocal images of TdT-positive PVALB cells (magenta) in the hippocampal CA1 area of control (*Pvalb-TdT*) and *Pvalb-atg5-TdT* animals, immunolabelled with antibodies against BNIP3 (green) and stained with the nuclear dye Hoechst (blue). Scale bar: 20 µm. (K) Quantification of the mean BNIP3 signal intensity in control versus *Pvalb-atg5-TdT* PVALB cells in hippocampal CA1. Results are expressed as a percentage of control values. Statistical analysis was performed using unpaired, two-tailed Student’s t test (****p= < 0.0001, t = 15.76, df = 4). Bars represent mean values ± SEM. N = 3 animals per genotype. (L) representative confocal images of PVALB cells in the hippocampal CA1 area of control (*Pvalb-Cre*) and *Pvalb-atg5* animals, immunolabelled with antibodies against PVALB (magenta), BNIP3L (green) and stained with the nuclear dye Hoechst (blue). Scale bar: 20 µm. (M) Quantification of the mean BNIP3L signal intensity in control versus *Pvalb-atg5* PVALB cells. Results are expressed as a percentage of control values. Statistical analysis was performed using Mann-Whitney U test (Mann-Whitney U = 0, n = 5 per group, **p = 0.0079). Bars represent mean values ± SEM. N = 5 animals per genotype.

Given the fundamental role of autophagy in neuronal protein and organelle turnover, we then speculated that despite not compromising the survival of PVALB interneurons, impaired autophagy may perturb their homeostasis, resulting in direct or indirect impacts on their function. To test this hypothesis, we first performed correlative light and electron microscopy (CLEM) on TdT-positive PVALB interneurons in brain sections prepared from control and *Pvalb-atg5-TdT* mice. We then focused our analysis on the hippocampal CA1 region, where PVALB interneurons are well integrated into memory-related circuits. Stereology analysis on the resulting images revealed that *Pvalb-atg5-TdT* interneurons exhibited prominent and widespread dilation of the endoplasmic reticulum (Figures 2A-C), a hallmark of ER stress and dysfunction. To determine whether this phenotype resulted from impaired reticulophagy/ERphagy, we immunolabelled cryosections using antibodies against the selective reticulophagy receptors RTN3 (reticulon 3) and CALCOCO1. Confocal analysis of the CA1 area revealed a marked accumulation of these receptors specifically in PVALB interneurons lacking *Atg5* (Figures 2D-G), suggesting that autophagy is required for ER maintenance in these cells. Interestingly, our EM analyses further revealed an increased mitochondrial volume in *Pvalb-atg5-TdT* interneurons compared to controls (Figures 2H-I). Moreover, the supernumerary mitochondria often exhibited a damaged morphology, associated with impaired mitophagy [32], whereas such alterations were rarely observed in control neurons (Figure 2H, arrowheads). To decipher whether this stems from impaired mitochondrial turnover, we took advantage of antibodies against the selective mitophagy receptors BNIP3 and BNIP3L/NIX. Immunostaining and confocal imaging of *Pvalb-TdT* and *Pvalb-atg5-TdT* hippocampi revealed that both receptors presented with a significant accumulation in *Pvalb-atg5-TdT* interneurons, indicative of reduced mitophagy in the absence of *Atg5* (Figures 2J-M). Additionally, we examined whether the PINK1-PRKN mitophagy pathway is deregulated by loss of autophagy in PVALB interneurons. PINK1, a mitochondrial kinase, accumulates on damaged mitochondria and recruits the E3 ubiquitin ligase PRKN, which tags damaged mitochondria with ubiquitin marking them for autophagic degradation [33]. Using antibodies against PRKN, we didn’t detect any differences between genotypes, neither in the number nor in the intensity of PRKN-positive puncta, suggesting that this pathway is not affected by loss of autophagy (Figure S3A-C). This result is consistent with previous work demonstrating that under basal conditions mitophagy in brain cells occurs independently of the PINK-PRKN pathway and is instead facilitated by ubiquitin-independent and BNIP3/BNIP3L-dependent mitophagy pathways [34].

To determine the direct and indirect repercussions of autophagy deficiency on their proteome, we next FACS-sorted PVALB interneurons from the isocortex and hippocampus of three-month-old control (*Pvalb-TdT*) and *Pvalb-atg5-TdT* mice and compared them by quantitative proteomics. Principal component analysis indicated a clear segregation of the four control and four knockout replicates based on genotype (Figure S3D), and our mass-spectrometry analysis identified a total of 5963 proteins that were detected by at least two unique peptides (Table S2). To characterize the effects of autophagy ablation on the proteome of PVALB-interneurons, a p-value of 0.05 (-log10(0.05) = 1.3) was set as the cutoff for the identification of proteins differentially abundant between control and *Pvalb-atg5-TdT* samples. This criterion was based on the enrichment of the positive controls SQSTM1/p62 and WDFY3/ALFY (Figure S2F), two selective aggrephagy receptors that accumulate in *Pvalb-atg5-TdT* interneurons. In contrast, as expected, ATG5 significantly decreased in the *Pvalb-atg5-TdT* samples (Figure S3F), in line with its genetic ablation. Further to this, we found 413 proteins that were differentially expressed between the two genotypes, 249 of which were enriched and 164 were less abundant in the *Pvalb-atg5-TdT* cells when compared to the controls (Table S3, Figure S3E). Consistent with our EM and confocal analyses, proteins that were enriched in *Pvalb-atg5-TdT* samples included several integral ER membrane proteins, such as SACM1L, DERL1, DNAJC1 among others, as well as the reticulophagy SAR CALCOCO1 (Figure S3F). In total, we identified 245 ER and 248 Golgi proteins, representing 4.10% and 4.16% of the *Pvalb-atg5-TdT* cell proteome (Figure S3G) and covering 51.04% and 30.1% of the ER and Golgi proteomes, respectively (Figures S3I,J) [35]. Similarly, several mitochondrial proteins were also enriched in the *Pvalb-atg5-TdT* samples compared to control, including TOMM20, MT-ATP6, and MRPL11 (Figure S3F). We also observed an enrichment of 714 mitochondrial proteins, representing 11.97% of the total proteins detected in our analysis (Figure S3H), and 62.63% of the MitoCarta 3.0 mitochondrial protein database [36] (Figure S3K).

By further comparing our proteomic list with the SynGO database [37], we identified 882 synaptic proteins, representing 14.79% of all proteins detected (Figure S3G) and 55.05% of the entire synaptic proteome (Figure S3L). This subset did not include any neurotransmitter receptor subunits, which may be attributed to technical limitations imposed by FAC-sorting. Comparison of the differentially abundant proteins with the SynGO database indicated that 10 synaptic proteins were significantly downregulated in *Pvalb-atg5-TdT* interneurons (ATG16L1, WASF1, ATP2B4, CAMK4, FRRS1L, NOS1, PHB, RAB11, RPLP2 and VPS33B), whereas 30 synaptic proteins were significantly enriched (RPS6KC1, SACML1, ARHGAP32, SCG2, VCP, RPS10, PFN1, ROCK2, YWHAZ, PAFAH1B1, CAP1, YWHAH, CASK, STX2, GNB2, ATP2B1, ADD1, SQSTM1, RHEB, CAPN1, INPP4A, TUBB2B, SLC24A2, TBC1D2B, MIB1, TANC1, P2RX4, GNG4, RAB3B, SYNJ2) when compared to controls (Table S3). A cellular component analysis of the dysregulated synaptic proteins indicated that they are mainly localized to the postsynaptic specialization of asymmetric synapses, whereas some also showed a presynaptic localization (Figure S4A; schematically summarized in Figure S4B). The only downregulated synaptic protein involved in calcium homeostasis was ATP2B4, a plasma membrane calcium ATPase that localizes to the presynaptic membrane, which was found to be significantly decreased in autophagy-deficient PVALB interneurons, as confirmed by Jess Simple Western™ analysis (Figures S4C,D).

**Figure 3.**
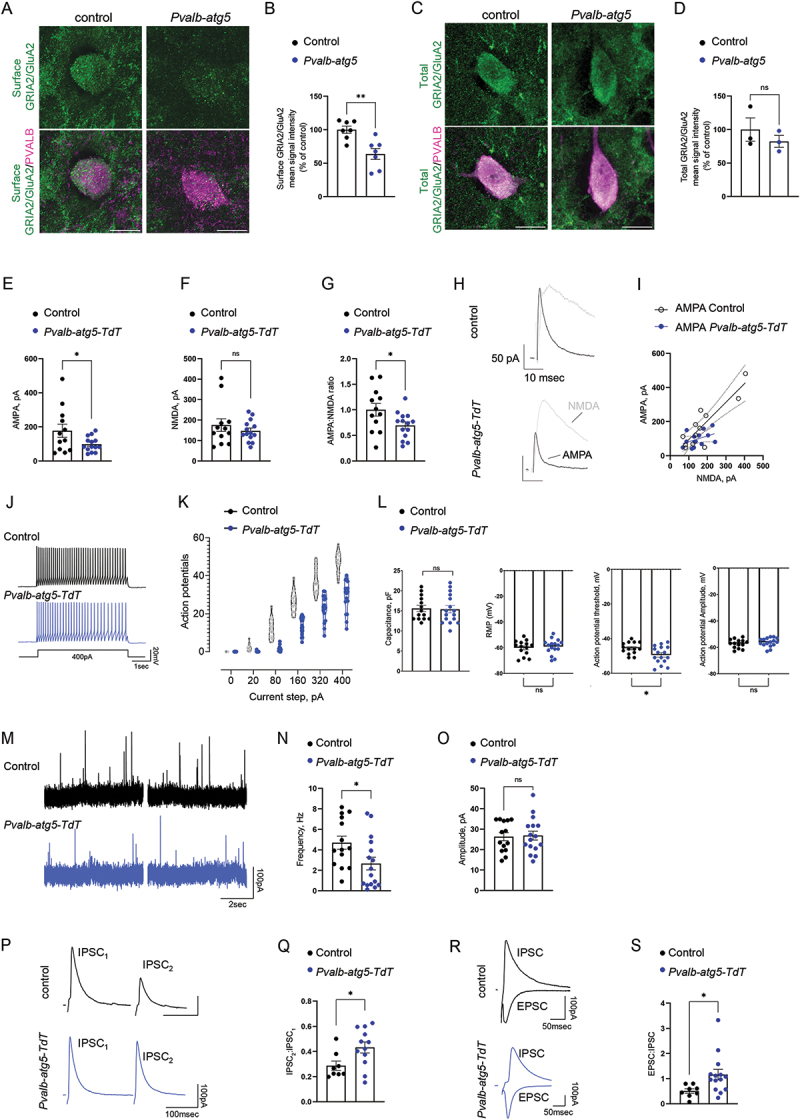
Autophagy-deficient PVALB-interneurons exhibit an altered synaptic composition, resulting in functional adaptations. (A) Representative images of surface GRIA2/GluA2 immunoreactivity (green) in hippocampal CA1 PVALB cells (magenta) from control (*Atg5*^*f/f*^) vs. *Pvalb-atg5* mice. Scale bar: 10 µm. (B) Quantification of the mean surface GRIA2/GluA2 intensity in control versus *Pvalb-atg5* PVALB cells in hippocampal CA1. Results are expressed as a percentage of control values. Statistical analysis was performed using an unpaired, two-tailed Student’s t test (**p = 0.0028, *t* = 3.737, df = 12). Bars represent mean values ±SEM. *N* = 7 mice per genotype. (C) Representative images of total GRIA2/GluA2 immunoreactivity (green) in hippocampal CA1 PVALB cells (magenta) from control (*Atg5*^*f/f*^) vs. *Pvalb-atg5* mice. Scale bar: 10 µm. (D) Quantification of the mean total GRIA2/GluA2 intensity in control versus *Pvalb-atg5* PVALB cells in hippocampal CA1. Results are expressed as a percentage of control values. Statistical analysis was performed using an unpaired, two-tailed Student’s t test (^ns^*p* = 0.4154, *t* = 0.9076, df = 4). Bars represent mean values ±SEM. *N* = 3 mice per genotype. (E-I) Patch clamp recordings of AMPA (E) and NMDA (F) currents from CA1 PVALB cells from control (*Pvalb-TdT*) and *Atg5*-deficient PVALB interneurons (*Pvalb-atg5-TdT*). These data were also plotted as the AMPA:NMDA ratio for controls and *Pvalb-atg5-TdT* animals, where all values were normalized to the control (G). (H) represents the average AMPA (black) and NMDA (gray) traces recorded at + 40 mV for control and *Pvalb-atg5-TdT* cells. (I) Reduced AMPA currents over NMDA currents in *Pvalb-atg5-TdT* mice when compared to controls. Black line indicates R^2^ = 1. Statistical analyses were performed using a Student’s t test (for AMPA currents: *p = 0.0464, *t* = 2.100, df = 24; for NMDA currents, ^ns^*p* = 0.3955, *t* = 0.8651, df = 24; for AMPA:NMDA ratios: *p = 0.0348, *t* = 2.238, df = 24). Bars represent mean values ± SEM. *N* = 3 animals per genotype (*Pvalb-TdT, n* = 14; *Pvalb-atg5-TdT*, *n* = 16). (J) Current-clamp sample traces (400 pA injection, top) and the action potentials evoked by current steps (bottom) from PVALB neurons, from slices of control *(Pvalb-TdT*, RMP = −63 mV) and mutant *Pvalb-atg5-TdT* (RMP = −62 mV) mice. (K) Input-output plots from control *(Pvalb-TdT)* (*n* = 14, *N* = 3) and *Pvalb-atg5-TdT* cells (*n* = 16, *N* = 3). Statistical analysis was performed using a two-way repeated measures ANOVA followed by Sidak post hoc test; F(5,140) = 23.85 (****p < 0.005). Bars represent mean values ± SEM. (L) Comparison of capacitance, resting membrane potential, action potential threshold and amplitude in control *(Pvalb-TdT)* (*n* = 14, *N* = 3) and *Pvalb-atg5-TdT* cells (*n* = 16, *N* = 3). Statistical analyses were performed using a Student’s t test (ap threshold, *p = 0.023, *t* = 2.398, df = 28). Bars represent mean values ± SEM. (M) Representative traces of sIPSC from slices of control *(Pvalb-TdT)* and mutant *Pvalb-atg5-TdT* mice recorded at + 5 mV. (N-O) Bar graphs and cumulative plots related tothe frequency and amplitude of sIpscs from control *(Pvalb-TdT)* (*n* = 14, *N* = 3) and mutant *Pvalb-atg5-TdT* mice (*n* = 16, *N* = 3). Statistical analysis was performed using an unpaired, two-tailed Student’s t test (*p = 0.0298, *t* = 2.289, df = 28 and ^ns^*p* = 0.8654, *t* = 0.1710, df = 28, respectively). Bars represent mean values ± SEM. (P) Representative traces of paired-pulse ratio (IPSC_2_:IPSC_1_) from three-month old control *(Pvalb-TdT)* and mutant *Pvalb-atg5-TdT* mice recorded at + 5 mV. (Q) Graph showing the paired-pulse ratio (IPSC_2_:IPSC_1_) from control *(Pvalb-TdT)* (*n* = 8, *N* = 2) and mutant *Pvalb-atg5-TdT* mice (*n* = 12, *N* = 2). Statistical analysis was performed using an unpaired, two-tailed Student’s t test (*p = 0.0310, *t* = 2.340, df = 18). Bars represent mean values ± SEM. (R) Representative traces of excitation to inhibition ratio (EPSC_−60 mV_:IPSC _+5 mV_) from control *(Pvalb-TdT)* and mutant *Pvalb-atg5-TdT* mice. (S) Graph showing the excitation to inhibition ratio (EPSC:IPSC) from control *(Pvalb-TdT)* (*n* = 8, *N* = 2) and mutant *Pvalb-atg5-TdT* (*n* = 14, *N* = 3). Statistical analysis was performed using an unpaired, two-tailed Student’s t test (*p = 0.0351, *t* = 2.261, df = 20). Bars represent mean values ± SEM.

Another protein that was significantly downregulated in *Pvalb-atg5-TdT* interneurons is FRRS1L, an outer component of AMPA receptors [38,39]. With recent work indicating that loss of FRRS1L leads to intracellular retention of GRIA2/GluA2 subunits [38], we compared the surface expression of GRIA2/GluA2 in hippocampal sections of control and *Pvalb-atg5* mice. In line with reduced FRRSL1 levels, we found that autophagy-deficient PVALB interneurons also presented with a significant reduction in surface GRIA2/GluA2 (Figures 3A, B), while the total levels remained unchanged (Figures 3C, D). By contrast, neither surface nor total levels of the AMPA receptor subunit GRIA1/GluA1 or of the NMDA receptor subunit GRIN2A/NR2A were affected by loss of autophagy (Figures S4E-L). Taken together, these findings indicated that autophagy-deficiency leads to changes in neurotransmitter receptor composition on the membrane of PVALB interneurons.

We then tested whether the changes in synaptic proteostasis have repercussions on PVALB interneuron function, subsequently affecting the networks in which they are embedded. To test this, we focused on the hippocampal CA1 region, where PVALB expression is restricted to inhibitory interneurons, mostly comprising of basket cells [40,41]. To examine whether *Atg5* deletion influences their synaptic and excitability properties, we performed patch clamp recordings from acute brain slices of control (*Pvalb-TdT*) and *Pvalb-atg5-TdT* mice. Measurements of synaptic AMPA and NMDA currents showed a marked reduction in AMPA to NMDA ratios in autophagy-deficient interneurons, indicating a reduced strength of AMPAR-mediated neurotransmission (Figures 3E-I). Furthermore, a reduced frequency in action potential firing and the rightward shift in input-output curves recorded from *Pvalb-atg5-TdT* interneurons indicate that *atg5* ablation results in impaired excitability of this population (Figures 3J, K). This occurred with a reduced action potential threshold, while capacitance, resting membrane potential (RMP) and action potential amplitude remained comparable across groups (Figure 3L).

Next, we examined whether the reduced PVALB interneuron synaptic excitation and excitability has repercussions on GABAergic neurotransmission impinging onto neighboring pyramidal cells (PC) in the CA1 region. Recording of spontaneous inhibitory postsynaptic currents (sIPSCs) of PCs (Figure 3M) revealed a reduction in the frequency, but not amplitude of events (Figures 3N,O, respectively), concomitantly with an increased paired-pulse ratio of evoked IPSCs (Figures 3P, Q). Additionally, we probed both the excitatory (−60 mV, EPSC) and inhibitory synaptic transmission (+5 mV, IPSC) which revealed an enhanced EPSC to IPSC ratio (EPSC:IPSC) in PCs from *Pvalb-atg5-TdT* mice compared to control littermates (Figures 3R, S). Taken together, these results support a diminished PVALB neuronal excitability, which accounts for a reduction in synaptic inhibition on PCs in the CA1 region of the hippocampus.

The enhanced excitation:inhibition ratio on PCs urged us to further investigate the presence of spontaneous epileptic seizures, as observed in mice with a conditional ablation of autophagy in forebrain excitatory neurons [7,42] and human patients with mutations in autophagy genes [43]. To this end, we performed frontal and parietal electroencephalogram (EEG) recordings in combination with electromyogram (EMG) recordings in control (N*=*8 mice) and *Pvalb-atg5* (*N* = 7 mice) animals for three consecutive light-dark phases. Animals cycled between the three vigilance states of wake, non-rapid-eye-movement sleep (NREM) and rapid-eye-movement sleep (REM), as evident by standard spectral EEG signatures. Analysis of these recordings using the Epi-AI online peer-reviewed tool [44] confirmed the absence of overt spontaneous epileptic seizures in the *Pvalb-atg5* mice (Figure S4M), indicating that perturbed autophagy in PVALB-positive cells alone is not sufficient to induce spontaneous epileptic phenotypes, yet not excluding the possibility that these animals may be more susceptible to pharmacologically induced seizures.

**Figure 4.**
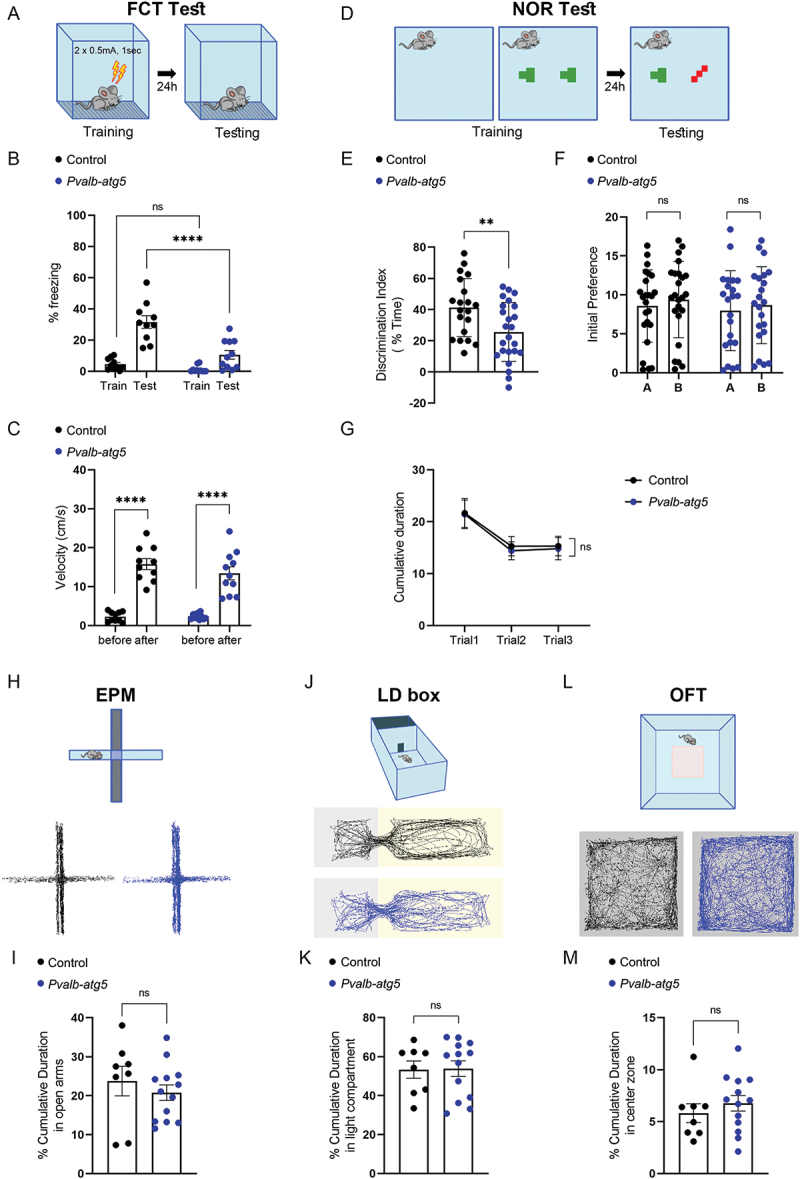
Animals with autophagy deficiency in PVALB-expressing neurons exhibit deficits in memory. (A) Graphical representation of the experimental pipeline for the fear conditioning test (FCT). (B-C) The percentage of freezing responses (b) and the average speed (c) of control (*Atg5*^*f/f*^) and *Pvalb-atg5* littermates in the FCT test. Statistical analyses were performed using a two-way repeated measures ANOVA followed by Sidak post hoc test; F (1,19) = 13.17, (train day ^ns^*p* = 0.5732 and test day ****p < 0.0001) and F (1,19) = 1.255, (before and after in control ****p < 0.0001 and in *Pvalb-atg5* ****p < 0.0001) respectively. Bars represent mean values ± SEM. *N* = 10 and 11 animals for control and *Pvalb-atg5*, respectively. (D) graphical representation of the experimental pipeline for the novel object recognition test (nor). (E-G) the discrimination index (DI) on testing day (e), the initial object preference (f), and the cumulative duration (g) spent investigating objects per trial. DI was calculated by the following formula: DI=[time in novel object-familiar one)/total time in objects] × 100%. Statistical analyses were performed using an unpaired, two-tailed Student’s t test (**p = 0.0088, *t* = 2.747, df = 42) for DI and two-way repeated measures ANOVA followed by Sidak post hoc test for the other two analyses (for initial preference F(1,80) = 0.08937, for control ^ns^*p* = 0.6333 and *Pvalb-atg5* ^ns^*p* = 0.8728) and (for cumulative duration F (2,88) = 0.02500, for trial 1: ^ns^*p* = 0.9999, for trial 2: ^ns^*p* = 0.9815 and for trial 3: ^ns^*p* = 0.9974 respectively). Bars represent mean values ± SEM. *N* = 20 and *N* = 24 animals for control and *Pvalb-atg5*, respectively. (H) overview of the experimental pipeline for elevated plus maze test (EPM) and representative tracking traces from one control (*Atg5*^*f/f*^) and one mutant *Pvalb-atg5*. (I) the percentage of cumulative duration in open arms of control (*Atg5*^*f/f*^) and *Pvalb-atg5* mutants during the EPM test. Statistical analysis was performed using an unpaired, two-tailed Student’s t test (^ns^*p* = 0.4565, *t* = 0.7602, df = 19). Bars represent mean values ± SEM. *N* = 8 and 13 animals for control and *Pvalb-atg5*, respectively. (J) overview of the experimental pipeline for the light dark box test (LD box) and representative tracking traces from one control (*Atg5*^*f/f*^) and one mutant *Pvalb-atg5* mouse. (K) the percentage of the cumulative duration spent by control (*Atg5*^*f/f*^) and *Pvalb-atg5* littermates in the light compartment during the LD box test. Statistical analysis was performed using an unpaired, two-tailed Student’s t test (^ns^*p* = 0.94120.4565, *t* = 0.07473, df = 19). Bars represent mean values ± SEM. *N* = 8 and 13 animals for control and *Pvalb-atg5*, respectively. (L) graphical overview of the experimental pipeline for open field test (oft) and representative tracking traces from one control (*Atg5*^*f/f*^) and one mutant *Pvalb-atg5* mouse. (M) the percentage of the cumulative duration spent in the central zone by control (*Atg5*^*f/f*^) versus *Pvalb-atg5* littermates during the oft test. Statistical analysis was performed using an unpaired, two-tailed Student’s t test (^ns^*p* = 0.4358, *t* = 0.7961, df = 19). Bars represent mean values ± SEM. *N* = 8 and 13 animals for control and *Pvalb-atg5*, respectively.

As PVALB interneuron activity has been implicated in memory [45–47] and anxiety [48], we then sought to determine whether impaired autophagy affects cognition via changes in PVALB interneuron excitability. To this end, control and *Pvalb-atg5* mice were subjected to a fear conditioning test, a paradigm of associative memory (see Materials and Methods, summarized in Figure 4A). While control and *Pvalb-atg5* animals exhibited comparable freezing percentages during training, *Pvalb-atg5* animals exhibited a significant reduction in freezing on the test day when compared to control littermates (Figure 4B). Differences in freezing were not attributed to an impaired ability of *Pvalb-atg5* animals to perceive the stimulus, since both genotypes showed a significant increase in reactivity upon shock delivery (Figure 4C).

To further probe the involvement of autophagy in the regulation of memory processes, control and *Pvalb-atg5* mice were subjected to a novel object recognition test, a paradigm of recognition memory (see Materials and Methods, summarized in Figure 4D). The difference in time spent with the familiar or the novel object is measured as a discrimination index (DI) and is a proxy for the memory of the familiar object. *Pvalb-atg5* mice exhibited a significant decrease in their discrimination index compared to control littermates, indicating a defective memory for the familiar object (Figure 4E). This deficit was neither due to differences in initial preference (Figure 4F) nor to differences in the cumulative duration spent exploring the objects (Figure 4G). Taken together, these findings indicate that autophagy deficiency in PVALB neurons is sufficient to induce profound deficits in both associative and recognition memory.

Lastly, to determine whether autophagy-deficient PVALB neurons impact anxiety-like behaviors, we tested control and *Pvalb-atg5* mice in three anxiety tests: 1) the elevated plus maze, 2) the light/dark box, and 3) the open field test. In all three tests, no significant differences were observed in the cumulative duration that *Pvalb-atg5* animals spent in the open arm (Figures 4H, I), in the light compartment (Figure 4J, K) or in the center of the arena (Figure 4L, M) when compared to control littermates. Moreover, the total locomotion of control and *Pvalb-atg5* animals was comparable in all three tests (Figures S4N-P). Taken together, these findings suggest that the described proteostatic and synaptic deficits of autophagy-deficient PVALB neurons, as well as of the networks in which they take part, do not culminate into changes in anxiety-like behaviors.

## Discussion

Emerging evidence from animal models suggests that the well-conserved autophagic machinery, which is fundamental for the turnover of proteins and organelles, is implicated in higher order behaviors that depend on the orchestrated function of multiple neuronal populations. However, our knowledge of the roles of autophagy at cellular and synaptic levels is thus far limited to glutamatergic and other projection neurons. Here we expanded this knowledge to other neuronal populations, demonstrating that autophagy regulates the homeostasis and physiology of PVALB-expressing interneurons, safeguarding hippocampal network dynamics and memory.

Although the physiological roles of autophagy in interneurons were previously overlooked, interneurons have already been implicated in the pathology of congenital diseases of autophagy, such as tuberous sclerosis complex (TSC), a disorder that manifests with severe synaptic and psychiatric deficits, including memory deficits [49], epilepsy, developmental delay, and autism [50]. In this disorder, autosomal dominant germline mutations in *TSC1* or *TSC2* genes lead to the hyperactivation of the MTORC1 pathway, an important positive regulator of protein translation and negative regulator of autophagy. Interestingly, mice with a conditional ablation of *Tsc1* in GABAergic interneuron progenitor cells [51] exhibit reduced numbers of GABAergic cells and synapses in the forebrain. Here, we directly tested whether autophagy regulates PVALB-interneuron numbers and whether it safeguards their maintenance in the brain but found no evidence for such a role. Instead, across all tested brain areas, PVALB-interneuron numbers were comparable between control mice and those lacking *Atg5* conditionally in PVALB expressing neurons. Therefore, we conclude that autophagy is dispensable for the survival and maintenance of postmitotic PVALB interneurons. We speculate that the interneuron loss observed in the *tsc1*-conditional knockout model may be due to the concomitant dysregulation of the translation machinery. Alternatively, it may also be due to the fact that ablation of *Tsc1* starts embryonically in GABAergic neuron progenitors, consistent with previous work showing that MTOR signaling regulates the early proliferation of these cells [52].

In this study, we directly examined the requirement of autophagy for neurotransmission and excitability, focusing on PVALB-expressing interneurons of the hippocampus. We report that autophagy-deficient PVALB interneurons are less excitable compared to controls. Consistently, we also found reduced inhibitory input to neighboring excitatory neurons of the hippocampus of *Pvalb-atg5* animals, compared to control littermates. Together, these findings demonstrate that autophagy-deficient PVALB interneurons exhibit reduced basal transmission, which accounts for an imbalance between excitation and inhibition in the hippocampus. Our findings help explain previous work, showing that loss of *Tsc1* reduces inhibitory synaptic transmission [53,54] and results in fewer and smaller synapses formed by PVALB interneurons, which are responsible for social deficits [55]. As synaptic deficits of PVALB-interneurons are likely to also pertain to human TSC patients, restoring autophagy specifically in these cells may represent a novel strategy for reinstating inhibitory transmission and network balance, to ameliorate some aspects of the cognitive impairment pathology, such as memory, learning and sociability.

A recent study reported that autophagy-deficient excitatory glutamatergic neurons exhibit enhanced basal transmission, which was attributed to the failed turnover of presynaptic ER and the consequent elevation of intracellular calcium stores [4]. Taken together with our findings, it emerges that under basal conditions, autophagy regulates excitatory and inhibitory neurotransmission in diametrical ways, restricting the former, yet enhancing the latter. However, we cannot exclude that calcium levels aren’t implicated in the reduced inhibitory transmission of autophagy-deficient PVALB interneurons, given that our EM and proteomic analyses revealed marked impairments to ER turnover in the absence of *Atg5*, not only presynaptically but also in the PVALB interneuron somatodendritic area. Indeed, changes to calcium homeostasis in *Pvalb-atg5* may also be exacerbated by reduced levels of ATP2B4, a P-type plasma membrane calcium ATPase (PMCA) localized to the presynaptic membrane [56]. We also found a significant reduction in FRRS1L and WASF1, both of which are involved in the plasma membrane retention and targeting of AMPA and NMDA receptor subunits, respectively [38,39]. This led us to reveal that autophagy indirectly regulates the surface levels of GRIA2/GluA2 in PVALB interneurons, resulting in a marked reduction in AMPA/NMDA current ratios. We cannot exclude the possibility that autophagy may additionally regulate surface expression of GRIA2/GluA2 levels via non-degradative functions. Taken together, these findings invite the speculation that these alterations collectively contribute to the reduced excitability of these neurons when autophagy is impaired. More generally, it also appears that the autophagic machinery sequesters and turns over distinct cellular substrates in different neuronal populations, adding yet another aspect of selectivity to its contribution to protein and organelle homeostasis.

In addition, we found that *Pvalb-atg5* animals exhibit deficits in associative and recognition memory, two major components of declarative memory that mainly involve the hippocampus, among other brain areas. Previous work has clearly shown that conditional deletion of AMPA or NMDA receptor subunits in PVALB neurons causes reduced excitability of fast-spiking PVALB interneurons in the hippocampus, leading to impaired hippocampal network synchrony and memory deficits [57,58]. Consistently, pharmacogenetic inhibition of hippocampal PVALB expressing interneurons impairs hippocampal network dynamics, leading to deficits in the consolidation of associative memory [46]. Therefore, we believe that the memory deficits of *Pvalb-atg5* animals are attributed to the diminished activity of PVALB interneurons in the hippocampus and not related to the loss of the cerebellar Purkinje cells, which are instead involved in implicit memories, including procedural memories and motor learning [21].

By contrast, anxiety was not impaired in *Pvalb-atg5* animals, which is not surprising given the complex role of PVALB interneurons in anxiety, involving multiple brain areas and networks. For example, one study showed that chronic activation of PVALB interneurons cells in the prefrontal cortex leads to increased anxiety-related behaviors in female mice only [22]. By contrast, in another study, it was shown that optogenetically inhibiting PVALB interneurons in the ventral hippocampus induces persistent anxiety [59]. Therefore, we speculate that the synaptic changes caused by autophagy deficiency are not sufficient to perturb the anxiety-relevant networks where PVALB interneurons are embedded and to cause a manifestation of an anxiety phenotype. As a note, all our behavioral experiments were performed in male mice, therefore not excluding the possibility of female-specific effects in anxiety phenotypes upon loss of autophagy in PVALB interneurons.

In conclusion, it appears that the requirement of autophagy for proper brain function relies on cell-type specific functions in different neuronal subpopulations. Here, we have highlighted the distinct roles played by autophagy in PVALB-expressing neurons. Whether autophagy regulates the neurotransmission of other types of inhibitory neurons, such as those expressing somatostatin, calretinin, VIP or other markers, remains to be investigated by future studies to gain a complete picture on the collective impact of autophagy impairment on multi-neuronal networks.

## Materials and Methods

### Animal husbandry

The animal protocols of this study were approved by the Animal Ethics Committee of the Foundation for Research and Technology Hellas (FORTH) and by the Swiss veterinary services of the Canton Vaud (Direction generale de l’agriculture, de la viticulture et des affaires veterinaires; licenses VD3460, VD3461). Special attention was paid to the implementation of the 3Rs, housing conditions and analgesia, for assurance of animal welfare throughout.

Mice were housed in Individually Ventilated Cages (IVCs; Innovive, France) in single-sex groups of 3–5 per cage. Water and food were provided *ad libitum* with constant temperature (22°C), humidity (55%), air changes (60 per h) and a 12 h light/dark cycle (07:00 on; 19:00 off), except for EEG recordings where the cycle was shifted by 2 h (09:00 on; 21:00 off). All behavioral assays were performed between 10:00 and 15:00.

For all experiments, mouse lines were maintained on a C57BL/6 genetic background for at least 20 generations. To generate mice lacking *Atg5* in PVALB interneurons, we crossed floxed *Atg5* mutants (*Atg5*^*f/f*^) [3] with the Cre-recombinase line *Pvalb-Cre*^*±*^ (B6;129P2-*Pvalb*^tm1(cre)Arbr^/J; strain 008069, The Jackson Laboratory) [25], yielding *Pvalb-cre*^*±*^;*atg5*^*f/f*^ progeny (hereafter referred to as *Pvalb-atg5)*. To label Cre-expressing PVALB cells, we also crossed this line with the Cre-reporter *tdTom*^*tg/+*^ (*TdT*) (B6.Cg-*Gt(ROSA)26Sor*^*tm9(CAG-tdTomato*)*Hze*^/J; strain 007909, The Jackson Laboratory) [28], to generate *Pvalb-Cre*^*±*^;*Atg5*^*f/f*^;*tdTom*^*tg/+*^ (*Pvalb-atg5-TdT)* offspring. For all experiments, *Atg5*^*f/f*^ and *Pvalb-cre*^*±*^;*Atg5*^*+/+*^;*tdTom*^*tg/+*^ *(Pvalb-TdT)* littermates were used as controls. Male *Pvalb-cre*^*±*^ mice were excluded from all breeding pairs to exclude the possibility of unwanted germline recombination and global recombination due to the low expression of PVALB in sperm. Unless otherwise stated, all experiments were performed on male mice at approximately three months-of-age.

### Western blotting

Western blots were performed as previously described [60] with minor modifications. Briefly, tissues were lysed in RIPA Buffer (50 mM Tris-HCl [Roth, 9090.2] pH 7.4, 150 mM NaCl [VWR Chemicals, 27,800.291], 1 mM EDTA [Sigma-Aldrich, 1,084,180,250], 1% Triton X-100 (Merck, 1,086,031,000), 0.1% SDS (Sigma-Aldrich, 822,050) and 0.1% sodium deoxycholate (Sigma-Aldrich, 30,970) supplemented with Roche cOmplete™ Mini, EDTA-free Protease Inhibitor Cocktail (Merck, 11,836,170,001). Tissues were sonicated for 20 s on ice and centrifuged twice for 30 min at 16,200 ×g to precipitate DNA. Protein concentrations were then determined using a Pierce™ BCA Protein Assay Kit (ThermoFisher Scientific, 23,225) according to the manufacturer’s instructions. Proteins were separated using 4–20% polyacrylamide gels (Bio-Rad, 4,561,093) and transferred onto 0.2-µm nitrocellulose membranes (Cytiva, 1,060,004). Total protein levels were visualized using the No-Stain™ Protein Labeling Reagent (Invitrogen, A44449) according to the manufacturer’s instructions. Membranes were then blocked in 5% skimmed milk for 1 h at room temperature (RT) and then incubated in the primary antibody overnight at 4°C. The next day, the membranes were washed three times for 10 min with PBS-T (137 mM NaCl, 2.7 mM KCl (Merck, P4504), 10 mM Na_2_HPO_4_ [Merck, 1.06586.1000], 1.8 mM KH_2_PO_4_ [Sigma-Aldrich, P5379], 0.1% Tween-20 [Sigma-Aldrich, P9416], pH 7.4) and incubated with a secondary horseradish peroxidase (HRP)-conjugated antibody (see Table 1) for 1 h at room temperature. Membranes were then washed three times for 10 min with PBS-T and incubated with SuperSignal West Pico™ or Femto™ Chemiluminescent Substrate (ThermoFisher Scientific, 34,580 and 34,095, respectively) according to the manufacturer’s instructions. Working dilutions of primary and secondary antibodies used for western blot are listed in Table 1. Resulting bands were quantified using the “Analyzing Gels” module of FIJI/ImageJ [61]. Protein levels were calculated as the optical density ratio (OD) of the band of interest/total protein levels, with all data normalized to the mean of the control group.

**Table 1.** List of antibodies used in Jess™ Simple Western, western blot and immunohistochemistry experiments.

Antibody	Species	Company	Cat #	Dilution (procedure)
**Primary Antibodies**				
Active-CASP3/caspase 3	Rabbit	R&D Systems	AF835	1:200 (IHC)
ATP2B4	Rabbit	Sigma-Aldrich	SAB2100184	1:500 (WB)
WDFY3/ALFY [B-4]	Mouse	Santa Cruz Biotechnologies	sc-514569	1:1000 (WB)
TUBB3/β3-Tubulin/Tuj1	Mouse	Santa Cruz Biotechnology	80,005	1:5000 (WB, IHC)
TUBB3/β3-Tubulin/Tuj1	Rabbit	Abcam	ab18207	1:1000 (WB, IHC)
BNIP3 [EPR4034]	Rabbit	Abcam	ab109362	1:100 (IHC)
BNIP3L	Rabbit	Proteintech	12,986–1-AP	1:100 (IHC)
CALB/calbindin D28K	Rabbit	Swant	CB-38a	1:5000 (IHC)
CALCOCO1 [A-10]	Mouse	Santa Cruz Biotechnology	sc-515670	1:50 (IHC)
GABRG2 subunit	Rabbit	Synaptic Systems	224,003	1:1000 (WB, IHC)
GAD2/GAD65	Mouse	Synaptic Systems	198,111	1:500 (WB, IHC)
GPHN (gephyrin)	Mouse	Synaptic Systems	SYSY147021	1:500 (WB, IHC)
GRIA1/GluA1 [N355/1] N-terminal	Mouse	Abcam	ab174785	1:500 (IHC)
GRIA2/GluA2 C- terminus	Mouse	Synaptic Systems	182,211	1:100 (WB)
GRIA2/GluA2 (extracellular)	Guinea pig	Alomone labs	AGC-005-GP	1:500 (IHC)
MAP1LC3B	Rabbit	Sigma-Aldrich	L7543	1:1000 (WB)
MAP1LC3B	Mouse	Santa Cruz Biotechnology	271,625	1:1000 (WB)
GRIN2A/NR2A	Rabbit	Alomone Labs	AGC-002	1:500 (WB, IHC)
PRKN	Rabbit	Sigma-Aldrich	P5748	1:1000 (IHC)
PVALB/Parvalbumin	Mouse	Swant	235	1:1000 (WB), 1:500 IHC)
PVALB/Parvalbumin	Rabbit	Swant	PV27	1:2000 (WB), 1:500 IHC)
RFP	Rabbit	Rockland Immunochemicals	42,896	1:1000 (WB, IHC)
RTN3	Rabbit	Proteintech	12,055–2-AP	1:100 (IHC)
SQSTM1/p62	Mouse	Calbiochem Research Biochemicals	DR1057	1:5000 (WB)
SQSTM1/p62	Guinea pig	PROGEN	GP62-C	1:2000 (IHC)
**Secondary antibodies**				
Goat anti-Mouse IgG (Alexa Fluor) 488	Mouse	Abcam	ab150113	1:1000 (IHC)
Goat anti-Rabbit IgG H&L (Alexa Fluor) 488	Rabbit	Abcam	ab150077	1:1000 (IHC)
Goat anti-Mouse IgG H&L (Alexa Fluor) 594	Mouse	Abcam	ab150116	1:1000 (IHC)
Goat anti-Guinea Pig IgG H&L (Alexa Fluor) 647	Guinea pig	Abcam	ab150187	1:1000 (IHC)
Goat anti-Mouse IgG H&L (Alexa Fluor) 647	Mouse	Abcam	ab150115	1:1000 (IHC)
Donkey anti-Rabbit IgG H&L (Alexa Fluor) 647	Rabbit	Abcam	Ab150075	1:1000 (IHC)
Anti-mouse IgG (Dylight 800 4xPEG)	Mouse	Invitrogen	SA5-35521	1:10000 (WB)
Peroxidase AffiniPure Mouse anti-Rabbit IgG (H+L)	Rabbit	Jackson ImmunoResearch Europe Ltd.	211–035-109	1:10000 (WB)
Peroxidase AffiniPure Donkey Anti-Mouse IgG (H+L)	Mouse	Jackson ImmunoResearch Europe Ltd.	715–035-150	1:10000 (WB)
ProteinSimple Anti-Rabbit Detection Module	Rabbit	Bio-techne	DM-001	1X, (Jess™ Simple Western)
ProteinSimple Anti-Mouse Detection Module	Mouse	Bio-techne	DM-002	1X, (Jess™ Simple Western)

### Tissue fixation and cryo-protection

Mice were transcardially perfused with ice-cold 4% PFA (4% w:v, Roth, 0335.5) in PBS pH 7.4) and then post-fixed for 3 h at 4°C. Prior to cryosectioning, tissues were cryoprotected in 30% sucrose solution (30% w:v sucrose [Sigma-Aldrich, 1,076,875,000] in PBS), embedded in Tissue Tek O.C.T. Compound (Sakura, 4583), frozen on dry ice, and stored at −80°C. During cryosectioning, all brains were cut into coronal or sagittal sections of 16 μm and embedded onto Superfrost Plus™ slides (Fisher Scientific, 15,438,060). Post-embedding, tissue slices were dried at 37°C for 2–3 h and then stored long-term at −80°C.

### TUNEL cell death assay

The presence of apoptosis in control (*Pvalb-TdT*) and *Pvalb-atg5-TdT* 20-µm cryosections were assessed by *in situ* terminal deoxynucleotidyl transferase nick end-labeling (TUNEL) using the *In Situ* Cell Detection Kit (Roche, 11,684,795,910). In line with the manufacturer’s instructions, both positive- and negative control samples were prepared for each experimental replication, with the latter used for thresholding during quantification (see below).

### Immunostaining

#### Immunohistochemistry on cryosections

Cryosections (16 - 20 µm thick) prepared from control (*Pvalb-Cre* or *Pvalb-TdT*) and *Pvalb-atg5* (*Pvalb-atg5* or *Pvalb-atg5-TdT)* were first rehydrated in 1 ×PBS for 1 h and then incubated in blocking solution (10% FBS [ThermoFisher Scientific, 10,500–064] and 0.3% Triton X-100 in PBS pH 7.4) for 1 h at RT. Sections were then incubated in blocking solution containing primary antibodies (listed in Table 1) overnight at 4°C. To test for nonspecific labeling, blocking solution without primary antibody was applied in parallel onto separate samples. The next day, sections were rinsed three times for 5 min in PBS and incubated with secondary antibodies (see Table 1) in 10% FBS in 1x PBS pH 7.4 and the nuclear dye Hoechst (1:5000) (Hello Bio, HB0787) for 1 h at room temperature. Tissues were then rinsed three times for 5 min in PBS and coverslipped using Fluoroshield Mounting Medium (abcam, ab104135).

#### Surface and total GRIA1/GluA1, GRIA2/GluA2 and GRIN2A/NR2A labeling

Free-floating 70 µm brain sections from control (*Atg5*^*f/f*^) and mutant *Pvalb-atg5* mice were washed 3 ×in PBS for 10 min. For total receptor labeling, sections were incubated in blocking solution (see above) for 1 h at RT, while surface labeling was performed using a non-permeabilizing block (10% FBS in PBS, pH 7.4) for the same duration. Sections were then incubated overnight at 4°C in the respective blocking solutions containing primary antibodies (as listed in Table 1; both anti-PVALB and anti-GRIA1/GluA1, GRIA2/GluA2 or GRIN2A/NR2A for total receptor labeling, and exclusively anti-GRIA1/GluA1, GRIA2/GluA2 or GRIN2A/NR2A for surface labeling). The following day, all sections were washed 3 ×in PBS and incubated for 1 h at RT with secondary antibodies (see Table 1) in non-permeabilizing block together with Hoechst nuclear dye (1:5000). After 3 ×PBS washes, total receptor-labeled sections were mounted on SuperFrost slides (Fisher Scientific, 15,438,060) using Dako mounting medium (Agilent Technologies, S302380-2). For surface labeling, after the first application of secondary antibodies, sections were immediately fixed in 1% PFA in PBS (pH 7.4) for 8 minutes at RT, washed three times in PBS, and incubated overnight at 4°C with anti-PVALB antibodies in permeabilizing blocking solution. The next day, these sections were then washed and incubated as before with secondary antibodies and Hoechst for 1 hr at RT, followed by 3 ×PBS washes and mounting as described above.

### Confocal imaging and analysis

Confocal images were acquired using a LSM900 confocal microscope analysis and were analyzed using FIJI/ImageJ [61] or Imaris (Version 9.6.1, Oxford Instruments). Unless otherwise stated, all representative images are orthogonal projections of acquired Z-stacks. During all confocal imaging and subsequent analyses, blinding was performed to mask both the animal’s genotype and ID.

### Pj cell and interneuron labeling

Immunolabelling for TdTomato, PVALB, and CALB in brain sections were imaged as Z-stacks using a 40x objective. To assess the number of PVALB-positive cells also positive for TdTomato, a cellular mask was first created using the PVALB channel as reference in FIJI/ImageJ. Cells also positive for TdT were then identified by manual thresholding and counted as an independent sub-group. Counts were performed exclusively in *Pvalb-TdT* mice, *n* = 3 images per area, per N.

To quantify numbers of Purkinje cells (PVALB and CALB positive) and PVALB-positive interneurons (PVALB-positive only) in the cerebellar cortex, PVALB-positive cells were first identified using the “Cells” function of Imaris. Cerebellar Purkinje cells (PJ) were then identified as PVALB-positive cells also immunoreactive for CALB (determined by manual thresholding), and PVALB interneurons counted as PVALB-positive, CALB negative cells in the molecular layer. To analyze changes in interneuron and PJ cell numbers between groups, counts were performed on multiple images of matched sagittal planes of control (*Atg5*^*f/f*^) and *Pvalb-atg5* animals (*N* = 3 animals per genotype; *n* = 9 and *n* = 8 images, respectively).

### Active CASP3 labelling

As above, active CASP3 labeling was imaged as Z-stacks using a 40x objective. First, PVALB-interneurons and PJ cells were identified using the “Cells” function of Imaris. Cells were then identified as active CASP3 positive by manual thresholding of active CASP3 immunoreactivity and identified as a subset of the total interneuron and PJ cell numbers. To identify potential differences between groups, counts were performed on multiple images of matched sagittal planes of control (*Atg5*^*f/f*^) and *Pvalb-atg5* animals. *n* = 1 image analyzed per mouse (N), *N* = 7 per genotype.

#### TUNEL cell death assay

Immediately after embedding, TUNEL-labeled sections were imaged as tile scans of a single Z-plane using a 20× objective to cover both the cortex and hippocampal formation. To quantify Fluorescin signals exclusively in forebrain PVALB cells, the “Cells” function of Imaris was used to identify and mask TdT-positive PVALB neurons. The mean signal intensity (sum of pixel intensities/number of pixels within mask) of Fluorescin was then recorded for each cell. In experimental (*Pvalb-TdT* or *Pvalb-atg5-TdT*) and positive control samples, Fluorescin-positive cells were determined as any cell with a mean fluorescein intensity above the highest recorded value found in negative control sections from the same experimental replicate. *n* = 2–3 tile scans were analyzed per mouse (N), *N* = 4 per genotype.

#### Quantification of SAR labeling

The hippocampal CA1 region of SAR labeled sections were imaged as z-stacks using a 63× objective. To quantify levels of each SAR in PVALB cells and their processes, masks were generated using the “Surfaces” function of Imaris, which identified PVALB cells using either TdT fluorescence or PVALB co-labeling. The mean signal intensities of RTN3, CALCOCO1, BNIP3, or BNIP3L were then recorded within the masked areas (as above). *n* = 7–12 images analyzed per mouse (N), N detailed in each legend.

For PRKN-labeled sections, PRKN puncta were identified using the “Spots” function of Imaris. To assess changes in puncta density between control (*Pvalb-Cre*) and *Pvalb-atg5* cells, counts of PRKN-positive puncta were normalized by dividing the number of puncta by the PVALB mask volume (recorded in µm^3^). To also examine potential differences in puncta intensity between groups, the mean PRKN intensity was calculated as the sum of pixel intensities within identified puncta, divided by the total number of pixels in those puncta. *n* = 10–14 images analyzed per mouse (N), *N* = 4 per genotype.

#### Surface and total GRIA1/GluA1, GRIA2/GluA2 and GRIN2A/NR2A labeling

The hippocampal CA1 region of GRIA1/GluA1, GRIA2/GluA2 or GRIN2A/NR2A-labeled sections were imaged as z-stacks using a 63× objective and 3× digital zoom. To quantify total and surface levels of each receptor in PVALB cells and their processes, masks were generated using the “Surfaces” function of Imaris using PVALB co-labeling. The mean signal intensity (sum of pixel intensities/number of pixels within mask) of GRIA1/GluA1, GRIA2/GluA2, or GRIN2A/NR2A were then recorded within the masked areas (as above). *n* = 9–16 images (cells) analyzed per N. N detailed in each legend.

### Tissue dissociation and FACS

Brains from control *(Pvalb-TdT)*, mutant *Pvalb-atg5-TdT*, and TdTomato-negative *(Atg5*^*f/f*^) mice (serving as a negative control) were isolated in ice-cold PBS. Individual cells from dissected isocortices and hippocampi were then isolated using the Papain Dissociation System (Worthington Biochemical Corp, LK003150), according to the manufacturer’s instructions. Myelin elimination was then performed using a Percoll™ [Sigma-Aldrich, P1644] gradient. First, 3 ml of PBS and 1 ml of Percoll™ solution (90% Percoll™ in PBS, pH 7.4) were added to dissociated cells and mixed well. Then, 4 ml of PBS was layered over the Percoll-cell mixture to create two phases. Samples were then centrifuged at 3000 ×g for 10 min at 4°C, with an acceleration ramp at 1 and braking ramp at 0. The resulting supernatant and myelin were then discarded, and the cell pellet resuspended in 1 ml FACS medium (PBS containing 2% FBS and 2 mM EDTA). Fluorescence Activated Cell Sorting (FACS) was performed using a MoFlo AstriosEQ High Speed Cell Sorter (Beckman Coulter).

### Clarity and 3D image acquisition

Animals were transcardially perfused with 4% PFA and brains dissected and were postfixed overnight in 4% PFA. Tissues were then clarified using the CLARITY protocol [62], and X-CLARITY^TM^ system (Logos Biosystems). Brains were immersed in a refractive index matching solution/RIMS containing Histodenz (Sigma Aldrich, D2158) for at least 24 h before being imaged. Imaging was performed using a mesoSPIM system [63] at Wyss Center, Geneva. The microscope consists of a dual-sided excitation path using a fiber-coupled multiline laser combiner (405, 488, 561 and 647 nm, Toptica MLE) and a detection path comprising a 42 Olympus MVX-10 zoom macroscope with a 1× objective (Olympus MVPLAPO 1×), a filter wheel (Ludl 96A350), and a scientific CMOS (sCMOS) camera (Hamamatsu Orca Flash 4.0 V3). The excitation paths also contain galvo scanners for light-sheet generation and reduction of shadow artifacts due to absorption of the light-sheet. In addition, the beam waist is scanned using electrically tunable lenses (ETL, Optotune EL-16–40-5D-TC-L) synchronized with the rolling shutter of the sCMOS camera. This axially scanned light-sheet mode (ASLM) leads to a uniform axial resolution across the field-of-view (FOV) of 5 μm. Image acquisition is done using custom software written in Python. Z-stacks were acquired with a zoom set at 1.25X at 5 μm spacing, resulting in an in-plane pixel size of 5.26 X 5.26 μm (2048 ×2048 pixels). Excitation wavelength of the td-Tomato and autofluorescence channels were set at 561 and 488 nm, respectively with an emission filter, 593/40 LP and 530/43 nm bandpass filter respectively (BrightLine HC, AHF). Images were stitched and reconstructed in 3D using Arivis Vision 4D software (Zeiss).

### Registration to the Allen reference Atlas, mask extraction and segmentation

#### Brain registration to Allen brain Atlas

Mouse brains were un-anisotropically down-sampled and registered to the 25 µm Allen brain mouse atlas [64] using the AMAP pipeline [65] through Brainreg frontend [66] using the autofluorescence channel. The affine registration was computed using 5 steps and the registration was computed for each affine step. The freeform registration was computed using 5 steps and the registration was computed on the last 3 steps only. The bending weight energy was set to 0.9 which was a good trade-off for accommodating sample imperfections while maintaining high regularization to avoid overfitting the data. All other parameters were kept as default.

#### Mask creation for sub-region segmentation

To accelerate the segmentation in Arivis Vision 4D software, we restricted the volume on which the segmentation was performed by using a binary mask of the volume of interest. To create the binary masks, we used a custom developed tool which interfaces with the Allen Brain Atlas API and automatically generates any region as a binary mask. This tool allows us to create a mask using Allen Brain Atlas region names or acronyms. It automatically and efficiently looks for children’s regions to create masks and allows the user to batch create masks when the input is a list of regions. The output binary masks were produced at 25 µm isotropic resolution for memory footprint efficiency and resampled later in Arivis Vision 4D software at the sample resolution for the segmentation step.

#### Segmentation

The masked region was imported into Arivis Vision 4D software and used for the creation of 3D masked region for cell counting analysis. TdT-positive cells (PVALB-cells) and neuronal fibers were classified using a random forest machine learning-based algorithm (ML). Due to the diversity of the brain areas and heterogeneity of the background of whole brains scans, the ML algorithm was trained for each brain area and each sample separately and blindly. We attributed the heterogeneity of the background to the different light scattering properties of each analyzed volume. Based on the ML training the 3D objects were segmented and filtered by features, such as volume (voxel count) and object sphericity using Arivis Vision 4D software. The raw counted cells were normalized by masked region volume.

### Correlative light and electron microscopy (CLEM) analysis

#### Tissue preparation and imaging

*Pvalb-TdT* and *Pvalb-atg5-TdT* mice were euthanized with an overdose of isofluorane inhalation and immediately perfused, via the heart, with a buffered mix of 1.25% glutaraldehyde [Roth, 3778.1] and 2.0% paraformaldehyde in 0.1 M phosphate buffer (pH 7.4). Brains were then removed after 2 hours, and coronal vibratome sections cut at a thickness of 80 µm. At this point, confocal images were taken of TdT-positive PVALB cells of interest, taking care to collect high- and low-resolution images so that the exact region could be imaged in the electron microscope. The imaged sections were then post-fixed in potassium ferrocyanide (1.5%; Merck, 1,548,269) and osmium tetraoxide (2%; Roth, 8088.1), followed by thiocarbohydrazide (1%; Sigma-Aldrich, 223,220) and then osmium tetraoxide (2%). They were then stained overnight in uranyl acetate (1%; Electron Microscopy Sciences, 22,400/1), washed in distilled water at 50°C, before being stained with lead aspartate solution (Sigma-Aldrich, 228,621) at the same temperature. They were finally dehydrated in increasing concentrations of alcohol and embedded in Spurs resin (Sigma-Aldrich, EM0300-1KT) and hardened at 65°C for 24 h between glass slides. The regions containing the cells previously imaged with confocal light microscopy were trimmed from the rest of the section using a razor blade, glued to an aluminum stub, and then further trimmed using a glass knife mounted inside a scanning electron microscope (Zeiss Merlin, Zeiss NTS). Serial sections were cut from the face using an ultramicrotome mounted in the microscope (Gatan, 3View) and the block face imaged after every cut using a beam voltage of 1.7 kV and pixel size of 7 nm with a dwell time of 1 microsecond. Series of sections were cut to include the entire volume containing the cells of interest. Entire cell volumes, comprising of serial Z-planes, were then reconstructed in FIJI/ImageJ using the TrakEM2 plugin [67]. Measurements of ER volume, mitochondrial volume and the percentage of damaged mitochondria were recorded using the “Stereology” plugin of IMOD (Version 4.11) [68]. ER diameter measurements were performed in FIJI/ImageJ [61] using line and “Measurement” functions.

### Proteomic analysis

#### Protein digestion

Replicate samples were digested according to a modified version of the iST method (named miST method) [69]. Briefly, frozen cell pellets were resuspended in 30 µl miST lysis buffer (1% sodium deoxycholate, 100 mM Tris [Roth, AE15.1] pH 8.6, 10 mM DTT [Roth, 6908.3]) by vortexing. Resuspended samples were heated at 95°C for 5 min. Samples were then diluted 1:1 (v:v) with water containing 4 mM MgCl_2_ (Sigma-Aldrich, M8266) and benzonase (Merck, 70,746; 100x dil. of stock = 250 Units/μl) and incubated for 15 min at RT to digest nucleic acids. Reduced disulfides were alkylated by adding ¼ vol of 160 mM chloroacetamide (final 32 mM; Sigma-Aldrich, C0267) and incubating at 25°C for 45 min in the dark. Samples were adjusted to 3 mM EDTA and digested with 0.2 μg trypsin/LysC mix (Promega, V5073) for 1 h at 37°C, followed by a second 1 h digestion with a second and identical aliquot of proteases. To remove sodium deoxycholate, two sample volumes of isopropanol (Sigma-Aldrich, 1.02781) containing 1% TFA (Sigma-Aldrich, T6508) were added to the digests, and the samples were desalted on a strong cation exchange (SCX) plate (Oasis MCX; Waters Corp., Milford, MA) by centrifugation. After washing with isopropanol, 1%TFA, peptides were eluted in 200 μL of 80% MeCN (Supelco, 1.00029), 19% water, 1% (v:v) ammonia (Sigma-Aldrich, 499,145).

#### Liquid chromatography-tandem mass spectrometry

Eluates after SCX desalting were dried and resuspended in variable volumes of 0.05% trifluoroacetic acid, 2% acetonitrile (ThermoFisher Scientific, 047138.K2) to equilibrate concentrations. 1 μg of each sample was injected onto columns for nanoLC-MS analysis.

#### MS analysis

Data-dependent LC-MS/MS analyses of samples were carried out on a Fusion Tribrid Orbitrap mass spectrometer (Thermo Fisher Scientific) interfaced through a nano-electrospray ion source to an Ultimate 3000 RSLCnano HPLC system (Dionex, USA). Peptides were separated on a reversed-phase custom packed 40 cm C18 column (75 μm ID, 100Å, Reprosil Pur 1.9 μm particles (Dr. Maisch, r119.b9) with a 4–90% acetonitrile gradient in 0.1% formic acid (total time 140 min). Full MS survey scans were performed at 120,000 resolution. A data-dependent acquisition method controlled by Xcalibur 4.2 software (Thermo Fisher Scientific) was used that optimized the number of precursors selected (“top speed”) of charge 2+ to 5+ while maintaining a fixed scan cycle of 0.6 s. HCD fragmentation mode was used at a normalized collision energy of 32%, with a precursor isolation window of 1.6 m/z, and MS/MS spectra were acquired in the ion trap. Peptides selected for MS/MS were excluded from further fragmentation during 60 s.

#### MS data analysis

Tandem MS data were processed by the MaxQuant software (version 1.6.14) [70] incorporating the Andromeda search engine [71]. The UniProt *Mus musculus* reference proteome (RefProt) database of 26 August 2020 was used (55,508 sequences), supplemented with sequences of common contaminants. Trypsin (cleavage at K,R) was used as the enzyme definition, allowing 2 missed cleavages. Carbamidomethylation of cysteine was specified as a fixed modification. N-terminal acetylation of protein and oxidation of methionine were specified as variable modifications. All identifications were filtered at 1% FDR at both the peptide and protein levels with default MaxQuant parameters. MaxQuant data were further processed with Perseus software [72]. iBAQ [73] values were used for quality control assessment, after which LFQ values [74] were used for quantitation after log2 transformation. Only protein groups were retained which had all four valid values in at least one condition. In the resulting table, missing values were imputed with values determined from a normal distribution (parameters used in Perseus: width 0.3 and down-shift 2.2 SD). All cellular component analyses were performed using the ShinyGO tool version 0.77 [75]. All further analyses were performed using the SynGO and Human Protein Atlas database.

### Jess™ Simple Western analysis

Cell pellets were resuspended in SDS lysis buffer (50 mM Tris, pH 6.8, 2% SDS, 5% glycerol [Roth, 3783.1], 2 mM DTT, 2.5 mM EDTA, 2.5 mM EGTA [Sigma-Aldrich, E4378], 4 mM Na_3_VO_4_ [Sigma-Aldrich, S6508], 20 mM NaF [Sigma-Aldrich, 201,154] complemented with Roche cOmplete™ Mini, EDTA-free Protease Inhibitors and Phos Stop [Roche, 11,836,153,001 and 4,906,845,001, respectively]) and heated 10 min at 65°C. Then, 4.8 μL of protein extracts were subjected to the capillary-based immunoassay Jess™ system (ProteinSimple) using the 12 - 230 kDa separation module (ProteinSimple SM-W004) with the Replex (RP-001) and the Total Protein (DM-TP01) modules according to the manufacturer’s instructions. Analyses were performed using Compass software version 6.1. Peaks were determined using the dropped line method and data were normalized to the total protein.

### In vitro electrophysiology

Mice were anesthetized (ketamine and xylazine; 150 mg and 100 mg/kg, respectively), sacrificed, and their brains were transferred in ice-cold carbogenated (95% O_2_, 5% CO_2_) solution, containing choline chloride 110 mM (Sigma-Aldrich, C7017); glucose 25 mM (ThermoFisher, A16828.0E); NaHCO_3_ 25 mM (Merck, 1,063,290,500), MgCl_2_ 7 mM, ascorbic acid 11.6 mM (Sigma-Aldrich, A90902), sodium pyruvate 3.1 mM (Sigma-Aldrich, P2256); KCl 2.5 mM, NaH_2_PO_4_ 1.25 mM (Merck, S0751) and CaCl_2_ 20.5 mM (Sigma-Aldrich, 1,023,820,250). Coronal brain slices (250 μm thickness) were prepared and transferred for 5 min to warmed solution (34°C) of identical composition, before transfer at room temperature in carbogenated artificial cerebrospinal fluid (ACSF) containing NaCl 124 mM; NaHCO_3_ 26.2 mM; glucose 11 mM; KCl 2.5 mM; CaCl_2_ 2.5 mM; MgCl_2_ 1.3 mM; NaH_2_PO_4_ 1 mM. During recordings, slices were immersed in ACSF and continuously super fused at a flow rate of 2.5 mL min^−1^ at 32°C. Neurons were patch-clamped using borosilicate glass pipettes (2.7–4 MΩ; Phymep, France) under an Olympus-BX51 microscope (Olympus, France). Signal was amplified, filtered at 5 kHz and digitized at 10 kHz (Multiclamp 200B; Molecular Devices, USA). Data were acquired using Igor Pro with NIDAQ tools (Wavemetrics, USA). Access resistance was continuously monitored with a −4 mV step delivered at 0.1 Hz. Extracellular stimulation from AMPI ISO-Flex stimulator was delivered through glass electrodes placed nearby the pyramidal cell patched. Current-clamp recordings were obtained using an internal solution containing: potassium gluconate 140 mM (Sigma-Aldrich, P1847); KCl 5 mM; HEPES 10 mM (Roth, HN77.2); EGTA 0.2 mM; MgCl_2_ 2 mM; Na_2_ATP 4 mM (Sigma-Aldrich, A26209); Na_3_GTP 0.3 mM (Sigma-Aldrich, G8877), creatine phosphate 10 mM (Sigma-Aldrich, 2380). Current-clamp experiments were performed by a series of current steps (negative to positive) injected to induce action potentials (−20, 0, 2,080,160,320,400 pA injection current, 800 ms). Analysis of capacitance and RMP was obtained online from Multiclamp reading. Action potential threshold was computed at the onset of the first action potential produced by current steps, while action potential amplitude was computed on the first evoked action potential and measured from the action potential threshold to peak. For recordings in voltage-clamp configuration, evoked EPSCs were recorded at −60 mV while evoked and spontaneous inhibitory currents (IPSCs) were recorded at +5 mV. Internal solution contained CsMeSO_3_ 120 mM (Sigma-Aldrich, C1426), CsCl 10 mM (Sigma-Aldrich, 289,329), HEPES 10 mM, EGTA 10 mM, creatine phosphate 5 mM, Na_2_ATP 4 mM; Na_3_GTP 0.4 mM, QX-314 5 mM (Hellobio, HB1030). Reversal potential for sodium and chloride was −3 mV and −67 mV, respectively enabling to isolate excitatory and inhibitory components at −60 mV and +5 mV.

### Polysomnographic EEG/EMG recordings

Control (*Atg5*^*f/f*^) and mutant *Pvalb-atg5* littermates were housed in a 12:12 h light/dark cycle (light onset at 09:00, offset at 21:00). EEG and EMG electrodes were implanted as described [76]. Briefly, animals underwent gas anesthesia (1–2% isoflurane with O_2_) and gold-plated electrodes were implanted over *the dura mater* through frontal and parietal bones to acquire frontal and parietal EEG recordings. Two EMG electrodes (gold pellets) were inserted into the neck muscles. A silver wire (Harvard Apparatus) implanted in the bone of cerebellum was used as ground and neutral reference to record referential EEG signals. Electrodes were glued to the skull (cyanoacrylate), soldered to a male-to-male connector, and covered with dental cement (Palavit, Heraeus Kulzer GmbH). Paracetamol (2 mg/ml, Dafalgan) was diluted into the drinking water for 7 days of recovery after the surgery, and an additional week of adaptation to the freely moving cabling system. Animals were monitored according to a score sheet established with the Veterinarian Authorities. Signals were recorded in freely moving conditions with animals in their home cage, amplified, digitized and acquired (RHD2132 amplifier chip, connected to a RHD USB interface board C3100 from Intan Technologies, Los Angeles, CA) at 1000 Hz sampling frequency via Matlab recording software and Intan Matlab toolbox.

### Seizure and epileptic events detection

For the seizure and epileptic event detection, we used the following free online software (https://lifescience.ucd.ie/Epi-AI/), as described [44].

### Behavioral analyses

#### Analysis of behavioral data

All behavioral recordings and analyses were performed in Ethovision XT15 (Noldus Information Technology, Wageningen, The Netherlands), eliminating the potential of experimenter bias and the blinding of animal genotypes/IDs.

#### Open field test (OFT)

Control (*Atg5*^*f/f*^) and mutant *Pvalb-atg5* littermates were habituated in the behavioral room in single cages for at least 1 h before each experiment. Anxiety-like behavior was assessed in a PVC square open field arena (L = 45 cm, W = 45 cm and H = 30 cm) with gray opaque walls, where a central square zone was virtually marked 15 cm from the four walls of the arena. The ambient illumination was 20 l×in the chamber (measurement without infrared light). Infrared light (850 nm) was used during the experiment to improve contrast and tracking detection. Mice were placed for 20 min in the arena and their body-center was tracked with EthoVision XT15. Total distance (cm), velocity (cm/s), number of entries in the center zone, and the percentage of time spent in the center zone were scored.

#### Light/Dark test

Control (*Atg5*^*f/f*^) and mutant *Pvalb-atg5* littermates were tested for their preference for the dark or light compartment of a two-chambered arena, using the Noldus Light/Dark set up. In this set up, the dark compartment (L = 20 cm/W = 20 cm/H = 20 cm) is smaller than the light compartment (L = 40 cm/W = 20 cm/H = 20 cm) with the two being separated by a manually operated sliding door. The walls and ceiling of the dark compartment are permeable to infrared light, allowing optimal mouse tracking. The ambient illumination was 3–4 l×in the dark chamber and 600–700 l×in the light one (measurement without infrared light). Mice were habituated in single cages inside the experimental room for at least 1 h prior to the test. During the test, mice were first placed in the dark compartment with the door closed. After 10 s, the sliding door was opened manually, allowing free transition between compartments for 5 min. The frequency of shuttling between compartments and the time spent in each compartment were scored using EthoVision XT15. The frequency of transition between the areas, the duration, the total distance (cm) and the velocity (cm/s) were measured throughout the test session.

#### Elevated plus maze (EPM)

For an evaluation of anxiety-like behavior, control (*Atg5*^*f/f*^) and mutant *Pvalb-atg5* littermates were subjected to Elevated Plus Maze (EPM). The EPM (Noldus Information Technologies) is a cross-shaped arena elevated from the floor (H: 47 cm) consisting of two open and two closed arms (dimensions of each arm: L = 36 cm, W = 6 cm). The arms are infra-red backlit to optimize the tracking of the mouse in closed arms. The ambient illumination was measured in the closed arm (4–6 lx), in the center (40–60 lx), and in the open arm (104–108 lx). The mice were habituated in a single cage inside the testing room for 1 h before the test. Each mouse was individually placed in the center of the EPM facing one of the closed arms and was allowed to freely explore the maze for 5 min. The number of transitions between the open and closed arms, the percentage time spent inside the zones of the maze, the total distance traveled (cm), and the velocity (cm/s) were then monitored with EthoVision XT15. Anxiety-like behaviors were measured as an increase in closed arms activity (either duration or number entries).

#### Spontaneous activity test

Spontaneous activity of control (*Atg5*^*f/f*^) and mutant *Pvalb-atg5* littermates was measured in the PhenoTyper 3000 (Noldus Information Technologies). The PhenoTyper 3000 is a home-cage for mice (L:30 cm ×W:30 cm) optimized for video tracking via EthoVision XT15. Mice were placed in Phenotypers during the light phase (14:00 - 15:00) with video recording commencing at 19:00 for 72 h. During this period, regular food and water were provided *ad libitum*. A set of spontaneous behavior parameters (as described in [77]) were collected, including food and water consumption, locomotion, sheltering, dark light index, and dark light phase transition.

#### Rotarod test

Control (*Atg5*^*f/f*^) and mutant *Pvalb-atg5* littermates were subjected to the rotarod test to evaluate their balance and motor coordination as previously described [78]. Each mouse was habituated in a single cage inside the experimental room for at least 1 h. The ambient illumination of the experimental room was 25–30 lx. Mice were habituated on the rotarod apparatus for 2 min at constant speed (4 rpm). After 10 min, they were tested on rotarod for 3 trials of 5 min with an inter-trial interval of 10 min. The speed of rotarod was accelerated from 4 rpm to 40 rpm during each 5 min trial and their latency to fall from the rod recorded. The average latency to fall, as well as the latency to fall per trial, were measured in these 5 min trials.

#### Novel object recognition test (NOR)

Control (*Atg5*^*f/f*^) and mutant *Pvalb-atg5* littermates were subjected to a two-day NOR test. On each day, mice were habituated in single cages inside the experimental room, at least 1 h before testing. The first day, mice are individually placed inside an open arena (L = 45 cm, W = 45 cm and H = 30 cm) with gray PVC opaque walls in dim light conditions (20 lx). The arena was filled with bedding (2 cm) in all trials. On the first day, the mouse was habituated inside the empty arena for two trials of 10 min. During the following three trials (inter trial interval = 5 min), two identical objects (Lego bricks) were placed in the arena and the mouse was free to explore the objects for 10 min. At the end of the third object exploration trial, the mouse was brought back to its own home cage in the animal vivarium. 24 h later, mice were individually placed inside the arena for one trial with one familiar object and a novel object. The objects were located at the same distance from each wall (15 cm). Mouse activity was tracked with EthoVision XT15 software. The distance traveled per trial, the time spent exploring each object, the object exploration time per trial, and the discrimination index were measured. Discrimination index was calculated using the following formula: Discrimination Index (%)=[time spent exploring the novel object new – time spent exploring the familiar one)/total time spent exploring all objects]X100%.

#### Fear conditioning test

Control (*Atg5*^*f/f*^) and mutant *Pvalb-atg5* littermates were subjected to a two-day Contextual Fear Conditioning CFC) test. Mice were first habituated in single cages inside the experimental room at least 1 h before testing. Each mouse was placed individually in the fear conditioning chamber (Ugo Basile Srl), which is composed of 4 transparent Plexiglas walls and an electrified metal grid floor. After 2 min of free exploration, two mild foot-shocks (0.5 mA, 2 s) separated by 1 × 1 min interval, were delivered through the grid-floor. Thirty s after the termination of the second foot-shock, the mouse was placed back to its home cage. Memory for the association between the context and the aversive stimulus was probed 24 h later for 5 min inside the same conditioning chamber used during the training session. An overhead camera connected to the software Ethovision XT15 automatically monitors freezing behavior throughout the training and test sessions (i.e. crouched posture and absence of any movement, except for respiratory movements). The percentage of time spent freezing during the test session was used as a proxy measure for the memory of the association formed during the training session. Footshock currents were measured across experiments to ensure that they correspond to 0.5 mA.

### Statistical analysis

Analyses were performed using GraphPad Prism software (Version 10, Dotmatics). All statistical tests are reported in the respective figure legend. Unpaired two-tailed Student’s t-tests were used for comparison between two groups. For comparison of two groups, two-way ANOVA, Multiple-t-test with Tukey or Bonferroni or Holm-Sidak’s multiple comparison correction was used among groups. For datasets violating the assumptions of normality and homogeneity of variances, a Mann-Whitney U-Test (2 samples) or a Kruskal-Wallis H test was used in combination with and a post-hoc Dunn’s multiple comparisons test. For all data analysis, *p* < 0.05 was considered as significant and reported as follows: *p < 0.05, **p ≤ 0.01, ***p ≤ 0.001 and ****p ≤ 0.0001. Results were presented as mean and standard error of mean (±SEM).

### Power analysis

An a priori power analysis was conducted using G*Power version 3.1 [79] to determine the sample size required for *Pvalb-atg5* mice to exhibit any significant alteration in anxiety-like tests (EPM, LD box and OFT). Results showed the minimum sample size to achieve 80% power 1-β = 0.8 (probability of Type II error) to detect a medium effect, at a significance criterion of α = 0.05 (probability of Type I error) when analyzed with a two-tailed Student’s t test (see Table S4).

## Supplementary Material

Chalatsi_Supplement_ACCEPTED_R5.docx

Supplementary_Tables_R5.docx

## Data Availability

Requests for resources and reagents should be addressed to the Lead Contact, Vassiliki Nikoletopoulou (vassiliki.nikoletopoulou@unil.ch). All raw MS data together with MaxQuant output tables are available via the Proteomexchange data repository (www.proteomexchange.org). Project Name: Autophagy is dispensable for the maintenance of PVALB interneurons but required for their inhibitory neurotransmission and memory. Project accession: PXD035264. Username: reviewer_pxd035264@ebi.ac.uk Password: mlWXmW0O
