# Supplementary material for: Autophagy regulates PVALB (parvalbumin) interneuron excitability and memory
Source: Autophagy. 2025 Dec 14;22(2):351–70. doi: 10.1080/15548627.2025.2597463 (PMC12834171; doi:10.1080/15548627.2025.2597463)
Supplement: Chalatsi_Supplement_ACCEPTED_R5.docx [file KAUP_A_2597463_SM2651.docx]

**Supplement**

**Autophagy regulates PVALB (parvalbumin) interneuron excitability and memory**

Theodora Chalatsi^1$^, Erin Wosnitzka^1#^, Angeliki Kolaxi^1#^, Laura M.J. Fernandez^1^, Jules Scholler^2^, Laura Batti^2^, Leonardo Restivo^1^, Graham Knott^3^, Anita Lüthi^1^, Manuel Mameli^1^ and Vassiliki Nikoletopoulou^1*^

^1^Department of Fundamental Neurosciences, Rue du Bugnon 9, CH-1005, Lausanne, Switzerland; ^2^Wyss Center, Chemin des Mines 9, CH-1202, Geneva, Switzerland; 3;

$ present address:

# equal contribution

*Corresponding author: [vassiliki.nikoletopoulou@unil.ch](mailto:vassiliki.nikoletopoulou@unil.ch)

**
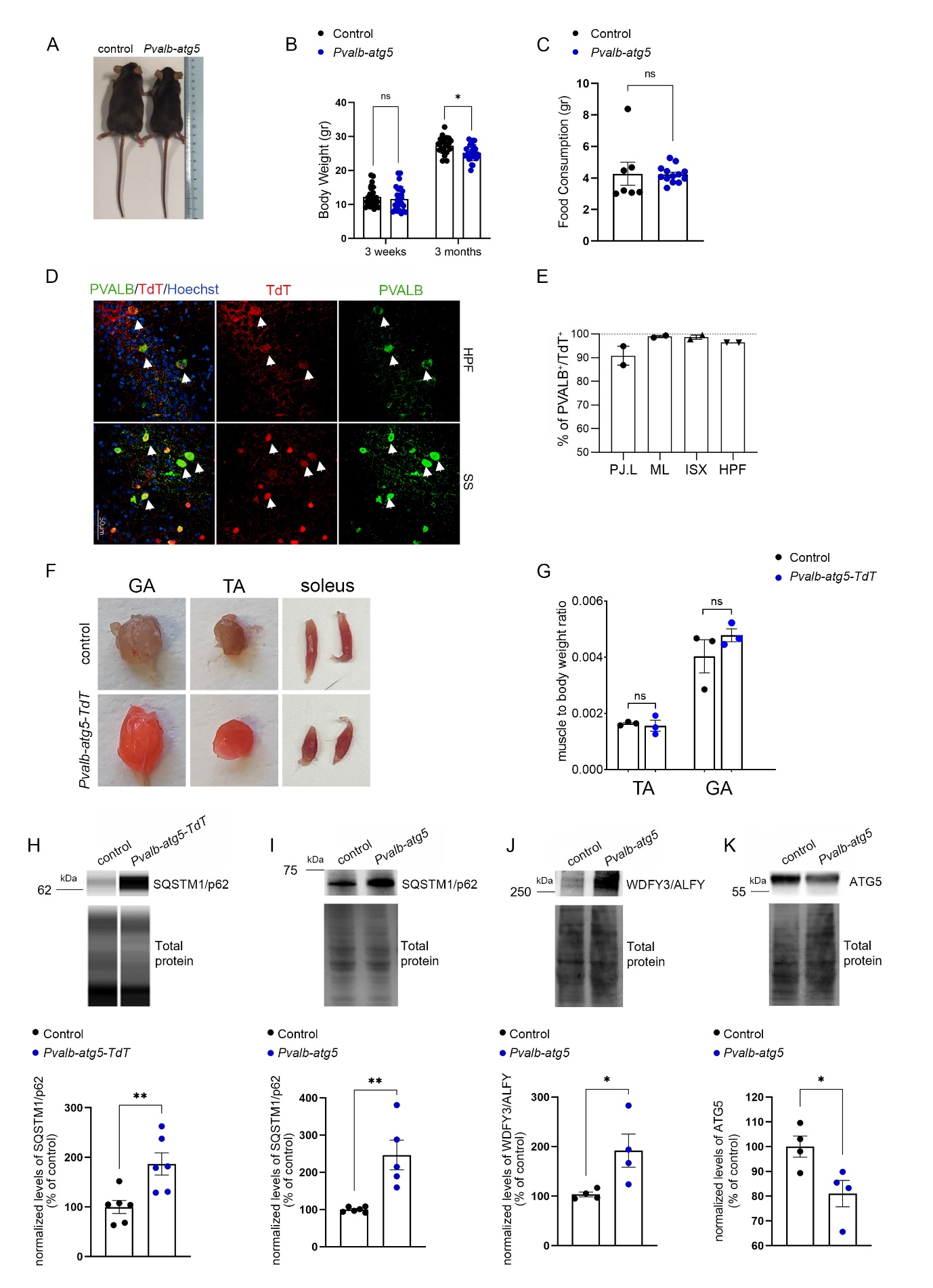
**

**Figure S1**. Validation of autophagy impairment in *Pvalb-atg5* animals. (**A**) Representative images of three-month old control *(Atg5^f/f^)* and *Pvalb-atg5* male littermates. (**B**) Mean bodyweight measurements from three-week and three-month-old control *(Atg5^f/f^)* and *Pvalb-atg5* littermates. Group comparison was performed with a two-way repeated measures ANOVA followed by Sidak post hoc test; F(1,48)=1.987 (^ns^p=0.7207, t=0.7258, df=48 and *p=0.0223, t=2.637, df=48 for 3-weeks- and 3-months-old respectively). Bars represent mean values ±SEM. N=25 animals per genotype. (**C**) Mean food consumption of three-month-old control *(Atg5^f/f^)* and *Pvalb-atg5* littermates recorded during the spontaneous behavior assay. Statistical analysis was performed using unpaired, two-tailed Student’s t test (^ns^p=0.9396, t=0.07686, df=18). Bars represent mean values ±SEM. N=7 and 13 animals for control and *Pvalb-atg5,* respectively. (**D**) Representative confocal images from the hippocampal formation (HPF) and somatosensory cortex (SS) of three-month-old controls *(Pvalb-TdT),* where endogenous TdT is indicated by red and PVALB immunolabelling shown in green. Arrows indicate PVALB cells co-expressing TdT. Scale bar: 50 μm. (**E**) The average percentage of PVALB-immunolabelled cells co-expressing endogenous TdT in the cerebellar Purkinje cell layer (PJ.L) and molecular layer (ML), the isocortex (ISX) and the hippocampal formation (HPF) of control (*Pvalb-TdT)* mice. Bars represent mean values ±SEM. N=2 animals analyzed per genotype. (**F**) Representative images of the glycolytic muscles *gastrocnemius* (GA) and *tibialis anterior* (TA) as well as of the oxidative muscle *soleus* in control (*Atg5^f/f^;TdT*) and *Pvalb-atg5-TdT* animals. Note the expression of TdT in the oxidative muscles in the knockout. (**G**) Graph showing the ratio of muscle to body weight for GA and TA muscles in control and conditional knockout animals. Bars represent mean values ±SEM. N=3 animals analyzed per genotype. Statistical analyses were performed using unpaired, two-tailed t-test (GA: ^ns^p=0.3041, t=1.178, df=4; TA: ^ns^p=0.7163, t=0.3902, df=4). (**H**) Jess Simple Western™ analysis of SQSTM1/p62 levels in FACS sorted cells from three-month-old control *(Pvalb-TdT)* and *Pvalb-atg5-TdT* littermates. Graph indicates levels of SQSTM1/p62 normalized to total protein levels and expressed as a percentage of the mean of the control group. Statistical analysis was performed using unpaired, two-tailed Student’s t test (*p=0.0074, t=3.350, df=10). Bars represent mean values ±SEM. N=6 animals per genotype. (**I-K**) Representative images of western blot analyses for SQSTM1/p62, WDFY3/ALFY and ATG5 in cerebellar lysates of three-month old control (*Atg5^f/f^*) and *Pvalb-atg5* littermates. Graphs indicate levels of SQSTM1/p62, WDFY3/ALFY and ATG5 normalized to total protein and expressed as a percentage of the mean of the control group*.* Statistical analyses were performed using unpaired, two-tailed Student’s t test (for SQSTM/p62: *p=0.0028, t=4.071, df=9; for WDFY3/ALFY: *p=0.0406, t=2.601, df=6; for ATG5: *p=0.0322, t=2.776, df=6). Bars represent mean values ±SEM. N=4 animals analyzed per genotype.


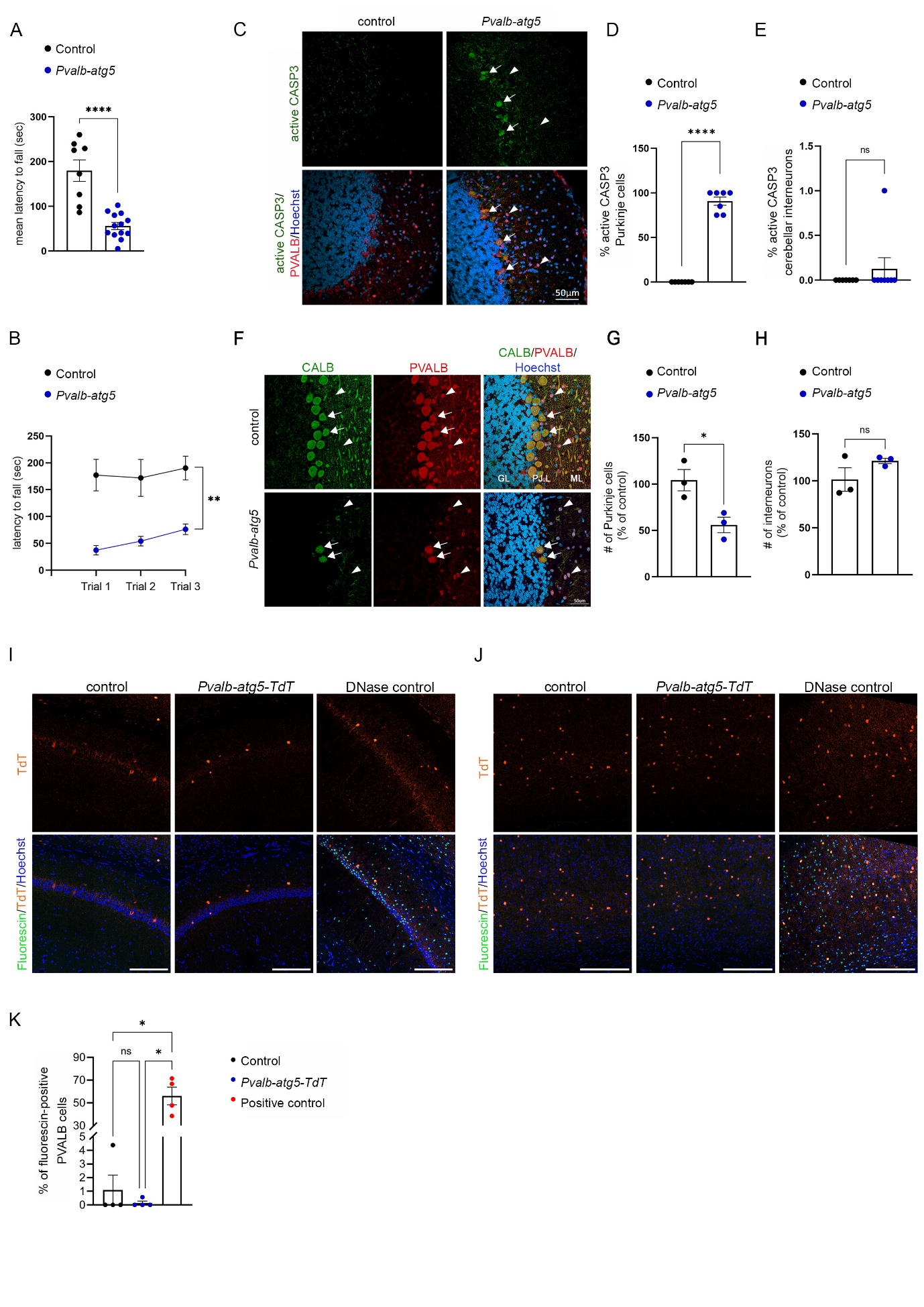


**Figure S2**. Motor and cell survival deficits in *Pvalb-atg5* animals. **(A-B)** Rotarod test was performed on control *(Atg5^f/f^)* and *Pvalb-atg5* littermates. Graphs showing the mean latency to fall (A) and the latency to fall in seconds (B) across trials. Statistical analyses were performed using unpaired, two-tailed Student’s t test (****p<0.0001, t=5.838, df=19) and two-way repeated measures ANOVA followed by Sidak post hoc test; F(2,38)=0.6652 (trial 1 **p=0.0051, trial 2 *p=0.0328 and trial 3 **p=0.0027, respectively). Bars represent mean values ±SEM. N=8 and 13 animals for control and *Pvalb-atg5,* respectively. (**C**) Representative confocal images of active CASP3 in cerebellar sections from three-month-old control *(Atg5^f/f^)* and *Pvalb-atg5* littermates. Immunostaining against active-CASP3 (green) and PVALB (red), as well as with the nuclear dye Hoechst (blue) was performed. (Arrows: Purkinje cells, arrowheads: cerebellar interneurons, GL: Granular layer, PJ: Purkinje cell layer, ML: molecular layer). Scale bar: 50 μm. (**D-E**) The mean relative percentage of cerebellar PJ cells (**C**) and PVALB GABAergic interneurons (**D**) from three-month old control *(Atg5^f/f^)* and *Pvalb-atg5* littermates positive for active CASP3. Statistical analyses were performed using unpaired, two-tailed Student’s t test (****p<0.0001, t=19.92, df=12 and ^ns^p=0.3688, t=0.9309, df=13, for PJ and PVALB interneurons respectively). Bars represent mean values ±SEM. N=7 animals per genotype. (**F**) Representative confocal images of cerebellar sections from six-month-old control (*Atg5^f/f^*) and *Pvalb-atg5* littermates immunolabelled with antibodies against CALB (green), PVALB (red) and the nuclear dye Hoechst (blue). Arrows: Purkinje cells; arrowheads: cerebellar interneurons, GL: granular layer, PJL: Purkinje cell layer, ML: molecular layer. Scale bar: 50 μm. (**G-H**) Relative percentages of PJ cells and PVALB-interneurons in the cerebellar cortices of *Pvalb-atg5* mutants in relation to controls (*Atg5^f/f^*). Statistical analyses were performed using an unpaired, two-tailed Student’s t test (For PJ cells: *p=0.0272, t=3.403, df=4; for interneurons: nsp=0.1989, t=1.538, df=4). Bars represent mean values ± SEM. N=3 animals/genotype. (**I-J**) Representative confocal images from the hippocampus (**I**) and cerebral cortex (**J**) of control (*Pvalb-TdT*) and *Pvalb-atg5-TdT* labelled using a TUNEL assay, showing TdT (orange), Fluorescin (green) and Hoechst (blue). Scale bar: 150 µm. (**K**) Percentage of cells counted as Fluorescin positive in control (*Pvalb-TdT) and Pvalb-atg5-TdT* animals. Statistical analyses were performed using a Kruskal-Wallis (H(2) = 8.425, **p = 0.0061) and a post-hoc Dunn’s multiple comparisons test (Control vs. *Pvalb-atg5-TdT: ^ns^*p=0.999; Control vs. Positive control: *p=0.0417; *Pvalb-atg5-TdT vs.* Positive control: *p=0.0310). N=4 animals per genotype/condition.

*
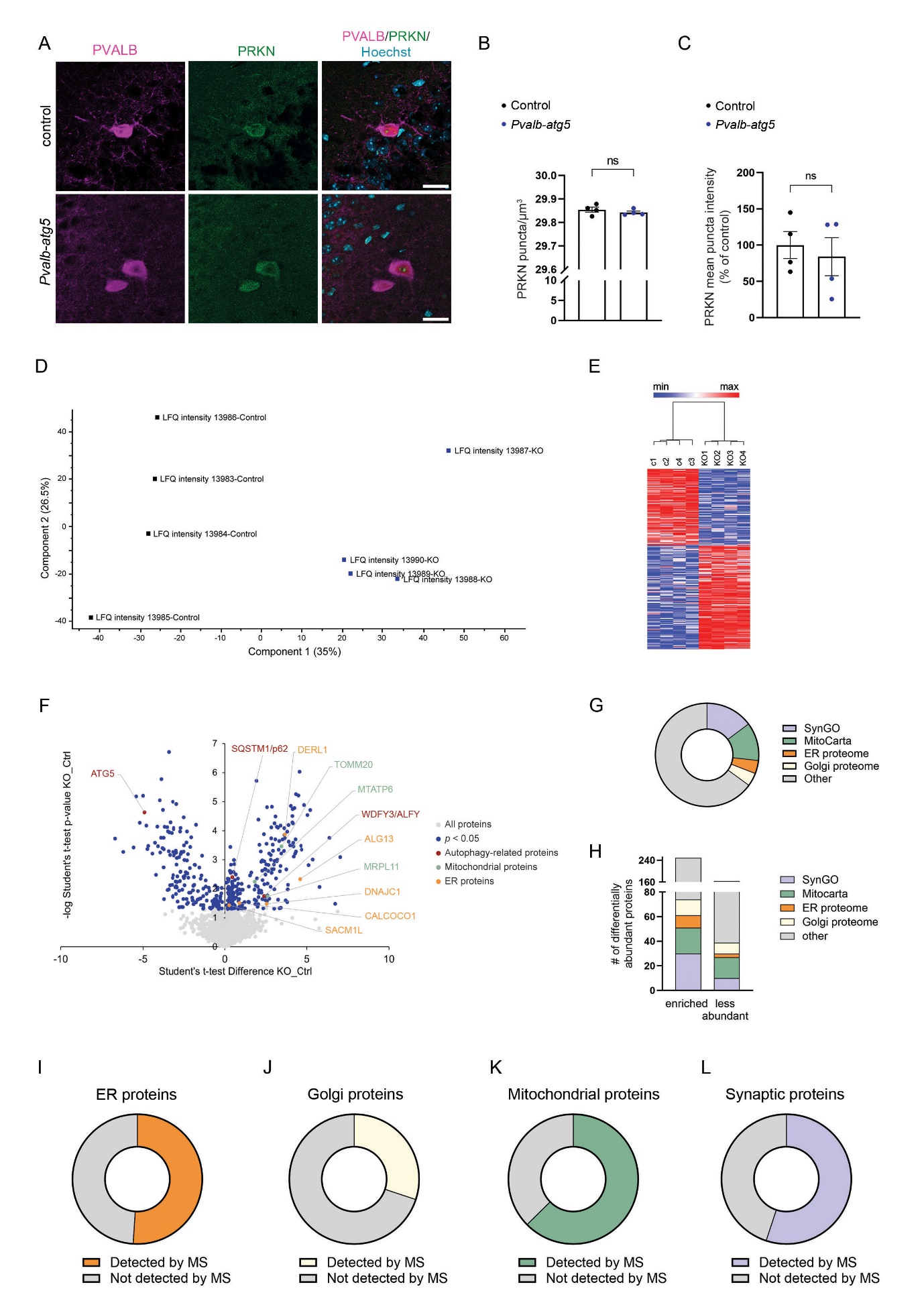
*

**Figure S3**. Mass-spectrometry analysis of control and autophagy-deficient PVALB-expressing interneurons from the cortex and hippocampus. (**A**) Representative single z-plane confocal images of PVALB cells in the CA1 hippocampal area of control (*Pvalb-Cre*) and *Pvalb-atg5* animals, immunolabelled with antibodies against PVALB (magenta), PRKN (green) and stained with the nuclear dye Hoechst (blue). Scale bar: 20 µm. (**B**) Quantification of PRKN puncta count per volume in hippocampal CA1 PVALB cells of control versus *Pvalb-atg5* animals. Statistical analysis was performed using unpaired two-tailed Student’s t test (^ns^p= 0.3833, t=0.9404, df=6). Bars represent mean values ± SEM. N=4 animals per genotype. (**C**) Quantification of the mean PRKN puncta intensity in hippocampal CA1 PVALB cells of control versus *Pvalb-atg5* animals. Results are expressed as a percentage of control values. Statistical analysis was performed using unpaired, two-tailed Student’s t test (^ns^p= 0.6378, t=0.4956, df=6). Bars represent mean values ± SEM. N=4 animals per genotype. (**D**) Principal component analysis of FAC-sorted PVALB cells from control (*Pvalb-TdT*) and mutant *Pvalb-atg5-TdT* brains. N=4 animals per genotype. (**E**) Heatmap indicating the 249 proteins enriched and the 164 proteins decreased in *Pvalb-atg5-TdT* cells compared to control, plus their hierarchical clustering. Significance was set at p<-log10(p-value) ≥1.7;(-log10(0.02)=1.7). (**F**) Volcano plot indicating all 5963 proteins detected by proteomic analysis in FACS sorted control (*Pvalb-TdT*) and *Pvalb-atg5-TdT* interneurons. Analyzed using a Student’s t test, q < 0.05. Grey dots indicate -log10(p-value) < 1.3, remaining colors represent a -log10(p-value) > 1.3; where -log10(0.05)=1.3. Red dots: enriched autophagy-related proteins; green dots: enriched mitochondrial proteins; yellow dots: enriched ER and ERphagy-related proteins. (**G**) Donut chart indicating the proportions of synaptic, mitochondrial, ER and Golgi proteins in our list of identified proteins, as determined by comparison with the SynGO, MitoCarta, The Human Protein Atlas ER and Golgi databases, respectively. (**H**) Bar plot showing enriched versus less abundant synaptic, mitochondrial, ER and Golgi proteins in FACS-sorted *Pvalb-atg5-TdT* interneurons when compared to controls. (**I-L**) The proportion of proteins from each database detected in FAC-sorted PVALB interneurons: 55.06% of synaptic proteins (SynGO), 62.63% of mitochondrial proteins (MitoCarta), 51.04% of ER proteins and 30.09% of Golgi proteins (both The Human Protein Atlas) were observed as MS-identified proteins.


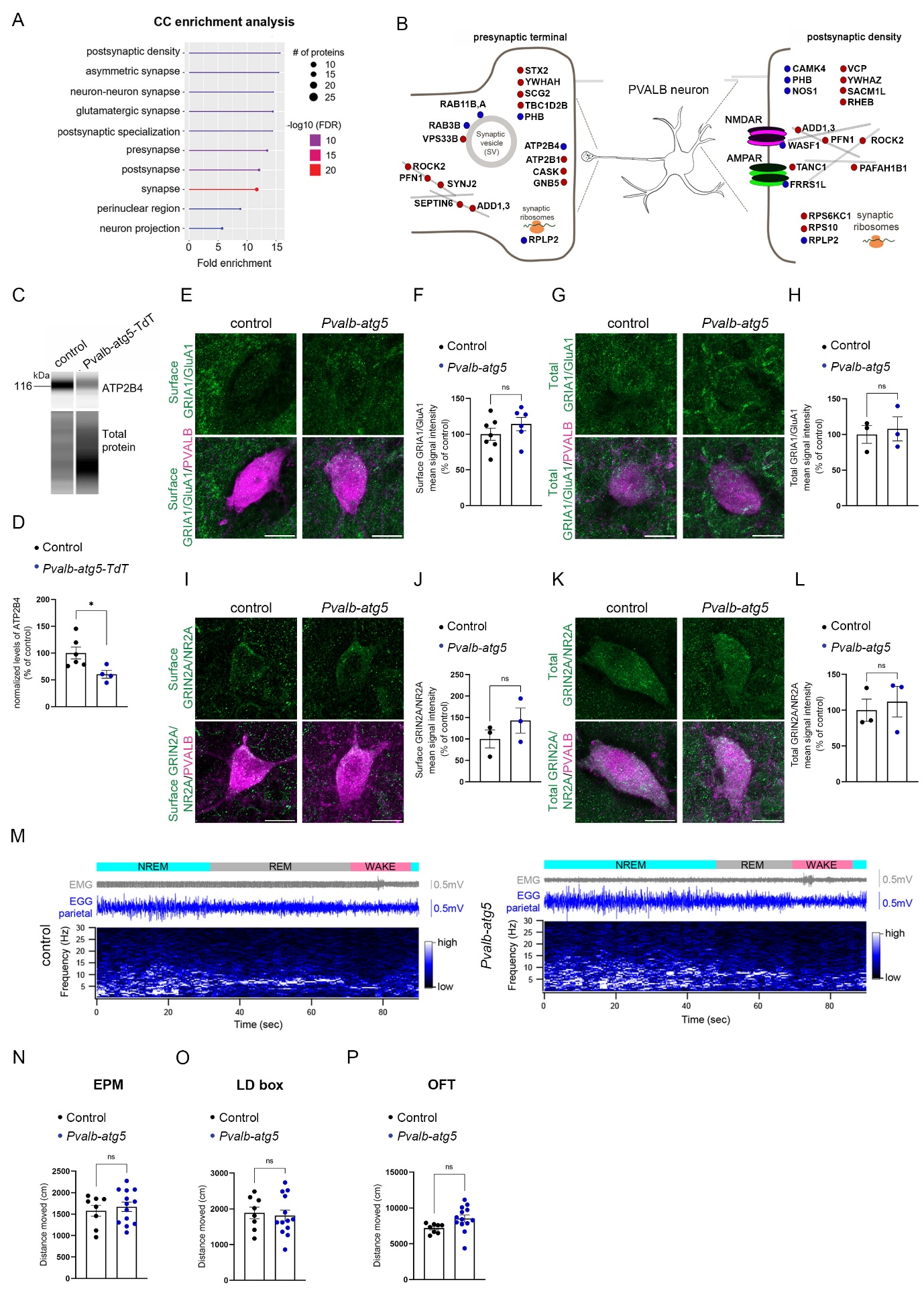


**Figure S4.** Changes in synaptic protein abundance observed in *Pvalb-atg5-TdT* interneurons lead to changes in E/I imbalance but do not result in epileptic seizures. (**A**) ShinyGO cellular component (CC) analysis of the enriched proteins found in *Pvalb-atg5-TdT* FACS sorted interneurons when compared with controls (*Pvalb-TdT*). Size of dots indicates the number of associated genes, whereas the length of the line corresponds to -log10 of the false discovery rate (FDR). (**B**) Graphical representation of the 32 differentially abundant synaptic proteins in the pre- and post-synaptic compartments of PVALB interneurons (red dots: enriched, blue dots: decreased). (**C-D**) Jess Simple Western analysis of ATP2B4 levels in FACS sorted cells from control (*Pvalb~~-~~TdT*) and mutant *Pvalb-atg5-TdT* animals. Graph indicates ATP2B4 protein levels normalized to total protein and expressed as a percentage of the control group mean. Statistical analysis was performed using an unpaired, two-tailed Student’s t test (*p=0.0308, t=2.616, df=8). Bars represent mean values ±SEM. N=6 and 4 animals for control and *Pvalb-atg5-TdT*, respectively. (**E**) Representative images of surface GRIA1/GluA1 (sGluA1) immunoreactivity (green) in CA1 PVALB cells (magenta) from control (*Atg5^f/f^*) vs. *Pvalb-atg5* mice. Scale bar: 10 µm. (**F**) Quantification of the mean surface GRIA1/GluA1 intensity in control versus *Pvalb-atg5* PVALB cells in hippocampal CA1. Results are expressed as a percentage of control values. Statistical analysis was performed using an unpaired, two-tailed Student’s t test (^ns^p=0.2833, t=1.128, df=11). Bars represent mean values ±SEM. N=7 and 6 mice for control and *Pvalb-atg5*, respectively. (**G**) Representative images of total GRIA1/GluA1 immunoreactivity (green) in CA1 PVALB cells (magenta) from control (*Atg5^f/f^*) vs. *Pvalb-atg5* mice. Scale bar: 10 µm. (**H**) Quantification of the mean total GRIA1/GluA1 intensity in control versus *Pvalb-atg5* PVALB cells in hippocampal CA1. Results are expressed as a percentage of control values. Statistical analysis was performed using an unpaired, two-tailed Student’s t test (^ns^p=0.7304, t=0.3697, df=4). Bars represent mean values ±SEM. N=3 mice per genotype. (**I**) Representative images of surface GRIN2A/NR2A immunoreactivity (green) in CA1 PVALB cells (magenta) from control (*Atg5^f/f^*) vs. *Pvalb-atg5* mice. Scale bar: 10 µm. (**J**) Quantification of the mean surface GRIN2A/NR2A intensity in control versus *Pvalb-atg5* PVALB cells in hippocampal CA1. Results are expressed as a percentage of control values. Statistical analysis was performed using an unpaired, two-tailed Student’s t test (^ns^p=0.1035, t=2.101, df=4). Bars represent mean values ±SEM. N=3 mice per genotype. (**K**) Representative images of total GRIN2A/NR2A immunoreactivity (green) in CA1 PVALB cells (magenta) from control (*Atg5^f/f^*) vs. *Pvalb-atg5* mice. Scale bar: 10 µm. (**L**) Quantification of the mean total GRIN2A/NR2A intensity in control versus *Pvalb-atg5* PVALB cells in hippocampal CA1. Results are expressed as a percentage of control values. Statistical analysis was performed using an unpaired, two-tailed Student’s t test (^ns^p=0.6736, t=0.4536, df=4). Bars represent mean values ±SEM. N=3 mice per genotype. (**M**) Representative 90-sec traces and corresponding time-frequency plots as heatmaps from EEG/EMG recordings from control (*Atg5^f/f^*) and *Pvalb-atg5* mice. NREM: Non-rapid eye movement sleep; REM: rapid eye movement sleep. (**N**) The mean distance moved (cm) by control (*Atg5^f/f^*) and *Pvalb-atg5* littermates during the EPM test. Statistical analyses were performed using a two-tailed Student’s t test (^ns^p=0.5968, t=0.5380, df=19). Bars represent mean values ±SEM. N=8 and 13 animals for control and *Pvalb-atg5*, respectively. (**O**) The mean distance moved (cm) by control (*Atg5^f/f^*) and *Pvalb-atg5* littermates during the LD box test. Statistical analyses were performed using a two-tailed Student’s t test (^ns^p=0.7562, t=0.3150, df=19). Bars represent mean values ±SEM. N=8 and 13 animals for control and *Pvalb-atg5*, respectively. (**P**) The mean distance moved (cm) by control (*Atg5^f/f^*) and *Pvalb-atg5* littermates during the OFT test. Statistical analyses were performed using a two-tailed Student’s t test (^ns^p=0.0531, t=2.062, df=19). Bars represent mean values ±SEM. N=8 and 13 animals for control and *Pvalb-atg5*, respectively.
