# Supplementary material for: Autophagy regulates PVALB (parvalbumin) interneuron excitability and memory
Source: Autophagy. 2025 Dec 14;22(2):351–70. doi: 10.1080/15548627.2025.2597463 (PMC12834171; doi:10.1080/15548627.2025.2597463)
Supplement: Supplementary_Tables_R5.docx [file KAUP_A_2597463_SM2650.docx]

Table S1. TdT-positive cell counts and brain volumes of *Pvalb-TdT* and *Pvalb-atg5-TdT* animals.

| **control *Pvalb-TdT* (N = 4)** | | | | | ***Pvalb-atg5-TdT* (N = 3)** | | | |
| --- | --- | --- | --- | --- | --- | --- | --- | --- |
| **ROI Full name** | **Mean (# of cells)** | **SD** | **Mean (volume in mm^3^)** | **SD** | **Mean (# of cells)** | **SD** | **Mean (volume in mm^3^)** | **SD** |
| CB Cerebellum | 128450 | 39818 | 128450 | 39818 | 40295 | 28574 | 40295 | 28574 |
| Isocortex Isocortex | 509304 | 123591 | 509304 | 123591 | 581596 | 76326 | 581596 | 76326 |
| HPF Hippocampal formation | 40689 | 12458 | 40689 | 12458 | 44478 | 19960 | 44478 | 19960 |
| STR Striatum | 31980 | 8781 | 31980 | 8781 | 33188 | 4742 | 33188 | 4742 |
| MSC+LS Medium and lateral septum | 3876 | 733.9 | 3876 | 733.9 | 3668 | 944.2 | 3668 | 944.2 |
| BLA Basolateral amygdalar nucleus | 2504 | 508 | 2504 | 508 | 2947 | 428.4 | 2947 | 428.4 |
| LA Lateral amygdala | 632 | 251.1 | 632 | 251.1 | 525.3 | 196.3 | 525.3 | 196.3 |
| TH Thalamus | 34416 | 12442 | 34416 | 12442 | 29416 | 2689 | 29416 | 2689 |
| HY Hypothalamus | 10631 | 2707 | 10631 | 2707 | 10328 | 4014 | 10328 | 4014 |

# Table S2. List of proteins detected in mass-spec analysis with < 2 peptides.

| LFQ | LFQ | LFQ | LFQ | LFQ | LFQ | LFQ | LFQ | Student's T-test Signif icant KO_Ctrl | Student's | GOBP | GOCC | KEGG | Reactom | Peptides | Razor + | Unique | Sequence | Unique + | Unique | Mol. | Q-v alue | Score | Intensity | **MS/MS** | Razor + | Razor + | Razor + | Razor + | Razor + | Razor + | Razor + | Razor + | -Log Student's T-test p- | Student's T-test q-v alue | Student's T-test | Student's | Protein IDs | Majority | **Protein names** | Gene | Fasta |
| --- | --- | --- | --- | --- | --- | --- | --- | --- | --- | --- | --- | --- | --- | --- | --- | --- | --- | --- | --- | --- | --- | --- | --- | --- | --- | --- | --- | --- | --- | --- | --- | --- | --- | --- | --- | --- | --- | --- | --- | --- | --- |
| intensity 13983-ctrl | intensity 13984-ctrl | intensity 13985-ctrl | intensity 13986-ctrl | intensity 13987-KO | intensity 13988-KO | intensity 13989-KO | intensity 13990-KO |  | T-test signif ican t | slim name | slim name | name | e |  | unique peptides | peptides | cov erage [%] | razor sequence cov erage | sequence cov erage [%] | weight [kDa] |  |  |  | **count** | unique peptides 13983-ctrl | unique peptides 13984-ctrl | unique peptides 13985-ctrl | unique peptides 13986-ctrl | unique peptides 13987-KO | unique peptides 13988-KO | unique peptides 13989-KO | unique peptides 13990-KO | v alue KO_Ctrl | KO_Ctrl | Dif f erence KO_Ctrl | T-test Test statistic KO_Ctrl |  | protein IDs |  | names | headers |
|  |  |  |  |  |  |  |  |  |  |  |  |  |  |  |  |  |  | [%] |  |  |  |  |  |  |  |  |  |  |  |  |  |  |  |  |  |  |  |  |  |  |  |

23.77062 23.47457 24.00949 23.55039 20.42706 20.22727 20.23676 20.23835 + KO_Ctrl biological cell part;intracellular membrane- 1 1 1 3.7 3.7 3.7 34.943 0 4.0677 25.73771 1 1 1 1 1 0 0 0 0 6.705578967 0 -3.418909073 -26.35396 F6V6B6;Q8VBU F6V6B6;Q Protein BANP Banp tr|F6V6B6|F6V6B6_MOUSE Protein BANP (Fragment) OS=Mus musculus OX=10090 GN=Banp PE=1 SV=2;sp|Q8VBU8|BANP_MOUSE Protein BANP OS=Mus musculus OX=10090 GN=Banp PE=1 SV=1

19.24876 18.87222 19.43807 19.85525 23.656 24.03591 23.82852 24.02935 + KO_Ctrl biological cell part;c ErbB sign REACT_2 2 2 2 8.4 8.4 8.4 59.312 0 5.4187 26.38306 5 0 0 0 0 2 1 2 2 6.027577032 0 4.533864975 20.263918 Q8CE90 Q8CE90 Dual specif icity m Map2k7 sp|Q8CE90|MP2K7_MOUSE Dual specif icity mitogen-activ ated protein kinase kinase 7 OS=Mus musculus OX=10090 GN=Map2k7 PE=1 SV=1

23.61762 23.97614 23.84316 23.74622 19.47362 19.87977 20.43262 19.88661 + KO_Ctrl cell part;cy toplasm;intracellular 2 2 2 7.3 7.3 7.3 35.274 0.004644 1.774 25.7985 2 1 1 1 1 0 0 1 0 5.777751583 0 -3.877631187 -18.3865 Q9D0D4 Q9D0D4 Probable dimethy l Dimt1 sp|Q9D0D4|DIM1_MOUSE Probable dimethy ladenosine transf erase OS=Mus musculus OX=10090 GN=Dimt1 PE=2 SV=1

20.45553 20.22159 20.32584 20.57549 22.41596 22.43604 22.11092 22.30694 + KO_Ctrl biological cell part;cy toplasm;cy toskeleto 3 3 3 7 7 7 94.316 0 4.3306 24.89601 9 2 1 0 0 3 2 2 2 5.716823681 0 1.9228549 17.954914 Q5SSG4;F2Z4A Q5SSG4 GAS2-like protein Gas2l2 sp|Q5SSG4|GA2L2_MOUSE GAS2-like protein 2 OS=Mus musculus OX=10090 GN=Gas2l2 PE=1 SV=1

30.49247 30.46168 32.38193 30.76793 20.1866 20.58055 20.79382 21.83113 + KO_Ctrl 4 4 4 5.2 5.2 5.2 136.23 0.008305 1.468 33.39777 3 2 2 2 1 1 2 1 0 5.674096748 0 -10.17798042 -17.65812 Q3U3C9 Q3U3C9 Genetic suppresso Gse1 sp|Q3U3C9|GSE1_MOUSE Genetic suppressor element 1 OS=Mus musculus OX=10090 GN=Gse1 PE=1 SV=2

19.43297 20.14212 19.44708 20.59836 24.34699 24.20284 24.24743 24.10981 + KO_Ctrl biological cell part;intracellular REACT_2 4 4 4 18.4 18.4 18.4 42.554 0 10.609 26.70372 8 0 2 0 0 3 3 3 2 5.25403581 0 4.321633816 14.981043 P19426;G3UY 39 P19426;G Negativ e elongatio Nelf e sp|P19426|NELFE_MOUSE Negativ e elongation f actor E OS=Mus musculus OX=10090 GN=Nelf e PE=1 SV=2;tr|G3UY 39|G3UY 39_MOUSE Negativ e elongation f actor E (Fragment) OS=Mus musculus OX=10090 GN=Nelf e PE=1 SV=1

20.2305 21.60173 20.94029 20.54033 25.09889 25.3772 25.38215 25.32776 + KO_Ctrl cell part;extracellular organelle; 7 7 7 29 29 29 46.201 0 15.131 28.34075 6 4 4 3 3 2 3 4 2 5.210242084 0 4.468287468 14.725493 Q8K2Q7;A0A0A6 Q8K2Q7 BRO1 domain-con Brox sp|Q8K2Q7|BROX_MOUSE BRO1 domain-containing protein BROX OS=Mus musculus OX=10090 GN=Brox PE=1 SV=1

25.79 25.57906 25.1563 24.85548 20.36623 20.44236 19.64469 20.94786 + KO_Ctrl anatomica cell part;c Adherens junction;Ba 4 3 3 14.7 12.3 12.3 61.508 0 6.8994 27.5451 4 2 1 3 2 1 0 0 1 5.200115315 0 -4.994924545 -14.667 Q8R5H6 Q8R5H6 Wiskott-Aldrich sy Wasf 1 sp|Q8R5H6|WASF1_MOUSE Wiskott-Aldrich sy ndrome protein f amily member 1 OS=Mus musculus OX=10090 GN=Wasf 1 PE=1 SV=2

25.44269 25.41044 25.6725 25.83615 21.24265 19.80773 19.71467 19.94933 + KO_Ctrl 4 4 4 61.9 61.9 61.9 11.546 0 10.094 27.52425 6 3 4 3 4 0 0 0 0 5.180714305 0 -5.411850929 -14.55554 Q9CRC6 Q9CRC6 UPF0693 protein C10orf 32 h sp|Q9CRC6|BORC7_MOUSE BLOC-1-related complex subunit 7 OS=Mus musculus OX=10090 GN=Borcs7 PE=1 SV=1

23.76213 24.34489 24.53249 23.70725 20.04198 20.67945 19.73948 19.98771 + KO_Ctrl biological cell part;cy toplasmic REACT_3 4 4 4 8.1 8.1 8.1 82.79 0 15.184 26.37171 5 1 2 3 1 0 1 1 0 5.04478679 0 -3.974534988 -13.79695 Q80WG7;E9Q3A Q80WG7; E3 ubiquitin-protein Trim36 sp|Q80WG7|TRI36_MOUSE E3 ubiquitin-protein ligase Trim36 OS=Mus musculus OX=10090 GN=Trim36 PE=1 SV=2;tr|E9Q3A0|E9Q3A0_MOUSE E3 ubiquitin-protein ligase Trim36 OS=Mus musculus OX=10090 GN=Trim36 PE=1 SV=1;tr|A0A494B9M7|A0A494B9M7_MOUSE E3 ubiquitin-protein

24.07145 23.78756 23.78616 23.48431 20.55931 20.70005 21.07911 20.10386 + KO_Ctrl anatomica cell part;c Histidine metabolism 2 2 2 11.9 11.9 11.9 33.664 0 6.985 26.90185 3 1 1 1 1 0 1 1 2 4.995452395 0 -3.171787262 -13.53101 Q91VF2 Q91VF2 Histamine N-meth Hnmt sp|Q91VF2|HNMT_MOUSE Histamine N-methy ltransf erase OS=Mus musculus OX=10090 GN=Hnmt PE=1 SV=1

23.1791 23.64339 23.58795 23.71461 21.12309 20.53921 20.43202 20.24417 + KO_Ctrl carbohy dr cell part;m Gly cosphi REACT_3 4 4 4 23.9 23.9 23.9 35.414 0 5.6812 26.20631 6 3 2 1 2 1 1 2 1 4.918779923 0 -2.946641922 -13.12728 Q9WUV2;A0A0H Q9WUV2; Alpha-N-acety lgala St6galnac sp|Q9WUV2|SIA7C_MOUSE Alpha-N-acety lgalactosaminide alpha-2,6-sialy ltransf erase 3 OS=Mus musculus OX=10090 GN=St6galnac3 PE=1 SV=2;tr|A0A0H2UKC4|A0A0H2UKC4_MOUSE Alpha-N-acety lgalactosaminide alpha-2,6-sialy ltransf erase 3 (Fragment) OS=Mus musculus OX=1009

21.46049 21.50612 19.96153 20.80542 25.91775 25.50127 25.75901 25.91239 + KO_Ctrl biological cell part;intracellular REACT_2 4 4 4 8.2 8.2 8.2 92.632 0 23.604 28.26548 3 1 2 1 0 2 1 1 1 4.880280558 0 4.839215755 12.928868 Q8CIG3;F6V3V2 Q8CIG3;F Ly sine-specif ic his Kdm1b sp|Q8CIG3|KDM1B_MOUSE Ly sine-specif ic histone demethy lase 1B OS=Mus musculus OX=10090 GN=Kdm1b PE=1 SV=1;tr|F6V3V2|F6V3V2_MOUSE Ly sine-specif ic histone demethy lase 1B (Fragment) OS=Mus musculus OX=10090 GN=Kdm1b PE=1 SV=1

20.16019 20.3142 20.96852 19.76841 23.87173 24.78616 24.23579 24.36833 + KO_Ctrl anatomica cell part;cy toplasm;i REACT_2 5 5 5 14.3 14.3 14.3 63.053 0 7.3383 27.15991 7 1 1 1 1 3 4 2 2 4.85765267 0 4.012675762 12.813569 A0A286Y DB8;Q A0A286Y Elongator complex Elp3 tr|A0A286Y DB8|A0A286Y DB8_MOUSE Elongator complex protein 3 OS=Mus musculus OX=10090 GN=Elp3 PE=1 SV=1;sp|Q9CZX0|ELP3_MOUSE Elongator complex protein 3 OS=Mus musculus OX=10090 GN=Elp3 PE=1 SV=1

20.88966 21.50397 20.50443 20.9035 24.05167 23.67691 23.83798 23.65578 + KO_Ctrl biosy nthe cell part;intracellular membrane- 2 2 2 3.3 3.3 3.3 96.382 0 3.973 26.44444 3 0 0 0 0 1 2 1 2 4.82421093 0 2.855196953 12.644934 V9GWX3;Q80TE V9GWX3; RNA poly merase I Rpap1 tr|V9GWX3|V9GWX3_MOUSE RNA poly merase II-associated protein 1 OS=Mus musculus OX=10090 GN=Rpap1 PE=1 SV=1;sp|Q80TE0|RPAP1_MOUSE RNA poly merase II-associated protein 1 OS=Mus musculus OX=10090 GN=Rpap1 PE=1 SV=2

20.71244 21.19106 20.2771 20.68534 23.32882 23.10074 23.19191 23.46538 + KO_Ctrl biological cell part;membrane 3 3 3 7.9 7.9 7.9 67.487 0 5.8282 25.77992 3 0 0 0 0 1 1 3 1 4.809823812 0 2.555227757 12.573026 A0A2R8W6Y 5;A A0A2R8W La-related protein Larp4 tr|A0A2R8W6Y 5|A0A2R8W6Y 5_MOUSE La-related protein 4 OS=Mus musculus OX=10090 GN=Larp4 PE=1 SV=1;tr|A0A2R8VKL5|A0A2R8VKL5_MOUSE La-related protein 4 OS=Mus musculus OX=10090 GN=Larp4 PE=1 SV=1;tr|E9Q066|E9Q066_MOUSE La-related protein 4 OS=Mus musculus OX=1

20.34271 20.18703 20.70218 19.75355 25.30281 24.111 24.18382 24.31679 + KO_Ctrl biological cell part;e TP53 signal REACT_2 2 2 2 5.9 5.9 5.9 54.747 0.004235 1.875 26.68672 2 0 0 0 0 2 2 1 2 4.781149192 0 4.232235909 12.430849 A0A0R4J1G9;E9 A0A0R4J1 Metalloreductase SSteap3 tr|A0A0R4J1G9|A0A0R4J1G9_MOUSE Metalloreductase STEAP3 OS=Mus musculus OX=10090 GN=Steap3 PE=1 SV=1;tr|E9QN92|E9QN92_MOUSE Metalloreductase STEAP3 OS=Mus musculus OX=10090 GN=Steap3 PE=1 SV=1;sp|Q8CI59|STEA3_MOUSE Metalloreductase STEAP3 OS=Mus musculus OX

20.34237 20.74874 19.34951 20.53098 24.49546 24.3319 24.01095 24.61807 + KO_Ctrl cell part;endoplasmic reticulum; 2 2 2 12.2 12.2 12.2 58.507 0 4.0991 26.90736 2 0 1 0 0 1 1 1 1 4.747717865 0.002444444 4.121191978 12.266984 Q99J23 Q99J23 GH3 domain-conta Ghdc sp|Q99J23|GHDC_MOUSE GH3 domain-containing protein OS=Mus musculus OX=10090 GN=Ghdc PE=2 SV=2

23.50142 23.81861 23.92823 23.94878 20.79448 19.39289 19.7955 20.06021 + KO_Ctrl cell part;cell surf ace;extracellul 2 2 2 7 7 7 50.555 0 3.7937 25.75929 2 1 2 1 1 0 0 0 0 4.717008911 0.002315789 -3.788486481 -12.11824 Q80V42 Q80V42 Carboxy peptidase Cpm sp|Q80V42|CBPM_MOUSE Carboxy peptidase M OS=Mus musculus OX=10090 GN=Cpm PE=1 SV=2

21.65785 20.45514 19.90482 19.76704 25.70749 25.69365 25.6876 25.5616 + KO_Ctrl biological cell part;cy toplasm;endosome;i 3 3 3 4.2 4.2 4.2 115.77 0 6.5397 28.25156 7 1 1 1 0 1 2 2 2 4.709776163 0.0022 5.216374397 12.083454 E9QMX4;Q8BLK E9QMX4;QRibosomal protein Rps6kc1 tr|E9QMX4|E9QMX4_MOUSE Non-specif ic serine/threonine protein kinase OS=Mus musculus OX=10090 GN=Rps6kc1 PE=1 SV=1;sp|Q8BLK9|KS6C1_MOUSE Ribosomal protein S6 kinase delta-1 OS=Mus musculus OX=10090 GN=Rps6kc1 PE=1 SV=2

26.02378 24.99261 25.02378 25.55256 20.19153 21.08622 19.66109 20.9935 + KO_Ctrl anatomica cell part;c Regulation REACT_2 4 4 4 32.4 32.4 32.4 32.402 0 13.874 27.42037 13 4 4 3 3 0 0 0 0 4.636409863 0.002095238 -4.91509676 -11.7358 Q99J83 Q99J83 Autophagy protein Atg5 sp|Q99J83|ATG5_MOUSE Autophagy protein 5 OS=Mus musculus OX=10090 GN=Atg5 PE=1 SV=1

20.24517 20.10065 20.8374 20.73649 26.22597 25.82607 25.70844 24.34469 + KO_Ctrl biological cell part;c mRNA su REACT_2 1 1 1 3.2 3.2 3.2 75.769 0.004501 1.7871 28.11515 3 0 0 0 0 1 1 1 1 4.537294151 0.002 5.046363831 11.280827 Q8K3W3 Q8K3W3 Protein CASC3 Casc3 sp|Q8K3W3|CASC3_MOUSE Protein CASC3 OS=Mus musculus OX=10090 GN=Casc3 PE=1 SV=3

20.39159 19.809 20.63519 19.52756 24.60947 23.796 24.6903 23.67336 + KO_Ctrl biological extracellular organelle;extracellu 11 3 3 35.2 10.5 10.5 46.879 0 7.5086 26.70359 5 0 0 0 0 2 3 3 3 4.501830503 0.001913043 4.101448536 11.122033 P07759 P07759 Serine protease in Serpina3k sp|P07759|SPA3K_MOUSE Serine protease inhibitor A3K OS=Mus musculus OX=10090 GN=Serpina3k PE=1 SV=2

20.27791 19.93435 20.49174 21.17364 23.60128 23.72775 23.28107 23.56871 + KO_Ctrl metabolic cell part;cy toplasm;extracellular 4 4 4 22.5 22.5 22.5 24.443 0 3.7943 26.19126 7 0 1 0 1 2 3 2 2 4.48999472 0.001833333 3.075294018 11.069496 Q9D142;A0A217 Q9D142;A Uridine diphosphat Nudt14 sp|Q9D142|NUD14_MOUSE Uridine diphosphate glucose py rophosphatase NUDT14 OS=Mus musculus OX=10090 GN=Nudt14 PE=1 SV=1;tr|A0A217FL49|A0A217FL49_MOUSE Uridine diphosphate glucose py rophosphatase NUDT14 OS=Mus musculus OX=10090 GN=Nudt14 PE=1 SV=1

22.45949 22.88082 22.89567 22.36469 20.12964 19.69584 20.19645 20.57897 + KO_Ctrl biological cell part;intracellular membrane- 2 2 2 2 2 2 246.7 0.000164 3.0005 24.74945 3 2 1 1 1 0 0 0 1 4.465493874 0.00176 -2.499941826 -10.96146 E9Q507;K4DI71; E9Q507;K GON-4-like protein Gon4l tr|E9Q507|E9Q507_MOUSE GON-4-like protein OS=Mus musculus OX=10090 GN=Gon4l PE=1 SV=1;tr|K4DI71|K4DI71_MOUSE GON-4-like protein OS=Mus musculus OX=10090 GN=Gon4l PE=1 SV=1;sp|Q9DB00|GON4L_MOUSE GON-4-like protein OS=Mus musculus OX=10090 GN=Gon4l PE=1 SV=3

20.77943 21.20188 20.33858 20.58886 22.78257 22.93885 22.69534 22.65417 + KO_Ctrl anatomica cell part;c Calcium s REACT_3 1 1 1 3.2 3.2 3.2 52.377 0.00693 1.5966 25.26116 2 0 0 0 0 1 1 1 1 4.378619607 0.003384615 2.040545464 10.58614 F7AAE0;Q8K2J0 F7AAE0;QPhosphoinositide p Plcd3 tr|F7AAE0|F7AAE0_MOUSE Phosphoinositide phospholipase C (Fragment) OS=Mus musculus OX=10090 GN=Plcd3 PE=1 SV=1;sp|Q8K2J0|PLCD3_MOUSE 1-phosphatidy linositol 4,5-bisphosphate phosphodiesterase delta-3 OS=Mus musculus OX=10090 GN=Plcd3 PE=1 SV=2

23.60738 23.7495 24.98007 24.30744 20.55938 20.97726 20.92064 20.88424 + KO_Ctrl biological cell part;c Arrhy thmo REACT_3 3 3 3 4.5 4.5 4.5 112.68 0 8.5893 26.34099 5 2 2 3 2 0 0 0 0 4.287824665 0.008148148 -3.325715065 -10.20643 Q9QUM0 Q9QUM0 Integrin alpha-IIb;I Itga2b sp|Q9QUM0|ITA2B_MOUSE Integrin alpha-IIb OS=Mus musculus OX=10090 GN=Itga2b PE=1 SV=2

19.30159 19.76437 20.6989 19.83308 24.30459 24.33429 24.18359 23.17072 + KO_Ctrl anatomica cell body ;cell part;cell projection 9 9 9 8.3 8.3 8.3 200.06 0 4.271 27.17874 12 4 2 0 0 2 4 2 3 4.281544937 0.007857143 4.098815441 10.180627 E9QAF9;Q0VGY E9QAF9;QProtein TANC1 Tanc1 tr|E9QAF9|E9QAF9_MOUSE Protein TANC1 OS=Mus musculus OX=10090 GN=Tanc1 PE=1 SV=1;sp|Q0VGY 8|TANC1_MOUSE Protein TANC1 OS=Mus musculus OX=10090 GN=Tanc1 PE=1 SV=2

25.50446 25.87734 26.39632 25.50624 21.5926 20.44213 19.42048 21.05728 + KO_Ctrl biosy nthe cell part;m Gly ceroph REACT_2 1 1 1 4.2 4.2 4.2 55.603 0.002322 2.1657 27.97842 1 1 1 1 1 0 0 0 0 4.274179055 0.007586207 -5.192968369 -10.15044 Q99LH2 Q99LH2 Phosphatidy lserine Ptdss1 sp|Q99LH2|PTSS1_MOUSE Phosphatidy lserine sy nthase 1 OS=Mus musculus OX=10090 GN=Ptdss1 PE=1 SV=1

20.34203 19.5003 20.45849 19.43405 23.05051 22.5906 22.98557 23.19187 + KO_Ctrl anatomica cell part;c Allograf t r REACT_3 4 1 0 14.1 4.3 0 37.41 0.005068 1.7206 25.38479 1 0 0 0 0 1 1 1 1 4.256018593 0.007333333 3.020916939 10.07636 P14430;E9PWT4 P14430;E H-2 class I histoco H2-Q8;H2 sp|P14430|HA18_MOUSE H-2 class I histocompatibility antigen, Q8 alpha chain OS=Mus musculus OX=10090 GN=H2-Q8 PE=3 SV=1;tr|E9PWT4|E9PWT4_MOUSE H-2 class I histocompatibility antigen, Q7 alpha chain OS=Mus musculus OX=10090 GN=H2-Q7 PE=3 SV=2;tr|E9QJR9|E9QJ

19.40817 21.02242 20.10306 20.22212 25.0471 24.11791 24.11593 24.00941 + KO_Ctrl response cell part Arachidon REACT_3 3 3 3 23.9 23.9 23.9 24.148 0.000635 2.5206 26.76395 5 1 0 0 0 2 1 2 1 4.255478022 0.007096774 4.133644104 10.074162 Q9D7B7 Q9D7B7 Probable glutathion Gpx8 sp|Q9D7B7|GPX8_MOUSE Probable glutathione peroxidase 8 OS=Mus musculus OX=10090 GN=Gpx8 PE=1 SV=1

22.61178 22.7911 22.48734 22.09381 20.84937 20.47259 20.74079 20.91656 + KO_Ctrl biological cell part;c Bladder ca REACT_3 2 1 1 6.7 3.5 3.5 56.135 0.004229 1.8681 24.52307 1 1 1 1 1 0 0 0 0 4.207678356 0.0085 -1.751177788 -9.881541 E9JGN0;Q8VDF E9JGN0;QDeath-associated Dapk2 tr|E9JGN0|E9JGN0_MOUSE DAP-kinase-related protein 1 beta isof orm OS=Mus musculus OX=10090 GN=Dapk2 PE=1 SV=1;sp|Q8VDF3|DAPK2_MOUSE Death-associated protein kinase 2 OS=Mus musculus OX=10090 GN=Dapk2 PE=1 SV=1

19.79345 19.96064 21.37589 20.1433 24.4025 23.7543 24.15599 24.43841 + KO_Ctrl anatomica cell part;centrosome; REACT_2 5 5 5 9.1 9.1 9.1 110.09 0 12.749 26.79501 6 1 1 0 1 1 4 3 2 4.200793478 0.008242424 3.869479179 9.8540709 Q80SY 4;F6ZBL2 Q80SY 4;F E3 ubiquitin-protein Mib1 sp|Q80SY 4|MIB1_MOUSE E3 ubiquitin-protein ligase MIB1 OS=Mus musculus OX=10090 GN=Mib1 PE=1 SV=1;tr|F6ZBL2|F6ZBL2_MOUSE RING-ty pe E3 ubiquitin transf erase (Fragment) OS=Mus musculus OX=10090 GN=Mib1 PE=1 SV=1

18.67668 20.20793 19.38173 19.23711 22.73136 23.05623 22.37984 22.87455 + KO_Ctrl biological cell part;macromolecular compl 3 3 3 13.8 13.8 13.8 42.354 0.006643 1.606 25.97695 3 2 1 1 0 1 1 1 1 4.174025527 0.008 3.384632111 9.7479214 Q8BUY 9;A0A49 Q8BUY 9; Gerany lgerany l tra Pggt1b sp|Q8BUY 9|PGTB1_MOUSE Gerany lgerany l transf erase ty pe-1 subunit beta OS=Mus musculus OX=10090 GN=Pggt1b PE=1 SV=1;tr|A0A494BA07|A0A494BA07_MOUSE Gerany lgerany l transf erase ty pe-1 subunit beta OS=Mus musculus OX=10090 GN=Pggt1b PE=1 SV=1

25.02454 24.5257 24.92873 24.86429 18.99915 20.34118 19.5888 21.22073 + KO_Ctrl biological regulation;cellular process;respo 2 2 2 6.1 6.1 6.1 116.24 0 10.43 26.83714 4 2 2 1 2 0 0 0 0 4.171759688 0.007771429 -4.79834938 -9.738983 Q99MR1;A0A0G Q99MR1; PERQ amino acid- Gigy f 1 sp|Q99MR1|GGY F1_MOUSE GRB10-interacting GYF protein 1 OS=Mus musculus OX=10090 GN=Gigy f 1 PE=1 SV=2;tr|A0A0G2JGR7|A0A0G2JGR7_MOUSE GRB10-interacting GYF protein 1 (Fragment) OS=Mus musculus OX=10090 GN=Gigy f 1 PE=1 SV=1

24.8259 24.92606 24.86835 25.05109 21.0721 21.69309 19.6595 20.72869 + KO_Ctrl cellular co cell part;intracellular membrane- 7 7 7 18.8 18.8 18.8 68.171 0 47.942 26.7497 15 4 6 3 4 0 1 0 0 4.144504496 0.007555556 -4.129500866 -9.632044 Q8C0J2;D3YZW Q8C0J2;D Autophagy -related Atg16l1 sp|Q8C0J2|A16L1_MOUSE Autophagy -related protein 16-1 OS=Mus musculus OX=10090 GN=Atg16l1 PE=1 SV=1;tr|D3YZW7|D3Y ZW7_MOUSE Autophagy -related protein 16-1 (Fragment) OS=Mus musculus OX=10090 GN=Atg16l1 PE=1 SV=1

20.40773 20.97622 20.18011 19.79838 23.38337 24.14585 23.1965 23.2832 + KO_Ctrl anatomica cell part;cell projectio REACT_2 4 4 4 2.4 2.4 2.4 289.07 0.001866 2.2273 26.29957 4 0 0 0 0 2 2 1 2 4.139544691 0.007351351 3.16161871 9.612697 Q6A078;E9Q9M0 Q6A078;E Centrosomal prote Cep290 sp|Q6A078|CE290_MOUSE Centrosomal protein of 290 kDa OS=Mus musculus OX=10090 GN=Cep290 PE=1 SV=2;tr|E9Q9M0|E9Q9M0_MOUSE Centrosomal protein of 290 kDa OS=Mus musculus OX=10090 GN=Cep290 PE=1 SV=1

24.13548 23.576 24.8597 23.71177 20.07889 20.99465 20.59611 19.87459 + KO_Ctrl cell death; cell part;macromolecular compl 2 2 2 13.7 13.7 13.7 20.116 0.006213 1.6382 26.48932 5 2 2 2 1 0 0 1 1 4.134255687 0.007157895 -3.684676647 -9.592104 Q9CZX9 Q9CZX9 ER membrane pro Emc4 sp|Q9CZX9|EMC4_MOUSE ER membrane protein complex subunit 4 OS=Mus musculus OX=10090 GN=Emc4 PE=1 SV=1

23.42714 23.85386 23.76091 23.92533 21.15326 19.73049 20.37249 19.61962 + KO_Ctrl anatomica cell part;c Basal tran REACT_2 2 2 2 9.7 9.7 9.7 35.848 0.001262 2.4553 25.66457 2 1 1 2 2 0 0 0 1 4.118135789 0.006974359 -3.522845268 -9.529584 P51949;A0A087 P51949;A CDK-activ ating kin Mnat1 sp|P51949|MAT1_MOUSE CDK-activ ating kinase assembly f actor MAT1 OS=Mus musculus OX=10090 GN=Mnat1 PE=1 SV=2;tr|A0A087WSQ7|A0A087WSQ7_MOUSE CDK-activ ating kinase assembly f actor MAT1 OS=Mus musculus OX=10090 GN=Mnat1 PE=1 SV=1;tr|A0A087WSG5|A0A087WSG5_MOUSE

20.29174 19.34304 20.14296 19.84513 22.76967 22.32405 22.52597 23.29936 + KO_Ctrl alcohol m cell part REACT_2 2 2 2 4 4 4 79.582 0.007464 1.5214 25.08582 2 0 0 0 0 1 1 2 1 4.113594102 0.0068 2.824043751 9.5120346 Q9JKZ2 Q9JKZ2 Sodium/my o-inosit Slc5a3 sp|Q9JKZ2|SC5A3_MOUSE Sodium/my o-inositol cotransporter OS=Mus musculus OX=10090 GN=Slc5a3 PE=1 SV=2

20.36119 19.94426 20.97083 20.29634 24.28926 23.01995 23.45438 23.72265 + KO_Ctrl biological cell part;c Inositol ph REACT_2 2 2 2 5.8 5.8 5.8 54.158 0.009867 1.4163 27.42037 2 1 1 1 1 1 1 1 1 4.105402113 0.006634146 3.228402615 9.4804537 Q8C5L6;Q5ND4 Q8C5L6;Q Inositol poly phosp Inpp5k sp|Q8C5L6|INP5K_MOUSE Inositol poly phosphate 5-phosphatase K OS=Mus musculus OX=10090 GN=Inpp5k PE=1 SV=2;tr|Q5ND44|Q5ND44_MOUSE Inositol poly phosphate 5-phosphatase K (Fragment) OS=Mus musculus OX=10090 GN=Inpp5k PE=1 SV=1

23.54498 23.29572 23.53112 24.04469 21.0698 20.84446 19.82096 20.16593 + KO_Ctrl alcohol m cell part;c Gly coly si REACT_3 2 1 1 5.5 3.5 3.5 58.82 0.000163 2.9653 25.54984 2 1 1 1 1 0 0 0 0 4.101113111 0.00647619 -3.128841877 -9.463957 E9Q509;G3X925 E9Q509;G Py ruv ate kinase;P Pklr tr|E9Q509|E9Q509_MOUSE Py ruv ate kinase OS=Mus musculus OX=10090 GN=Pklr PE=1 SV=1;tr|G3X925|G3X925_MOUSE Py ruv ate kinase OS=Mus musculus OX=10090 GN=Pklr PE=1 SV=1;sp|P53657|KPY R_MOUSE Py ruv ate kinase PKLR OS=Mus musculus OX=10090 GN=Pklr PE=1 SV=1;tr|D3Z2

23.4353 23.51376 22.79269 23.79501 21.11765 20.90062 20.42377 20.32163 + KO_Ctrl behav ior;b cell part;c ABC trans REACT_3 1 1 1 1.1 1.1 1.1 237.75 0.000164 3.0021 25.28132 1 1 1 1 1 0 0 0 0 4.101083546 0.006325581 -2.693272114 -9.463843 E9Q6G4;Q91V24 E9Q6G4;QATP-binding casse Abca7 tr|E9Q6G4|E9Q6G4_MOUSE ATP-binding cassette sub-f amily A member 7 OS=Mus musculus OX=10090 GN=Abca7 PE=1 SV=1;sp|Q91V24|ABCA7_MOUSE ATP-binding cassette sub-f amily A member 7 OS=Mus musculus OX=10090 GN=Abca7 PE=1 SV=1

21.05871 19.37294 20.47064 20.27798 23.69424 24.18162 23.58187 24.1795 + KO_Ctrl cellular metabolic process;cellular process 5 5 5 11.8 11.8 11.8 63.858 0 6.3921 27.01829 9 1 1 1 1 1 2 4 3 4.090546522 0.006181818 3.614237785 9.4234223 A0A2I3BR81;Q9 A0A2I3BR81;Q91X76;A0A2I3 Nt5dc2 tr|A0A2I3BR81|A0A2I3BR81_MOUSE 5-nucleotidase domain-containing 2 OS=Mus musculus OX=10090 GN=Nt5dc2 PE=1 SV=1;tr|Q91X76|Q91X76_MOUSE 5-nucleotidase domain-containing 2 OS=Mus musculus OX=10090 GN=Nt5dc2 PE=1 SV=1;tr|A0A2I3BQR1|A0A2I3BQR1_MOUSE 5-nucleo

24.02648 23.90784 23.60784 23.89717 20.71085 21.66061 20.3113 20.25468 + KO_Ctrl biological cell part;intracellular membrane- 3 3 3 9 9 9 58.867 0 4.5184 25.9162 2 1 1 1 1 0 1 1 0 4.05161128 0.006044444 -3.125471115 -9.275392 Q91W36;E9Q8W Q91W36; Ubiquitin carboxy l- Usp3 sp|Q91W36|UBP3_MOUSE Ubiquitin carboxy l-terminal hy drolase 3 OS=Mus musculus OX=10090 GN=Usp3 PE=2 SV=1;tr|E9Q8W9|E9Q8W9_MOUSE Ubiquitin carboxy l-terminal hy drolase OS=Mus musculus OX=10090 GN=Usp3 PE=1 SV=1;tr|G3UZF0|G3UZF0_MOUSE Ubiquitin carboxy l-termin

23.31617 23.55485 23.56023 23.52552 20.56948 20.44231 18.89794 20.12268 + KO_Ctrl anatomica cell part;c MAPK sig REACT_3 6 3 3 14.2 6.4 6.4 55.541 0.00202 2.2179 26.12524 3 1 1 2 1 0 1 1 1 3.98136648 0.010173913 -3.481090546 -9.013527 Q9JI11;Q8CDG4 Q9JI11 Serine/threonine-pr Stk4 sp|Q9JI11|STK4_MOUSE Serine/threonine-protein kinase 4 OS=Mus musculus OX=10090 GN=Stk4 PE=1 SV=1

23.7683 23.21128 23.59685 23.54851 21.7713 21.28005 20.66037 21.21014 + KO_Ctrl biological cell part;cy toplasm;intracellular 3 3 3 35.7 35.7 35.7 22.656 0 7.4909 26.60428 5 1 1 2 1 0 1 1 0 3.978745537 0.009957447 -2.300767899 -9.003883 O88843 O88843 Death domain-con Cradd sp|O88843|CRADD_MOUSE Death domain-containing protein CRADD OS=Mus musculus OX=10090 GN=Cradd PE=1 SV=2

23.53825 24.02217 24.1888 22.90514 21.02552 20.77086 21.17044 20.81687 + KO_Ctrl cellular m cell part;cy toplasm;intracellular 3 3 3 6.6 6.6 6.6 74.331 0 4.5618 27.11734 4 1 2 2 2 1 1 1 1 3.971832558 0.00975 -2.717667103 -8.978492 Q8VEG4;A0A1W Q8VEG4; Exonuclease 3-5 d Exd2 sp|Q8VEG4|EXD2_MOUSE Exonuclease 3-5 domain-containing protein 2 OS=Mus musculus OX=10090 GN=Exd2 PE=1 SV=2;tr|A0A1W2P7Q1|A0A1W2P7Q1_MOUSE Exonuclease 3-5 domain-containing protein 2 (Fragment) OS=Mus musculus OX=10090 GN=Exd2 PE=1 SV=1

21.42704 21.44333 20.71926 19.76252 24.98542 24.30932 24.7434 24.48763 + KO_Ctrl biological cell part;cell projection;cy toplas 2 2 2 8.3 8.3 8.3 43.478 0 10.768 27.19657 4 0 0 0 0 1 2 2 2 3.969346609 0.00955102 3.793403149 8.9693771 Q99N69 Q99N69 Leupaxin Lpxn sp|Q99N69|LPXN_MOUSE Leupaxin OS=Mus musculus OX=10090 GN=Lpxn PE=1 SV=2

23.308 23.41854 23.3254 24.35844 20.86574 19.46901 20.23508 20.26803 + KO_Ctrl cell part;intracellular membrane- 2 2 2 14.9 14.9 14.9 22.178 0 3.9892 25.39382 6 2 1 1 2 0 0 1 0 3.945021488 0.0104 -3.393128395 -8.880611 Q9CR10;H3BK0 Q9CR10; Oxidoreductase-lik Oxld1 sp|Q9CR10|OXLD1_MOUSE Oxidoreductase-like domain-containing protein 1 OS=Mus musculus OX=10090 GN=Oxld1 PE=1 SV=1;tr|H3BK04|H3BK04_MOUSE Oxidoreductase-like domain-containing protein 1 (Fragment) OS=Mus musculus OX=10090 GN=Oxld1 PE=1 SV=8

23.12027 24.26807 24.08278 23.17579 20.11472 20.73951 19.98362 20.84016 + KO_Ctrl establishmcell part;macromolec REACT_2 2 2 2 13.7 13.7 13.7 20.749 0 4.7269 25.85017 2 2 2 2 2 0 0 0 0 3.91709071 0.012470588 -3.242221355 -8.779639 A0A2I3BS49;Q9 A0A2I3BS Mitochondrial impo Timm23 tr|A0A2I3BS49|A0A2I3BS49_MOUSE Mitochondrial import inner membrane translocase subunit Tim23 OS=Mus musculus OX=10090 GN=Timm23 PE=1 SV=1;tr|Q9CXU4|Q9CXU4_MOUSE Mitochondrial import inner membrane translocase subunit TIM23 OS=Mus musculus OX=10090 GN=Timm2

21.14826 22.2755 21.29392 21.68699 23.735 24.16967 24.15182 23.85576 + KO_Ctrl cellular pr cell part;Golgi apparatus;intrace 9 9 9 16.4 16.4 16.4 87.337 0 39.002 27.06928 13 3 2 2 3 2 5 5 4 3.885570836 0.013307692 2.376896381 8.6668976 Q9CW79;A0A0N Q9CW79 Golgin subf amily Golga1 sp|Q9CW79|GOGA1_MOUSE Golgin subf amily A member 1 OS=Mus musculus OX=10090 GN=Golga1 PE=1 SV=2

19.76926 20.12755 21.65599 20.58333 23.89541 24.34442 24.15182 24.34807 + KO_Ctrl biological cell part;e Amy otrophic lateral s 1 1 1 4.4 4.4 4.4 28.834 0.000636 2.5471 26.72299 2 0 0 0 0 1 1 1 1 3.874801278 0.013056604 3.650896072 8.6286672 Q99J56 Q99J56 Derlin-1 Derl1 sp|Q99J56|DERL1_MOUSE Derlin-1 OS=Mus musculus OX=10090 GN=Derl1 PE=1 SV=1

20.29405 19.73695 20.95164 18.97851 23.5354 23.48296 23.73965 23.66405 + KO_Ctrl 3 3 3 9.2 9.2 9.2 49.401 0.000164 3.026 26.02333 5 0 0 0 0 1 2 2 3 3.852376073 0.012814815 3.61523056 8.5495315 D3Z286 D3Z286 Tmppe tr|D3Z286|D3Z286_MOUSE Transmembrane protein with metallophosphoesterase domain OS=Mus musculus OX=10090 GN=Tmppe PE=1 SV=2

22.26408 22.24394 21.88937 21.83688 20.4614 19.55907 19.73886 20.31719 + KO_Ctrl biological cell part;cy toplasm;intracellular 2 2 2 10.1 10.1 10.1 26.02 0.002459 2.0822 24.09015 3 2 2 1 1 0 0 0 0 3.772976545 0.013672727 -2.039439678 -8.274371 Q9CWV0 Q9CWV0 Mitochondrial asse Malsu1 sp|Q9CWV0|MASU1_MOUSE Mitochondrial assembly of ribosomal large subunit protein 1 OS=Mus musculus OX=10090 GN=Malsu1 PE=1 SV=1

23.62466 24.02462 24.34442 25.03322 19.82641 20.02488 21.20256 20.99221 + KO_Ctrl anatomica cell part;c Pathway s REACT_3 3 3 3 7.2 7.2 7.2 83.601 0 7.7247 26.95759 4 1 1 1 1 0 2 2 1 3.763005618 0.013428571 -3.745216846 -8.240362 Q8CEC2;E9QLT Q8CEC2; Ary l hy drocarbon r Arnt tr|Q8CEC2|Q8CEC2_MOUSE Ary l hy drocarbon receptor nuclear translocator OS=Mus musculus OX=10090 GN=Arnt PE=1 SV=1;tr|E9QLT6|E9QLT6_MOUSE Ary l hy drocarbon receptor nuclear translocator OS=Mus musculus OX=10090 GN=Arnt PE=1 SV=2;tr|Q3ULM2|Q3ULM2_MOUSE Ary l hy

24.37451 24.20068 24.71009 24.33702 21.22102 21.11243 19.21026 20.48319 + KO_Ctrl cell part;cy toskeleton;cy tosol;G 5 5 5 12.3 12.3 12.3 83.193 0 11.471 27.07521 10 1 2 3 3 2 1 1 1 3.757383787 0.013192982 -3.898848057 -8.221241 Q8C167;A0A3Q4 Q8C167 Proly l endopeptida Prepl sp|Q8C167|PPCEL_MOUSE Proly l endopeptidase-like OS=Mus musculus OX=10090 GN=Prepl PE=1 SV=1

19.95231 20.38956 22.13735 18.37037 26.74227 26.41796 26.69352 26.50685 + KO_Ctrl 3 3 3 9 9 9 70.046 0.001712 2.2388 29.06108 3 2 2 2 0 1 1 1 1 3.749665978 0.012965517 6.377750397 8.1950517 Q8BMC4 Q8BMC4 Nucleolar protein 9 Nop9 sp|Q8BMC4|NOP9_MOUSE Nucleolar protein 9 OS=Mus musculus OX=10090 GN=Nop9 PE=1 SV=1

20.55824 20.40462 21.89583 19.92751 24.14172 24.77394 24.14888 24.31417 + KO_Ctrl biological cell part;endoplasmic REACT_3 4 3 3 5.4 4.8 4.8 133.53 0.009871 1.4209 27.07684 5 2 0 0 0 1 1 1 1 3.739160994 0.012745763 3.648127079 8.15952 Q6DFW5;A0A0G Q6DFW5; Phospholipid-trans Atp11b tr|Q6DFW5|Q6DFW5_MOUSE Phospholipid-transporting ATPase OS=Mus musculus OX=10090 GN=Atp11b PE=1 SV=1;tr|A0A0G2JE89|A0A0G2JE89_MOUSE Phospholipid-transporting ATPase (Fragment) OS=Mus musculus OX=10090 GN=Atp11b PE=1 SV=1

19.73077 19.33988 20.67715 19.40971 22.14286 22.81282 22.7777 22.69053 + KO_Ctrl metabolic cell part;intracellular membrane- 2 2 2 25.2 25.2 25.2 13.43 0 3.1548 25.28701 7 0 0 0 0 1 2 2 2 3.737648871 0.012533333 2.816601753 8.1544163 Q9CWB7 Q9CWB7 Glutaredoxin-like protein C5or sp|Q9CWB7|Y D286_MOUSE Glutaredoxin-like protein C5orf 63 homolog OS=Mus musculus OX=10090 PE=1 SV=1

26.97487 26.99032 27.01903 27.06517 22.11159 21.10642 19.71679 18.33058 + KO_Ctrl biological cell body ; Fc gamm REACT_3 2 2 2 3.1 3.1 3.1 151.55 0 13.975 28.8039 6 2 2 2 2 0 0 0 0 3.728263328 0.013377049 -6.696001053 -8.1228 E9Q5G1;F8VQB E9Q5G1;F Unconv entional m My o10 tr|E9Q5G1|E9Q5G1_MOUSE Unconv entional my osin-X OS=Mus musculus OX=10090 GN=My o10 PE=1 SV=1;sp|F8VQB6|MY O10_MOUSE Unconv entional my osin-X OS=Mus musculus OX=10090 GN=My o10 PE=1 SV=1

19.36966 20.19112 19.64166 21.65739 24.35016 24.39585 24.97439 24.31693 + KO_Ctrl cellular m cell part;m mRNA surv eillance p 3 3 3 14.4 14.4 14.4 27.419 0.00126 2.407 27.22867 6 1 1 1 0 2 3 1 2 3.70621307 0.01316129 4.294373035 8.0489315 B2LVG5;B2LVG6 B2LVG5;B Cleav age and poly Cpsf 4 tr|B2LVG5|B2LVG5_MOUSE Cleav age and poly adeny lation specif icity f actor subunit 4 OS=Mus musculus OX=10090 GN=Cpsf 4 PE=1 SV=1;tr|B2LVG6|B2LVG6_MOUSE Cleav age and poly adeny lation specif icity f actor subunit 4 OS=Mus musculus OX=10090 GN=Cpsf 4 PE=1 SV=1;tr|E0C

19.50461 19.78819 21.12996 20.43719 23.44594 23.53801 23.03465 23.11913 + KO_Ctrl 3 3 3 17.8 17.8 17.8 20.146 0 3.4172 25.82243 3 0 1 0 0 1 2 1 1 3.700469487 0.012952381 3.069447994 8.029785 C0HK80;C0HK7 C0HK80;C0HK79 Arxes2 sp|C0HK80|ARXS2_MOUSE Adipocy te-related X-chromosome expressed sequence 2 OS=Mus musculus OX=10090 GN=Arxes2 PE=1 SV=1;sp|C0HK79|ARXS1_MOUSE Adipocy te-related X-chromosome expressed sequence 1 OS=Mus musculus OX=10090 GN=Arxes1 PE=1 SV=1

20.08302 21.72649 19.79976 20.32181 24.1168 23.83519 24.16269 23.8292 + KO_Ctrl anatomica cell part;membrane;plasma me 3 3 3 2.1 2.1 2.1 181.72 0.005071 1.7254 26.27341 3 0 0 0 0 2 2 3 2 3.69485467 0.01275 3.503199577 8.0111052 Q3LAC4 Q3LAC4 Phosphatidy linosit Prex2 sp|Q3LAC4|PREX2_MOUSE Phosphatidy linositol 3,4,5-trisphosphate-dependent Rac exchanger 2 protein OS=Mus musculus OX=10090 GN=Prex2 PE=1 SV=2

19.29831 20.54048 21.29059 20.14291 23.56256 23.70546 23.44342 23.78367 + KO_Ctrl biological cell part;cy toplasm;intracellular 9 1 1 8.4 1.8 1.8 234.41 0 3.3916 26.09925 2 0 0 0 0 1 1 1 1 3.64235884 0.013353846 3.305704117 7.8382371 A0A1W2P832;Q A0A1W2P Ral GTPase-activ a Ralgapa1 tr|A0A1W2P832|A0A1W2P832_MOUSE Ral GTPase-activ ating protein subunit alpha-1 OS=Mus musculus OX=10090 GN=Ralgapa1 PE=1 SV=1;sp|Q6GYP7|RGPA1_MOUSE Ral GTPase-activ ating protein subunit alpha-1 OS=Mus musculus OX=10090 GN=Ralgapa1 PE=1 SV=1

20.89674 19.35231 20.66953 20.14085 24.94695 24.45212 23.91752 23.25652 + KO_Ctrl biological cell part;endoplasmic reticulum; 4 4 4 10.1 10.1 10.1 90.093 0.000165 3.0861 30.04817 7 1 1 2 0 2 2 3 3 3.621416176 0.013151515 3.878420353 7.7701601 Q8BZ36 Q8BZ36 RAD50-interacting Rint1 sp|Q8BZ36|RINT1_MOUSE RAD50-interacting protein 1 OS=Mus musculus OX=10090 GN=Rint1 PE=1 SV=2

22.93884 22.47256 22.603 22.54985 19.58689 20.88874 20.61611 20.14018 + KO_Ctrl biological cell part;e Cy tokine- REACT_3 1 1 1 1.9 1.9 1.9 56.578 0.007879 1.4836 24.64136 1 1 1 1 1 0 0 0 0 3.593720582 0.013910448 -2.333082199 -7.680897 O35664 O35664 Interf eron alpha/be If nar2 sp|O35664|INAR2_MOUSE Interf eron alpha/beta receptor 2 OS=Mus musculus OX=10090 GN=If nar2 PE=1 SV=2

24.05821 24.13743 24.62906 23.62545 20.7578 19.4994 20.58697 18.53138 + KO_Ctrl biological cell part;m Gly cosy lp REACT_2 1 1 1 3.9 3.9 3.9 49.951 0 6.6963 26.24932 1 1 1 1 1 0 0 0 0 3.584033491 0.013705882 -4.268649101 -7.64988 Q3TAA8;Q8K358 Q3TAA8;Q Phosphatidy linosit Pigu tr|Q3TAA8|Q3TAA8_MOUSE Phosphatidy linositol gly can anchor biosy nthesis class U protein OS=Mus musculus OX=10090 GN=Pigu PE=1 SV=1;sp|Q8K358|PIGU_MOUSE Phosphatidy linositol gly can anchor biosy nthesis class U protein OS=Mus musculus OX=10090 GN=Pigu PE=1 SV=

21.29694 21.60609 19.24849 20.25642 24.66481 24.80055 24.57086 24.97018 + KO_Ctrl alcohol m cell part;intracellular REACT_2 3 3 3 20.7 20.7 20.7 20.123 0 3.739 27.28542 8 0 0 1 0 2 2 3 3 3.583355348 0.013507246 4.149619102 7.6477127 P46656;A0A1L1 P46656 Adrenodoxin, mito Fdx1 sp|P46656|ADX_MOUSE Adrenodoxin, mitochondrial OS=Mus musculus OX=10090 GN=Fdx1 PE=1 SV=1

20.71435 21.01074 21.1228 20.38202 24.01206 23.63323 22.92607 22.78535 + KO_Ctrl anatomica cell part;c Huntingto REACT_3 4 4 4 10 10 10 80.443 0 8.4375 26.07457 6 2 0 0 1 2 1 2 2 3.554614073 0.0152 2.531698227 7.5563239 G3X8Q0;O89090 G3X8Q0;O Transcription f acto Sp1 tr|G3X8Q0|G3X8Q0_MOUSE Transcription f actor Sp1 OS=Mus musculus OX=10090 GN=Sp1 PE=1 SV=1;sp|O89090|SP1_MOUSE Transcription f actor Sp1 OS=Mus musculus OX=10090 GN=Sp1 PE=1 SV=2

20.21366 19.53491 21.15477 20.51848 23.61908 22.81796 23.04388 23.58612 + KO_Ctrl biological cell part;centrosome;intracellula 10 10 10 6.2 6.2 6.2 243.72 0 27.321 26.12248 13 3 2 0 1 3 3 2 6 3.518479161 0.016676056 2.911305904 7.4427204 Q61043;A0A1Y 7 Q61043;A Ninein Nin sp|Q61043|NIN_MOUSE Ninein OS=Mus musculus OX=10090 GN=Nin PE=1 SV=4;tr|A0A1Y 7VNC5|A0A1Y 7VNC5_MOUSE Ninein OS=Mus musculus OX=10090 GN=Nin PE=1 SV=1

20.66014 20.75572 21.36814 21.0338 22.55253 23.65655 23.15103 22.87136 + KO_Ctrl cell part;nucleoplasm 3 3 3 5 5 5 96.062 0 7.738 26.69126 6 1 1 1 1 1 2 3 3 3.515571807 0.017333333 2.10341835 7.4336421 A0A2I3BQJ1;A0 A0A2I3BQ Serine/threonine-pr Ankrd28 tr|A0A2I3BQJ1|A0A2I3BQJ1_MOUSE Serine/threonine-protein phosphatase 6 regulatory anky rin repeat subunit A OS=Mus musculus OX=10090 GN=Ankrd28 PE=1 SV=1;tr|A0A2I3BQ07|A0A2I3BQ07_MOUSE Serine/threonine-protein phosphatase 6 regulatory anky rin repeat subunit

22.61865 23.56744 23.94601 22.92011 20.60164 20.75045 21.20814 20.5743 + KO_Ctrl biosy nthe cell part;intracellular REACT_2 2 2 2 5.2 5.2 5.2 49.939 0.001712 2.2392 25.56552 4 1 2 2 1 0 0 1 0 3.502089089 0.018027397 -2.479419708 -7.391662 Q9D0G0 Q9D0G0 28S ribosomal prot Mrps30 sp|Q9D0G0|RT30_MOUSE 28S ribosomal protein S30, mitochondrial OS=Mus musculus OX=10090 GN=Mrps30 PE=1 SV=1

20.54629 19.48987 21.71489 19.50413 24.46905 24.3694 24.02749 24.26184 + KO_Ctrl biological cell cortex;cell part;c REACT_2 5 5 5 3.5 3.5 3.5 229.72 0 4.4414 27.04781 7 0 0 1 0 3 4 3 3 3.501764907 0.017783784 3.968152046 7.3906552 Q811P8;S4R2G6 Q811P8 Rho GTPase-activ Arhgap32 sp|Q811P8|RHG32_MOUSE Rho GTPase-activ ating protein 32 OS=Mus musculus OX=10090 GN=Arhgap32 PE=1 SV=2

21.49199 19.31736 20.20984 20.10621 26.25618 24.49124 24.50094 24.43695 + KO_Ctrl biological cell part;membrane;plasma me 37 1 1 54.3 1.5 1.5 72.403 0.008307 1.4687 27.22656 2 0 0 0 0 1 1 1 1 3.482065858 0.018133333 4.639980793 7.3296798 B2RXQ9;D3Z0E B2RXQ9 Sorbs2 tr|B2RXQ9|B2RXQ9_MOUSE Sorbin and SH3 domain-containing protein 2 OS=Mus musculus OX=10090 GN=Sorbs2 PE=1 SV=1

24.01979 23.93391 25.0135 25.01052 18.25184 20.15163 19.9229 20.93952 + KO_Ctrl cell div isio cell part;intracellular membrane- 4 4 4 10 10 10 55.196 0 3.3518 27.1379 6 2 2 2 1 0 2 2 1 3.477142434 0.017894737 -4.677959442 -7.314505 Q80Y V2;H3BKM Q80Y V2;HNuclear-interacting Zc3hc1 sp|Q80Y V2|NIPA_MOUSE Nuclear-interacting partner of ALK OS=Mus musculus OX=10090 GN=Zc3hc1 PE=1 SV=1;tr|H3BKM2|H3BKM2_MOUSE Nuclear-interacting partner of ALK OS=Mus musculus OX=10090 GN=Zc3hc1 PE=1 SV=1;tr|D3Z3D0|D3Z3D0_MOUSE Nuclear-interacting partner o

23.94512 24.26571 24.99865 24.07692 19.16678 20.55674 19.72766 21.50282 + KO_Ctrl cell part;macromolecular compl 2 2 2 5.1 5.1 5.1 59.007 0 3.9001 26.47888 2 2 2 2 1 0 0 0 0 3.46666565 0.017662338 -4.083095074 -7.282301 Q3TH73 Q3TH73 Protein tweety homTty h2 sp|Q3TH73|TTY H2_MOUSE Protein tweety homolog 2 OS=Mus musculus OX=10090 GN=Tty h2 PE=1 SV=1

19.48201 21.92945 20.02202 20.61882 25.11668 25.04118 24.37862 24.20836 + KO_Ctrl 3 3 3 22.3 22.3 22.3 22.769 0 6.3708 27.20834 7 1 0 1 0 1 2 3 2 3.462123991 0.017435897 4.173138142 7.2683765 G5E8Q5;G5E8Q G5E8Q5;GDCN1-like protein; Dcun1d2 tr|G5E8Q5|G5E8Q5_MOUSE DCN1-like protein 2 OS=Mus musculus OX=10090 GN=Dcun1d2 PE=1 SV=1;tr|G5E8Q6|G5E8Q6_MOUSE DCN1-like protein 2 OS=Mus musculus OX=10090 GN=Dcun1d2 PE=1 SV=1;tr|A0A0N4SW97|A0A0N4SW97_MOUSE DCN1-like protein 2 OS=Mus musculus OX=10090 GN

19.70107 20.14514 21.17771 21.70404 24.42515 24.1397 24.0937 23.85557 + KO_Ctrl cellular pr cell part;intracellular REACT_2 2 2 2 22.1 22.1 22.1 16.284 0 3.717 27.52051 1 1 1 1 1 1 1 1 1 3.459326039 0.018025316 3.446538925 7.2598092 Q9DCC8 Q9DCC8 Mitochondrial impo Tomm20 sp|Q9DCC8|TOM20_MOUSE Mitochondrial import receptor subunit TOM20 homolog OS=Mus musculus OX=10090 GN=Tomm20 PE=1 SV=1

27.53771 27.68852 27.48915 27.53081 27.85733 27.89232 27.93528 27.90782 + KO_Ctrl biological cell part;intracellular membrane- 13 13 13 20.3 20.3 20.3 127.77 0 64.564 31.27236 55 10 9 7 9 8 10 10 9 3.447999711 0.0194 0.336639881 7.2252128 A6PWC3;A2A9Q A6PWC3; Nardily sin Nrd1 tr|A6PWC3|A6PWC3_MOUSE Nardily sin, N-arginine dibasic conv ertase, NRD conv ertase 1 OS=Mus musculus OX=10090 GN=Nrd1 PE=1 SV=1;tr|A2A9Q2|A2A9Q2_MOUSE Nardily sin, N-arginine dibasic conv ertase, NRD conv ertase 1 OS=Mus musculus OX=10090 GN=Nrd1 PE=1 SV=1;sp|Q

20.81475 20.30515 20.99749 21.28004 18.82056 19.14962 18.85319 18.16776 + KO_Ctrl cellular pr cell part;membrane 2 1 1 7.4 4.2 4.2 47.244 0.001264 2.4627 21.33336 4 0 0 0 0 1 1 1 1 3.443523641 0.019160494 -2.101574898 -7.211578 Q3UED7 Q3UED7 Gm4951 tr|Q3UED7|Q3UED7_MOUSE Interf eron-gamma-inducible GTPase If gga2 protein OS=Mus musculus OX=10090 GN=Gm4951 PE=1 SV=1

26.74766 25.91459 25.42518 26.02897 21.26189 18.82785 22.04556 19.70416 + KO_Ctrl biological cell part;cy toplasm;intracellular 15 3 3 41.9 14.1 14.1 49.067 0 9.9849 28.04661 2 3 3 2 2 0 0 0 0 3.422257822 0.018926829 -5.569231987 -7.147087 Q8VDW0;D6RH Q8VDW0 ATP-dependent RNDdx39a sp|Q8VDW0|DX39A_MOUSE ATP-dependent RNA helicase DDX39A OS=Mus musculus OX=10090 GN=Ddx39a PE=1 SV=1

23.20084 25.3141 24.04034 24.04718 20.18293 19.5644 20.55808 21.06248 + KO_Ctrl anatomica cell part;cy toplasm;cy tosol;intr 3 3 3 1.8 1.8 1.8 182.35 0.009453 1.4359 26.3658 3 2 1 3 1 0 0 0 0 3.399366624 0.021012048 -3.808644295 -7.078194 Q9ESK9;F7CC5 Q9ESK9;F RB1-inducible coile Rb1cc1 sp|Q9ESK9|RBCC1_MOUSE RB1-inducible coiled-coil protein 1 OS=Mus musculus OX=10090 GN=Rb1cc1 PE=1 SV=3;tr|F7CC56|F7CC56_MOUSE RB1-inducible coiled-coil protein 1 (Fragment) OS=Mus musculus OX=10090 GN=Rb1cc1 PE=1 SV=1;tr|F7CCJ3|F7CCJ3_MOUSE RB1-inducible c

25.38906 24.50773 24.65009 24.92556 21.76154 18.75289 20.5045 19.53758 + KO_Ctrl biological cell part;cy toplasm;intracellular 3 3 3 17.5 17.5 17.5 22.517 0 5.8738 26.86207 10 3 2 2 3 1 0 0 0 3.367883556 0.02152381 -4.72898531 -6.984328 Q9CY 97 Q9CY 97 RNA poly merase I Ssu72 sp|Q9CY 97|SSU72_MOUSE RNA poly merase II subunit A C-terminal domain phosphatase SSU72 OS=Mus musculus OX=10090 GN=Ssu72 PE=1 SV=1

24.28757 25.18918 24.23674 24.32231 19.87368 22.07882 20.91246 20.22359 + KO_Ctrl biological cell body ; Mineral ab REACT_3 1 1 1 14.6 14.6 14.6 14.486 0.002173 2.2061 26.5451 1 1 1 1 1 0 0 0 0 3.360766314 0.022023529 -3.736813068 -6.963249 A8Y 5P1;Q8K211 A8Y 5P1;Q High af f inity copp Slc31a1 tr|A8Y 5P1|A8Y 5P1_MOUSE Copper transporter OS=Mus musculus OX=10090 GN=Slc31a1 PE=1 SV=1;sp|Q8K211|COPT1_MOUSE High af f inity copper uptake protein 1 OS=Mus musculus OX=10090 GN=Slc31a1 PE=2 SV=1

21.23576 20.1435 20.6037 22.13903 24.43148 23.76213 24.35743 24.6525 + KO_Ctrl biological cell part;c Basal tran REACT_2 1 1 1 18.1 18.1 18.1 14.09 0.000164 2.9868 26.66397 1 0 0 0 0 1 1 1 1 3.348806663 0.021767442 3.270386219 6.9279433 A0A087WSE8;Q A0A087W General transcripti Gtf 2e2 tr|A0A087WSE8|A0A087WSE8_MOUSE General transcription f actor IIE subunit 2 (Fragment) OS=Mus musculus OX=10090 GN=Gtf 2e2 PE=1 SV=1;sp|Q9D902|T2EB_MOUSE General transcription f actor IIE subunit 2 OS=Mus musculus OX=10090 GN=Gtf 2e2 PE=1 SV=2

20.03981 19.76958 21.13637 18.07202 23.88865 24.65992 24.96974 24.23125 + KO_Ctrl cellular co cell part;centrosome; REACT_2 2 2 2 6.9 6.9 6.9 71.309 0 7.195 27.00303 4 0 0 0 0 2 2 2 2 3.345477419 0.021517241 4.682944775 6.918141 Q8BKL6;Q8BY N Q8BKL6;Q Gamma-tubulin co Tubgcp4 tr|Q8BKL6|Q8BKL6_MOUSE Gamma-tubulin complex component OS=Mus musculus OX=10090 GN=Tubgcp4 PE=1 SV=1;tr|Q8BY N2|Q8BYN2_MOUSE Gamma-tubulin complex component OS=Mus musculus OX=10090 GN=Tubgcp4 PE=1 SV=1;sp|Q9D4F8|GCP4_MOUSE Gamma-tubulin complex component 4

23.26914 24.05862 24.20814 23.84479 19.91763 21.00533 21.78177 20.78546 + KO_Ctrl anatomica cell part;m Long-term REACT_2 5 5 5 17.9 17.9 17.9 44.095 0 3.4366 26.36511 5 1 3 3 1 1 1 1 1 3.315482471 0.022 -2.972628593 -6.830329 P27600;A0A0G2 P27600;A Guanine nucleotide Gna12 sp|P27600|GNA12_MOUSE Guanine nucleotide-binding protein subunit alpha-12 OS=Mus musculus OX=10090 GN=Gna12 PE=1 SV=3;tr|A0A0G2JG40|A0A0G2JG40_MOUSE Guanine nucleotide-binding protein subunit alpha-12 (Fragment) OS=Mus musculus OX=10090 GN=Gna12 PE=1 SV=1

21.11304 20.77113 19.8479 19.36487 23.46326 22.96229 22.93647 23.45362 + KO_Ctrl anatomica cell part;intracellular membrane- 1 1 1 12.3 12.3 12.3 9.1541 0.001715 2.2552 25.6248 3 0 0 0 0 1 1 1 1 3.309628476 0.022247191 2.929673195 6.8132965 A0A494BAZ3;Q8 A0A494BA Protein DPCD Dpcd tr|A0A494BAZ3|A0A494BAZ3_MOUSE Predicted gene 17018 OS=Mus musculus OX=10090 GN=Gm17018 PE=1 SV=1;sp|Q8BPA8|DPCD_MOUSE Protein DPCD OS=Mus musculus OX=10090 GN=Dpcd PE=1 SV=1

20.52184 18.49047 20.832 20.01613 23.48148 23.31202 23.71188 23.71513 + KO_Ctrl catabolic cell part;c Arachidon REACT_2 3 3 3 10.8 10.8 10.8 57.935 0 4.5647 26.06495 5 0 0 0 0 1 1 3 2 3.302037026 0.022 3.590016365 6.791259 Q924D1;B1AWM Q924D1 Cy p2j9 tr|Q924D1|Q924D1_MOUSE Cy tochrome P450 CYP2J9 OS=Mus musculus OX=10090 GN=Cy p2j9 PE=1 SV=1

22.99132 23.11022 24.14981 22.83459 20.30303 18.6013 20.23234 20.20235 + KO_Ctrl cellular m cell part;c Ubiquitin mediated pr 3 3 3 27 27 27 21.11 0 9.5381 26.6679 6 2 2 1 2 0 1 1 0 3.291546712 0.022857143 -3.436730385 -6.7609 Q9CY 34;Q3UW Q9CY 34 NEDD8-conjugatin Ube2f sp|Q9CY 34|UB2FA_MOUSE NEDD8-conjugating enzy me UBE2F OS=Mus musculus OX=10090 GN=Ube2f PE=1 SV=1

19.47533 21.27955 21.39393 18.92633 24.95136 24.43981 24.37159 24.59679 + KO_Ctrl establishmcell part;membrane 3 3 3 4.8 4.8 4.8 101.7 0.007194 1.5547 27.16816 3 0 0 1 1 1 1 1 2 3.287173241 0.022608696 4.321103096 6.7482756 G5E833;Q9ER64 G5E833;Q Oxy sterol-binding Osbpl5 tr|G5E833|G5E833_MOUSE Oxy sterol-binding protein OS=Mus musculus OX=10090 GN=Osbpl5 PE=1 SV=1;sp|Q9ER64|OSBL5_MOUSE Oxy sterol-binding protein-related protein 5 OS=Mus musculus OX=10090 GN=Osbpl5 PE=1 SV=3

20.31516 21.0709 20.55417 21.28647 23.59651 22.83648 23.03444 22.48458 + KO_Ctrl biological cell body ;cell part;DNA-directed 3 3 3 14.5 14.5 14.5 41.236 0 9.0763 25.66725 5 0 0 0 0 1 3 2 3 3.286833836 0.022365591 2.181328773 6.7472966 Q6P6I6 Q6P6I6 DNA-directed RNA Polr2m sp|Q6P6I6|GRL1A_MOUSE DNA-directed RNA poly merase II subunit GRINL1A OS=Mus musculus OX=10090 GN=Polr2m PE=2 SV=2

22.93801 23.19015 23.04308 23.75032 21.58367 20.70386 20.61311 20.00548 + KO_Ctrl biological cell part;c RNA degr REACT_3 2 2 2 7.6 7.6 7.6 62.693 0 3.7421 25.10173 2 2 1 1 1 0 0 0 0 3.281195106 0.02212766 -2.50385952 -6.731049 B9EIX0;Q3U564 B9EIX0;Q mRNA-decapping Dcp1b tr|B9EIX0|B9EIX0_MOUSE DCP1 decapping enzy me homolog b (S. cerev isiae) OS=Mus musculus OX=10090 GN=Dcp1b PE=1 SV=1;sp|Q3U564|DCP1B_MOUSE mRNA-decapping enzy me 1B OS=Mus musculus OX=10090 GN=Dcp1b PE=1 SV=1

23.40328 22.90045 23.18306 23.61279 21.19422 19.46309 20.46977 19.29071 + KO_Ctrl 2 2 2 23 23 23 13.331 0.00231 2.1032 25.20262 2 2 2 1 1 0 0 0 0 3.276118605 0.022442105 -3.17044735 -6.716449 Q9CZH3 Q9CZH3 Proteasome assemPsmg3 sp|Q9CZH3|PSMG3_MOUSE Proteasome assembly chaperone 3 OS=Mus musculus OX=10090 GN=Psmg3 PE=1 SV=1

27.90959 27.56957 28.24921 24.66904 20.59453 20.7 20.18752 22.13869 + KO_Ctrl extracellul Alzheimer disease; 7 1 1 49.7 24.8 24.8 16.701 0 4.7922 29.78032 3 1 1 1 1 0 0 0 0 3.267557069 0.02275 -6.194168091 -6.691882 Q9D6P8 Q9D6P8 Calmodulin-like pro Calml3 sp|Q9D6P8|CALL3_MOUSE Calmodulin-like protein 3 OS=Mus musculus OX=10090 GN=Calml3 PE=2 SV=1

22.44975 23.88474 23.99602 22.99381 20.64778 21.09369 20.48093 20.52178 + KO_Ctrl biological cell part;c Jak-STAT signaling p 2 2 2 5.4 5.4 5.4 50.664 0 3.1769 25.61869 2 2 2 2 1 0 0 0 0 3.266869832 0.022515464 -2.645036221 -6.689913 Q924S8 Q924S8 Sprouty -related, E Spred1 sp|Q924S8|SPRE1_MOUSE Sprouty -related, EVH1 domain-containing protein 1 OS=Mus musculus OX=10090 GN=Spred1 PE=1 SV=1

22.53476 23.09149 22.70683 22.67947 21.2057 19.56149 20.17021 19.73619 + KO_Ctrl dev elopm cell part Wnt signaling pathwa 3 3 3 13.2 13.2 13.2 54.365 0 11.94 25.09716 6 3 1 1 1 1 0 1 1 3.26066414 0.022816327 -2.584741116 -6.672155 Q3UXU7;Q80Z9 Q3UXU7; Vang-like protein 1 Vangl1 tr|Q3UXU7|Q3UXU7_MOUSE Vang-like protein OS=Mus musculus OX=10090 GN=Vangl1 PE=1 SV=1;sp|Q80Z96|VANG1_MOUSE Vang-like protein 1 OS=Mus musculus OX=10090 GN=Vangl1 PE=1 SV=2;tr|A0A0H2UH25|A0A0H2UH25_MOUSE Vang-like protein 1 OS=Mus musculus OX=10090 GN=Vang

24.17394 24.78551 25.20292 24.7638 20.43305 21.60408 19.36021 21.72981 + KO_Ctrl biological cell body ;cell part;cell projection 7 7 7 25 25 25 39.835 0 7.821 27.3955 8 2 3 5 1 1 1 2 1 3.247084857 0.023232323 -3.949756622 -6.633427 Q8VCM5 Q8VCM5 Mitochondrial ubiqu Mul1 sp|Q8VCM5|MUL1_MOUSE Mitochondrial ubiquitin ligase activ ator of NFKB 1 OS=Mus musculus OX=10090 GN=Mul1 PE=1 SV=2

23.78207 23.11364 23.48505 22.97706 21.59026 19.96582 20.13239 19.82557 + KO_Ctrl biosy nthe cell part;e Py rimidine REACT_3 3 3 3 31.5 31.5 31.5 17.384 0.000639 2.6413 25.61661 4 2 1 1 1 0 0 1 0 3.240541295 0.023 -2.96094656 -6.614829 Q9CQ43;Q8VCG Q9CQ43;Q8VCG1 Dut tr|Q9CQ43|Q9CQ43_MOUSE Deoxy uridine 5-triphosphate nucleotidohy drolase OS=Mus musculus OX=10090 GN=Dut PE=1 SV=1;tr|Q8VCG1|Q8VCG1_MOUSE Deoxy uridine 5-triphosphate nucleotidohy drolase OS=Mus musculus OX=10090 GN=Dut PE=1 SV=1

19.96138 19.19254 21.23183 21.26181 23.68752 24.02563 24.08448 23.55918 + KO_Ctrl cell part;intracellular REACT_2 3 3 3 11.3 11.3 11.3 49.299 0 6.0241 26.55986 7 0 0 0 0 1 3 3 2 3.219309 0.023287129 3.427313328 6.554765 Q5NCE8 Q5NCE8 Magnesium transp Mrs2 sp|Q5NCE8|MRS2_MOUSE Magnesium transporter MRS2 homolog, mitochondrial OS=Mus musculus OX=10090 GN=Mrs2 PE=2 SV=2

20.12453 20.36079 21.15759 20.36275 24.13281 22.47813 23.12166 23.0418 + KO_Ctrl anatomica cell part;cell projection;cy toskel 4 3 3 4.4 3.3 3.3 108.29 0.00464 1.7572 25.67777 5 1 1 0 0 1 2 2 1 3.213843091 0.023058824 2.692185879 6.5393725 Q3TN34;E9PZD Q3TN34;E MICAL-like protein Micall2 sp|Q3TN34|MILK2_MOUSE MICAL-like protein 2 OS=Mus musculus OX=10090 GN=Micall2 PE=1 SV=1;tr|E9PZD2|E9PZD2_MOUSE MICAL-like protein 2 OS=Mus musculus OX=10090 GN=Micall2 PE=1 SV=1

24.48616 24.70409 24.16461 24.70915 18.50408 20.14365 19.94853 21.75834 + KO_Ctrl cell death;cellular process;death 2 2 2 16.2 16.2 16.2 26.353 0.000638 2.6261 26.45527 6 2 2 2 2 0 0 0 1 3.210048967 0.024737864 -4.427349567 -6.528705 A0A0U1RNX8;O A0A0U1R B-cell CLL/ly mpho Bcl7c tr|A0A0U1RNX8|A0A0U1RNX8_MOUSE B-cell CLL/ly mphoma 7 protein f amily member C OS=Mus musculus OX=10090 GN=Bcl7c PE=1 SV=1;sp|O08664|BCL7C_MOUSE B-cell CLL/ly mphoma 7 protein f amily member C OS=Mus musculus OX=10090 GN=Bcl7c PE=1 SV=1

22.02433 20.73706 19.07306 20.79528 25.65363 24.77188 24.57762 24.69376 + KO_Ctrl biological apical part Calcium signaling pa 4 4 4 20.1 20.1 20.1 38.139 0 5.298 27.33986 4 0 1 1 1 2 2 1 3 3.208449898 0.0245 4.266787052 6.5242126 D3Y YR5;Q9Z25 D3YYR5; P2X purinoceptor;P P2rx4 tr|D3YYR5|D3YYR5_MOUSE ATP receptor OS=Mus musculus OX=10090 GN=P2rx4 PE=1 SV=2;tr|Q9Z256|Q9Z256_MOUSE P2X purinoceptor OS=Mus musculus OX=10090 GN=P2rx4 PE=1 SV=1;tr|D3Z5U5|D3Z5U5_MOUSE P2X purinoceptor OS=Mus musculus OX=10090 GN=P2rx4 PE=1 SV=2;tr|Q9Z25

23.67325 23.59525 23.79054 23.68635 21.78112 19.96698 20.98163 19.76506 + KO_Ctrl biological cell part;e Protein processing in 2 2 2 32.5 32.5 32.5 8.3635 0 7.5319 26.07087 2 1 1 1 1 1 1 0 0 3.20352154 0.024266667 -3.062650681 -6.510383 Q3UZP4 Q3UZP4 Small VCP/p97-int Sv ip sp|Q3UZP4|SVIP_MOUSE Small VCP/p97-interacting protein OS=Mus musculus OX=10090 GN=Sv ip PE=3 SV=1

23.46026 23.33224 23.29151 23.46849 19.89363 20.5975 20.4897 21.85017 + KO_Ctrl cell dif f er cell part;macromolec REACT_2 1 1 1 17.1 17.1 17.1 16.461 0.002604 2.0342 25.3502 3 1 1 1 1 0 0 0 0 3.193474905 0.024037736 -2.680379391 -6.482264 G3UVU6;Q9R0X G3UVU6; Mediator of RNA p Gm20517; tr|G3UVU6|G3UVU6_MOUSE Mediator of RNA poly merase II transcription subunit 20 OS=Mus musculus OX=10090 GN=Gm20517 PE=3 SV=1;sp|Q9R0X0|MED20_MOUSE Mediator of RNA poly merase II transcription subunit 20 OS=Mus musculus OX=10090 GN=Med20 PE=1 SV=1

22.77227 22.49819 22.93359 23.17175 20.66028 20.08971 21.47304 20.17769 + KO_Ctrl 1 1 1 9.6 9.6 9.6 12.336 0.007192 1.5536 24.7761 2 1 1 1 1 0 0 0 0 3.187843316 0.023813084 -2.243768692 -6.466543 A0A571BE82;A0 A0A571BE82;A0A571BEH6;A Ccdc30 tr|A0A571BE82|A0A571BE82_MOUSE Coiled-coil domain-containing protein 30 OS=Mus musculus OX=10090 GN=Ccdc30 PE=4 SV=1;tr|A0A571BEH6|A0A571BEH6_MOUSE Coiled-coil domain-containing protein 30 OS=Mus musculus OX=10090 GN=Ccdc30 PE=4 SV=1;tr|A0A571BGB9|A0A571BG

18.95649 19.6701 20.51289 20.87499 22.81372 22.76701 22.62605 22.9828 + KO_Ctrl anatomica cell part;intracellular non-membr 1 1 1 7.3 7.3 7.3 28.543 0 3.3278 25.2367 1 0 0 0 0 1 1 1 1 3.161557109 0.025962963 2.793778419 6.3935578 Q9D8M4 Q9D8M4 60S ribosomal prot Rpl7l1 sp|Q9D8M4|RL7L_MOUSE 60S ribosomal protein L7-like 1 OS=Mus musculus OX=10090 GN=Rpl7l1 PE=1 SV=1

23.54793 24.84417 24.64411 23.1495 20.4849 20.88021 19.94432 21.47647 + KO_Ctrl biological cell part;cell projection;cy toplas 19 1 1 52.2 3.9 3.9 63.138 0.001567 2.327 26.36471 2 1 1 1 1 0 0 0 0 3.159845855 0.025724771 -3.349951267 -6.388829 F8SLP9;F8SLQ3 F8SLP9;F PEX5-related prote Pex5l tr|F8SLP9|F8SLP9_MOUSE PEX5-related protein OS=Mus musculus OX=10090 GN=Pex5l PE=1 SV=1;tr|F8SLQ3|F8SLQ3_MOUSE PEX5-related protein OS=Mus musculus OX=10090 GN=Pex5l PE=1 SV=1;tr|F8SLQ1|F8SLQ1_MOUSE PEX5-related protein OS=Mus musculus OX=10090 GN=Pex5l PE

22.65921 23.14443 22.60173 23.0851 20.9749 19.83923 18.69889 19.49942 + KO_Ctrl biological cell part;membrane;plasma me 1 1 1 6.5 6.5 6.5 33.221 0 6.0428 24.78676 1 1 1 1 1 0 0 0 0 3.138675873 0.026836364 -3.119507313 -6.33055 Q6PGA2;O88667 Q6PGA2; GTP-binding protei Rrad tr|Q6PGA2|Q6PGA2_MOUSE GTP-binding protein OS=Mus musculus OX=10090 GN=Rrad PE=1 SV=1;sp|O88667|RAD_MOUSE GTP-binding protein RAD OS=Mus musculus OX=10090 GN=Rrad PE=1 SV=1

22.66711 21.91747 21.98256 22.32696 20.45063 20.99509 20.93156 21.0055 + KO_Ctrl anatomica cell part;cy toplasm 2 2 2 6 6 6 40.32 0.004509 1.8044 24.5131 2 1 1 1 1 0 1 1 1 3.134501852 0.026594595 -1.377830029 -6.319109 Q3UCV8;A0A2I3 Q3UCV8; Ubiquitin thioester Otulin sp|Q3UCV8|OTUL_MOUSE Ubiquitin thioesterase otulin OS=Mus musculus OX=10090 GN=Otulin PE=1 SV=1;tr|A0A2I3BRS5|A0A2I3BRS5_MOUSE Ubiquitin thioesterase otulin (Fragment) OS=Mus musculus OX=10090 GN=Otulin PE=1 SV=1

19.5264 20.49161 20.456 20.82121 25.57868 25.55066 23.51256 23.39532 + KO_Ctrl alcohol m extracellular space;in REACT_3 4 4 4 16.6 16.6 16.6 30.372 0 15.998 27.62038 6 0 0 0 0 3 4 1 1 3.107096501 0.027178571 4.185503483 6.2443818 Q07079 Q07079 Insulin-like growth Igf bp5 sp|Q07079|IBP5_MOUSE Insulin-like growth f actor-binding protein 5 OS=Mus musculus OX=10090 GN=Igf bp5 PE=1 SV=1

21.25718 20.64483 20.7667 19.27925 28.37447 30.106 26.01604 25.60334 + KO_Ctrl cell part;extracellular REACT_3 19 19 19 48 48 48 53.326 0 84.483 31.42852 41 0 0 0 0 9 18 8 5 3.087374576 0.028707965 7.037973404 6.1910277 P47867;A0A1L1 P47867 Secretogranin-3 Scg3 sp|P47867|SCG3_MOUSE Secretogranin-3 OS=Mus musculus OX=10090 GN=Scg3 PE=1 SV=1

23.78437 23.67896 23.67411 23.24016 21.91159 19.91368 21.04928 20.40303 + KO_Ctrl 2 2 2 7.8 7.8 7.8 47.416 0 3.1517 25.65968 3 2 2 1 1 0 0 0 0 3.085679421 0.02845614 -2.775003433 -6.186458 Q922R1 Q922R1 UPF0183 protein C16orf 70 h sp|Q922R1|CP070_MOUSE UPF0183 protein C16orf 70 homolog OS=Mus musculus OX=10090 PE=1 SV=2

24.30911 23.50749 24.0694 23.4148 19.93372 20.51172 21.37051 21.82294 + KO_Ctrl anatomica cell part;intracellular membrane- 4 4 4 22.5 22.5 22.5 22.03 0 4.3536 25.79835 6 3 2 1 2 0 0 1 0 3.062117611 0.029669565 -2.915478706 -6.12321 Q08024 Q08024 Core-binding f acto Cbf b sp|Q08024|PEBB_MOUSE Core-binding f actor subunit beta OS=Mus musculus OX=10090 GN=Cbf b PE=1 SV=1

19.79542 19.92663 20.84907 22.38997 26.14137 27.98212 25.25947 25.04552 + KO_Ctrl biological cell part;c Tight junction 4 3 3 21.9 18.3 18.3 24.757 0 13.66 29.41818 5 0 0 0 0 1 3 1 1 3.009835413 0.033896552 5.366847038 5.984621 Q9CZT8;A2A7Z6 Q9CZT8;ARas-related protein Rab3b sp|Q9CZT8|RAB3B_MOUSE Ras-related protein Rab-3B OS=Mus musculus OX=10090 GN=Rab3b PE=1 SV=1;tr|A2A7Z6|A2A7Z6_MOUSE Ras-related protein Rab-3B OS=Mus musculus OX=10090 GN=Rab3b PE=1 SV=1

20.15105 21.20956 21.16768 19.63963 23.82813 23.51075 22.59585 23.40419 + KO_Ctrl biological cell part;cy toplasm;i REACT_2 5 3 3 6.4 5.4 5.4 105.38 0 3.3714 25.52109 3 0 0 1 0 1 1 2 1 3.002787337 0.034700855 2.792748928 5.9661207 Q99K46 Q99K46 Ubiquitin carboxy l- Usp11 sp|Q99K46|UBP11_MOUSE Ubiquitin carboxy l-terminal hy drolase 11 OS=Mus musculus OX=10090 GN=Usp11 PE=1 SV=4

20.75383 21.94249 19.48253 20.42555 23.92379 23.36078 24.16385 23.90003 + KO_Ctrl 3 3 3 8.8 8.8 8.8 46.338 0.00157 2.347 26.48373 3 1 1 0 1 2 2 2 2 2.996925122 0.03440678 3.186012268 5.9507659 Q80UY 1;A0A494 Q80UY 1 UPF0586 protein C9orf 41 ho sp|Q80UY 1|CARME_MOUSE Carnosine N-methy ltransf erase OS=Mus musculus OX=10090 GN=Carnmt1 PE=1 SV=1

26.10197 25.94911 26.05354 25.95607 26.81054 26.54261 26.39751 26.5401 + KO_Ctrl cell part;cy toplasm;intracellular 14 14 14 23.8 23.8 23.8 81.163 0 27.216 29.59527 37 9 6 4 6 8 9 9 9 2.98873462 0.036840336 0.557516098 5.9293622 Q3TVI8;D3Y Y 15 Q3TVI8 Pre-B-cell leukemi Pbxip1 sp|Q3TVI8|PBIP1_MOUSE Pre-B-cell leukemia transcription f actor-interacting protein 1 OS=Mus musculus OX=10090 GN=Pbxip1 PE=1 SV=2

22.49568 22.95397 23.92958 22.2199 20.31834 20.81177 20.62706 19.99957 + KO_Ctrl cellular co cell part;endoplasmic reticulum; 1 1 1 6.6 6.6 6.6 21.102 0.000163 2.9377 25.20568 1 1 1 1 1 0 0 0 0 2.986323781 0.0369 -2.460598946 -5.923073 Q5XKN4 Q5XKN4 Protein jagunal ho Jagn1 sp|Q5XKN4|JAGN1_MOUSE Protein jagunal homolog 1 OS=Mus musculus OX=10090 GN=Jagn1 PE=1 SV=2

22.07676 22.82452 22.44316 23.41015 19.97921 19.93821 20.59233 21.05106 + KO_Ctrl anatomica cell part;cell projection;intracellu 1 1 1 1.7 1.7 1.7 73.373 0.000635 2.5217 24.62355 3 1 1 1 1 0 0 0 0 2.975373422 0.037652893 -2.298445702 -5.89457 P0C7T6 P0C7T6 Ataxin-1-like Atxn1l sp|P0C7T6|ATX1L_MOUSE Ataxin-1-like OS=Mus musculus OX=10090 GN=Atxn1l PE=1 SV=1

20.3553 19.93437 21.69164 20.67227 22.71915 22.90879 23.17185 22.945 + KO_Ctrl biological cell part;cy toplasm;macromolec 3 3 3 6.2 6.2 6.2 83.305 0.000642 2.7008 25.53851 5 0 0 0 0 1 3 3 1 2.973328338 0.037344262 2.27280426 5.8892581 Q6PHQ8;E9Q2U Q6PHQ8; N-alpha-acety ltran Naa35 sp|Q6PHQ8|NAA35_MOUSE N-alpha-acety ltransf erase 35, NatC auxiliary subunit OS=Mus musculus OX=10090 GN=Naa35 PE=1 SV=1;tr|E9Q2U4|E9Q2U4_MOUSE N-alpha-acety ltransf erase 35, NatC auxiliary subunit (Fragment) OS=Mus musculus OX=10090 GN=Naa35 PE=1 SV=1;tr|E9Q

20.65441 19.29964 21.21075 21.33265 24.04993 23.45186 23.95819 23.10933 + KO_Ctrl biological cell part;cy toplasm;i REACT_3 3 3 3 5.1 5.1 5.1 76.956 0 3.9669 25.92286 4 0 0 0 0 3 1 2 3 2.960355155 0.037560976 3.017965794 5.8556446 Q8BWR8 Q8BWR8 Rhophilin-2 Rhpn2 sp|Q8BWR8|RHPN2_MOUSE Rhophilin-2 OS=Mus musculus OX=10090 GN=Rhpn2 PE=1 SV=2

19.48609 19.91102 21.51694 20.23512 22.56412 23.7167 23.43275 24.04527 + KO_Ctrl cell motilit cell part;endosome;intracellular 3 3 3 5.5 5.5 5.5 72.185 0 7.3998 26.30095 7 0 0 0 0 2 3 3 1 2.950473836 0.037258065 3.152414322 5.830138 Q80U56 Q80U56 Late secretory pat Av l9 sp|Q80U56|AVL9_MOUSE Late secretory pathway protein AVL9 homolog OS=Mus musculus OX=10090 GN=Av l9 PE=1 SV=2

21.98428 20.05707 21.22872 21.16691 23.32704 23.45739 23.40847 23.82085 + KO_Ctrl biosy nthe cell part;cy toplasm;intracellular 4 4 4 10 10 10 74.575 0 5.184 26.89076 5 1 1 1 0 2 2 2 1 2.945074416 0.03696 2.394190788 5.8162352 H9KV04;Q8R3C H9KV04;Q Mini-chromosome Mcmbp tr|H9KV04|H9KV04_MOUSE Mini-chromosome maintenance complex-binding protein OS=Mus musculus OX=10090 GN=Mcmbp PE=1 SV=1;sp|Q8R3C0|MCMBP_MOUSE Mini-chromosome maintenance complex-binding protein OS=Mus musculus OX=10090 GN=Mcmbp PE=1 SV=1

20.84101 21.37069 21.63391 19.96215 22.89139 23.36653 23.2014 23.16759 + KO_Ctrl biosy nthe cell part;intracellular REACT_2 1 1 1 4.3 4.3 4.3 23.366 0.002607 2.0537 25.71618 1 0 0 0 0 1 1 1 1 2.929086272 0.03815873 2.204789162 5.7752118 Q9D1N9 Q9D1N9 39S ribosomal prot Mrpl21 sp|Q9D1N9|RM21_MOUSE 39S ribosomal protein L21, mitochondrial OS=Mus musculus OX=10090 GN=Mrpl21 PE=1 SV=1

23.25667 23.2213 23.54227 23.27355 23.84699 23.97745 23.72983 24.06521 biological cell part;cy toskeleton;intracellul 2 2 2 10.1 10.1 10.1 22.345 0 3.4963 26.82736 8 2 2 2 1 2 1 1 2 2.852293979 0.05215748 0.581423283 5.5811202 A0A1B0GRZ8;Q A0A1B0G ELMO domain-con Elmod2 tr|A0A1B0GRZ8|A0A1B0GRZ8_MOUSE ELMO domain-containing protein 2 (Fragment) OS=Mus musculus OX=10090 GN=Elmod2 PE=1 SV=1;sp|Q8BGF6|ELMD2_MOUSE ELMO domain-containing protein 2 OS=Mus musculus OX=10090 GN=Elmod2 PE=1 SV=1

20.4419 19.94461 21.52575 20.44479 22.72167 22.52464 23.88642 23.28362 amine me cell part;cy tosol 1 1 1 47.1 47.1 47.1 5.9886 0.000163 2.8884 25.70841 5 0 0 0 0 1 1 1 1 2.840885732 0.052625 2.51482439 5.5526953 H3BKG8;H3BLA H3BKG8;H3BLA5 Ahcy l2 tr|H3BKG8|H3BKG8_MOUSE Putativ e adenosy lhomocy steinase 3 (Fragment) OS=Mus musculus OX=10090 GN=Ahcy l2 PE=1 SV=1;tr|H3BLA5|H3BLA5_MOUSE Putativ e adenosy lhomocy steinase 3 (Fragment) OS=Mus musculus OX=10090 GN=Ahcy l2 PE=1 SV=1

21.91195 20.2308 19.36429 20.16104 23.26785 23.51424 23.3199 23.49179 establishment of localization;protein transp 2 2 2 1.3 1.3 1.3 269.25 0 4.2643 25.93924 4 0 0 0 0 1 2 2 2 2.831567599 0.054108527 2.981428623 5.5295555 Q8BL99;H7BWZ Q8BL99;H Protein dopey -1 Dopey 1 sp|Q8BL99|DOP1_MOUSE Protein dopey -1 OS=Mus musculus OX=10090 GN=Dop1a PE=1 SV=2;tr|H7BWZ9|H7BWZ9_MOUSE Protein dopey -1 OS=Mus musculus OX=10090 GN=Dop1a PE=1 SV=1;tr|A0A087WQ48|A0A087WQ48_MOUSE Protein dopey -1 OS=Mus musculus OX=10090 GN=Dop1a PE=1 SV=1

24.26728 23.63334 24.91789 23.64042 21.01472 22.24778 21.3211 20.14887 dev elopm cell part;c Cardiac muscle cont 46 2 0 82.9 6.4 0 32.495 0 5.6822 27.36645 4 1 2 2 1 1 1 1 1 2.83069502 0.053692308 -2.931612492 -5.527392 E9Q452;Q8BSH E9Q452;Q8BSH3;E9Q454 Tpm1 tr|E9Q452|E9Q452_MOUSE Tropomy osin alpha-1 chain OS=Mus musculus OX=10090 GN=Tpm1 PE=1 SV=1;tr|Q8BSH3|Q8BSH3_MOUSE Tropomy osin alpha-1 chain OS=Mus musculus OX=10090 GN=Tpm1 PE=1 SV=1;tr|E9Q454|E9Q454_MOUSE Tropomy osin alpha-1 chain OS=Mus musculus OX=1009

21.82384 22.73167 22.51965 22.63314 20.20796 21.17346 20.89573 20.10908 localizatio cell part;cy toplasm;intracellular 1 1 1 1.4 1.4 1.4 118 0.000641 2.6774 24.42284 2 1 1 1 1 0 0 0 0 2.828524865 0.053770992 -1.830520153 -5.522015 Q3TAA7 Q3TAA7 Serine/threonine-pr Stk11ip sp|Q3TAA7|S11IP_MOUSE Serine/threonine-protein kinase 11-interacting protein OS=Mus musculus OX=10090 GN=Stk11ip PE=1 SV=1

26.6487 26.70715 26.77769 26.94503 27.17274 27.15634 27.13917 27.08608 catabolic cell part;c Purine metabolism 10 10 10 31.9 31.9 31.9 50.239 0 33.111 30.36797 43 7 7 4 4 6 10 9 8 2.827903113 0.053363636 0.368943691 5.5204745 Q8BIW1 Q8BIW1 Protein prune hom Prune sp|Q8BIW1|PRUN1_MOUSE Exopoly phosphatase PRUNE1 OS=Mus musculus OX=10090 GN=Prune1 PE=1 SV=1

22.32677 22.56023 22.51944 22.94312 20.17047 21.61696 20.65792 20.66895 carbohy dr cell part;membrane REACT_3 1 1 1 9 9 9 18.725 0.003198 1.9574 24.52987 2 1 1 1 1 0 0 0 0 2.818623648 0.053774436 -1.808812141 -5.497527 A0A0G2JDH8;D A0A0G2J UDP-N-acety lgluc Slc35a3 tr|A0A0G2JDH8|A0A0G2JDH8_MOUSE UDP-N-acety lglucosamine transporter (Fragment) OS=Mus musculus OX=10090 GN=Slc35a3 PE=1 SV=1;tr|D3YXZ7|D3Y XZ7_MOUSE UDP-N-acety lglucosamine transporter (Fragment) OS=Mus musculus OX=10090 GN=Slc35a3 PE=1 SV=1;sp|Q8R1T4|S35A3_

20.49185 19.66107 19.96988 19.60486 23.87718 22.35216 22.02982 22.06526 biological cell part;c Axon guidance 4 4 4 4.7 4.7 4.7 106.22 0 9.6748 26.09909 7 1 1 1 1 1 3 3 3 2.814267187 0.053373134 2.649188042 5.4867766 Q9DC04;F6X4A2 Q9DC04;FRegulator of G-pro Rgs3 sp|Q9DC04|RGS3_MOUSE Regulator of G-protein signaling 3 OS=Mus musculus OX=10090 GN=Rgs3 PE=1 SV=2;tr|F6X4A2|F6X4A2_MOUSE Regulator of G-protein-signaling 3 (Fragment) OS=Mus musculus OX=10090 GN=Rgs3 PE=1 SV=1;tr|Q542M0|Q542M0_MOUSE Regulator of G-protein

21.07425 18.86931 19.00991 22.65107 25.84859 25.91707 24.91237 25.38106 biological cell part;e Chemokin REACT_2 4 4 4 66.7 66.7 66.7 8.4047 0 12.468 28.33858 5 1 1 1 1 2 3 2 2 2.811284938 0.053451852 5.113639355 5.4794264 P50153 P50153 Guanine nucleotide Gng4 sp|P50153|GBG4_MOUSE Guanine nucleotide-binding protein G(I)/G(S)/G(O) subunit gamma-4 OS=Mus musculus OX=10090 GN=Gng4 PE=1 SV=1

21.22636 18.65542 20.7474 19.99605 23.89245 24.1332 23.10449 23.03263 biological cell part;intracellular membrane- 3 3 3 29.2 29.2 29.2 18.128 0 10 26.22944 4 2 1 2 1 1 1 1 1 2.783034051 0.057352941 3.384383678 5.4101439 A0A1B0GT89;Q9 A0A1B0G Inhibitor of growth Ing1 tr|A0A1B0GT89|A0A1B0GT89_MOUSE Inhibitor of growth protein 1 (Fragment) OS=Mus musculus OX=10090 GN=Ing1 PE=1 SV=1;sp|Q9QXV3|ING1_MOUSE Inhibitor of growth protein 1 OS=Mus musculus OX=10090 GN=Ing1 PE=2 SV=1

24.38251 24.62165 24.22964 25.81683 20.67764 21.9119 22.03672 19.44147 biological cell part;c Alzheimer' REACT_2 4 4 4 4.9 4.9 4.9 160.05 0 5.4661 26.80021 4 2 3 2 3 1 0 0 0 2.736836539 0.062773723 -3.745727062 -5.298184 S4R255;F8WGF S4R255;F Nitric oxide sy ntha Nos1 tr|S4R255|S4R255_MOUSE Nitric oxide sy nthase OS=Mus musculus OX=10090 GN=Nos1 PE=1 SV=1;tr|F8WGF2|F8WGF2_MOUSE Constitutiv e NOS OS=Mus musculus OX=10090 GN=Nos1 PE=1 SV=1;sp|Q9Z0J4|NOS1_MOUSE Nitric oxide sy nthase, brain OS=Mus musculus OX=10090 GN=Nos1 PE

25.30372 26.16691 25.74496 26.41232 24.53688 24.28546 24.67584 23.93382 anatomica cell part;endosome;Golgi appara 3 3 3 5.5 5.5 5.5 92.403 0.006788 1.6034 28.37397 3 2 3 3 1 1 1 2 1 2.730824654 0.063536232 -1.548979759 -5.283734 A6H6A9 A6H6A9 Rab GTPase-activ Rabgap1l sp|A6H6A9|RBG1L_MOUSE Rab GTPase-activ ating protein 1-like OS=Mus musculus OX=10090 GN=Rabgap1l PE=1 SV=1

22.96243 23.03043 23.38574 23.12807 21.95412 20.44781 21.34937 20.27094 biological cell body ; Retinol m REACT_2 3 3 3 17.3 17.3 17.3 34.826 0 3.2119 25.28577 3 1 1 1 1 0 0 1 2 2.709323987 0.065323741 -2.121110439 -5.23228 O55240;D3Z0N7 O55240;D 11-cis retinol dehy Rdh5 sp|O55240|RDH5_MOUSE Retinol dehy drogenase 5 OS=Mus musculus OX=10090 GN=Rdh5 PE=1 SV=1;tr|D3Z0N7|D3Z0N7_MOUSE Retinol dehy drogenase 5 (Fragment) OS=Mus musculus OX=10090 GN=Rdh5 PE=1 SV=1

21.26861 20.73513 19.64873 20.91442 22.3769 23.24457 22.8358 23.87145 anatomica cell part;cy tosol;intracellular me 2 2 2 6 6 6 48.343 0 3.8767 25.70972 6 0 1 0 0 1 2 2 1 2.684866409 0.068885714 2.440455914 5.1741723 P97346 P97346 Nucleoredoxin Nxn sp|P97346|NXN_MOUSE Nucleoredoxin OS=Mus musculus OX=10090 GN=Nxn PE=1 SV=1

19.94652 21.30523 18.53086 19.99499 22.79846 22.69051 23.11777 23.04203 biological cell part;nucleoplasm 1 1 1 11.5 11.5 11.5 19.312 0.000485 2.7776 25.39614 2 0 0 0 0 1 1 1 1 2.677744036 0.069446809 2.96779108 5.157334 Q921P9 Q921P9 Transcription elong Tceal1 sp|Q921P9|TCAL1_MOUSE Transcription elongation f actor A protein-like 1 OS=Mus musculus OX=10090 GN=Tceal1 PE=2 SV=1

20.53537 20.15522 21.88434 21.04237 22.98633 23.47643 23.68228 24.93544 brush border;cell cort REACT_2 28 1 1 44.5 2 2 78.776 0.001567 2.3227 26.4356 2 0 0 0 0 1 1 1 1 2.666677821 0.072140845 2.865793705 5.1312462 Q9QY B5 Q9QY B5 Gamma-adducin Add3 sp|Q9QY B5|ADDG_MOUSE Gamma-adducin OS=Mus musculus OX=10090 GN=Add3 PE=1 SV=2

20.10697 22.57741 21.94415 19.64944 25.03779 24.50936 25.17607 24.48155 2 2 2 10.1 10.1 10.1 21.137 0.005495 1.6853 27.30904 2 0 0 0 0 1 2 1 1 2.663421479 0.072391608 3.73169899 5.1235867 Q9CPR1;H3BJI4 Q9CPR1; RWD domain-cont Rwdd4;Rwsp|Q9CPR1|RWDD4_MOUSE RWD domain-containing protein 4 OS=Mus musculus OX=10090 GN=Rwdd4 PE=2 SV=1;tr|H3BJI4|H3BJI4_MOUSE RWD domain-containing 4A OS=Mus musculus OX=10090 GN=Rwdd4a PE=1 SV=1;tr|H3BJN8|H3BJN8_MOUSE RWD domain-containing 4A (Fragment) OS=Mus

22.06178 20.16715 21.30695 20.50147 23.99265 23.70028 23.36813 22.89628 anatomica apical part Basal cell REACT_2 1 1 1 2 2 2 78.86 0.001561 2.2717 25.90458 1 0 0 0 0 1 1 1 1 2.657185634 0.07225 2.480000496 5.1089407 Q60838 Q60838 Segment polarity p Dv l2 sp|Q60838|DVL2_MOUSE Segment polarity protein dishev elled homolog DVL-2 OS=Mus musculus OX=10090 GN=Dv l2 PE=1 SV=2

19.27231 20.74066 19.55768 21.08192 22.25069 22.41748 22.70946 22.35893 biological cell part;cy toplasm;intracellular 2 2 2 5.5 5.5 5.5 48.795 0.005067 1.7205 24.9725 2 0 0 1 0 1 1 1 1 2.620229456 0.078896552 2.270998955 5.0227224 Q99LG4 Q99LG4 Tetratricopeptide r Ttc5 sp|Q99LG4|TTC5_MOUSE Tetratricopeptide repeat protein 5 OS=Mus musculus OX=10090 GN=Ttc5 PE=1 SV=2

28.56874 28.6732 28.57395 28.76787 28.94843 28.92076 29.05559 28.87569 biological cell part;c Tight junct REACT_2 31 31 31 47.2 47.2 47.2 99.226 0 134.34 32.08046 174 23 22 19 20 25 26 26 27 2.613617166 0.079123288 0.304179668 5.0073998 A0A067XG53;O7 A0A067XG Peripheral plasma Cask tr|A0A067XG53|A0A067XG53_MOUSE Peripheral plasma membrane protein CASK (Fragment) OS=Mus musculus OX=10090 GN=Cask PE=1 SV=1;sp|O70589|CSKP_MOUSE Peripheral plasma membrane protein CASK OS=Mus musculus OX=10090 GN=Cask PE=1 SV=2

19.31263 21.21331 21.09294 21.64139 23.16875 23.67777 23.39008 23.55215 anatomica cell part;c Chagas di REACT_2 2 2 2 4.8 4.8 4.8 117.51 0.004643 1.7696 26.20963 2 0 1 0 1 1 1 1 1 2.611901736 0.078911565 2.632124424 5.0034298 F6QCP8;P09470 F6QCP8; Angiotensin-conv e Ace tr|F6QCP8|F6QCP8_MOUSE Angiotensin-conv erting enzy me (Fragment) OS=Mus musculus OX=10090 GN=Ace PE=1 SV=1;sp|P09470|ACE_MOUSE Angiotensin-conv erting enzy me OS=Mus musculus OX=10090 GN=Ace PE=1 SV=3

20.1382 18.95128 19.34222 21.87197 23.53077 23.09162 23.18829 23.88176 anatomica cell part Retinol m REACT_2 2 2 2 9.9 9.9 9.9 33.652 0 3.9221 25.80944 2 0 0 0 0 1 1 2 2 2.602008512 0.080540541 3.34719038 4.9805748 O88876;G5E8W O88876;G Short-chain dehy d Dhrs3 sp|O88876|DHRS3_MOUSE Short-chain dehy drogenase/reductase 3 OS=Mus musculus OX=10090 GN=Dhrs3 PE=1 SV=2;tr|G5E8W9|G5E8W9_MOUSE Short-chain dehy drogenase/reductase 3 OS=Mus musculus OX=10090 GN=Dhrs3 PE=1 SV=1;tr|B1ARS9|B1ARS9_MOUSE Short-chain dehy drogenas

27.67283 27.77643 27.77436 27.85252 28.00737 28.24509 28.0177 28.14686 establishmcell part;cy toplasm;cy tosol;end 15 15 10 49.4 49.4 36.4 54.325 0 63.853 31.17039 69 11 11 11 11 11 10 10 10 2.592648451 0.082040268 0.335219383 4.9590154 O88746;Q3UDC O88746;Q Target of My b prot Tom1 sp|O88746|TOM1_MOUSE Target of My b protein 1 OS=Mus musculus OX=10090 GN=Tom1 PE=1 SV=1;tr|Q3UDC3|Q3UDC3_MOUSE Target of My b protein 1 OS=Mus musculus OX=10090 GN=Tom1 PE=1 SV=1

23.48407 23.61986 23.3668 23.10594 21.92352 21.1014 19.42138 20.35488 behav ior;b cell part;cy toplasm 1 1 1 15.3 15.3 15.3 14.7 0 5.0106 25.4406 1 1 1 1 1 0 0 0 0 2.588459579 0.08184 -2.693872929 -4.949387 Q9D6V8 Q9D6V8 Poly adeny late-bin Paip2 sp|Q9D6V8|PAIP2_MOUSE Poly adeny late-binding protein-interacting protein 2 OS=Mus musculus OX=10090 GN=Paip2 PE=1 SV=1

23.11296 23.74026 22.75079 23.01985 19.90671 20.82913 21.68542 19.41062 biological cell part;e Cell adhesion molecu 2 2 2 12.8 12.8 12.8 23.054 0 3.4349 25.1828 3 2 1 1 1 0 0 0 0 2.586844604 0.081695364 -2.697994232 -4.945678 O54942 O54942 Claudin-5 Cldn5 sp|O54942|CLD5_MOUSE Claudin-5 OS=Mus musculus OX=10090 GN=Cldn5 PE=1 SV=2

26.64238 26.91889 26.69458 26.62617 27.03622 27.44016 27.26204 27.25052 biological cell part;c Nucleotide REACT_2 10 10 9 27.2 27.2 27.2 43.512 0 52.043 30.32002 40 5 6 5 7 8 8 8 8 2.579806633 0.084157895 0.526728153 4.9295371 P54728 P54728 UV excision repair Rad23b sp|P54728|RD23B_MOUSE UV excision repair protein RAD23 homolog B OS=Mus musculus OX=10090 GN=Rad23b PE=1 SV=2

22.65701 22.56367 22.58284 22.91351 20.31013 21.57689 19.28383 20.05654 anatomica cell part;centrosome;intracellula 1 1 1 4.3 4.3 4.3 39.316 0.005934 1.6737 25.66161 1 1 1 1 1 0 1 1 1 2.571757774 0.085986928 -2.372410774 -4.91112 A0A1W2P6E5;Q A0A1W2P POC1 centriolar pr Poc1b tr|A0A1W2P6E5|A0A1W2P6E5_MOUSE POC1 centriolar protein homolog B OS=Mus musculus OX=10090 GN=Poc1b PE=1 SV=1;tr|Q9D3W6|Q9D3W6_MOUSE POC1 centriolar protein homolog B OS=Mus musculus OX=10090 GN=Poc1b PE=1 SV=1;tr|A6H699|A6H699_MOUSE POC1 centriolar protein

20.86061 19.90746 19.63859 20.90513 21.98381 21.94135 22.04517 21.78113 cell part;cy toplasm 3 2 2 5.2 3.7 3.7 70.612 0.004505 1.7997 24.45168 3 0 0 0 0 1 2 2 1 2.557217513 0.087974026 1.609915733 4.8779645 B9EI38;A2AFI8; B9EI38;A2 RalBP1-associated Reps2 tr|B9EI38|B9EI38_MOUSE RalBP1-associated Eps domain-containing protein 2 OS=Mus musculus OX=10090 GN=Reps2 PE=1 SV=1;tr|A2AFI8|A2AFI8_MOUSE RalBP1-associated Eps domain-containing protein 2 OS=Mus musculus OX=10090 GN=Reps2 PE=1 SV=1;sp|Q80XA6|REPS2_MOUSE

21.06654 20.10677 22.05288 20.78642 25.06919 23.27611 23.78826 23.26342 cell part;nucleoplasm 2 2 2 6.5 6.5 6.5 59.241 0.007195 1.5559 26.04233 2 0 0 0 0 2 2 1 2 2.548078165 0.091716129 2.846091747 4.8571995 Q80ZU5 Q80ZU5 Coiled-coil domain Ccdc181 sp|Q80ZU5|CC181_MOUSE Coiled-coil domain-containing protein 181 OS=Mus musculus OX=10090 GN=Ccdc181 PE=1 SV=1

24.01078 24.14833 24.21897 24.03699 24.59861 24.33552 24.45432 24.43879 establishmcell part;cy toplasm;cy toskeleto 6 6 6 7.2 7.2 7.2 147.62 0 24.847 27.83533 20 3 2 3 1 2 5 6 4 2.546098137 0.091128205 0.353040695 4.8527084 E9PY J7;Q6ZPQ E9PY J7;Q Membrane-associa Pitpnm2 tr|E9PYJ7|E9PY J7_MOUSE Membrane-associated phosphatidy linositol transf er protein 2 OS=Mus musculus OX=10090 GN=Pitpnm2 PE=1 SV=1;sp|Q6ZPQ6|PITM2_MOUSE Membrane-associated phosphatidy linositol transf er protein 2 OS=Mus musculus OX=10090 GN=Pitpnm2 PE=1 SV=2

23.58555 23.51232 23.51316 23.40367 20.34479 21.56345 20.61306 22.40286 cell part;extracellular organelle; 2 2 2 12.4 12.4 12.4 35.384 0 4.222 26.64155 2 1 1 1 1 0 1 1 1 2.531596076 0.094751592 -2.272631168 -4.819897 Q9D136 Q9D136 2-oxoglutarate and Ogf od3 sp|Q9D136|OGFD3_MOUSE 2-oxoglutarate and iron-dependent oxy genase domain-containing protein 3 OS=Mus musculus OX=10090 GN=Ogf od3 PE=2 SV=1

23.02163 23.66351 24.52259 22.96397 21.6083 20.67167 21.90498 20.66647 cell part;m Tight junction;Viral m 4 3 3 1.9 1.5 1.5 221.84 0.003644 1.9226 25.93526 3 2 2 1 1 1 0 0 0 2.53021377 0.095240506 -2.330069065 -4.816777 E9Q264 E9Q264 My h15 tr|E9Q264|E9Q264_MOUSE My osin, heav y chain 15 OS=Mus musculus OX=10090 GN=My h15 PE=1 SV=1

27.04895 27.31155 27.4827 27.56449 27.77944 28.03826 28.15248 28.1053 cell motilit cell part;c Gap juncti REACT_2 32 2 2 80.4 3.6 3.6 49.953 0 6.9667 31.14625 14 2 2 2 2 2 2 2 2 2.490867049 0.102415094 0.666944504 4.7285124 Q9CWF2 Q9CWF2 Tubulin beta-2B ch Tubb2b sp|Q9CWF2|TBB2B_MOUSE Tubulin beta-2B chain OS=Mus musculus OX=10090 GN=Tubb2b PE=1 SV=1

19.40408 20.50213 20.18166 21.44801 23.53576 23.01856 22.78642 22.05732 amine me cell part;e Gly cosam REACT_3 2 2 2 4.7 4.7 4.7 82.063 0.000637 2.6029 25.34601 5 1 0 1 0 1 1 1 1 2.485762287 0.1029 2.465542793 4.7171375 P70428;F6WHC P70428;F Exostosin-2 Ext2 sp|P70428|EXT2_MOUSE Exostosin-2 OS=Mus musculus OX=10090 GN=Ext2 PE=1 SV=2;tr|F6WHC9|F6WHC9_MOUSE Exostosin-2 (Fragment) OS=Mus musculus OX=10090 GN=Ext2 PE=1 SV=1;tr|A2AIG3|A2AIG3_MOUSE Exostosin-2 (Fragment) OS=Mus musculus OX=10090 GN=Ext2 PE=1 SV=1;tr

24.6633 24.41873 27.15422 23.55391 21.64286 20.33587 19.67593 21.29771 biological cell part;intracellular 7 3 3 41.9 26.7 26.7 22.215 0 7.5424 27.94425 4 2 2 3 1 0 0 0 0 2.481576726 0.103826087 -4.20944643 -4.707824 Q8BGZ1;F2Z3Z Q8BGZ1;FHippocalcin-like pr Hpcal4 sp|Q8BGZ1|HPCL4_MOUSE Hippocalcin-like protein 4 OS=Mus musculus OX=10090 GN=Hpcal4 PE=1 SV=3;tr|F2Z3Z1|F2Z3Z1_MOUSE Hippocalcin-like protein 4 (Fragment) OS=Mus musculus OX=10090 GN=Hpcal4 PE=1 SV=1

23.60896 23.52648 24.15892 22.34649 20.53648 19.81886 21.64228 19.03615 biological cell part;intracellular REACT_2 4 4 4 12.7 12.7 12.7 77.864 0 6.2985 27.16385 4 3 3 3 1 1 1 1 1 2.472516015 0.104617284 -3.151773453 -4.687702 A0A140LHQ0;A0 A0A140LH Mediator of RNA p Med25 tr|A0A140LHQ0|A0A140LHQ0_MOUSE Mediator complex subunit 25 OS=Mus musculus OX=10090 GN=Med25 PE=1 SV=1;tr|A0A140LHG7|A0A140LHG7_MOUSE Mediator complex subunit 25 OS=Mus musculus OX=10090 GN=Med25 PE=1 SV=1;sp|Q8VCB2|MED25_MOUSE Mediator of RNA poly merase I

22.16306 22.07561 21.72925 22.85732 21.04882 20.29428 21.13142 20.67163 anatomica cell part;cell surf ace; REACT_3 4 3 3 7.5 6.4 6.4 98.19 0.004242 1.8872 25.31479 4 1 1 1 1 0 1 2 1 2.460266649 0.107705521 -1.419774055 -4.660586 Q00993;Q6PE80 Q00993;Q Ty rosine-protein ki Axl sp|Q00993|UFO_MOUSE Ty rosine-protein kinase receptor UFO OS=Mus musculus OX=10090 GN=Axl PE=1 SV=2;tr|Q6PE80|Q6PE80_MOUSE Receptor protein-ty rosine kinase OS=Mus musculus OX=10090 GN=Axl PE=1 SV=1

20.36487 20.80998 21.64421 21.62541 22.90421 22.60779 22.44187 22.62638 biosy nthe cell part;intracellular REACT_2 2 2 2 18.9 18.9 18.9 15.944 0 7.7583 25.03616 2 0 1 0 0 1 1 1 1 2.455013064 0.107341463 1.533944607 4.6489859 Q99N92 Q99N92 39S ribosomal prot Mrpl27 sp|Q99N92|RM27_MOUSE 39S ribosomal protein L27, mitochondrial OS=Mus musculus OX=10090 GN=Mrpl27 PE=1 SV=1

22.7543 22.73938 23.49607 22.99193 19.971 20.83818 21.82765 19.57914 biological cell part;cy toplasm;Golgi appar 3 3 3 5.4 5.4 5.4 103.68 0 5.1447 24.99929 4 1 1 1 2 0 0 1 0 2.436699488 0.113260606 -2.441424847 -4.608692 Q8CIE4;A0A5H1 Q8CIE4 Parp10 sp|Q8CIE4|PAR10_MOUSE Protein mono-ADP-ribosy ltransf erase PARP10 OS=Mus musculus OX=10090 GN=Parp10 PE=2 SV=1

22.89898 23.5511 22.98134 22.91386 19.63466 19.51184 22.02599 19.64104 anatomica cell part;cy toplasm;cy toskeleto 2 2 2 3.9 3.9 3.9 77.009 0.000485 2.7683 25.12605 3 2 2 1 2 0 0 0 0 2.428674351 0.115204819 -2.882936954 -4.591103 A0A5F8MPG0;Q A0A5F8M Tectonic-2 Tctn2 tr|A0A5F8MPG0|A0A5F8MPG0_MOUSE Tectonic-2 OS=Mus musculus OX=10090 GN=Tctn2 PE=1 SV=1;sp|Q2MV57|TECT2_MOUSE Tectonic-2 OS=Mus musculus OX=10090 GN=Tctn2 PE=1 SV=2

22.37417 22.24754 22.6781 21.95943 18.4706 19.50467 20.3765 20.88145 biological cell part;c Colorectal REACT_3 2 2 2 3.3 3.3 3.3 123.28 0.000637 2.6 24.4332 4 1 2 2 1 0 0 0 0 2.415357641 0.117628743 -2.506503105 -4.56201 E9QPY 6;A0A087 E9QPY 6; DNA mismatch re Msh3 tr|E9QPY 6|E9QPY 6_MOUSE DNA mismatch repair protein OS=Mus musculus OX=10090 GN=Msh3 PE=1 SV=2;tr|A0A087WQ16|A0A087WQ16_MOUSE DNA mismatch repair protein OS=Mus musculus OX=10090 GN=Msh3 PE=1 SV=1;tr|A0A087WP43|A0A087WP43_MOUSE DNA mismatch repair protein M

22.52619 22.90354 23.46151 22.19755 19.96915 21.24515 21.20283 21.17715 biological cell part;cy toplasm;cy toplasmic 2 2 2 8.4 8.4 8.4 37.829 0 3.3407 24.86717 4 1 1 1 2 0 0 0 0 2.408591696 0.119452381 -1.873628616 -4.547271 Q9ER39 Q9ER39 Torsin-1A Tor1a sp|Q9ER39|TOR1A_MOUSE Torsin-1A OS=Mus musculus OX=10090 GN=Tor1a PE=1 SV=1

26.84362 26.92207 26.92468 27.0591 27.61996 27.21697 27.28445 27.43825 autophagy cell part;c Osteoclas REACT_2 10 10 10 44.1 44.1 44.1 48.162 0 76.907 30.39039 62 9 9 7 7 8 6 9 8 2.396004454 0.122035503 0.452541828 4.5199302 Q64337;D3Y ZJ1 Q64337;D Sequestosome-1 Sqstm1 sp|Q64337|SQSTM_MOUSE Sequestosome-1 OS=Mus musculus OX=10090 GN=Sqstm1 PE=1 SV=1;tr|D3YZJ1|D3YZJ1_MOUSE Sequestosome-1 OS=Mus musculus OX=10090 GN=Sqstm1 PE=1 SV=1

24.04068 22.81644 23.56999 23.19188 21.89561 21.90341 20.70979 20.25833 cell part Ly sine de REACT_2 1 1 1 4.9 4.9 4.9 55.051 0.008739 1.4609 25.51848 1 1 1 1 1 0 0 0 0 2.377339059 0.125082353 -2.212958813 -4.479572 D6RCH2;Q6NV D6RCH2; Procollagen galact Colgalt2 tr|D6RCH2|D6RCH2_MOUSE Procollagen galactosy ltransf erase 2 OS=Mus musculus OX=10090 GN=Colgalt2 PE=1 SV=1;sp|Q6NVG7|GT252_MOUSE Procollagen galactosy ltransf erase 2 OS=Mus musculus OX=10090 GN=Colgalt2 PE=2 SV=2

24.02268 24.27696 24.4393 25.11231 22.27274 22.36798 20.9743 19.50352 biological cell part;cy toplasm;intracellular 2 2 2 6.8 6.8 6.8 61.159 0 13.941 25.92198 2 2 1 1 2 0 0 0 0 2.375658544 0.124350877 -3.183177948 -4.475949 D3Y TU2;Q3U0K D3YTU2; Proly l 3-hy droxy la Ogf od1 tr|D3YTU2|D3Y TU2_MOUSE 2-oxoglutarate and iron-dependent oxy genase domain-containing protein 1 OS=Mus musculus OX=10090 GN=Ogf od1 PE=1 SV=1;sp|Q3U0K8|OGFD1_MOUSE Proly l 3-hy droxy lase OGFOD1 OS=Mus musculus OX=10090 GN=Ogf od1 PE=1 SV=1

22.44354 22.32622 22.74544 23.43288 21.26747 20.08023 20.7283 18.95408 cell cy cle;cellular process 1 1 1 14.9 14.9 14.9 9.6308 0.007059 1.574 24.72993 1 1 1 1 1 0 0 0 0 2.367601521 0.125976744 -2.479502678 -4.458604 F2Z4B3;O35207 F2Z4B3;O Cy clin-dependent Cdk2ap1 tr|F2Z4B3|F2Z4B3_MOUSE Cy clin-dependent kinase 2-associated protein 1 OS=Mus musculus OX=10090 GN=Cdk2ap1 PE=1 SV=1;sp|O35207|CDKA1_MOUSE Cy clin-dependent kinase 2-associated protein 1 OS=Mus musculus OX=10090 GN=Cdk2ap1 PE=1 SV=2

20.23354 21.51531 19.89028 20.74542 22.38582 22.52981 23.2627 22.01662 catabolic process;cellular metabolic proces 7 7 7 5.4 5.4 5.4 176.66 0 7.6467 26.0897 8 1 2 2 0 2 5 3 1 2.360794892 0.127375723 1.952600956 4.4439827 Q8BL06 Q8BL06 Inactiv e ubiquitin c Usp54 sp|Q8BL06|UBP54_MOUSE Inactiv e ubiquitin carboxy l-terminal hy drolase 54 OS=Mus musculus OX=10090 GN=Usp54 PE=1 SV=2

20.26056 19.44755 21.09068 18.85697 21.69655 23.08698 23.16659 24.15954 biological cell part;c Adherens REACT_2 6 2 2 33.3 12 12 21.441 0.006501 1.6208 26.32944 2 0 1 1 0 1 1 1 1 2.356374546 0.126643678 3.113476753 4.4345028 Q05144;A0A2R8 Q05144;A Ras-related C3 bo Rac2 sp|Q05144|RAC2_MOUSE Ras-related C3 botulinum toxin substrate 2 OS=Mus musculus OX=10090 GN=Rac2 PE=1 SV=1;tr|A0A2R8VHH0|A0A2R8VHH0_MOUSE Ras-related C3 botulinum toxin substrate 2 (Fragment) OS=Mus musculus OX=10090 GN=Rac2 PE=1 SV=1

26.44925 26.36833 25.87319 26.42594 27.27622 26.96444 27.26428 26.79823 biological cell part;cy toplasm;e REACT_2 6 6 6 83.2 83.2 83.2 11.871 0 120.44 29.94391 37 5 4 4 5 3 5 5 4 2.353366919 0.126148571 0.79661417 4.4280596 Q9QUH0;A0A1Y Q9QUH0; Glutaredoxin-1 Glrx sp|Q9QUH0|GLRX1_MOUSE Glutaredoxin-1 OS=Mus musculus OX=10090 GN=Glrx PE=1 SV=3;tr|A0A1Y 7VM65|A0A1Y 7VM65_MOUSE Glutaredoxin-1 OS=Mus musculus OX=10090 GN=Glrx PE=1 SV=1

21.43634 21.39973 21.65158 22.45287 20.12275 19.74987 20.82582 20.36003 biological cell part;intracellular membrane- 2 2 2 4.6 4.6 4.6 98.1 0 6.2838 25.20896 4 1 1 1 1 1 0 2 1 2.345874243 0.128545455 -1.470515251 -4.412032 A0A1B0GRV3;P A0A1B0G MHC class II regu Rf x1 tr|A0A1B0GRV3|A0A1B0GRV3_MOUSE MHC class II regulatory f actor RFX1 OS=Mus musculus OX=10090 GN=Rf x1 PE=1 SV=1;sp|P48377|RFX1_MOUSE MHC class II regulatory f actor RFX1 OS=Mus musculus OX=10090 GN=Rf x1 PE=1 SV=2

29.92238 29.99159 29.9592 29.99873 30.20758 30.15424 30.14528 30.04349 biological cell part;cy toplasm;intracellular 22 22 3 59.4 59.4 9.7 51.596 0 201.97 33.31959 194 21 21 21 21 19 22 22 22 2.345732832 0.127819209 0.169671535 4.4117302 P61202 P61202 COP9 signalosom Cops2 sp|P61202|CSN2_MOUSE COP9 signalosome complex subunit 2 OS=Mus musculus OX=10090 GN=Cops2 PE=1 SV=1

28.07852 28.30008 28.23959 28.10944 28.45678 28.38286 28.4311 28.4182 biosy nthe cell part;c Fatty acid biosy nthe 11 11 11 47.7 47.7 47.7 48.627 0 79.511 31.56606 77 9 8 9 8 8 10 10 11 2.332503913 0.132359551 0.240328789 4.3835185 Q9D404 Q9D404 3-oxoacy l-[acy l-ca Oxsm sp|Q9D404|OXSM_MOUSE 3-oxoacy l-[acy l-carrier-protein] sy nthase, mitochondrial OS=Mus musculus OX=10090 GN=Oxsm PE=1 SV=1

21.23706 20.593 23.30433 20.97197 26.24538 27.97569 23.8447 26.3652 2 2 1 52.5 52.5 41 6.912 0 9.4266 29.69029 3 0 0 0 0 1 2 1 1 2.331499363 0.131977654 4.581148148 4.3813806 E9Q161 E9Q161 Alg13 tr|E9Q161|E9Q161_MOUSE N-acety lglucosaminy ldiphosphodolichol N-acety lglucosaminy ltransf erase OS=Mus musculus OX=10090 GN=Alg13 PE=1 SV=2

19.64272 21.04344 21.1756 19.20917 22.14096 22.32292 22.95883 22.82067 anatomica cell part;c Acute my REACT_2 2 2 2 5.5 5.5 5.5 60.539 0.006921 1.5835 25.27569 3 1 0 1 0 1 1 2 1 2.298068372 0.144466667 2.293115139 4.3105824 S4R1S1;Q8BTI9 S4R1S1;Q Phosphatidy linosit Pik3cb tr|S4R1S1|S4R1S1_MOUSE Phosphatidy linositol-4,5-bisphosphate 3-kinase OS=Mus musculus OX=10090 GN=Pik3cb PE=1 SV=1;sp|Q8BTI9|PK3CB_MOUSE Phosphatidy linositol 4,5-bisphosphate 3-kinase cataly tic subunit beta isof orm OS=Mus musculus OX=10090 GN=Pik3cb PE=1 S

25.17828 25.32982 25.4519 25.44919 25.70203 25.81885 26.03802 26.26617 biological cell part;cy toplasm;intracellular 2 2 2 21.2 21.2 21.2 20.29 0 12.63 28.98777 10 2 2 2 2 2 2 2 2 2.294779107 0.143955801 0.603971958 4.303653 A0A494BBA2;G3 A0A494BB Steroid receptor R Sra1 tr|A0A494BBA2|A0A494BBA2_MOUSE Steroid receptor RNA activ ator 1 (Fragment) OS=Mus musculus OX=10090 GN=Sra1 PE=1 SV=1;tr|G3X8R2|G3X8R2_MOUSE Steroid receptor RNA activ ator 1 (Fragment) OS=Mus musculus OX=10090 GN=Sra1 PE=1 SV=1;sp|Q80VJ2|SRA1_MOUSE Steroid

32.05727 32.04791 32.25901 32.16036 32.34763 32.34776 32.32507 32.3714 anatomica cell part;e Benzoate REACT_2 28 28 28 79.7 79.7 79.7 44.816 0 323.31 35.50462 401 24 24 23 24 27 27 27 26 2.288031009 0.144835165 0.216823578 4.2894573 Q8QZT1 Q8QZT1 Acety l-CoA acety l Acat1 sp|Q8QZT1|THIL_MOUSE Acety l-CoA acety ltransf erase, mitochondrial OS=Mus musculus OX=10090 GN=Acat1 PE=1 SV=1

20.3841 20.77995 20.30179 20.41691 18.95506 19.16435 20.0454 19.05309 anatomica cell part;intracellular membrane- 2 2 2 2.2 2.2 2.2 109.98 0.000164 2.9924 21.93762 2 0 0 0 0 1 1 2 2 2.286382794 0.145289617 -1.166211128 -4.285994 V9GXA5;G3UW9 V9GXA5;G Zinc f inger transcr Trps1 tr|V9GXA5|V9GXA5_MOUSE Zinc f inger transcription f actor Trps1 OS=Mus musculus OX=10090 GN=Trps1 PE=1 SV=1;tr|G3UW90|G3UW90_MOUSE Zinc f inger transcription f actor Trps1 OS=Mus musculus OX=10090 GN=Trps1 PE=1 SV=1;tr|V9GX74|V9GX74_MOUSE Zinc f inger transcrip

22.83859 23.38047 23.07589 23.25854 18.80583 21.24904 21.57472 20.01299 anatomica cell part;cell projectio REACT_2 6 6 6 5.3 5.3 5.3 197.55 0 10.919 26.28757 8 1 2 2 2 1 2 2 3 2.266089196 0.151804348 -2.727729321 -4.243486 Q6VH22;A0A0J9 Q6VH22 Intraf lagellar trans If t172 sp|Q6VH22|IF172_MOUSE Intraf lagellar transport protein 172 homolog OS=Mus musculus OX=10090 GN=If t172 PE=1 SV=1

23.50069 22.80906 25.01958 24.05895 21.59397 19.27324 21.3806 21.1789 alcohol m cell part;cy toplasm;c REACT_3 3 3 2 2.6 2.6 1.7 159.92 0 3.2154 26.26331 6 2 1 2 2 1 1 2 0 2.251377275 0.155827027 -2.990395546 -4.212822 A2ARP1;E9Q9J4 A2ARP1 Inositol hexakisph Ppip5k1 sp|A2ARP1|VIP1_MOUSE Inositol hexakisphosphate and diphosphoinositol-pentakisphosphate kinase 1 OS=Mus musculus OX=10090 GN=Ppip5k1 PE=1 SV=1

32.01963 32.02059 32.08819 32.20763 32.28273 32.27772 32.25721 32.26791 anatomica cell part;c Regulation REACT_3 13 13 13 95 95 95 14.957 0 134.05 35.46322 206 13 13 13 13 12 12 12 12 2.246736675 0.155698925 0.187382698 4.2031755 P62962;Q5SX49; P62962;Q Prof ilin-1;Prof ilin Pf n1 sp|P62962|PROF1_MOUSE Prof ilin-1 OS=Mus musculus OX=10090 GN=Pf n1 PE=1 SV=2;tr|Q5SX49|Q5SX49_MOUSE Prof ilin OS=Mus musculus OX=10090 GN=Pf n1 PE=1 SV=1

19.52673 18.87617 20.66379 19.98592 22.03379 21.03651 21.47105 21.78684 biosy nthe cell part;e Alzheimer' REACT_2 1 1 1 16.4 16.4 16.4 25.095 0 4.5946 24.14554 4 0 0 1 0 1 1 1 1 2.241278845 0.156898396 1.818896294 4.1918464 P00848 P00848 ATP sy nthase sub Mtatp6 sp|P00848|ATP6_MOUSE ATP sy nthase subunit a OS=Mus musculus OX=10090 GN=Mtatp6 PE=1 SV=1

28.28493 28.22426 28.25246 27.99189 28.76424 28.73483 28.57888 28.41768 catabolic cell part;c Nicotinate REACT_2 7 7 7 59 59 59 23.076 0 142.2 31.61824 54 6 5 5 6 5 5 5 5 2.238606709 0.157212766 0.435521603 4.1863061 Q9JM14;A2A9X5 Q9JM14;A 5(3)-deoxy ribonucl Nt5c sp|Q9JM14|NT5C_MOUSE 5(3)-deoxy ribonucleotidase, cy tosolic ty pe OS=Mus musculus OX=10090 GN=Nt5c PE=1 SV=1;tr|A2A9X5|A2A9X5_MOUSE 5(3)-deoxy ribonucleotidase, cy tosolic ty pe OS=Mus musculus OX=10090 GN=Nt5c PE=1 SV=1

23.6659 23.19161 23.60082 23.36292 19.87945 21.10474 22.1881 19.05948 biological cell part;intracellular membrane- 2 2 2 21.7 21.7 21.7 16.837 0 3.6314 26.58992 4 1 1 1 1 1 1 1 1 2.2285472 0.159174603 -2.897371292 -4.165486 A0A2R8W747;D A0A2R8W Zinc f inger protein Zf p740;Zn tr|A0A2R8W747|A0A2R8W747_MOUSE Zinc f inger protein 740 OS=Mus musculus OX=10090 GN=Zf p740 PE=1 SV=1;tr|D3Z4A3|D3Z4A3_MOUSE Zinc f inger protein 740 (Fragment) OS=Mus musculus OX=10090 GN=Zf p740 PE=1 SV=9;sp|Q6NZQ6|ZN740_MOUSE Zinc f inger protein 740 OS=Mus

24.36339 24.26471 24.53035 24.44164 24.57126 24.91242 24.92823 24.92379 biological cell part;Golgi apparatus;intrace 2 2 2 11.8 11.8 11.8 34.79 0 9.1577 28.05165 11 1 1 1 1 1 2 2 2 2.226351959 0.158610526 0.43390274 4.1609501 P52875;D3Y V67 P52875;D Transmembrane pr Tmem165 sp|P52875|TM165_MOUSE Transmembrane protein 165 OS=Mus musculus OX=10090 GN=Tmem165 PE=1 SV=2;tr|D3Y V67|D3Y V67_MOUSE GDT1 f amily protein (Fragment) OS=Mus musculus OX=10090 GN=Tmem165 PE=1 SV=1

33.69322 33.68677 33.65662 33.68708 33.73459 33.77225 33.7597 33.83479 biological cell part;c Protein pr REACT_3 59 59 59 75.3 75.3 75.3 89.321 0 323.31 36.96935 762 55 56 57 53 50 54 55 55 2.212526687 0.160774869 0.09441185 4.132448 Q01853 Q01853 Transitional endopl Vcp sp|Q01853|TERA_MOUSE Transitional endoplasmic reticulum ATPase OS=Mus musculus OX=10090 GN=Vcp PE=1 SV=4

21.38825 20.85861 20.66386 18.68436 23.66958 22.56188 22.92928 22.7978 anatomica cell part;Golgi appara REACT_3 4 4 4 7.4 7.4 7.4 81.997 0.002605 2.045 25.28302 4 0 0 0 0 3 2 1 2 2.177345795 0.171666667 2.590865612 4.060408 Q9D5R3 Q9D5R3 Centrosomal prote Cep83 sp|Q9D5R3|CEP83_MOUSE Centrosomal protein of 83 kDa OS=Mus musculus OX=10090 GN=Cep83 PE=1 SV=2

28.81559 29.15979 28.63996 29.10652 29.87439 29.83032 29.27793 29.98454 anatomica cell part;cy toplasm;cy toskeleto 32 25 12 56.3 48.4 27.6 54.565 0 124.18 32.72588 192 17 20 18 18 20 22 24 22 2.17407404 0.171875648 0.811332226 4.0537437 P11679 P11679 Keratin, ty pe II cy Krt8 sp|P11679|K2C8_MOUSE Keratin, ty pe II cy toskeletal 8 OS=Mus musculus OX=10090 GN=Krt8 PE=1 SV=4

26.5064 26.2346 26.61105 26.20732 26.77618 26.98555 26.77781 26.82542 biological cell part;c Drug meta REACT_3 10 10 10 33.9 33.9 33.9 52.292 0 53.188 29.99469 33 6 5 7 6 5 8 7 7 2.170950748 0.173257732 0.451397419 4.0473873 P13439;D6RJ62 P13439 Uridine 5-monopho Umps sp|P13439|UMPS_MOUSE Uridine 5-monophosphate sy nthase OS=Mus musculus OX=10090 GN=Umps PE=1 SV=3

20.388 20.77877 20.78659 20.20251 21.09165 22.07002 21.66071 21.41282 biological cell part;in Ribosome biogenesis 1 1 1 2 2 2 76.908 0.0076 1.5033 24.22634 1 0 0 0 0 1 1 1 1 2.165476934 0.174564103 1.019833565 4.0362603 Q8R2N2 Q8R2N2 Cirhin Cirh1a sp|Q8R2N2|UTP4_MOUSE U3 small nucleolar RNA-associated protein 4 homolog OS=Mus musculus OX=10090 GN=Utp4 PE=2 SV=3

21.27389 19.38749 20.84843 22.06364 23.68966 22.73948 23.43593 23.3009 biological cell part;cell surf ace; REACT_2 1 1 1 2.5 2.5 2.5 128.41 0 3.7427 25.6919 6 0 0 0 0 1 1 1 1 2.157976553 0.176081633 2.398127556 4.0210407 A0A286Y CQ5;O A0A286Y Nidogen-2 Nid2 tr|A0A286Y CQ5|A0A286Y CQ5_MOUSE Nidogen-2 (Fragment) OS=Mus musculus OX=10090 GN=Nid2 PE=1 SV=1;sp|O88322|NID2_MOUSE Nidogen-2 OS=Mus musculus OX=10090 GN=Nid2 PE=1 SV=2

23.26999 21.94113 22.51415 22.28198 21.1663 21.31735 19.71718 20.38867 anatomica cell part;cell projection;cilium;cy 3 3 3 4.4 4.4 4.4 96.955 0.005933 1.6714 27.58248 4 1 1 1 1 1 1 2 1 2.138399387 0.183208122 -1.854440689 -3.98146 A9Q751;G3V015 A9Q751 Cilia- and f lagella- Cf ap221 sp|A9Q751|PCDP1_MOUSE Cilia- and f lagella-associated protein 221 OS=Mus musculus OX=10090 GN=Cf ap221 PE=1 SV=1

19.51105 19.25484 22.70837 21.02434 25.66441 24.6142 24.55912 23.01637 anatomica cell part;Golgi apparatus;intrace 1 1 1 8.4 8.4 8.4 17.127 0.009596 1.4338 26.79092 1 0 0 0 0 1 1 1 1 2.135704842 0.183353535 3.838870049 3.9760289 E9PZK7;Q8BXL7 E9PZK7;Q ADP-ribosy lation f Arf rp1 tr|E9PZK7|E9PZK7_MOUSE ADP-ribosy lation f actor-related protein 1 OS=Mus musculus OX=10090 GN=Arf rp1 PE=1 SV=1;sp|Q8BXL7|ARFRP_MOUSE ADP-ribosy lation f actor-related protein 1 OS=Mus musculus OX=10090 GN=Arf rp1 PE=1 SV=2

23.34191 23.89125 23.86041 23.58497 18.51622 22.28601 19.95869 21.03288 4 4 4 49.3 49.3 49.3 16.277 0 4.1711 26.89816 5 3 1 2 3 1 1 1 1 2.13376895 0.182653266 -3.221186161 -3.972129 A2AA85;Q6NXN A2AA85;Q SUZ domain-conta Szrd1 tr|A2AA85|A2AA85_MOUSE SUZ domain-containing protein 1 (Fragment) OS=Mus musculus OX=10090 GN=Szrd1 PE=1 SV=1;sp|Q6NXN1|SZRD1_MOUSE SUZ domain-containing protein 1 OS=Mus musculus OX=10090 GN=Szrd1 PE=1 SV=1

23.56663 24.16745 25.26628 23.46526 19.39536 19.7708 21.2399 22.64475 biological cell part;e Peroxisome 5 5 5 18.4 18.4 18.4 27.003 0 5.5134 26.9257 7 3 3 3 1 1 1 1 2 2.12003693 0.18756 -3.353699684 -3.944525 E9PXK7;D6RFQ E9PXK7;D Peroxisomal mem Pex11b tr|E9PXK7|E9PXK7_MOUSE Peroxisomal membrane protein 11B OS=Mus musculus OX=10090 GN=Pex11b PE=1 SV=1;tr|D6RFQ2|D6RFQ2_MOUSE Predicted gene 42957 OS=Mus musculus OX=10090 GN=Gm42957 PE=1 SV=1;sp|Q9Z210|PX11B_MOUSE Peroxisomal membrane protein 11B OS=Mus mus

24.44979 22.54748 22.25237 23.14041 21.15927 21.43147 20.21274 19.79025 biological cell part;c Calcium signaling pa 2 2 2 14.1 14.1 14.1 34.407 0 5.7487 25.11696 3 2 2 1 2 0 0 0 0 2.115420515 0.189651741 -2.449081898 -3.935268 P56528 P56528 ADP-ribosy l cy cla Cd38 sp|P56528|CD38_MOUSE ADP-ribosy l cy clase/cy clic ADP-ribose hy drolase 1 OS=Mus musculus OX=10090 GN=Cd38 PE=1 SV=2

27.54407 27.5465 27.36369 27.88518 26.73402 27.056 26.9914 27.22242 cell part;intracellular REACT_2 5 5 5 26.3 26.3 26.3 32.118 0 23 30.52561 18 3 3 3 3 4 4 4 4 2.109951885 0.191069307 -0.583902359 -3.924316 Q5SUD5;Q5SUCQ5SUD5; Protein SCO1 hom Sco1 tr|Q5SUD5|Q5SUD5_MOUSE Protein SCO1 homolog, mitochondrial OS=Mus musculus OX=10090 GN=Sco1 PE=1 SV=1;sp|Q5SUC9|SCO1_MOUSE Protein SCO1 homolog, mitochondrial OS=Mus musculus OX=10090 GN=Sco1 PE=1 SV=1

19.11296 21.37884 20.53806 20.07166 22.04677 22.02476 22.17048 22.37281 biosy nthe cell part;c Cy tosolic REACT_2 2 2 2 21.6 21.6 21.6 22.948 0 7.4256 25.12479 4 0 0 1 0 1 1 1 1 2.107612705 0.191014778 1.878323078 3.9196367 Q9D2C6;A0A2R Q9D2C6; DNA-directed RNA Polr3h sp|Q9D2C6|RPC8_MOUSE DNA-directed RNA poly merase III subunit RPC8 OS=Mus musculus OX=10090 GN=Polr3h PE=1 SV=2;tr|A0A2R8VK29|A0A2R8VK29_MOUSE DNA-directed RNA poly merase III subunit RPC8 OS=Mus musculus OX=10090 GN=Polr3h PE=1 SV=1

23.51027 23.66384 22.81605 22.57062 21.05892 22.3602 20.38872 21.10792 anatomica cell part;cell projectio REACT_3 3 3 3 10.2 10.2 10.2 47.952 0 5.7197 25.42085 3 2 1 1 1 1 1 0 0 2.101877082 0.191843137 -1.911253452 -3.908175 Q8R3P7;D3Z1F Q8R3P7;DClusterin-associat Cluap1 sp|Q8R3P7|CLUA1_MOUSE Clusterin-associated protein 1 OS=Mus musculus OX=10090 GN=Cluap1 PE=1 SV=1;tr|D3Z1F2|D3Z1F2_MOUSE Clusterin-associated protein 1 (Fragment) OS=Mus musculus OX=10090 GN=Cluap1 PE=1 SV=9;tr|E9Q8M4|E9Q8M4_MOUSE Clusterin-associated prot

22.69398 22.83478 25.34895 22.95312 21.74056 20.28467 20.11638 19.86242 biological cell part;cell projection;centroso 5 5 5 25.8 25.8 25.8 28.378 0 4.9075 26.88332 5 1 1 3 2 2 2 1 1 2.091476658 0.194926829 -2.956697464 -3.887435 Q8QZT2 Q8QZT2 Centriole, cilia and Ccsap sp|Q8QZT2|CCSAP_MOUSE Centriole, cilia and spindle-associated protein OS=Mus musculus OX=10090 GN=Ccsap PE=1 SV=1

26.36601 26.34597 26.57542 26.39761 26.6088 27.31147 27.242 27.35548 autophagy cell part;c Regulation of autoph 6 6 5 56.4 56.4 50.4 13.667 0 7.6017 30.21037 27 4 4 4 3 5 6 6 5 2.084700018 0.196718447 0.708180904 3.8739528 P60521 P60521 Gamma-aminobuty Gabarapl2 sp|P60521|GBRL2_MOUSE Gamma-aminobuty ric acid receptor-associated protein-like 2 OS=Mus musculus OX=10090 GN=Gabarapl2 PE=1 SV=1

24.99309 24.85723 25.09648 24.88054 25.30205 25.89301 25.64491 25.32701 catabolic process;ce Inositol ph REACT_2 7 7 7 15.1 15.1 15.1 99.971 0 20.087 28.78251 27 3 3 3 3 2 6 5 5 2.082592105 0.196850242 0.584907532 3.8697639 Q3UEQ1;E9Q9A Q3UEQ1; Ty pe I inositol 3,4- Inpp4a tr|Q3UEQ1|Q3UEQ1_MOUSE Phosphatidy linositol-3,4-bisphosphate 4-phosphatase OS=Mus musculus OX=10090 GN=Inpp4a PE=1 SV=1;tr|E9Q9A0|E9Q9A0_MOUSE Phosphatidy linositol-3,4-bisphosphate 4-phosphatase OS=Mus musculus OX=10090 GN=Inpp4a PE=1 SV=2;tr|F6V2U0|F6V2U0

27.57015 27.96185 27.57304 27.40422 28.08096 28.06384 28.10099 28.09482 cell cy cle; cell part;cy toplasmic membrane 17 9 9 60.8 43.5 43.5 49.619 0 72.192 31.16273 69 7 7 6 6 9 8 8 9 2.078288588 0.198615385 0.457836628 3.861219 Q9R1T4;A2A3W Q9R1T4 Septin-6 SEPTIN6 sp|Q9R1T4|SEPT6_MOUSE Septin-6 OS=Mus musculus OX=10090 GN=Septin6 PE=1 SV=4

20.84257 20.05609 19.36762 20.31221 21.15128 21.95088 22.00103 21.28811 1 1 1 3.4 3.4 3.4 66.798 0 4.6631 24.23462 3 0 0 0 0 1 1 1 1 2.072818928 0.199827751 1.453201771 3.8503725 Q8C318;A2RSJ4 Q8C318;A UHRF1-binding pr Uhrf 1bp1l tr|Q8C318|Q8C318_MOUSE UHRF1-binding protein 1-like (Fragment) OS=Mus musculus OX=10090 GN=Uhrf 1bp1l PE=1 SV=1;sp|A2RSJ4|UH1BL_MOUSE UHRF1-binding protein 1-like OS=Mus musculus OX=10090 GN=Uhrf 1bp1l PE=1 SV=2

21.78536 21.4291 22.57607 22.07503 20.87997 20.96432 21.10156 20.29664 biological cell part;c Gly cerolip REACT_3 2 2 2 3.3 3.3 3.3 135.21 0 3.7076 24.09362 4 1 2 1 1 0 0 1 0 2.071938906 0.199466667 -1.155769348 -3.848629 E9PUQ8 E9PUQ8 Diacy lgly cerol kin Dgkd sp|E9PUQ8|DGKD_MOUSE Diacy lgly cerol kinase delta OS=Mus musculus OX=10090 GN=Dgkd PE=1 SV=1

30.49677 30.6806 30.76659 30.66704 30.88344 30.88016 30.83598 30.91371 biological cell part;e Carbon f ix REACT_3 34 34 34 55.8 55.8 55.8 79.921 0 219.67 34.02622 266 27 28 26 28 27 28 29 28 2.069034719 0.19943128 0.225569725 3.8428774 Q91ZA3;H3BL62 Q91ZA3 Propiony l-CoA car Pcca sp|Q91ZA3|PCCA_MOUSE Propiony l-CoA carboxy lase alpha chain, mitochondrial OS=Mus musculus OX=10090 GN=Pcca PE=1 SV=2

20.75918 19.60962 21.96017 20.99217 23.28785 22.71708 22.96012 22.36188 cell part REACT_2 1 1 1 9.1 9.1 9.1 23.528 0 3.6824 25.30556 3 0 0 0 0 1 1 1 1 2.068641754 0.198698113 2.001448631 3.8420995 Q9CY 24 Q9CY 24 Transmembrane pr Tmem179 sp|Q9CY 24|T179B_MOUSE Transmembrane protein 179B OS=Mus musculus OX=10090 GN=Tmem179b PE=1 SV=1

20.25522 22.12033 19.16615 21.31925 23.17711 23.16782 24.52199 23.13411 biological cell part;cy toplasm;i REACT_2 2 2 2 16.7 16.7 16.7 26.529 0 12.586 26.10653 2 0 0 0 0 2 1 2 2 2.062796278 0.200037559 2.785019875 3.8305376 Q9CR11 Q9CR11 YEATS domain-co Y eats4 sp|Q9CR11|Y ETS4_MOUSE YEATS domain-containing protein 4 OS=Mus musculus OX=10090 GN=Y eats4 PE=2 SV=1

21.184 19.51077 20.6726 26.25519 28.33471 27.6013 27.09114 27.70418 cellular lip cell part;c Inositol ph REACT_2 5 4 4 5.5 5 5 154.71 0.000165 3.0759 30.63019 8 1 1 1 1 2 3 3 1 2.056378246 0.20271028 5.777192593 3.8178636 D3YZB2;F8WH D3YZB2;FSy naptojanin-2 Sy nj2 tr|D3YZB2|D3Y ZB2_MOUSE Phosphoinositide 5-phosphatase OS=Mus musculus OX=10090 GN=Sy nj2 PE=1 SV=1;tr|F8WHD8|F8WHD8_MOUSE Phosphoinositide 5-phosphatase OS=Mus musculus OX=10090 GN=Sy nj2 PE=1 SV=1;sp|Q9D2G5|SY NJ2_MOUSE Sy naptojanin-2 OS=Mus musculus OX=1009

23.75379 22.79013 24.27916 24.04009 19.12133 19.92273 21.46889 22.29077 amine me cell part;cy toplasm;extracellular 2 2 2 25.8 25.8 25.8 20.489 0 23.77 25.8087 6 2 1 1 1 0 0 0 0 2.05431973 0.202288372 -3.014863014 -3.813803 O88593 O88593 Peptidogly can rec Pgly rp1 sp|O88593|PGRP1_MOUSE Peptidogly can recognition protein 1 OS=Mus musculus OX=10090 GN=Pgly rp1 PE=1 SV=1

23.88064 23.37146 23.70683 23.10034 18.6945 21.86107 21.72483 20.12407 catabolic cell part;e Parkinson's disease; 2 2 2 16.8 16.8 16.8 25.848 0 4.1202 25.64955 3 1 2 2 1 0 0 0 0 2.044460331 0.206425926 -2.913696289 -3.794385 B0QZM1;B1ASK B0QZM1; Ubiquitin-conjugati Ube2j2 tr|B0QZM1|B0QZM1_MOUSE Ubiquitin-conjugating enzy me E2 J2 OS=Mus musculus OX=10090 GN=Ube2j2 PE=1 SV=1;tr|B1ASK8|B1ASK8_MOUSE Ubiquitin-conjugating enzy me E2 J2 OS=Mus musculus OX=10090 GN=Ube2j2 PE=1 SV=1;sp|Q6P073|UB2J2_MOUSE Ubiquitin-conjugating enzy me

24.94454 25.22762 24.99123 24.76086 24.4899 24.60196 24.69503 24.42944 biological cell part;c Insulin sig REACT_2 3 3 3 42.2 42.2 42.2 14.703 0 8.9529 28.15258 13 3 2 3 2 2 3 2 3 2.040799751 0.20875576 -0.426981926 -3.787188 M0QWD1;D3Z73 M0QWD1; Eukary otic transla Eif 4e2 tr|M0QWD1|M0QWD1_MOUSE Eukary otic translation initiation f actor 4E ty pe 2 (Fragment) OS=Mus musculus OX=10090 GN=Eif 4e2 PE=1 SV=1;tr|D3Z730|D3Z730_MOUSE Eukary otic translation initiation f actor 4E ty pe 2 OS=Mus musculus OX=10090 GN=Eif 4e2 PE=1 SV=1;tr|Q0P6

27.24472 27.6102 27.20834 27.5786 27.09154 26.05681 26.15493 26.46256 cell part;cell projection;cy toplas 2 2 2 4.9 4.9 4.9 81.332 0.007605 1.5089 30.3742 6 1 1 1 1 1 2 2 2 2.035916089 0.210678899 -0.969006538 -3.777596 Q9DBQ7 Q9DBQ7 Protein-associating Scy l3 sp|Q9DBQ7|PACE1_MOUSE Protein-associating with the carboxy l-terminal domain of ezrin OS=Mus musculus OX=10090 GN=Scy l3 PE=1 SV=3

28.98533 28.9888 28.8079 28.95191 28.67815 28.72745 28.79634 28.77621 biological cell part;cy toplasm;extracellular 8 8 3 53.1 53.1 26.9 16.367 0 45.322 31.94084 83 8 7 7 7 7 7 7 8 2.02879326 0.214063927 -0.188949108 -3.763629 Q9D2M8;A6X925 Q9D2M8; Ubiquitin-conjugati Ube2v 2;U sp|Q9D2M8|UB2V2_MOUSE Ubiquitin-conjugating enzy me E2 v ariant 2 OS=Mus musculus OX=10090 GN=Ube2v 2 PE=1 SV=4;tr|A6X925|A6X925_MOUSE Ubiquitin-conjugating enzy me E2 v ariant 2 OS=Mus musculus OX=10090 GN=Ube2v 2 PE=1 SV=1;tr|B7ZBY 6|B7ZBY 6_MOUSE Ubiquitin-conj

28.64578 28.87622 28.75232 28.91271 29.09718 29.16796 28.98818 29.02316 biological cell part;e Pathway s REACT_2 8 8 8 81.4 81.4 81.4 13.17 0 13.42 32.21011 84 7 7 7 7 7 8 8 7 2.01508257 0.221072727 0.272363186 3.736816 P62869;A0A3B2 P62869;A Transcription elong Tceb2 sp|P62869|ELOB_MOUSE Elongin-B OS=Mus musculus OX=10090 GN=Elob PE=1 SV=1;tr|A0A3B2WBM3|A0A3B2WBM3_MOUSE Elongin-B OS=Mus musculus OX=10090 GN=Elob PE=1 SV=1

34.73393 34.72736 34.56041 34.75658 34.86701 34.89681 34.83262 34.88667 anatomica cell part;c Antigen pr REACT_2 64 64 36 79.6 79.6 48.8 83.28 0 323.31 38.06803 991 60 58 60 59 62 60 59 61 2.008492156 0.223746606 0.17620945 3.7239609 P11499;E9Q3D6 P11499 Heat shock protein Hsp90ab1 sp|P11499|HS90B_MOUSE Heat shock protein HSP 90-beta OS=Mus musculus OX=10090 GN=Hsp90ab1 PE=1 SV=3

20.29896 20.53107 21.36397 20.31501 19.39452 18.61465 19.73332 19.54231 biosy nthe cell part;m Gly cosy lp REACT_3 1 1 1 4.4 4.4 4.4 25.679 0.004506 1.8018 21.88815 3 0 0 0 0 1 1 1 1 2.005531051 0.225945946 -1.306052685 -3.718192 F7BU94;A0A140 F7BU94;A Phosphatidy linosit Pigq tr|F7BU94|F7BU94_MOUSE Phosphatidy linositol N-acety lglucosaminy ltransf erase subunit Q (Fragment) OS=Mus musculus OX=10090 GN=Pigq PE=1 SV=2;tr|A0A140LIE2|A0A140LIE2_MOUSE Phosphatidy linositol N-acety lglucosaminy ltransf erase subunit Q OS=Mus musculus OX=100

24.6212 24.78062 24.71476 24.72546 25.25879 25.11625 25.37368 24.84824 biological cell part;c Gly cerolip REACT_3 6 6 6 9.2 9.2 9.2 130.1 0 11.953 28.39881 11 1 1 2 1 2 3 4 5 2.004804915 0.22561435 0.438726425 3.716778 A0A2I3BQ48;A0 A0A2I3BQ Diacy lgly cerol kin Dgkh tr|A0A2I3BQ48|A0A2I3BQ48_MOUSE Diacy lgly cerol kinase OS=Mus musculus OX=10090 GN=Dgkh PE=1 SV=1;tr|A0A2I3BQ43|A0A2I3BQ43_MOUSE Diacy lgly cerol kinase OS=Mus musculus OX=10090 GN=Dgkh PE=1 SV=1;tr|A0JP53|A0JP53_MOUSE Diacy lgly cerol kinase OS=Mus musculus OX=

24.05001 21.22601 20.64447 20.65352 24.86806 24.60987 24.70483 24.43078 3 3 3 4.8 4.8 4.8 109.95 0.008308 1.4698 27.52731 4 1 0 0 0 2 3 3 2 1.983942873 0.238982143 3.009881973 3.6762643 Q3U0J8 Q3U0J8 TBC1 domain f am Tbc1d2b sp|Q3U0J8|TBD2B_MOUSE TBC1 domain f amily member 2B OS=Mus musculus OX=10090 GN=Tbc1d2b PE=1 SV=2

23.80902 23.72619 26.32605 23.18394 19.88866 19.6552 22.1312 21.62528 cell part;in Gly cosam REACT_2 3 3 3 11.8 11.8 11.8 62.185 0 10.419 27.49503 5 1 2 2 1 0 0 1 1 1.981907583 0.239093333 -3.436214447 -3.672323 Q08890;F6X9C5 Q08890;F Iduronate 2-sulf ata Ids sp|Q08890|IDS_MOUSE Iduronate 2-sulf atase OS=Mus musculus OX=10090 GN=Ids PE=2 SV=3;tr|F6X9C5|F6X9C5_MOUSE Iduronate 2-sulf atase (Fragment) OS=Mus musculus OX=10090 GN=Ids PE=1 SV=8

27.2978 27.13193 27.36469 27.14783 27.07867 26.59349 26.65444 26.8169 anatomica cell part;cy toplasm;cy toplasmic 2 2 2 36.6 36.6 36.6 12.605 0 8.7342 30.27398 17 2 2 2 2 1 2 1 1 1.981758517 0.238336283 -0.449687958 -3.672035 Q9D8T7;F8WHU Q9D8T7;F SRA stem-loop-int Slirp sp|Q9D8T7|SLIRP_MOUSE SRA stem-loop-interacting RNA-binding protein, mitochondrial OS=Mus musculus OX=10090 GN=Slirp PE=1 SV=2;tr|F8WHU8|F8WHU8_MOUSE SRA stem-loop-interacting RNA-binding protein, mitochondrial (Fragment) OS=Mus musculus OX=10090 GN=Slirp

24.05001 20.62571 19.51472 19.92542 24.82934 24.45782 25.07055 25.0426 anatomica cell part;in Axon guid REACT_2 6 6 6 4.3 4.3 4.3 211.53 0 8.8608 27.7377 5 2 0 1 2 3 3 4 3 1.978415788 0.240123348 3.82111454 3.6655665 P70207;Q80UG2 P70207 Plexin-A2 Plxna2 sp|P70207|PLXA2_MOUSE Plexin-A2 OS=Mus musculus OX=10090 GN=Plxna2 PE=1 SV=2

21.11913 20.38406 20.18854 20.82582 21.41787 21.65508 21.71243 21.22835 biological cell part;m Basal tran REACT_2 1 1 1 1.9 1.9 1.9 89.125 0.0072 1.5652 24.02884 3 0 0 0 0 1 1 1 1 1.977931136 0.239807018 0.874043465 3.6646291 P49135 P49135 TFIIH basal transc Ercc3 sp|P49135|ERCC3_MOUSE General transcription and DNA repair f actor IIH helicase subunit XPB OS=Mus musculus OX=10090 GN=Ercc3 PE=2 SV=1

27.45057 27.31657 27.25467 27.4753 27.6526 27.68264 27.5974 27.51299 biological cell part;cy toplasm;i REACT_3 11 11 11 50.8 50.8 50.8 43.221 0 99.763 30.70775 77 9 8 7 10 9 9 10 7 1.976637232 0.239406114 0.237133026 3.6621272 Q8BP48;A0A0G2 Q8BP48 Methionine aminop Metap1 sp|Q8BP48|MAP11_MOUSE Methionine aminopeptidase 1 OS=Mus musculus OX=10090 GN=Metap1 PE=1 SV=1

20.12695 21.20509 20.18066 22.15529 22.80849 22.88291 22.4437 22.71354 biological cell part;cy toplasm;intracellular 2 2 2 3.8 3.8 3.8 104.48 0 5.6272 25.35468 2 1 0 0 0 1 1 1 1 1.973444375 0.241165217 1.795162678 3.6559568 A0A1Y 7VJH3;Q A0A1Y 7V Apoptosis-stimulat Ppp1r13b tr|A0A1Y 7VJH3|A0A1Y 7VJH3_MOUSE Apoptosis-stimulating of p53 protein 1 (Fragment) OS=Mus musculus OX=10090 GN=Ppp1r13b PE=1 SV=1;sp|Q62415|ASPP1_MOUSE Apoptosis-stimulating of p53 protein 1 OS=Mus musculus OX=10090 GN=Ppp1r13b PE=1 SV=2

20.5834 20.07523 20.15775 19.65017 20.90345 21.04707 21.89401 20.98801 biological cell part;intracellular;sy napse 3 3 3 4.9 4.9 4.9 53.837 0 5.4205 23.92922 4 0 0 0 0 2 3 2 1 1.967365767 0.244727273 1.091499329 3.6442232 Q3V2R3 Q3V2R3 Chn2 tr|Q3V2R3|Q3V2R3_MOUSE Chimaerin OS=Mus musculus OX=10090 GN=Chn2 PE=1 SV=1

22.77742 23.03475 22.48525 23.55391 19.30008 19.13947 21.70117 21.37242 biological cell part;cy toplasm 5 5 1 8 8 1.4 70.273 0 3.1528 25.89546 7 2 2 1 2 2 5 2 1 1.964766108 0.244086207 -2.584547043 -3.63921 E9Q1U6 E9Q1U6 Ctif tr|E9Q1U6|E9Q1U6_MOUSE CBP80/20-dependent translation initiation f actor OS=Mus musculus OX=10090 GN=Ctif PE=1 SV=1

23.44481 23.25025 23.1325 23.39257 24.42892 23.54498 24.41493 24.02859 biological cell part REACT_3 2 2 2 4.2 4.2 4.2 73.649 0 3.4104 27.1032 13 2 2 2 2 1 2 2 2 1.962331451 0.244085837 0.799326897 3.6345189 E9QJY 0;P18581 E9QJY 0;P Cationic amino aci Slc7a2 tr|E9QJY 0|E9QJY 0_MOUSE Cationic amino acid transporter 2 OS=Mus musculus OX=10090 GN=Slc7a2 PE=1 SV=1;sp|P18581|CTR2_MOUSE Cationic amino acid transporter 2 OS=Mus musculus OX=10090 GN=Slc7a2 PE=1 SV=3

25.19449 25.86985 26.16173 25.89668 27.04906 26.98902 26.90231 26.23564 cellular pr cell part;cy toplasm;cy toskeleto 4 4 4 5.5 5.5 5.5 115.45 0 6.0079 29.35111 8 3 2 3 1 2 2 2 2 1.958262835 0.245965812 1.013315678 3.6266849 H3BIZ7;Q3TB04 H3BIZ7;Q FERM domain-con Frmd4a tr|H3BIZ7|H3BIZ7_MOUSE FERM domain-containing protein 4A OS=Mus musculus OX=10090 GN=Frmd4a PE=1 SV=1;tr|Q3TB04|Q3TB04_MOUSE FERM domain-containing protein 4A OS=Mus musculus OX=10090 GN=Frmd4a PE=1 SV=1;sp|Q8BIE6|FRM4A_MOUSE FERM domain-containing protein

32.23925 32.25414 32.30756 32.41815 32.64229 32.59531 32.59257 32.39077 anatomica cell part;cy toplasm;c REACT_2 29 29 28 68.6 68.6 66.9 51.564 0 323.31 35.69675 385 27 27 24 28 26 26 26 25 1.95637018 0.245480851 0.250455856 3.6230434 P40124;B1ARS0 P40124 Adeny ly l cy clase- Cap1 sp|P40124|CAP1_MOUSE Adeny ly l cy clase-associated protein 1 OS=Mus musculus OX=10090 GN=Cap1 PE=1 SV=4

23.06766 22.95759 22.13166 26.4821 20.24778 20.058 19.96436 20.3586 3 3 3 1.8 1.8 1.8 211.54 0.009593 1.4297 26.34009 3 1 2 1 1 0 0 1 1 1.954284438 0.245627119 -3.502569675 -3.619032 E9Q5M6 E9Q5M6 Cf ap44 sp|E9Q5M6|CFA44_MOUSE Cilia- and f lagella-associated protein 44 OS=Mus musculus OX=10090 GN=Cf ap44 PE=2 SV=1

22.84272 20.36963 21.29665 20.76986 23.80705 23.00508 23.12888 23.68271 anatomica cell part;cy toplasm;c REACT_3 2 2 2 8.4 8.4 8.4 47.976 0 4.6045 26.01984 5 1 0 0 0 1 2 2 2 1.951362394 0.245907173 2.086217403 3.6134165 E0CZ81;Q8CIV8 E0CZ81;Q Tubulin-specif ic ch Tbce tr|E0CZ81|E0CZ81_MOUSE Tubulin-f olding cof actor E OS=Mus musculus OX=10090 GN=Tbce PE=1 SV=1;sp|Q8CIV8|TBCE_MOUSE Tubulin-specif ic chaperone E OS=Mus musculus OX=10090 GN=Tbce PE=1 SV=1

22.96782 23.13951 23.07028 23.17681 22.55913 21.10336 21.01531 19.95797 biosy nthe cell part;m Folate bio REACT_2 3 3 3 9.7 9.7 9.7 69.858 0 10.716 25.64232 5 3 2 3 1 1 1 1 0 1.943262654 0.248941176 -1.929663181 -3.597871 Q5RKZ7;G3UYX Q5RKZ7 Moly bdenum cof a Mocs1 sp|Q5RKZ7|MOCS1_MOUSE Moly bdenum cof actor biosy nthesis protein 1 OS=Mus musculus OX=10090 GN=Mocs1 PE=1 SV=2

24.27419 24.89157 24.47017 24.39015 21.57344 21.0838 23.90003 20.37237 catabolic cell part;in DNA replication 4 4 4 50 50 50 17.883 0 9.4324 27.4847 9 3 2 2 3 0 1 1 0 1.933169506 0.255280335 -2.77410984 -3.578543 A0A494BBB6;A0 A0A494BB Ribonuclease H2 s Rnaseh2c tr|A0A494BBB6|A0A494BBB6_MOUSE Ribonuclease H2 subunit C (Fragment) OS=Mus musculus OX=10090 GN=Rnaseh2c PE=1 SV=1;tr|A0A494B9C7|A0A494B9C7_MOUSE Ribonuclease H2 subunit C (Fragment) OS=Mus musculus OX=10090 GN=Rnaseh2c PE=1 SV=1;sp|Q9CQ18|RNH2C_MOUSE Ribo

21.57493 21.98629 19.47392 20.67959 23.92597 22.72422 22.96261 22.9344 1 1 1 4.7 4.7 4.7 37.574 0.001571 2.3533 25.53169 1 0 0 0 0 1 1 1 1 1.931133557 0.255366667 2.208114624 3.5746497 Q7TSF4 Q7TSF4 Leucine-rich repea Lrrc75a sp|Q7TSF4|LR75A_MOUSE Leucine-rich repeat-containing protein 75A OS=Mus musculus OX=10090 GN=Lrrc75a PE=1 SV=1

21.26259 21.78365 22.93043 22.9767 20.18748 20.70833 20.99049 20.54698 cell part;Golgi apparatus;intrace 3 3 3 7.1 7.1 7.1 62.055 0.000164 2.9771 25.74312 5 2 1 1 1 2 1 1 1 1.921914509 0.259419087 -1.630019188 -3.557046 Q8BUV8 Q8BUV8 Protein GPR107 Gpr107 sp|Q8BUV8|GP107_MOUSE Protein GPR107 OS=Mus musculus OX=10090 GN=Gpr107 PE=1 SV=2

25.69554 24.90118 24.82953 24.89717 24.34509 23.67023 24.29299 24.30208 amine me cell part;intracellular membrane- 1 1 1 16.8 16.8 16.8 16.667 0 29.75 27.86378 8 1 1 1 1 1 1 1 1 1.920103863 0.25953719 -0.928257465 -3.553593 Q8CBY 0 Q8CBY 0 Glutamy l-tRNA(Gl Gatc sp|Q8CBY 0|GATC_MOUSE Glutamy l-tRNA(Gln) amidotransf erase subunit C, mitochondrial OS=Mus musculus OX=10090 GN=Gatc PE=1 SV=1

27.07561 27.15199 26.99237 26.93225 27.24 27.19178 27.44008 27.30591 cellular pr cell part;c Toxoplasmosis 12 12 10 44 44 35.2 48.493 0 78.82 30.36223 46 7 8 7 8 6 7 7 7 1.91927542 0.259407407 0.256385326 3.5520138 Q9DCE9 Q9DCE9 Igtp tr|Q9DCE9|Q9DCE9_MOUSE Interf eron gamma-induced GTPase OS=Mus musculus OX=10090 GN=Igtp PE=1 SV=1

24.24038 25.08768 24.48677 24.42496 25.35595 25.42021 25.31648 25.02559 cellular m cell part;cy toplasm 10 10 10 21.8 21.8 21.8 67.969 0 80.027 28.37642 26 3 6 3 2 3 7 6 4 1.913321466 0.262852459 0.719611645 3.540673 O35239;A0A1L1 O35239 Ty rosine-protein p Ptpn9 sp|O35239|PTN9_MOUSE Ty rosine-protein phosphatase non-receptor ty pe 9 OS=Mus musculus OX=10090 GN=Ptpn9 PE=1 SV=2

28.43126 28.7197 28.34769 28.63989 28.85234 29.06733 28.95676 28.77806 anatomica cell part;c Chemokin REACT_3 20 20 20 62 62 62 50.716 0 78.603 32.04928 121 16 17 17 14 14 18 17 20 1.905943903 0.266612245 0.378988743 3.5266432 P41241;A0A1L1 P41241 Ty rosine-protein ki Csk sp|P41241|CSK_MOUSE Ty rosine-protein kinase CSK OS=Mus musculus OX=10090 GN=Csk PE=1 SV=2

26.19865 26.37577 25.74229 26.2661 26.63699 26.57658 26.6979 26.64113 cell death; cell part;cy toplasm;c REACT_3 20 13 13 29.9 23.6 23.6 83.886 0 48.458 29.76512 29 6 7 4 4 5 10 10 10 1.881131625 0.284276423 0.492445469 3.4796405 Q8BHL5;F6Y XR Q8BHL5 Engulf ment and ce Elmo2 sp|Q8BHL5|ELMO2_MOUSE Engulf ment and cell motility protein 2 OS=Mus musculus OX=10090 GN=Elmo2 PE=1 SV=1

22.03761 22.0413 23.13597 21.77872 20.03193 18.75956 20.71704 21.14767 4 4 4 5.6 5.6 5.6 137.32 0.004639 1.757 26.37146 3 2 2 1 1 1 2 2 0 1.869829251 0.293117409 -2.084352493 -3.458322 Q9CXK9;D3Z5I9 Q9CXK9;DRNA-binding prote Rbm33 sp|Q9CXK9|RBM33_MOUSE RNA-binding protein 33 OS=Mus musculus OX=10090 GN=Rbm33 PE=1 SV=2;tr|D3Z5I9|D3Z5I9_MOUSE RNA-binding protein 33 OS=Mus musculus OX=10090 GN=Rbm33 PE=1 SV=1;tr|F6RNF9|F6RNF9_MOUSE RNA-binding protein 33 (Fragment) OS=Mus musculus OX=1

20.73051 19.45271 21.98203 19.87015 22.61856 21.96169 22.88084 22.826 anatomica cell part;membrane 3 3 3 16.9 16.9 16.9 39.968 0 8.0511 25.74684 5 1 1 2 0 1 1 1 1 1.86824049 0.293693548 2.062923908 3.45533 Q3TUA9 Q3TUA9 Protein O-mannos Pomk sp|Q3TUA9|SG196_MOUSE Protein O-mannose kinase OS=Mus musculus OX=10090 GN=Pomk PE=1 SV=2

28.78506 29.00996 28.80391 28.92895 29.02704 29.09695 29.10011 29.07584 biological cell part;cell projection;cy toplas 17 15 15 52.9 48.9 48.9 40.079 0 98.692 32.17102 78 11 12 12 13 11 10 10 10 1.867262261 0.293301205 0.193019867 3.4534883 Q91Y R1;D3Z2H Q91Y R1 Twinf ilin-1 Twf 1 sp|Q91Y R1|TWF1_MOUSE Twinf ilin-1 OS=Mus musculus OX=10090 GN=Twf 1 PE=1 SV=2

21.00343 19.24804 19.8352 22.07083 18.45411 18.36995 18.39435 18.25382 anatomica cell part;c Basal cell REACT_2 1 1 1 2.9 2.9 2.9 65.85 0.008594 1.4613 20.92622 2 0 0 0 0 1 1 1 1 1.866951017 0.292384 -2.171318054 -3.452902 Q3UMJ8;Q0VGT Q3UMJ8; Zinc f inger protein Gli3;Gli2 tr|Q3UMJ8|Q3UMJ8_MOUSE Transcriptional activ ator GLI3 (Fragment) OS=Mus musculus OX=10090 GN=Gli3 PE=1 SV=1;sp|Q0VGT2|GLI2_MOUSE Zinc f inger protein GLI2 OS=Mus musculus OX=10090 GN=Gli2 PE=1 SV=2;sp|Q61602|GLI3_MOUSE Transcriptional activ ator GLI3 OS=Mus

22.81902 20.55048 20.06985 20.38921 23.40263 23.21827 22.75896 23.35917 anatomica cell part;cell projectio REACT_3 2 2 2 3.5 3.5 3.5 79.931 0 4.5126 25.79082 8 1 0 0 0 2 2 2 2 1.86556339 0.291681275 2.227619648 3.4502908 Q9CWF6 Q9CWF6 Bardet-Biedl sy ndr Bbs2 sp|Q9CWF6|BBS2_MOUSE Bardet-Biedl sy ndrome 2 protein homolog OS=Mus musculus OX=10090 GN=Bbs2 PE=1 SV=1

24.2974 24.46139 24.6221 24.43301 24.93975 25.27491 25.18048 24.6184 establishmcell part;intracellular membrane- 8 8 8 35.7 35.7 35.7 32.63 0 20.139 28.14014 30 4 4 4 4 4 6 7 6 1.848354474 0.303428571 0.549911976 3.417973 Q5SWT3;A0A0A Q5SWT3; Solute carrier f ami Slc25a35 sp|Q5SWT3|S2535_MOUSE Solute carrier f amily 25 member 35 OS=Mus musculus OX=10090 GN=Slc25a35 PE=1 SV=2;tr|A0A0A0MQ70|A0A0A0MQ70_MOUSE Solute carrier f amily 25 member 35 OS=Mus musculus OX=10090 GN=Slc25a35 PE=1 SV=1

29.28881 29.39611 29.19779 29.3441 29.43446 29.60072 29.65378 29.46221 anatomica cell part;c Axon guid REACT_2 42 42 36 39.6 39.6 34.9 160.61 0 229.67 32.74047 222 32 30 29 27 31 35 37 31 1.839692945 0.310545455 0.231088161 3.4017561 F8VPK5;A0A1Y 7 F8VPK5;A Rho-associated pr Rock2 tr|F8VPK5|F8VPK5_MOUSE Rho-associated protein kinase OS=Mus musculus OX=10090 GN=Rock2 PE=1 SV=1;tr|A0A1Y 7VMN0|A0A1Y 7VMN0_MOUSE Rho-associated protein kinase 2 (Fragment) OS=Mus musculus OX=10090 GN=Rock2 PE=1 SV=1;sp|P70336|ROCK2_MOUSE Rho-associated prot

21.07978 20.71473 21.03432 21.51424 21.47041 22.36975 22.35877 21.85064 anatomica cell part;cy toplasm;nucleoplasm 1 1 1 2.1 2.1 2.1 100.78 0 3.7732 24.68736 2 0 0 0 0 1 1 1 1 1.838519107 0.309905512 0.926623821 3.3995608 Q8VBW5 Q8VBW5 HMG box transcrip Bbx sp|Q8VBW5|BBX_MOUSE HMG box transcription f actor BBX OS=Mus musculus OX=10090 GN=Bbx PE=1 SV=2

31.83684 31.81616 31.8671 31.83176 31.65527 31.66831 31.44973 31.73283 biosy nthe cell part;e Ribosome REACT_2 11 11 11 80.9 80.9 80.9 11.651 0 107.76 34.98749 88 10 10 6 9 8 9 11 9 1.836989104 0.309568627 -0.211429596 -3.3967 P99027;A0A5F8 P99027;A 60S acidic ribosom Rplp2 sp|P99027|RLA2_MOUSE 60S acidic ribosomal protein P2 OS=Mus musculus OX=10090 GN=Rplp2 PE=1 SV=3;tr|A0A5F8MPY 2|A0A5F8MPY 2_MOUSE 60S acidic ribosomal protein P2 OS=Mus musculus OX=10090 GN=Rplp2-ps1 PE=3 SV=1

22.35517 22.04076 22.04856 21.76203 20.64615 21.72103 21.09249 20.31178 cellular m cell part;m Ribosome biogenesis 1 1 1 7.8 7.8 7.8 29.473 0.004778 1.7337 24.1055 1 1 1 1 1 0 0 0 0 1.828697855 0.313984375 -1.108768463 -3.381217 O88796 O88796 Ribonuclease P pr Rpp30 sp|O88796|RPP30_MOUSE Ribonuclease P protein subunit p30 OS=Mus musculus OX=10090 GN=Rpp30 PE=1 SV=1

31.56061 31.46509 31.4747 31.57326 31.61601 31.77992 32.00919 31.82297 anatomica cell part;c Cell cy cle REACT_2 24 22 21 78.5 78.5 78.5 28.211 0 178.67 35.02015 249 20 19 19 19 18 20 20 19 1.823956093 0.315642023 0.288607597 3.3723752 P68510 P68510 14-3-3 protein eta Ywhah sp|P68510|1433F_MOUSE 14-3-3 protein eta OS=Mus musculus OX=10090 GN=Ywhah PE=1 SV=2

28.14448 28.24241 28.29518 28.0876 28.36861 28.46447 28.31147 28.52223 cellular co cell part;e Cell cy cle - Cauloba 8 8 8 37.1 37.1 37.1 29.8 0 27.324 31.6168 75 6 6 6 6 8 8 8 7 1.822690224 0.315426357 0.22427845 3.3700164 O88696 O88696 ATP-dependent Cl Clpp sp|O88696|CLPP_MOUSE ATP-dependent Clp protease proteoly tic subunit, mitochondrial OS=Mus musculus OX=10090 GN=Clpp PE=1 SV=1

25.06394 24.39833 23.72484 25.00749 21.42676 21.504 19.68197 23.70588 response cell part;intracellular REACT_3 2 2 2 11.8 11.8 11.8 43.001 0 5.449 27.30635 8 1 1 1 1 1 1 1 2 1.821288851 0.315196911 -2.968995094 -3.367406 A0A3B2W4I8;E9 A0A3B2W Chloride channel p Clcn7 tr|A0A3B2W4I8|A0A3B2W4I8_MOUSE H(+)/Cl(-) exchange transporter 7 OS=Mus musculus OX=10090 GN=Clcn7 PE=1 SV=1;tr|E9PY L4|E9PY L4_MOUSE Chloride channel protein OS=Mus musculus OX=10090 GN=Clcn7 PE=1 SV=1;tr|F6SUM2|F6SUM2_MOUSE Chloride channel protein OS=Mus

23.51629 23.81929 24.01733 23.56326 23.96164 24.93333 24.54481 24.62014 anatomica cell body ; Acute my REACT_2 5 5 5 9.4 9.4 9.4 67.581 0 5.5595 27.61119 11 3 3 2 2 4 3 4 3 1.816500879 0.317261538 0.785935879 3.3584933 P04627;Q8CAD1 P04627;Q Serine/threonine-pr Araf sp|P04627|ARAF_MOUSE Serine/threonine-protein kinase A-Raf OS=Mus musculus OX=10090 GN=Araf PE=1 SV=2;tr|Q8CAD1|Q8CAD1_MOUSE Non-specif ic serine/threonine protein kinase OS=Mus musculus OX=10090 GN=Araf PE=1 SV=1;tr|B1AUN8|B1AUN8_MOUSE Non-specif ic serine/

31.10718 30.99739 30.96334 30.86681 31.42547 31.32367 31.07508 31.30177 catabolic cell part;e Aminoben REACT_2 18 18 18 71 71 71 31.474 0 164.55 34.46057 177 16 16 16 15 15 13 16 16 1.81004101 0.319785441 0.297818661 3.3464839 Q8BH95 Q8BH95 Enoy l-CoA hy drat Echs1 sp|Q8BH95|ECHM_MOUSE Enoy l-CoA hy dratase, mitochondrial OS=Mus musculus OX=10090 GN=Echs1 PE=1 SV=1

26.49186 26.04964 25.88807 26.16262 26.5123 26.54271 26.69392 26.86715 biological regulation;b Purine me REACT_3 12 12 12 24.8 24.8 24.8 89.567 0 43.432 29.80466 45 4 3 5 3 5 11 9 8 1.807237724 0.320549618 0.505975246 3.3412779 A0A1L1SRX2;O0 A0A1L1SRAMP deaminase 3 Ampd3 tr|A0A1L1SRX2|A0A1L1SRX2_MOUSE AMP deaminase OS=Mus musculus OX=10090 GN=Ampd3 PE=1 SV=1;sp|O08739|AMPD3_MOUSE AMP deaminase 3 OS=Mus musculus OX=10090 GN=Ampd3 PE=1 SV=2

27.28947 27.44664 27.31086 27.55105 28.11148 27.88379 27.76338 27.56107 amine me extracellul Alanine, a REACT_3 17 17 17 52.8 52.8 52.8 51.739 0 82.323 30.91933 66 12 11 11 11 8 11 12 14 1.785433562 0.333536122 0.430422783 3.3008976 Q91Y I0;E0CY 49 Q91Y I0;E Argininosuccinate Asl sp|Q91Y I0|ARLY _MOUSE Argininosuccinate ly ase OS=Mus musculus OX=10090 GN=Asl PE=1 SV=1;tr|E0CY 49|E0CY 49_MOUSE Argininosuccinate ly ase (Fragment) OS=Mus musculus OX=10090 GN=Asl PE=1 SV=1

21.43781 20.25297 19.08348 22.53248 23.53528 23.68603 23.74632 22.65907 anatomica cell;cell pa Adherens REACT_2 1 1 1 3.6 3.6 3.6 47.575 0.006926 1.5872 26.1616 2 1 0 0 1 1 1 1 1 1.77465491 0.342045455 2.579993248 3.281009 Q9D5H8;E9Q41 Q9D5H8; Receptor protein s Tgf br1 tr|Q9D5H8|Q9D5H8_MOUSE Receptor protein serine/threonine kinase OS=Mus musculus OX=10090 GN=Tgf br1 PE=1 SV=1;tr|E9Q418|E9Q418_MOUSE Receptor protein serine/threonine kinase OS=Mus musculus OX=10090 GN=Tgf br1 PE=1 SV=1;sp|Q64729|TGFR1_MOUSE TGF-beta recepto

27.62199 27.43952 27.28842 27.74631 27.77693 28.15914 27.96681 27.88174 cellular co cell part;endosome;intracellular 26 26 26 25.6 25.6 25.6 162.33 0 133.53 31.09005 116 11 15 13 12 15 19 20 18 1.767401911 0.343833962 0.422096729 3.2676526 Q8VDC1;A0A14 Q8VDC1; FYVE and coiled-c Fy co1 sp|Q8VDC1|FY CO1_MOUSE FYVE and coiled-coil domain-containing protein 1 OS=Mus musculus OX=10090 GN=Fy co1 PE=1 SV=1;tr|A0A140T8V9|A0A140T8V9_MOUSE FYVE and coiled-coil domain-containing protein 1 (Fragment) OS=Mus musculus OX=10090 GN=Fy co1 PE=1 SV=3

26.18285 25.64254 25.95105 25.98311 25.4406 25.15731 25.41735 25.69928 cell part 5 5 5 46.5 46.5 46.5 25.222 0 23.029 28.91929 25 4 5 4 3 2 3 3 4 1.758927811 0.349428571 -0.511255741 -3.252075 Q8BTZ5 Q8BTZ5 Anky rin repeat do Ankrd46 sp|Q8BTZ5|ANR46_MOUSE Anky rin repeat domain-containing protein 46 OS=Mus musculus OX=10090 GN=Ankrd46 PE=1 SV=1

28.81363 28.97812 29.50472 29.14156 28.53971 28.7081 28.61101 28.60703 biological cell part;m Spliceoso REACT_2 5 5 5 19.6 19.6 19.6 44.355 0 40.913 32.14105 42 4 4 4 3 4 4 5 4 1.756968036 0.350277154 -0.493043423 -3.248476 Q8QZY 9 Q8QZY 9 Splicing f actor 3B Sf 3b4 sp|Q8QZY 9|SF3B4_MOUSE Splicing f actor 3B subunit 4 OS=Mus musculus OX=10090 GN=Sf 3b4 PE=1 SV=1

25.25634 25.17192 25.07451 25.44247 25.90412 25.57066 25.42822 25.70253 cell part;macromolecular compl 4 4 3 14 14 11.9 37.271 0 15.769 28.6567 16 4 4 4 3 3 3 3 3 1.756417299 0.350059701 0.415071964 3.2474652 E9PYD1;F6ZXW E9PYD1;F6ZXW2;D3Z100;F Fam98c tr|E9PYD1|E9PY D1_MOUSE Family with sequence similarity 98, member C OS=Mus musculus OX=10090 GN=Fam98c PE=1 SV=1;tr|F6ZXW2|F6ZXW2_MOUSE Family with sequence similarity 98, member C (Fragment) OS=Mus musculus OX=10090 GN=Fam98c PE=1 SV=1;tr|D3Z100|D3Z100_MO

24.82435 25.16818 25.06011 25.05883 25.81836 25.3433 25.37971 25.34452 biological cell part;cell projection;cy toplas 8 8 8 9 9 9 135.62 0 29.003 28.55677 21 4 3 4 2 3 6 7 5 1.754497273 0.350111524 0.443606377 3.2439416 F8VQK5;P59808 F8VQK5;PSAM and SH3 dom Sash1 tr|F8VQK5|F8VQK5_MOUSE SAM and SH3 domain-containing protein 1 OS=Mus musculus OX=10090 GN=Sash1 PE=1 SV=1;sp|P59808|SASH1_MOUSE SAM and SH3 domain-containing protein 1 OS=Mus musculus OX=10090 GN=Sash1 PE=1 SV=1

21.77489 23.45375 20.6177 19.97877 24.15638 24.14227 23.64515 23.85053 biological cell part;intracellular membrane- 4 4 4 10.5 10.5 10.5 71.035 0.000792 2.5099 26.67479 5 0 1 0 0 2 3 4 4 1.744990762 0.357896296 2.492305756 3.2265171 A0A1D5RM90;B A0A1D5R Transcription f acto Tcf 25 tr|A0A1D5RM90|A0A1D5RM90_MOUSE Transcription f actor 25 OS=Mus musculus OX=10090 GN=Tcf 25 PE=1 SV=1;tr|B2ZAC8|B2ZAC8_MOUSE Nuclear localized protein-1 isof orm d (Fragment) OS=Mus musculus OX=10090 GN=Tcf 25 PE=1 SV=1;sp|Q8R3L2|TCF25_MOUSE Transcription f acto

20.77689 21.80443 23.34868 19.96504 23.65042 23.95189 24.12171 23.69019 autophagy cell part;cy toplasm;endomembr 7 7 7 3.8 3.8 3.8 394.29 0 22.686 26.83774 11 0 1 2 0 1 5 5 3 1.743929218 0.357653137 2.379793644 3.2245737 G3UY W1;A0A1DG3UYW1; WD repeat and FY Wdf y3 tr|G3UYW1|G3UY W1_MOUSE WD repeat and FYVE domain-containing protein 3 OS=Mus musculus OX=10090 GN=Wdf y 3 PE=1 SV=1;tr|A0A1D5RLV7|A0A1D5RLV7_MOUSE WD repeat and FYVE domain-containing protein 3 OS=Mus musculus OX=10090 GN=Wdf y 3 PE=1 SV=1;sp|Q6VNB8|WDFY 3_MOUS

20.82895 19.86487 21.76876 20.70451 19.36444 19.19316 19.6554 19.69743 biological cell part;c Nucleotide REACT_2 1 1 1 0.9 0.9 0.9 130.81 0.004093 1.9006 21.97865 1 0 0 0 0 1 1 1 1 1.742534714 0.357323529 -1.314166546 -3.222021 E9QM61;P35689 E9QM61;P DNA repair protein Ercc5 tr|E9QM61|E9QM61_MOUSE DNA repair protein-complementing XP-G cells homolog OS=Mus musculus OX=10090 GN=Ercc5 PE=1 SV=1;sp|P35689|ERCC5_MOUSE DNA repair protein complementing XP-G cells homolog OS=Mus musculus OX=10090 GN=Ercc5 PE=1 SV=4

23.58795 24.67568 25.08974 24.18101 20.28497 19.83577 20.76608 23.94235 cell part;cy toplasm;Golgi appar 2 2 2 2.6 2.6 2.6 111.71 0.003196 1.9512 27.02707 7 2 1 1 1 1 0 1 1 1.742299157 0.356483516 -3.176304817 -3.22159 Q8BWZ3;G3UZ5 Q8BWZ3; N-alpha-acety ltran Naa25 sp|Q8BWZ3|NAA25_MOUSE N-alpha-acety ltransf erase 25, NatB auxiliary subunit OS=Mus musculus OX=10090 GN=Naa25 PE=1 SV=1;tr|G3UZ51|G3UZ51_MOUSE N-alpha-acety ltransf erase 25, NatB auxiliary subunit (Fragment) OS=Mus musculus OX=10090 GN=Naa25 PE=1 SV=1

30.42071 30.4181 30.58916 30.71645 30.29143 30.3434 30.16087 30.2922 biosy nthe cell part;cy toplasm;mREACT_3 9 9 9 61.8 61.8 61.8 24.693 0 143.15 33.65811 132 9 9 8 8 9 9 9 9 1.740706398 0.356510949 -0.2641325 -3.218676 O70251;A0A087 O70251;A Elongation f actor 1 Eef 1b;Eef sp|O70251|EF1B_MOUSE Elongation f actor 1-beta OS=Mus musculus OX=10090 GN=Eef 1b PE=1 SV=5;tr|A0A087WS46|A0A087WS46_MOUSE Eukary otic translation elongation f actor 1 beta 2 OS=Mus musculus OX=10090 GN=Eef 1b2 PE=1 SV=1

19.35315 20.60333 22.98815 19.85159 23.06955 23.26699 23.32773 23.5285 biosy nthe cell part;intracellular REACT_2 4 4 4 21.4 21.4 21.4 20.68 0.002311 2.1076 26.31902 4 1 1 1 1 1 2 2 2 1.733890063 0.360683636 2.599137783 3.2062162 Q9CQF0;A0A49 Q9CQF0; 39S ribosomal prot Mrpl11 sp|Q9CQF0|RM11_MOUSE 39S ribosomal protein L11, mitochondrial OS=Mus musculus OX=10090 GN=Mrpl11 PE=1 SV=1;tr|A0A494B9H5|A0A494B9H5_MOUSE 39S ribosomal protein L11, mitochondrial (Fragment) OS=Mus musculus OX=10090 GN=Mrpl11 PE=1 SV=1;tr|A0A494B9R3|A0A494B

28.21854 28.18589 28.11202 27.88215 28.22871 28.73767 28.50945 28.65564 amine me cell part;c One carbo REACT_3 27 27 27 46.1 46.1 46.1 107.5 0 161.12 31.65864 118 21 18 16 17 16 21 21 19 1.733798716 0.359521739 0.433215618 3.2060493 Q64737;D6RCG Q64737 Trif unctional purine Gart sp|Q64737|PUR2_MOUSE Trif unctional purine biosy nthetic protein adenosine-3 OS=Mus musculus OX=10090 GN=Gart PE=1 SV=3

28.69355 28.79421 28.89371 29.08715 29.67857 29.16684 29.12952 29.47798 biological cell part;intracellular membrane- 12 11 11 54 48.1 48.1 35.649 0 37.092 32.36403 74 10 10 10 9 8 8 9 9 1.724525148 0.365776173 0.4960742 3.1891274 Q99JR1 Q99JR1 Siderof lexin-1 Sf xn1 sp|Q99JR1|SFXN1_MOUSE Siderof lexin-1 OS=Mus musculus OX=10090 GN=Sf xn1 PE=1 SV=3

23.81597 23.67196 22.56246 24.78696 22.44133 18.1436 20.64227 20.89824 biological cell part;m Calcium s REACT_3 41 2 2 39.2 1.9 1.9 128.56 0.003493 1.9247 25.6949 3 1 1 1 1 1 0 1 0 1.719450805 0.369482014 -3.177976131 -3.179882 F7AAP4;E9Q828 F7AAP4;E Calcium-transporti Atp2b4 tr|F7AAP4|F7AAP4_MOUSE Calcium-transporting ATPase OS=Mus musculus OX=10090 GN=Atp2b4 PE=1 SV=1;tr|E9Q828|E9Q828_MOUSE Calcium-transporting ATPase OS=Mus musculus OX=10090 GN=Atp2b4 PE=1 SV=1

20.73595 18.79473 21.31014 21.65467 22.79931 22.88972 22.5956 22.41684 biological cell part;cy toplasm;i REACT_3 3 3 2 40.7 40.7 31 12.562 0.00424 1.8812 26.55497 2 1 1 1 2 1 1 1 1 1.713919145 0.37311828 2.051493168 3.1698152 B1AVF2;Q9Z2S7 B1AVF2;Q TSC22 domain f amTsc22d3 tr|B1AVF2|B1AVF2_MOUSE TSC22 domain f amily protein 3 OS=Mus musculus OX=10090 GN=Tsc22d3 PE=1 SV=1;sp|Q9Z2S7|T22D3_MOUSE TSC22 domain f amily protein 3 OS=Mus musculus OX=10090 GN=Tsc22d3 PE=1 SV=2

26.58162 26.97738 26.32074 26.86089 27.47769 27.27719 27.22426 26.99938 catabolic cell part;c Alzheimer' REACT_2 15 15 15 28 28 28 80.362 0 25.907 30.30965 40 6 5 5 4 10 12 10 9 1.709489186 0.376957143 0.559475422 3.1617617 A0A494BBB0;O3 A0A494BB Calpain-1 cataly tic Capn1 tr|A0A494BBB0|A0A494BBB0_MOUSE Calcium-activ ated neutral proteinase 1 (Fragment) OS=Mus musculus OX=10090 GN=Capn1 PE=1 SV=1;sp|O35350|CAN1_MOUSE Calpain-1 cataly tic subunit OS=Mus musculus OX=10090 GN=Capn1 PE=1 SV=1

23.3152 23.16154 23.2798 23.56209 23.70567 23.73975 23.9208 23.49057 establishmcell part;membrane;plasma me 5 5 4 8.3 8.3 7.2 76.278 0 4.0627 26.92875 10 1 1 1 2 2 3 4 4 1.705089339 0.378533808 0.384537697 3.1537704 Q32NY 4 Q32NY 4 Metal transporter CCnnm3 sp|Q32NY 4|CNNM3_MOUSE Metal transporter CNNM3 OS=Mus musculus OX=10090 GN=Cnnm3 PE=1 SV=2

22.89645 23.38903 23.66893 23.18376 23.61628 24.18495 23.85386 24.25162 biological cell part;cy toplasm;intracellular 6 3 0 59.4 34.6 0 14.659 0 28.638 27.14073 13 2 2 1 1 2 2 3 3 1.696986506 0.383106383 0.692135334 3.1390729 A0A0N4SUX1;Q A0A0N4SUX1;Q8CII5;G5E8L Tia1 tr|A0A0N4SUX1|A0A0N4SUX1_MOUSE Nucleoly sin TIA-1 OS=Mus musculus OX=10090 GN=Tia1 PE=1 SV=1;tr|Q8CII5|Q8CII5_MOUSE Nucleoly sin TIA-1 OS=Mus musculus OX=10090 GN=Tia1 PE=1 SV=1;tr|G5E8L2|G5E8L2_MOUSE Nucleoly sin TIA-1 OS=Mus musculus OX=10090 GN=Tia1 PE=1 S

23.95899 22.20872 22.17703 22.12909 21.24652 19.40115 21.36674 20.51578 cell activ a cell part;in Non-homologous end 1 1 1 8.8 8.8 8.8 32.739 0 15.719 24.85576 1 1 1 1 1 0 0 0 0 1.689384544 0.38780212 -1.985909462 -3.125306 Q3KNJ2 Q3KNJ2 Non-homologous e Nhej1 sp|Q3KNJ2|NHEJ1_MOUSE Non-homologous end-joining f actor 1 OS=Mus musculus OX=10090 GN=Nhej1 PE=1 SV=1

28.02992 28.24223 27.8527 28.10689 28.35699 28.29605 28.27298 28.35405 biological macromolecular complex;protei 16 16 15 53.2 53.2 49.6 42.467 0 88.237 31.43674 80 12 11 10 10 13 11 13 12 1.687408162 0.387647887 0.262085915 3.121731 B1AT82;Q9D0M1 B1AT82;Q Phosphoribosy l py Prpsap1 tr|B1AT82|B1AT82_MOUSE Phosphoribosy l py rophosphate sy nthase-associated protein 1 OS=Mus musculus OX=10090 GN=Prpsap1 PE=1 SV=1;sp|Q9D0M1|KPRA_MOUSE Phosphoribosy l py rophosphate sy nthase-associated protein 1 OS=Mus musculus OX=10090 GN=Prpsap1 PE=1 SV=1

25.96548 26.28773 26.25979 26.13809 26.52634 26.57946 26.41171 26.31164 lipid meta cell part;m Steroid hormone bios 7 7 7 17 17 17 66.59 0 17.711 29.6406 39 6 6 7 4 5 6 7 7 1.681451319 0.390498246 0.294512272 3.1109634 P50427 P50427 Stery l-sulf atase Sts sp|P50427|STS_MOUSE Stery l-sulf atase OS=Mus musculus OX=10090 GN=Sts PE=1 SV=1

26.59875 26.53508 26.63727 26.69205 26.19385 26.38359 26.54743 26.17722 11 11 11 34 34 34 54.044 0 24.234 29.80286 44 6 7 7 3 7 7 8 5 1.67686696 0.392671329 -0.290267944 -3.102686 Q8VE88 Q8VE88 Protein FAM114A2 Fam114a2 sp|Q8VE88|F1142_MOUSE Protein FAM114A2 OS=Mus musculus OX=10090 GN=Fam114a2 PE=1 SV=2

21.54472 21.85285 21.94913 21.91838 20.92956 19.7763 19.33405 21.31646 cellular co cell body ;cell part;cell projection 6 3 3 6.2 2.8 2.8 190.97 0.002321 2.155 23.80607 3 2 1 1 1 0 0 0 1 1.67249875 0.394522648 -1.477180004 -3.094806 E9QAN4;G3UW E9QAN4; Kinesin-like protein Kif 1a tr|E9QAN4|E9QAN4_MOUSE Kinesin-like protein KIF1A OS=Mus musculus OX=10090 GN=Kif 1a PE=1 SV=1;tr|G3UW47|G3UW47_MOUSE Kinesin-like protein KIF1A OS=Mus musculus OX=10090 GN=Kif 1a PE=1 SV=1;tr|Q6TA13|Q6TA13_MOUSE Kinesin-like protein KIF1A OS=Mus musculus OX

21.65119 21.61106 24.27455 20.27668 24.08562 25.05957 25.18465 24.29936 biological cell part;intracellular membrane- 4 4 4 10.1 10.1 10.1 63.538 0 4.4119 27.63582 8 0 0 1 0 2 4 3 3 1.659302716 0.406472222 2.703929901 3.071043 Q9JHW4;A0A0N Q9JHW4; Selenocy steine-sp Eef sec sp|Q9JHW4|SELB_MOUSE Selenocy steine-specif ic elongation f actor OS=Mus musculus OX=10090 GN=Eef sec PE=1 SV=2;tr|A0A0N4SUV6|A0A0N4SUV6_MOUSE Selenocy steine-specif ic elongation f actor OS=Mus musculus OX=10090 GN=Eef sec PE=1 SV=1;tr|A0A0N4SVA4|A0A0N4SVA4_MOUSE

25.12814 25.29204 25.1102 25.53917 26.17575 25.90104 25.39398 25.97821 biological cell part;cell projectio REACT_2 14 14 14 18 18 18 120.23 0 30.748 28.88951 41 8 6 5 7 10 11 10 12 1.657702259 0.406768166 0.594860554 3.0681653 B1AXI9;J3QMP9 B1AXI9;J3 Centrosomal prote Cep131 tr|B1AXI9|B1AXI9_MOUSE 5-azacy tidine induced gene 1 OS=Mus musculus OX=10090 GN=Cep131 PE=1 SV=1;tr|J3QMP9|J3QMP9_MOUSE Centrosomal protein of 131 kDa OS=Mus musculus OX=10090 GN=Cep131 PE=1 SV=1;sp|Q62036|CP131_MOUSE Centrosomal protein of 131 kDa OS=Mus

24.62611 22.13693 23.66709 22.10604 20.0628 20.00161 22.25751 20.10626 anatomica apical part Vasopress REACT_3 5 5 5 30.2 30.2 30.2 39.469 0 6.3035 28.36093 5 3 2 2 2 3 1 2 3 1.651562668 0.413089655 -2.526998043 -3.057135 Q8K0T2 Q8K0T2 Cy toplasmic dy ne Dy nc2li1 sp|Q8K0T2|DC2L1_MOUSE Cy toplasmic dy nein 2 light intermediate chain 1 OS=Mus musculus OX=10090 GN=Dy nc2li1 PE=1 SV=1

24.3534 23.72993 23.82114 24.16323 24.55374 24.52576 25.56491 24.87526 anatomica cell part;endosome;intracellular 8 8 8 6.9 6.9 6.9 180.2 0 36.845 27.9989 29 4 2 2 1 3 3 6 6 1.651435635 0.411670103 0.862991333 3.0569067 Q69Z37;E9PX59 Q69Z37;E Sterile alpha motif Samd9l sp|Q69Z37|SAM9L_MOUSE Sterile alpha motif domain-containing protein 9-like OS=Mus musculus OX=10090 GN=Samd9l PE=1 SV=2;tr|E9PX59|E9PX59_MOUSE Sterile alpha motif domain-containing protein 9-like OS=Mus musculus OX=10090 GN=Samd9l PE=1 SV=1

26.77505 26.85448 26.69883 27.04562 27.47507 27.08598 27.10629 27.14734 biological cell part;c Chemokin REACT_2 7 7 7 34.4 34.4 34.4 43.565 0 78.073 30.25878 52 6 5 4 4 5 5 7 5 1.650610045 0.411273973 0.360176563 3.0554245 P62881 P62881 Guanine nucleotide Gnb5 sp|P62881|GNB5_MOUSE Guanine nucleotide-binding protein subunit beta-5 OS=Mus musculus OX=10090 GN=Gnb5 PE=1 SV=1

21.67969 21.92143 22.41929 22.4139 20.97756 19.2652 21.51091 20.39078 anatomica cell body ;cell part;cell projection 1 1 1 3.5 3.5 3.5 36.28 0.006504 1.6287 24.33518 2 1 1 1 1 0 0 0 0 1.647446861 0.412081911 -1.572464943 -3.049748 A0A2R8VHX5;Q A0A2R8V Zinc f inger protein Znf 385a tr|A0A2R8VHX5|A0A2R8VHX5_MOUSE Zinc f inger protein 385A OS=Mus musculus OX=10090 GN=Zf p385a PE=1 SV=1;sp|Q8VD12|Z385A_MOUSE Zinc f inger protein 385A OS=Mus musculus OX=10090 GN=Znf 385a PE=1 SV=2

25.54259 25.75466 25.3947 25.57228 25.94606 26.03576 25.86802 25.68752 biological cell part;cy toplasm;intracellular 6 6 6 30.5 30.5 30.5 30.232 0 28.472 29.01289 28 5 4 4 4 5 5 6 5 1.643817475 0.414068027 0.318283081 3.043239 A0A1B0GR18;A0 A0A1B0G MOB kinase activ a Mob2 tr|A0A1B0GR18|A0A1B0GR18_MOUSE MOB kinase activ ator 2 OS=Mus musculus OX=10090 GN=Mob2 PE=1 SV=1;tr|A0A1B0GRX6|A0A1B0GRX6_MOUSE MOB kinase activ ator 2 (Fragment) OS=Mus musculus OX=10090 GN=Mob2 PE=1 SV=1;sp|Q8VI63|MOB2_MOUSE MOB kinase activ ator 2 OS=Mus

22.31282 22.29443 22.97215 23.44088 20.73953 19.39068 22.3192 19.2754 biological cell part;c Axon guid REACT_2 3 3 3 6.8 6.8 6.8 67.285 0 3.1333 25.90366 4 2 1 3 1 0 1 1 1 1.64308251 0.413098305 -2.323868752 -3.041922 E9Q0W6;E9Q4K E9Q0W6; Actin-binding LIM p Ablim2 tr|E9Q0W6|E9Q0W6_MOUSE Actin-binding LIM protein 2 OS=Mus musculus OX=10090 GN=Ablim2 PE=1 SV=1;tr|E9Q4K0|E9Q4K0_MOUSE Actin-binding LIM protein 2 OS=Mus musculus OX=10090 GN=Ablim2 PE=1 SV=1;sp|Q8BL65|ABLM2_MOUSE Actin-binding LIM protein 2 OS=Mus musculu

19.62844 20.95433 21.29149 18.50654 21.76276 23.48407 22.43886 21.8418 behav ior;b cell part;membrane REACT_3 2 1 1 24.4 13.7 13.7 18.513 0.004231 1.87 25.27142 1 0 0 0 0 1 1 1 1 1.640210727 0.413256757 2.286669731 3.0367754 F6RT95;B1AXF2 F6RT95;B1AXF2;Q8BUN9 Slc24a2 tr|F6RT95|F6RT95_MOUSE Solute carrier f amily 24 (sodium/potassium/calcium exchanger), member 2 (Fragment) OS=Mus musculus OX=10090 GN=Slc24a2 PE=1 SV=8;tr|B1AXF2|B1AXF2_MOUSE Solute carrier f amily 24 (sodium/potassium/calcium exchanger), member 2 OS=Mus mu

24.78302 24.67066 24.89865 24.68694 20.13773 20.04244 24.6692 19.30807 biological cell part;endoplasmic reticulum; 3 3 3 7 7 7 72.799 0.004239 1.8805 27.20023 6 2 3 3 1 0 0 1 1 1.640138085 0.41186532 -3.72045517 -3.036645 Q8BKS9;A0A0N Q8BKS9;A Pumilio domain-co Kiaa0020 sp|Q8BKS9|PUM3_MOUSE Pumilio homolog 3 OS=Mus musculus OX=10090 GN=Pum3 PE=1 SV=2;tr|A0A0N4SUH4|A0A0N4SUH4_MOUSE Pumilio homolog 3 OS=Mus musculus OX=10090 GN=Pum3 PE=1 SV=1

38.2795 37.68147 38.13542 38.52697 37.60967 37.24458 37.5433 37.73132 cell part;macromolec REACT_2 27 27 9 97.3 97.3 38.1 15.748 0 323.31 41.10583 1878 27 26 27 26 24 23 25 25 1.638670025 0.41138255 -0.623621941 -3.034016 A8DUK4;E9Q22 A8DUK4;E9Q223 Hbbt1;Hb tr|A8DUK4|A8DUK4_MOUSE Beta-globin OS=Mus musculus OX=10090 GN=Hbb-bs PE=1 SV=1;tr|E9Q223|E9Q223_MOUSE Hemoglobin, beta adult s chain (Fragment) OS=Mus musculus OX=10090 GN=Hbb-bs PE=1 SV=1

24.78736 24.48953 24.81948 24.47871 24.0573 24.34171 24.34232 23.71963 cell death; cell part;cy toplasm;endoplasmi 7 7 7 11.7 11.7 11.7 71.302 0 13 27.67566 16 4 5 5 3 2 4 4 4 1.63614652 0.412521739 -0.528532505 -3.029498 D3Z6S1;Q8BM5 D3Z6S1;Q Transmembrane pr Tmem214 tr|D3Z6S1|D3Z6S1_MOUSE Transmembrane protein 214 OS=Mus musculus OX=10090 GN=Tmem214 PE=1 SV=3;sp|Q8BM55|TM214_MOUSE Transmembrane protein 214 OS=Mus musculus OX=10090 GN=Tmem214 PE=1 SV=1

22.80089 22.43883 22.57071 22.74273 22.90277 22.80126 23.13591 23.13242 cellular co cell part;centrosome; REACT_2 5 2 2 4.5 2.4 2.4 195.7 0 3.6084 26.06363 9 2 2 1 1 1 1 1 1 1.626601404 0.42196 0.354801178 3.0124281 F8VQ75;Q9EQW F8VQ75;Q Kinesin-like protein Kif 13a tr|F8VQ75|F8VQ75_MOUSE Kinesin-like protein KIF13A OS=Mus musculus OX=10090 GN=Kif 13a PE=1 SV=1;sp|Q9EQW7|KI13A_MOUSE Kinesin-like protein KIF13A OS=Mus musculus OX=10090 GN=Kif 13a PE=1 SV=1

21.12923 22.083 19.60153 19.73146 22.70881 22.30042 22.28825 22.47912 cellular component organization;cellular me 3 3 3 7.4 7.4 7.4 70.083 0 4.0557 25.15221 3 1 0 0 0 2 1 1 1 1.62275533 0.423774086 1.807843208 3.0055592 D3Y WC9;Q6P9 D3YWC9; MTSS1-like protein Mtss1l tr|D3YWC9|D3Y WC9_MOUSE Protein MTSS 2 OS=Mus musculus OX=10090 GN=Mtss2 PE=1 SV=1;sp|Q6P9S0|MTSS2_MOUSE Protein MTSS 2 OS=Mus musculus OX=10090 GN=Mtss2 PE=1 SV=1;tr|F7D291|F7D291_MOUSE Protein MTSS 2 (Fragment) OS=Mus musculus OX=10090 GN=Mtss2 PE=1 SV=1

21.75136 22.79448 22.45413 22.81029 19.99566 20.48455 22.23701 20.47223 2 2 2 6.8 6.8 6.8 40.433 0.004095 1.9109 24.4128 2 1 1 1 1 0 1 0 0 1.619057624 0.427311258 -1.655198574 -2.99896 Q91W67;A0A1L1 Q91W67; Ubiquitin-like prote Ubl7 sp|Q91W67|UBL7_MOUSE Ubiquitin-like protein 7 OS=Mus musculus OX=10090 GN=Ubl7 PE=1 SV=2;tr|A0A1L1SRW2|A0A1L1SRW2_MOUSE Ubiquitin-like protein 7 (Fragment) OS=Mus musculus OX=10090 GN=Ubl7 PE=1 SV=1;tr|A0A1L1STF2|A0A1L1STF2_MOUSE Ubiquitin-like protein 7 (

20.48977 23.70852 21.8035 18.83183 24.75466 25.19577 24.00496 23.83316 cell part;cy toplasm 9 1 1 52.5 6 6 21.706 0 3.6712 27.28472 5 1 1 0 0 1 1 1 1 1.618987274 0.42590099 3.238733768 2.9988347 Q8BKP1;Q3TUJ Q8BKP1;Q3TUJ9;F6VQ81 Tpd52l2 tr|Q8BKP1|Q8BKP1_MOUSE Tumor protein D54 OS=Mus musculus OX=10090 GN=Tpd52l2 PE=1 SV=1;tr|Q3TUJ9|Q3TUJ9_MOUSE Tumor protein D54 OS=Mus musculus OX=10090 GN=Tpd52l2 PE=1 SV=1;tr|F6VQ81|F6VQ81_MOUSE Tumor protein D54 (Fragment) OS=Mus musculus OX=10090 GN=Tp

25.72046 25.95912 25.74258 25.92159 25.923 26.13825 26.22131 26.10821 6 6 6 36.7 36.7 36.7 24.33 0 14.162 29.31786 28 5 4 5 4 4 5 5 5 1.61266211 0.430697368 0.261754513 2.9875578 Q8VE95;Q9D1W Q8VE95 UPF0598 protein C8orf 82 ho sp|Q8VE95|CH082_MOUSE UPF0598 protein C8orf 82 homolog OS=Mus musculus OX=10090 PE=1 SV=1

19.58298 20.79267 22.58097 19.10726 22.58987 22.79687 22.70428 23.8399 biosy nthe cell part;c mRNA su REACT_2 4 4 4 32.1 32.1 32.1 18.934 0.000637 2.5872 26.45513 4 2 2 2 0 1 1 2 1 1.611030163 0.430937705 2.466760635 2.9846505 E9QAY 4;A0A0R E9QAY 4; Poly (A) poly meras Papola tr|E9QAY 4|E9QAY 4_MOUSE Poly nucleotide adeny ly ltransf erase OS=Mus musculus OX=10090 GN=Papola PE=1 SV=1;tr|A0A0R4J244|A0A0R4J244_MOUSE Poly nucleotide adeny ly ltransf erase OS=Mus musculus OX=10090 GN=Papola PE=1 SV=1;tr|E9PWC8|E9PWC8_MOUSE Poly (A) poly merase

22.72673 20.86763 23.36706 22.22858 19.70801 20.34104 19.87539 21.44197 extracellular organelle;organelle; 1 1 1 3.2 3.2 3.2 91.921 0 4.617 24.87591 2 1 1 1 1 0 0 0 1 1.602351232 0.438287582 -1.9559021 -2.969205 Q9D2I5 Q9D2I5 LisH domain-conta Armc9 sp|Q9D2I5|ARMC9_MOUSE LisH domain-containing protein ARMC9 OS=Mus musculus OX=10090 GN=Armc9 PE=1 SV=1

23.2169 23.01793 22.68945 23.08049 22.37359 20.91469 21.60291 19.34328 biological cell part;cell projectio REACT_2 15 1 1 21.1 1.4 1.4 123.69 0.004496 1.7809 24.92007 1 1 1 1 1 0 0 0 0 1.601950904 0.437798046 -1.942567825 -2.968493 E9PY 13;E9PY 12 E9PY 13;E Guanine nucleotide Mcf 2l tr|E9PY 13|E9PY 13_MOUSE Guanine nucleotide exchange f actor DBS OS=Mus musculus OX=10090 GN=Mcf 2l PE=1 SV=1;tr|E9PY 12|E9PY 12_MOUSE Guanine nucleotide exchange f actor DBS OS=Mus musculus OX=10090 GN=Mcf 2l PE=1 SV=1;tr|G3UX72|G3UX72_MOUSE Guanine nucleotide ex

19.665 21.44099 18.55232 24.16791 25.09366 24.53629 24.88362 24.07594 biosy nthe cell part;in Py ruv ate REACT_2 3 3 3 12 12 12 51.847 0 7.3456 27.10089 5 0 0 0 1 3 2 2 2 1.600187098 0.439298701 3.690825462 2.965357 Q7TNG8 Q7TNG8 Probable D-lactate Ldhd sp|Q7TNG8|LDHD_MOUSE Probable D-lactate dehy drogenase, mitochondrial OS=Mus musculus OX=10090 GN=Ldhd PE=1 SV=1

20.75009 20.15925 21.34692 19.73868 19.3483 19.27232 19.76268 19.20641 biological cell part;cy toplasm;intracellular 3 3 3 3.6 3.6 3.6 130.66 0.007479 1.5377 23.18057 3 0 0 1 0 1 2 1 1 1.59599951 0.441294498 -1.101304531 -2.957917 E9QKD1;Q3UHX E9QKD1; Nucleolar protein 8 Nol8 tr|E9QKD1|E9QKD1_MOUSE Nucleolar protein 8 OS=Mus musculus OX=10090 GN=Nol8 PE=1 SV=1;sp|Q3UHX0|NOL8_MOUSE Nucleolar protein 8 OS=Mus musculus OX=10090 GN=Nol8 PE=1 SV=2

29.95241 29.94 29.8935 30.09119 30.12325 30.24252 30.03983 30.23866 alcohol m cell part;c Inositol ph REACT_2 16 16 16 61 61 61 30.429 0 108.27 33.27017 150 15 15 15 15 13 16 15 15 1.59467933 0.440954839 0.191790581 2.9555722 Q924B0;O55023 Q924B0;O Inositol monophos Impa1 tr|Q924B0|Q924B0_MOUSE Inositol-1-monophosphatase OS=Mus musculus OX=10090 GN=Impa1 PE=1 SV=1;sp|O55023|IMPA1_MOUSE Inositol monophosphatase 1 OS=Mus musculus OX=10090 GN=Impa1 PE=1 SV=1;tr|Q80ZJ2|Q80ZJ2_MOUSE Inositol-1-monophosphatase OS=Mus musculus OX=

31.7384 31.74347 31.71338 31.71514 31.72777 31.97333 31.94465 31.98142 cell recog cell body ;cell part;ce REACT_2 35 35 35 81.3 81.3 81.3 59.623 0 310.47 35.14843 350 30 30 30 32 29 32 32 31 1.592934833 0.440578778 0.179194927 2.9524752 P80316;E0CZA1 P80316 T-complex protein Cct5 sp|P80316|TCPE_MOUSE T-complex protein 1 subunit epsilon OS=Mus musculus OX=10090 GN=Cct5 PE=1 SV=1

27.09637 26.79352 26.75875 26.57888 27.01808 27.31034 27.21364 27.60406 cell part;extracellular organelle;i 7 7 7 33.7 33.7 33.7 32.032 0 23.92 30.33405 37 5 5 4 5 6 6 6 6 1.591994764 0.439730769 0.479646683 2.9508067 Q91V64;A0A494 Q91V64;A Isochorismatase d Isoc1 sp|Q91V64|ISOC1_MOUSE Isochorismatase domain-containing protein 1 OS=Mus musculus OX=10090 GN=Isoc1 PE=1 SV=1;tr|A0A494B952|A0A494B952_MOUSE Isochorismatase domain-containing protein 1 OS=Mus musculus OX=10090 GN=Isoc1 PE=1 SV=1

25.09993 24.62556 24.94659 24.78756 25.35121 25.17303 25.05953 25.32347 7 7 7 30.3 30.3 30.3 43.126 0 22.433 28.31363 27 4 6 5 5 2 3 3 3 1.590284123 0.439884984 0.361900806 2.9477713 Q8K0G5;A0A1Y Q8K0G5;A Protein TSSC1 Tssc1 sp|Q8K0G5|EIPR1_MOUSE EARP and GARP complex-interacting protein 1 OS=Mus musculus OX=10090 GN=Eipr1 PE=1 SV=2;tr|A0A1Y 7VN36|A0A1Y 7VN36_MOUSE EARP and GARP complex-interacting protein 1 (Fragment) OS=Mus musculus OX=10090 GN=Eipr1 PE=1 SV=1

27.48278 27.58183 27.24943 27.51518 27.07826 27.33952 27.17655 27.17055 anatomica cell part;Golgi appara REACT_3 32 32 32 25.7 25.7 25.7 226.45 0 102.5 30.59272 88 13 14 17 16 17 22 22 18 1.587474831 0.441312102 -0.266086102 -2.942789 E9Q512;H3BJG4 E9Q512;H3BJG4 Trip11 tr|E9Q512|E9Q512_MOUSE Thy roid hormone receptor interactor 11 OS=Mus musculus OX=10090 GN=Trip11 PE=1 SV=1;tr|H3BJG4|H3BJG4_MOUSE Thy roid hormone receptor interactor 11 (Fragment) OS=Mus musculus OX=10090 GN=Trip11 PE=1 SV=1

26.24178 26.10187 25.89885 25.99688 26.44993 26.33441 26.39997 26.17307 anatomica cell part;cy toplasm;c REACT_3 6 6 6 15.6 15.6 15.6 65.853 0 22.419 29.44908 26 5 4 4 5 3 5 5 5 1.58685751 0.440165079 0.279497147 2.941694 Q9WVG6;D3YU Q9WVG6; Histone-arginine m Carm1 sp|Q9WVG6|CARM1_MOUSE Histone-arginine methy ltransf erase CARM1 OS=Mus musculus OX=10090 GN=Carm1 PE=1 SV=2;tr|D3Y UP1|D3Y UP1_MOUSE Coactiv ator-associated arginine methy ltransf erase 1 OS=Mus musculus OX=10090 GN=Carm1 PE=1 SV=1

21.29513 19.79762 20.66761 18.41202 21.51167 22.15001 22.30031 21.83434 anatomica cell part;histone met REACT_3 4 4 4 3.9 3.9 3.9 219.96 0.00064 2.6612 26.38652 10 1 2 0 3 1 1 1 1 1.585394013 0.440227848 1.905984879 2.9390996 Q5XJV5;A2AQM Q5XJV5;A Nuclear receptor c Ncoa6 tr|Q5XJV5|Q5XJV5_MOUSE Nuclear receptor coactiv ator 6 OS=Mus musculus OX=10090 GN=Ncoa6 PE=1 SV=1;tr|A2AQM9|A2AQM9_MOUSE Nuclear receptor coactiv ator 6 (Fragment) OS=Mus musculus OX=10090 GN=Ncoa6 PE=1 SV=8;sp|Q9JL19|NCOA6_MOUSE Nuclear receptor coactiv ato

21.6483 21.13289 22.10287 21.85003 22.6446 22.87923 22.53129 21.97179 anatomica cell part;cy toplasm;e REACT_2 2 2 2 4.3 4.3 4.3 74.609 0 4.0711 25.60685 13 1 1 1 1 1 2 2 2 1.575654253 0.44859306 0.823209286 2.9218517 Q61592 Q61592 Growth arrest-spec Gas6 sp|Q61592|GAS6_MOUSE Growth arrest-specif ic protein 6 OS=Mus musculus OX=10090 GN=Gas6 PE=2 SV=2

30.64108 30.69702 31.00243 30.73295 29.93255 30.32967 30.58118 30.3872 biological cell part;c Collecting REACT_2 27 27 27 45.1 45.1 45.1 103.13 0 151.81 33.82781 198 20 24 22 17 19 23 23 22 1.572050294 0.450515723 -0.460717678 -2.915478 P04919 P04919 Band 3 anion trans Slc4a1 sp|P04919|B3AT_MOUSE Band 3 anion transport protein OS=Mus musculus OX=10090 GN=Slc4a1 PE=1 SV=1

24.93418 24.4318 24.85861 24.88962 25.5263 25.27199 25.0466 25.11164 anatomica cell part;intracellular REACT_2 5 3 3 18.6 13.4 13.4 54.161 0 11.695 28.33803 13 2 1 1 3 1 2 2 1 1.570425375 0.45015674 0.460577965 2.912605 E9PUH7;Q3TY K E9PUH7; Nuclear f actor 1;N Nf ix tr|E9PUH7|E9PUH7_MOUSE Nuclear f actor 1 OS=Mus musculus OX=10090 GN=Nf ix PE=1 SV=1;tr|Q3TYK3|Q3TY K3_MOUSE Nuclear f actor 1 OS=Mus musculus OX=10090 GN=Nf ix PE=1 SV=1;tr|D3Y Z00|D3Y Z00_MOUSE Nuclear f actor 1 (Fragment) OS=Mus musculus OX=10090 GN=Nf ix PE=1 S

23.37901 24.22044 24.16285 23.8399 23.34733 22.71697 22.9749 23.50397 biological regulation;c Protein processing in 1 1 1 4.4 4.4 4.4 37.485 0 4.2758 26.7618 8 1 1 1 1 1 1 1 1 1.569328627 0.44985 -0.764759541 -2.910667 Q8CB27 Q8CB27 Ubiquitin thioester Y od1 sp|Q8CB27|OTU1_MOUSE Ubiquitin thioesterase OTU1 OS=Mus musculus OX=10090 GN=Yod1 PE=1 SV=1

22.25854 23.91862 20.67608 24.06751 26.09794 25.1736 24.32855 25.55286 digestion; extracellular organell REACT_3 1 1 1 8.1 8.1 8.1 26.274 0.004363 1.8157 28.48984 3 1 1 0 1 1 1 1 1 1.566333648 0.450990654 2.558049202 2.9053755 Q9R0T7;Q9QUK Q9R0T7;Q9QUK9 Try 4;Try 5 tr|Q9R0T7|Q9R0T7_MOUSE Pancreatic try psin OS=Mus musculus OX=10090 GN=Try 4 PE=1 SV=1;tr|Q9QUK9|Q9QUK9_MOUSE TESP4 OS=Mus musculus OX=10090 GN=Try 5 PE=1 SV=1

27.35984 27.43778 27.35026 27.25332 27.84416 27.78126 27.42101 27.56841 amine me cell part;e Arginine and proline 13 13 13 38.4 38.4 38.4 60.909 0 70.617 30.87247 72 10 11 10 5 9 12 11 10 1.564815582 0.451118012 0.303409576 2.9026947 Q60715;E9Q7B0 Q60715;E Proly l 4-hy droxy la P4ha1 sp|Q60715|P4HA1_MOUSE Proly l 4-hy droxy lase subunit alpha-1 OS=Mus musculus OX=10090 GN=P4ha1 PE=1 SV=2;tr|E9Q7B0|E9Q7B0_MOUSE Procollagen-proline 4-dioxy genase OS=Mus musculus OX=10090 GN=P4ha1 PE=1 SV=1

25.37971 25.79934 26.21764 25.7233 25.40882 25.21638 25.26721 25.10009 cell part;in Spliceosome 6 6 6 30.2 30.2 30.2 23.612 0 7.3073 28.83747 25 4 4 5 3 3 3 3 4 1.56163921 0.452668731 -0.531872272 -2.897088 Q9CPW7 Q9CPW7 Zinc f inger matrin- Zmat2 sp|Q9CPW7|ZMAT2_MOUSE Zinc f inger matrin-ty pe protein 2 OS=Mus musculus OX=10090 GN=Zmat2 PE=2 SV=1

23.03752 23.27113 26.02789 23.16886 20.1572 23.04298 20.42153 20.42072 cell part;membrane;plasma me 3 3 3 14.3 14.3 14.3 32.506 0 9.4661 26.94682 4 1 1 3 1 0 1 0 0 1.560820438 0.452037037 -2.865743637 -2.895643 B1AXV0;F6Q3S1 B1AXV0 DOMON domain-c Frrs1l sp|B1AXV0|FRS1L_MOUSE DOMON domain-containing protein FRRS1L OS=Mus musculus OX=10090 GN=Frrs1l PE=1 SV=1

25.86399 25.35924 25.09023 25.06669 24.57346 24.6381 24.98694 24.78835 cellular co cell part;cy toplasm;cy toplasmic 9 9 9 20.3 20.3 20.3 70.525 0 11.39 28.31203 18 4 4 4 4 4 4 4 6 1.55950656 0.451852308 -0.598323345 -2.893325 P59016;S4R1H4 P59016 Vacuolar protein s Vps33b sp|P59016|VP33B_MOUSE Vacuolar protein sorting-associated protein 33B OS=Mus musculus OX=10090 GN=Vps33b PE=1 SV=1

20.6586 22.06485 21.75193 20.05681 22.87527 22.62299 22.12417 22.60806 cell part;membrane 3 3 3 51.2 51.2 51.2 9.2858 0.000164 3.0064 25.08205 6 1 0 1 0 1 1 1 3 1.553567158 0.455730061 1.424571991 2.8828538 Q8BTE5 Q8BTE5 Protein CEBPZOS Cebpzos sp|Q8BTE5|CEBOS_MOUSE Protein CEBPZOS OS=Mus musculus OX=10090 GN=Cebpzos PE=3 SV=2

25.06283 25.05263 24.6392 25.26112 24.72562 24.15792 24.10773 24.6418 biological cell part;intracellular membrane- 2 2 2 19.2 19.2 19.2 17.307 0 4.8264 28.04046 9 1 1 1 1 2 2 2 2 1.552105992 0.455437309 -0.595677853 -2.880279 Q923X4;B7ZC40 Q923X4;B Glutaredoxin-2, mi Glrx2 sp|Q923X4|GLRX2_MOUSE Glutaredoxin-2, mitochondrial OS=Mus musculus OX=10090 GN=Glrx2 PE=1 SV=1;tr|B7ZC40|B7ZC40_MOUSE Glutaredoxin-2, mitochondrial OS=Mus musculus OX=10090 GN=Glrx2 PE=1 SV=1

32.00949 31.90688 32.01152 31.86515 31.71955 31.87665 31.84206 31.71387 anatomica cell part;c Endocy tosis;Vasopre 15 15 4 62.8 62.8 15.6 24.489 0 165.44 35.09805 124 14 14 12 15 13 12 12 11 1.547785574 0.45952439 -0.160225868 -2.872672 P46638;G3UZD3 P46638;G Ras-related protein Rab11b;R sp|P46638|RB11B_MOUSE Ras-related protein Rab-11B OS=Mus musculus OX=10090 GN=Rab11b PE=1 SV=3;tr|G3UZD3|G3UZD3_MOUSE Ras-related protein Rab-11B OS=Mus musculus OX=10090 GN=Rab11b PE=1 SV=1;tr|G3UY 29|G3UY 29_MOUSE Ras-related protein Rab-11B OS=Mus musculu

21.92369 20.55194 22.56132 21.73331 22.90327 22.94867 22.85516 22.86797 biological cell part;cy toplasm;intracellular 2 2 2 9.1 9.1 9.1 31.139 0 4.4418 25.87692 9 1 1 1 1 1 2 1 2 1.540858376 0.467890578 1.201202393 2.8604859 Q9DCX1 Q9DCX1 MAD2L1-binding pr Mad2l1bp sp|Q9DCX1|MD2BP_MOUSE MAD2L1-binding protein OS=Mus musculus OX=10090 GN=Mad2l1bp PE=1 SV=2

19.88583 20.68232 21.60699 21.85434 23.18456 22.15168 22.16435 22.38572 anatomica cell part;cy toplasm;intracellular 6 3 3 8.9 5.1 5.1 122.46 0 13.352 26.52973 10 1 1 1 1 1 2 2 1 1.538618437 0.468751515 1.464205742 2.8565488 Q64318;E9PXY 5 Q64318;E Zinc f inger E-box- Zeb1 sp|Q64318|ZEB1_MOUSE Zinc f inger E-box-binding homeobox 1 OS=Mus musculus OX=10090 GN=Zeb1 PE=1 SV=1;tr|E9PXY 5|E9PXY 5_MOUSE Zinc f inger E-box-binding homeobox 1 (Fragment) OS=Mus musculus OX=10090 GN=Zeb1 PE=1 SV=8

22.91307 21.00749 17.3131 19.8201 23.51689 25.94313 23.40626 23.37425 catabolic cell part;c Metabolis REACT_2 5 5 5 13.8 13.8 13.8 55.948 0 10.705 27.42021 13 1 0 0 0 2 4 1 3 1.538030961 0.468507553 3.796691895 2.8555165 P33267 P33267 Cy tochrome P450 Cy p2f 2 sp|P33267|CP2F2_MOUSE Cy tochrome P450 2F2 OS=Mus musculus OX=10090 GN=Cy p2f 2 PE=1 SV=1

23.3688 22.37944 22.73018 23.17335 19.72678 21.81511 22.32376 20.76027 biological cell part;cy toplasm;intracellular 3 3 3 21 21 21 17.521 0 6.123 25.76812 3 1 1 1 1 2 2 1 0 1.529029573 0.475698795 -1.756458759 -2.839713 Q5EBG6 Q5EBG6 Heat shock protein Hspb6 sp|Q5EBG6|HSPB6_MOUSE Heat shock protein beta-6 OS=Mus musculus OX=10090 GN=Hspb6 PE=1 SV=1

24.88725 24.65031 24.19873 24.86292 24.18722 23.87549 24.36165 24.02597 aging;anat cell part;c Adherens REACT_2 6 6 6 12 12 12 67.121 0 10.69 27.61288 15 4 3 4 4 3 3 4 1 1.515772363 0.487435435 -0.537220478 -2.816482 Q62312 Q62312 TGF-beta receptor Tgf br2 sp|Q62312|TGFR2_MOUSE TGF-beta receptor ty pe-2 OS=Mus musculus OX=10090 GN=Tgf br2 PE=1 SV=1

22.47259 23.56023 23.48591 21.35138 20.99569 18.72721 20.69472 21.48161 biological cell part;cy toplasm;endoplasmi 3 3 3 35.1 35.1 35.1 15.278 0.007878 1.4816 26.00434 3 2 1 2 1 1 1 1 1 1.515186874 0.486814371 -2.242717743 -2.815458 Q9ER81 Q9ER81 Torsin-1A-interacti Tor1aip2 sp|Q9ER81|IFG15_MOUSE Torsin-1A-interacting protein 2, isof orm IFRG15 OS=Mus musculus OX=10090 GN=Tor1aip2 PE=1 SV=1

28.60378 28.66197 29.04035 28.53438 28.8909 29.17453 29.13521 29.09713 establishmcell part;e Calcium s REACT_3 37 24 20 41.1 31.5 28 134.75 0 101.07 32.21749 141 17 15 19 16 19 18 20 18 1.513016303 0.487797015 0.364321232 2.8116596 G5E829;A0A1W G5E829 Plasma membrane Atp2b1 sp|G5E829|AT2B1_MOUSE Plasma membrane calcium-transporting ATPase 1 OS=Mus musculus OX=10090 GN=Atp2b1 PE=1 SV=1

25.64881 25.67471 24.8768 25.68223 26.82372 26.28884 26.62296 25.69458 biological cell part;cy toplasm;intracellular 10 10 10 24 24 24 60.785 0 27.867 29.37084 38 5 4 3 3 7 8 7 8 1.507103674 0.494166667 0.886889458 2.8013214 A0A2R8VHU8;E A0A2R8V Calcium-binding an Calcoco1 tr|A0A2R8VHU8|A0A2R8VHU8_MOUSE Calcium-binding and coiled-coil domain-containing protein 1 (Fragment) OS=Mus musculus OX=10090 GN=Calcoco1 PE=1 SV=1;tr|E9Q7U2|E9Q7U2_MOUSE Calcium-binding and coiled-coil domain-containing protein 1 OS=Mus musculus OX=10090

26.75774 26.42076 26.46456 26.85555 26.31869 25.94085 26.30661 26.33528 anatomica cell part;centrosome; REACT_2 10 10 7 40.4 40.4 33.4 38.522 0 34.976 29.66756 39 6 6 6 6 7 5 6 5 1.498080125 0.504047478 -0.399292946 -2.785564 Q9CZA6;F6Q32 Q9CZA6 Nuclear distributio Nde1 sp|Q9CZA6|NDE1_MOUSE Nuclear distribution protein nudE homolog 1 OS=Mus musculus OX=10090 GN=Nde1 PE=1 SV=1

29.15904 29.10672 28.70945 29.06381 29.30367 29.46428 29.2135 29.32075 cellular co cell part;macromolec REACT_2 21 21 21 69.4 69.4 69.4 55.971 0 210.34 32.51388 120 19 14 18 14 13 16 17 16 1.483420644 0.522508876 0.315793991 2.760016 Q8BJY 1;F7BA91 Q8BJY 1 26S proteasome n Psmd5 sp|Q8BJY 1|PSMD5_MOUSE 26S proteasome non-ATPase regulatory subunit 5 OS=Mus musculus OX=10090 GN=Psmd5 PE=1 SV=4

24.92025 25.08327 25.03687 24.64927 24.22435 24.51045 24.15399 24.81719 anatomica cell part;extracellular REACT_3 1 1 1 3.4 3.4 3.4 101.16 0 9.4515 27.9678 13 1 1 1 1 1 1 1 1 1.481423103 0.523575221 -0.495921612 -2.75654 Q03146 Q03146 Epithelial discoidin Ddr1 sp|Q03146|DDR1_MOUSE Epithelial discoidin domain-containing receptor 1 OS=Mus musculus OX=10090 GN=Ddr1 PE=2 SV=2

25.37617 25.32392 25.69368 25.63948 25.82663 25.86483 25.99393 25.6437 biological cell part;c SNARE interactions 8 7 7 46.2 43.8 43.8 33.203 0 40.983 29.03981 26 4 3 4 4 5 5 5 4 1.481087005 0.522376471 0.323959351 2.7559548 Q3TJ55;Q80W45 Q3TJ55;Q Sy ntaxin-2 Stx2 tr|Q3TJ55|Q3TJ55_MOUSE Sy ntaxin-2 OS=Mus musculus OX=10090 GN=Stx2 PE=1 SV=1;tr|Q80W45|Q80W45_MOUSE Sy ntaxin-2 OS=Mus musculus OX=10090 GN=Stx2 PE=1 SV=1;sp|Q00262|STX2_MOUSE Sy ntaxin-2 OS=Mus musculus OX=10090 GN=Stx2 PE=1 SV=1

20.35558 20.46128 23.18797 18.84089 22.74974 23.19711 23.60399 23.4665 biological cell part;e Protein processing in 2 2 2 4.7 4.7 4.7 63.869 0 4.5084 26.21191 5 0 0 1 0 1 2 1 1 1.478753615 0.523002933 2.542908192 2.7518956 Q61712;F6ZL86; Q61712;F DnaJ homolog sub Dnajc1 sp|Q61712|DNJC1_MOUSE DnaJ homolog subf amily C member 1 OS=Mus musculus OX=10090 GN=Dnajc1 PE=1 SV=1;tr|F6ZL86|F6ZL86_MOUSE DnaJ homolog subf amily C member 1 (Fragment) OS=Mus musculus OX=10090 GN=Dnajc1 PE=1 SV=1;tr|F6WEH1|F6WEH1_MOUSE DnaJ homolog subf am

25.95283 26.18058 26.54097 26.18956 26.17392 25.23542 25.25061 25.41983 biological cell part;cy toplasm;extracellular 20 10 10 30.6 20.1 20.1 111.84 0 31.219 29.25462 45 5 7 5 4 5 5 5 5 1.47840217 0.521473684 -0.696038246 -2.751284 Q80YR5;D6RHPQ80Y R5 Scaf f old attachme Saf b2 sp|Q80Y R5|SAFB2_MOUSE Scaf f old attachment f actor B2 OS=Mus musculus OX=10090 GN=Saf b2 PE=1 SV=2

21.20373 20.69266 20.2365 21.06271 31.00658 32.39433 24.08318 22.65452 anatomica cell part;cy toplasmic membrane 37 37 37 74.6 74.6 74.6 70.643 0 251.26 33.63526 95 0 0 0 0 29 37 4 2 1.478020549 0.519953353 6.735751629 2.7506207 Q03517;Q4W8U Q03517;Q Secretogranin-2;Se Scg2 sp|Q03517|SCG2_MOUSE Secretogranin-2 OS=Mus musculus OX=10090 GN=Scg2 PE=1 SV=1;tr|Q4W8U9|Q4W8U9_MOUSE Manserin OS=Mus musculus OX=10090 GN=Scg2 PE=1 SV=1

24.75353 24.18563 24.68442 24.62817 25.24978 25.01937 24.83586 24.88674 amine metabolic proc Py ruv ate metabolism 5 5 5 26.9 26.9 26.9 31.49 0 9.5044 28.10154 11 3 3 4 3 3 4 4 3 1.477757175 0.51905814 0.434996128 2.7501627 Q9DB32;D3Y ZU Q9DB32;DHy droxy acy lglutat Haghl sp|Q9DB32|HAGHL_MOUSE Hy droxy acy lglutathione hy drolase-like protein OS=Mus musculus OX=10090 GN=Haghl PE=1 SV=1;tr|D3Y ZU6|D3Y ZU6_MOUSE Hy droxy acy lglutathione hy drolase-like protein OS=Mus musculus OX=10090 GN=Haghl PE=1 SV=1

22.41573 22.59133 24.4883 22.54439 19.90209 21.47896 19.26742 22.18769 biological cell part 2 2 2 3.1 3.1 3.1 89.135 0 5.2754 25.46345 2 2 2 2 2 0 0 0 0 1.474042116 0.521101449 -2.300901413 -2.743704 Q80TN4 Q80TN4 DnaJ homolog sub Dnajc16 sp|Q80TN4|DJC16_MOUSE DnaJ homolog subf amily C member 16 OS=Mus musculus OX=10090 GN=Dnajc16 PE=1 SV=2

28.65687 27.84122 28.24291 28.53092 28.95155 28.74224 28.82745 28.7943 cellular ke cell part;c Pentose and glucuro 15 15 15 66.8 66.8 66.8 35.208 0 105.06 31.91267 101 13 12 11 12 12 14 15 14 1.472543979 0.521676301 0.510902405 2.741101 Q99KP3 Q99KP3 Lambda-cry stallin Cry l1 sp|Q99KP3|CRY L1_MOUSE Lambda-cry stallin homolog OS=Mus musculus OX=10090 GN=Cry l1 PE=1 SV=3

23.55251 24.88586 24.70625 24.38034 25.05875 25.83028 25.91668 24.96243 biological cell part;c RNA degr REACT_2 9 9 9 15.4 15.4 15.4 113.88 0 28.816 28.51326 28 3 6 4 3 4 7 5 4 1.471307811 0.522270893 1.060798645 2.7389534 Q8VHK9 Q8VHK9 ATP-dependent RNDhx36 sp|Q8VHK9|DHX36_MOUSE ATP-dependent DNA/RNA helicase DHX36 OS=Mus musculus OX=10090 GN=Dhx36 PE=1 SV=2

27.64451 27.53275 27.76945 27.67013 28.02818 27.97564 27.85365 27.70668 catabolic cell part;c RNA degr REACT_3 4 4 4 65 65 65 9.1275 0 74.029 31.0717 29 4 4 4 4 4 4 4 4 1.471192887 0.521103448 0.236827374 2.7387538 P62313 P62313 U6 snRNA-associ Lsm6 sp|P62313|LSM6_MOUSE U6 snRNA-associated Sm-like protein LSm6 OS=Mus musculus OX=10090 GN=Lsm6 PE=1 SV=1

20.62388 19.62415 21.09442 21.94903 22.07248 23.96702 21.92528 22.6756 biosy nthe cell part;e One carbon pool by f 4 4 4 20.9 20.9 20.9 37.863 0 5.386 26.24102 4 1 1 2 0 1 2 1 2 1.469450738 0.522578797 1.837224007 2.7357279 P18155 P18155 Bif unctional methy Mthf d2 sp|P18155|MTDC_MOUSE Bif unctional methy lenetetrahy drof olate dehy drogenase/cy clohy drolase, mitochondrial OS=Mus musculus OX=10090 GN=Mthf d2 PE=1 SV=1

20.46172 23.08834 20.67713 19.6939 23.09288 22.70246 23.25523 22.99056 biological cell part;centrosome;cy toplasm 2 2 2 4.4 4.4 4.4 60.642 0 4.4342 25.75791 5 0 1 0 0 1 1 2 2 1.467780562 0.522777143 2.030008316 2.7328279 Q3TVW5;F6Q72 Q3TVW5; Trichoplein keratin Tchp sp|Q3TVW5|TCHP_MOUSE Trichoplein keratin f ilament-binding protein OS=Mus musculus OX=10090 GN=Tchp PE=1 SV=2;tr|F6Q720|F6Q720_MOUSE Trichoplein keratin f ilament-binding protein (Fragment) OS=Mus musculus OX=10090 GN=Tchp PE=1 SV=1

23.90417 24.47655 24.40594 24.45444 24.54887 24.8275 24.68121 24.88171 carbohy dr cell part;e N-Gly can REACT_3 2 2 2 7.1 7.1 7.1 54.417 0 13.828 27.9219 11 2 2 1 2 2 2 2 2 1.466376594 0.522564103 0.424547195 2.7303907 Q921Q3 Q921Q3 Chitobiosy ldiphosp Alg1 sp|Q921Q3|ALG1_MOUSE Chitobiosy ldiphosphodolichol beta-mannosy ltransf erase OS=Mus musculus OX=10090 GN=Alg1 PE=1 SV=3

23.83277 23.64636 23.79956 22.99595 20.91756 20.91872 21.18145 23.66622 2 2 2 19.8 19.8 19.8 32.191 0 3.5283 26.00103 2 2 2 1 1 0 0 0 1 1.464391721 0.523909091 -1.897674561 -2.726946 B1APT1;Q9CZJ0 B1APT1;Q Metallophosphoest Mpped2 tr|B1APT1|B1APT1_MOUSE Metallophosphoesterase MPPED2 (Fragment) OS=Mus musculus OX=10090 GN=Mpped2 PE=1 SV=1;sp|Q9CZJ0|MPPD2_MOUSE Metallophosphoesterase MPPED2 OS=Mus musculus OX=10090 GN=Mpped2 PE=2 SV=1

34.53826 34.54737 34.3442 34.17199 34.63855 34.54927 34.80188 34.83879 behav ior;b cell part;c Aldosteron REACT_3 65 65 43 64.6 64.6 42.6 103.58 0 323.31 37.89301 985 59 61 59 60 64 61 61 62 1.463811546 0.522878187 0.306666374 2.7259394 D3YYN7;Q6PIE D3YYN7; Sodium/potassium Atp1a2 tr|D3YYN7|D3YYN7_MOUSE Sodium/potassium-transporting ATPase subunit alpha OS=Mus musculus OX=10090 GN=Atp1a2 PE=1 SV=1;sp|Q6PIE5|AT1A2_MOUSE Sodium/potassium-transporting ATPase subunit alpha-2 OS=Mus musculus OX=10090 GN=Atp1a2 PE=1 SV=1

24.00753 23.71733 23.46438 23.70049 23.58932 23.32553 23.16037 23.02739 2 2 2 8.7 8.7 8.7 31.593 0.000485 2.771 26.84518 7 1 1 1 1 1 2 1 1 1.459390774 0.527231638 -0.446779251 -2.718272 Q9CZT6 Q9CZT6 Protein CMSS1 Cmss1 sp|Q9CZT6|CMS1_MOUSE Protein CMSS1 OS=Mus musculus OX=10090 GN=Cmss1 PE=2 SV=1

25.68606 26.08343 26.04156 26.03465 26.18745 26.37731 26.20245 26.19661 8 2 0 50.6 12.9 0 28.687 0 11.165 29.39842 23 2 2 2 2 2 2 2 2 1.456314427 0.53063662 0.279533863 2.7129398 A0A1B0GSH8 A0A1B0GSH8 **Gm45808** tr|A0A1B0GSH8|A0A1B0GSH8_MOUSE Predicted gene 45808 (Fragment) OS=Mus musculus OX=10090 GN=Gm45808 PE=4 SV=1

27.38542 27.50877 27.57087 27.44947 27.53668 28.02923 27.8242 27.71967 biological extracellul Protein processing in 18 18 18 34.1 34.1 34.1 87.22 0 67.565 30.95303 96 15 15 12 11 13 17 16 13 1.454875238 0.531202247 0.298811436 2.7104461 P27612;F7D1R5 P27612 Phospholipase A-2 Plaa sp|P27612|PLAP_MOUSE Phospholipase A-2-activ ating protein OS=Mus musculus OX=10090 GN=Plaa PE=1 SV=4

29.79326 30.05827 29.98482 29.65222 29.95712 30.4175 30.23797 30.39734 biological cell part;c Neuroacti REACT_2 40 40 40 46.2 46.2 46.2 138.43 0 179.33 33.44369 220 29 26 23 19 23 34 32 35 1.450590344 0.537221289 0.380339622 2.7030251 Q8CIE6;F8WHL Q8CIE6;F Coatomer subunit Copa sp|Q8CIE6|COPA_MOUSE Coatomer subunit alpha OS=Mus musculus OX=10090 GN=Copa PE=1 SV=2;tr|F8WHL2|F8WHL2_MOUSE Coatomer subunit alpha OS=Mus musculus OX=10090 GN=Copa PE=1 SV=1

28.77812 29.11577 28.93013 28.65075 29.02292 29.32571 29.15617 29.44107 amine me cell part;in Valine, leu REACT_3 22 22 22 64.1 64.1 64.1 61.378 0 174.29 32.34369 165 18 18 19 15 16 17 16 19 1.449714216 0.536346369 0.36777401 2.7015083 Q3ULD5;Q6PD2 Q3ULD5; Methy lcrotonoy l-C Mccc2 sp|Q3ULD5|MCCB_MOUSE Methy lcrotonoy l-CoA carboxy lase beta chain, mitochondrial OS=Mus musculus OX=10090 GN=Mccc2 PE=1 SV=1;tr|Q6PD20|Q6PD20_MOUSE Mccc2 protein (Fragment) OS=Mus musculus OX=10090 GN=Mccc2 PE=1 SV=1

28.30691 28.71434 28.37997 28.29728 28.73331 28.71803 28.90182 28.60038 biological cell body ;cell part;cy toplasmic 17 17 17 44.2 44.2 44.2 67.789 0 87.299 31.87635 100 14 14 12 12 14 14 15 14 1.448139798 0.538038997 0.313760281 2.6987833 Q8C788;Q91ZR Q8C788;Q Sorting nexin;Sorti Snx18 tr|Q8C788|Q8C788_MOUSE Sorting nexin OS=Mus musculus OX=10090 GN=Snx18 PE=1 SV=1;sp|Q91ZR2|SNX18_MOUSE Sorting nexin-18 OS=Mus musculus OX=10090 GN=Snx18 PE=1 SV=1

26.99183 26.90403 26.93236 27.52021 27.84637 27.51382 27.44632 27.43102 biological cell body ; Insulin sig REACT_2 5 5 5 23.4 23.4 23.4 20.451 0 20.686 30.61737 33 3 4 3 3 3 4 4 4 1.447426802 0.537344444 0.472275257 2.6975494 Q921J2 Q921J2 GTP-binding protei Rheb sp|Q921J2|RHEB_MOUSE GTP-binding protein Rheb OS=Mus musculus OX=10090 GN=Rheb PE=1 SV=1

34.21053 34.0692 33.99167 34.34023 34.3901 34.26689 34.50894 34.44289 amine trancell leadin Cell cy cle REACT_2 28 28 23 83.7 83.7 71.8 27.771 0 323.31 37.55185 466 26 26 24 28 24 25 26 26 1.444182729 0.54033241 0.249298096 2.6919371 P63101;A0A2I3B P63101;A 14-3-3 protein zeta Ywhaz sp|P63101|1433Z_MOUSE 14-3-3 protein zeta/delta OS=Mus musculus OX=10090 GN=Ywhaz PE=1 SV=1;tr|A0A2I3BQ03|A0A2I3BQ03_MOUSE 14-3-3 protein zeta/delta (Fragment) OS=Mus musculus OX=10090 GN=Ywhaz PE=1 SV=1

22.28283 22.31777 22.86537 22.0001 22.13057 20.2246 20.45363 21.48239 autophagy cell part;c Regulation of autoph 1 1 1 3.7 3.7 3.7 52.055 0.00275 1.9937 24.47599 1 1 1 1 1 0 0 0 0 1.441289169 0.542950276 -1.293721676 -2.686934 Q811C2 Q811C2 Cy steine protease Atg4c sp|Q811C2|ATG4C_MOUSE Cy steine protease ATG4C OS=Mus musculus OX=10090 GN=Atg4c PE=1 SV=2

22.36212 23.01861 23.21693 22.50074 21.35019 20.14021 22.57881 21.24553 2 2 2 24.1 24.1 24.1 12.835 0.002462 2.0861 25.32876 3 1 2 2 1 0 0 1 0 1.43949761 0.543592287 -1.445913315 -2.683837 P0DP60 P0DP60 **Lynx1** sp|P0DP60|LY NX1_MOUSE Ly -6/neurotoxin-like protein 1 OS=Mus musculus OX=10090 GN=Ly nx1 PE=1 SV=1

27.90426 28.06384 27.96692 27.92649 28.11391 28.06758 28.18248 28.02322 cellular m cell part;c mRNA su REACT_2 12 12 12 51.7 51.7 51.7 48.381 0 85.861 31.26361 88 9 9 7 8 9 10 10 10 1.437562546 0.544549451 0.13142395 2.6804931 Q99LC2;A2APA3 Q99LC2 Cleav age stimulat Cstf 1 sp|Q99LC2|CSTF1_MOUSE Cleav age stimulation f actor subunit 1 OS=Mus musculus OX=10090 GN=Cstf 1 PE=1 SV=1

28.90021 28.95651 29.09494 29.00571 29.20163 29.3869 29.38636 29.02057 cellular lip cell part;Golgi appara REACT_3 25 25 25 49.2 49.2 49.2 66.943 0 157.85 32.45547 119 19 22 19 18 21 20 20 23 1.435737379 0.545764384 0.259520531 2.6773401 Q9EP69;A0A5F8 Q9EP69;A Phosphatidy linosit Sacm1l sp|Q9EP69|SAC1_MOUSE Phosphatidy linositol-3-phosphatase SAC1 OS=Mus musculus OX=10090 GN=Sacm1l PE=1 SV=1;tr|A0A5F8MPK9|A0A5F8MPK9_MOUSE Phosphatidy linositol-3-phosphatase SAC1 OS=Mus musculus OX=10090 GN=Sacm1l PE=1 SV=1

26.5125 26.69032 26.95527 25.98958 26.98479 27.11386 27.03957 27.39803 cell part;cy toplasm;i REACT_2 7 7 7 17.5 17.5 17.5 67.314 0 34.935 30.17997 42 7 7 7 6 3 5 5 4 1.43175655 0.549245902 0.597145557 2.6704663 P61222 P61222 ATP-binding casse Abce1 sp|P61222|ABCE1_MOUSE ATP-binding cassette sub-f amily E member 1 OS=Mus musculus OX=10090 GN=Abce1 PE=1 SV=1

28.21688 28.06507 28.06332 27.87625 27.35733 27.72585 27.76623 27.91182 cell part;endosome;intracellular 20 20 20 47.6 47.6 47.6 67.177 0 86.621 31.17099 80 13 14 13 13 12 18 17 15 1.428210595 0.553340599 -0.365071297 -2.664347 Q80UP5;F7B209 Q80UP5;F Anky rin repeat do Ankrd13a sp|Q80UP5|AN13A_MOUSE Anky rin repeat domain-containing protein 13A OS=Mus musculus OX=10090 GN=Ankrd13a PE=1 SV=2;tr|F7B209|F7B209_MOUSE Anky rin repeat domain-containing protein 13A (Fragment) OS=Mus musculus OX=10090 GN=Ankrd13a PE=1 SV=1

23.88818 24.90917 25.45764 25.50803 26.20927 25.86361 26.16406 25.71984 anatomica cell part;m Af rican try panosomi 8 1 1 44.9 8.8 8.8 15.878 0 4.7194 29.3258 10 1 1 1 1 1 1 1 1 1.425292809 0.554902174 1.048438549 2.6593141 P02089 P02089 Hemoglobin subun Hbb-b2 sp|P02089|HBB2_MOUSE Hemoglobin subunit beta-2 OS=Mus musculus OX=10090 GN=Hbb-b2 PE=1 SV=2

23.22933 23.43097 23.44291 23.80469 23.72088 23.7967 24.23286 23.90298 anatomica cell part;c Axon guid REACT_3 3 3 3 10.8 10.8 10.8 76.691 0 16.347 26.91878 14 2 2 1 2 2 2 2 1 1.421180475 0.558363144 0.436378479 2.6522247 Q9DBQ6;B5B2N Q9DBQ6; Nuclear f actor of Nf atc1 tr|Q9DBQ6|Q9DBQ6_MOUSE Nuclear f actor of activ ated T-cells c1 isof orm IB-VIII OS=Mus musculus OX=10090 GN=Nf atc1 PE=1 SV=1;tr|B5B2N5|B5B2N5_MOUSE Nuclear f actor of activ ated T-cells c1 isof orm IA-VIII OS=Mus musculus OX=10090 GN=Nf atc1 PE=1 SV=1;tr|B5B2N4|

25.97277 25.88232 25.95096 26.29854 26.26496 26.41914 26.4803 26.17331 biological cell part;extracellular REACT_2 8 8 8 19.3 19.3 19.3 62.643 0 7.6879 29.47018 29 6 6 6 7 3 4 4 4 1.418822604 0.560962162 0.308280945 2.6481618 A0A1L1SVG6;Q8 A0A1L1SV Choline transporte Slc44a2 tr|A0A1L1SVG6|A0A1L1SVG6_MOUSE Choline transporter-like protein 2 OS=Mus musculus OX=10090 GN=Slc44a2 PE=1 SV=1;sp|Q8BY 89|CTL2_MOUSE Choline transporter-like protein 2 OS=Mus musculus OX=10090 GN=Slc44a2 PE=1 SV=2

26.40641 25.77497 26.2394 25.50791 25.75855 25.04976 25.24949 25.09736 biological cell part;c Regulation REACT_3 7 5 5 7.6 6.5 6.5 180.53 0 8.7477 28.93239 15 5 2 4 1 1 3 3 3 1.412044098 0.568808625 -0.693383217 -2.63649 Q3UQ44 Q3UQ44 Ras GTPase-activ Iqgap2 sp|Q3UQ44|IQGA2_MOUSE Ras GTPase-activ ating-like protein IQGAP2 OS=Mus musculus OX=10090 GN=Iqgap2 PE=1 SV=2

27.81836 27.39272 27.1037 27.59619 27.89643 27.69869 28.10159 28.19282 cellular m cell part;extracellular organelle; 13 12 12 30.7 28.6 28.6 62.63 0 47.14 31.08151 64 9 9 7 9 10 11 11 11 1.410650859 0.569139785 0.494639874 2.6340924 Q9JLB0;E9PWC Q9JLB0 MAGUK p55 subf a Mpp6 sp|Q9JLB0|MPP6_MOUSE MAGUK p55 subf amily member 6 OS=Mus musculus OX=10090 GN=Mpp6 PE=1 SV=1

32.96013 32.84987 32.74707 32.70642 33.38934 33.13441 32.92788 33.02062 alcohol m cell part;c Gly coly si REACT_3 17 17 15 69.7 69.7 61.4 28.832 0 323.31 36.21796 295 16 16 15 15 16 16 15 16 1.409367156 0.569297587 0.302189827 2.6318837 Q9DBJ1 Q9DBJ1 Phosphogly cerate Pgam1 sp|Q9DBJ1|PGAM1_MOUSE Phosphogly cerate mutase 1 OS=Mus musculus OX=10090 GN=Pgam1 PE=1 SV=3

23.73231 23.97334 23.58302 23.80577 24.12888 24.14717 23.89883 24.52636 cell part;endoplasmic reticulum; 1 1 1 4.5 4.5 4.5 27.283 0.000637 2.583 27.2683 2 1 1 1 1 1 1 1 1 1.403824136 0.575860963 0.401697159 2.6223518 Q8C407 Q8C407 Protein YIPF4 Yipf 4 sp|Q8C407|Y IPF4_MOUSE Protein YIPF4 OS=Mus musculus OX=10090 GN=Yipf 4 PE=1 SV=1

23.28122 23.25566 23.83836 23.52313 24.19189 23.83326 24.34218 23.67874 autophagy cell part;intracellular organelle;m 2 2 2 7.7 7.7 7.7 47.339 0 7.3196 27.25665 15 1 1 1 1 1 2 2 2 1.401807567 0.576565333 0.536925793 2.618886 D6RGM8;D3YW D6RGM8; Transmembrane 9 Tm9sf 1 tr|D6RGM8|D6RGM8_MOUSE Transmembrane 9 superf amily member OS=Mus musculus OX=10090 GN=Tm9sf 1 PE=1 SV=1;tr|D3YWH4|D3Y WH4_MOUSE Transmembrane 9 superf amily member (Fragment) OS=Mus musculus OX=10090 GN=Tm9sf 1 PE=1 SV=1;tr|D3Z6X7|D3Z6X7_MOUSE Transmembrane 9

26.1388 26.65963 26.34294 26.15908 26.43668 26.83762 26.84745 26.81127 autophagy cell part;cy toplasmic membrane 8 8 8 35 35 35 27.328 0 21.585 29.90096 27 6 7 5 6 5 7 7 5 1.399161001 0.578457447 0.408143997 2.614339 P35283;A2CG35 P35283;A Ras-related protein Rab12 sp|P35283|RAB12_MOUSE Ras-related protein Rab-12 OS=Mus musculus OX=10090 GN=Rab12 PE=1 SV=3;tr|A2CG35|A2CG35_MOUSE Ras-related protein Rab-12 OS=Mus musculus OX=10090 GN=Rab12 PE=1 SV=1

24.74196 24.68715 25.32759 24.7383 24.43447 24.0296 24.5402 24.4932 biological cell part;intracellular membrane- 4 4 4 13 13 13 47.346 0 6.8285 27.92071 13 3 3 3 2 2 2 2 2 1.394396235 0.583119363 -0.49938345 -2.606157 Q9JJA4;D3Z369 Q9JJA4 Ribosome biogene Wdr12 sp|Q9JJA4|WDR12_MOUSE Ribosome biogenesis protein WDR12 OS=Mus musculus OX=10090 GN=Wdr12 PE=1 SV=1

29.02871 29.27048 29.35651 29.32068 29.5185 29.65214 29.43839 29.36643 anatomica cell body ; Ether lipid REACT_2 17 17 17 55.6 55.6 55.6 46.67 0 112.77 32.65606 158 14 15 14 13 11 14 12 13 1.391998246 0.585185185 0.249768257 2.6020417 P63005;Q5SW16 P63005 Platelet-activ ating Paf ah1b1 sp|P63005|LIS1_MOUSE Platelet-activ ating f actor acety lhy drolase IB subunit beta OS=Mus musculus OX=10090 GN=Paf ah1b1 PE=1 SV=2

25.68974 25.29586 25.69663 24.46076 25.77876 26.75226 26.01521 26.30803 cellular pr cell part;cy toplasm;Golgi appar 9 9 9 30.7 30.7 30.7 82.463 0 39.014 29.22952 23 5 3 3 2 6 6 7 4 1.384833614 0.59391029 0.927817822 2.5897539 R4H4V1;A0A494 R4H4V1;A N-terminal kinase-l Scy l1 tr|R4H4V1|R4H4V1_MOUSE N-terminal kinase-like protein OS=Mus musculus OX=10090 GN=Scy l1 PE=1 SV=1;tr|A0A494BBD3|A0A494BBD3_MOUSE N-terminal kinase-like protein (Fragment) OS=Mus musculus OX=10090 GN=Scy l1 PE=1 SV=1;tr|R4H4Y 7|R4H4Y 7_MOUSE N-terminal kinase-

22.40707 22.52566 22.81084 21.78405 22.93276 23.98181 22.81241 23.33265 cell part;membrane 2 2 2 5.7 5.7 5.7 41.99 0.004222 1.8434 26.35026 7 1 1 1 1 2 2 1 2 1.38413472 0.594410526 0.883002758 2.5885559 Q6NSU3 Q6NSU3 Gly cosy ltransf era Glt8d1 sp|Q6NSU3|GL8D1_MOUSE Gly cosy ltransf erase 8 domain-containing protein 1 OS=Mus musculus OX=10090 GN=Glt8d1 PE=1 SV=1

25.90139 25.49723 25.87659 25.48781 25.74348 26.57055 26.35668 26.25039 alcohol m extracellul Amino sugar and nuc 8 8 8 28.8 28.8 28.8 41.984 0 21.693 29.39828 31 5 4 4 3 6 6 7 6 1.376381967 0.603811024 0.539520264 2.5752749 Q8K0C9;J3QMC Q8K0C9 GDP-mannose 4,6 Gmds sp|Q8K0C9|GMDS_MOUSE GDP-mannose 4,6 dehy dratase OS=Mus musculus OX=10090 GN=Gmds PE=1 SV=1

23.39493 20.43313 23.06558 20.97326 23.83132 24.38469 23.38732 24.22971 biological cell part;cy toplasm;mREACT_2 5 5 5 9.7 9.7 9.7 93.092 0 20.219 26.7103 13 1 1 1 2 1 2 2 4 1.375621729 0.603225131 1.991538048 2.5739734 Q91WG4 Q91WG4 Elongator complex Elp2 sp|Q91WG4|ELP2_MOUSE Elongator complex protein 2 OS=Mus musculus OX=10090 GN=Elp2 PE=1 SV=1

24.59912 24.16285 23.96446 24.65125 25.29992 25.27352 24.72946 24.60416 anatomica cell part;c Bladder ca REACT_2 4 3 3 14.9 12.2 12.2 33.75 0 7.2557 28.01654 18 2 2 2 2 3 3 3 3 1.37088253 0.608344648 0.632347584 2.5658628 P30285;E9Q9E4 P30285;E Cy clin-dependent Cdk4 sp|P30285|CDK4_MOUSE Cy clin-dependent kinase 4 OS=Mus musculus OX=10090 GN=Cdk4 PE=1 SV=1;tr|E9Q9E4|E9Q9E4_MOUSE Cy clin-dependent kinase 4 (Fragment) OS=Mus musculus OX=10090 GN=Cdk4 PE=1 SV=1;tr|E9PZX7|E9PZX7_MOUSE Cy clin-dependent kinase 4 (Fragment) OS=

25.75491 25.41761 26.07331 25.66433 25.38479 25.41938 25.41596 25.142 cell part;endosome;intracellular 5 5 5 42.8 42.8 42.8 19.742 0 14.576 28.81733 13 3 2 5 3 3 3 3 4 1.369101265 0.60884375 -0.387005329 -2.562816 Q8VE99 Q8VE99 Coiled-coil domain Ccdc115 sp|Q8VE99|CC115_MOUSE Coiled-coil domain-containing protein 115 OS=Mus musculus OX=10090 GN=Ccdc115 PE=1 SV=1

22.65877 22.64786 26.19599 22.81384 21.7185 21.33465 19.30855 21.3868 behav ior;b cell part;c Calcium s REACT_2 6 6 6 16 16 16 52.542 0 8.2575 26.95826 13 3 3 6 2 1 0 1 1 1.368037966 0.608051948 -2.641989708 -2.560997 Q8BGR3;P08414 Q8BGR3; Calcium/calmoduli Camk4 tr|Q8BGR3|Q8BGR3_MOUSE Calcium/calmodulin-dependent protein kinase IV OS=Mus musculus OX=10090 GN=Camk4 PE=1 SV=1;sp|P08414|KCC4_MOUSE Calcium/calmodulin-dependent protein kinase ty pe IV OS=Mus musculus OX=10090 GN=Camk4 PE=1 SV=2

28.65973 28.51235 28.184 28.29798 28.81029 28.76847 28.71751 28.57232 cell part;cy toplasm;intracellular 15 15 15 54.8 54.8 54.8 55.939 0 136.65 31.83853 104 13 13 11 8 13 13 14 14 1.367943557 0.606476684 0.303634644 2.5608358 Q6NSR8;F6T2H Q6NSR8 Probable aminopep Npepl1 sp|Q6NSR8|PEPL1_MOUSE Probable aminopeptidase NPEPL1 OS=Mus musculus OX=10090 GN=Npepl1 PE=1 SV=1

22.23196 23.586 23.85215 23.89698 20.50847 23.32882 18.90937 20.62576 biological cell part;cell projection;cy toplas 3 2 2 15.6 12.4 12.4 42.178 0 4.8892 25.80065 5 1 2 1 1 0 1 0 0 1.365354706 0.608981912 -2.548666477 -2.556409 Q8BHE3 Q8BHE3 Cay taxin Atcay sp|Q8BHE3|ATCAY _MOUSE Cay taxin OS=Mus musculus OX=10090 GN=Atcay PE=1 SV=1

25.32193 25.52937 25.08042 25.3807 25.46236 25.78497 25.76731 25.55213 alcohol m cell part;in Pentose phosphate p 6 6 6 11.2 11.2 11.2 89.909 0 13.152 28.76402 27 3 3 3 3 3 4 6 4 1.362941728 0.611206186 0.313586712 2.5522851 A2A7A7;Q8CFX1 A2A7A7;Q GDH/6PGL endopl H6pd tr|A2A7A7|A2A7A7_MOUSE GDH/6PGL endoplasmic bif unctional protein OS=Mus musculus OX=10090 GN=H6pd PE=1 SV=1;sp|Q8CFX1|G6PE_MOUSE GDH/6PGL endoplasmic bif unctional protein OS=Mus musculus OX=10090 GN=H6pd PE=1 SV=2

23.79154 24.09192 24.50736 23.65042 19.12853 24.18662 20.43717 21.20402 cellular m cell part;cy toplasm;intracellular 3 3 3 15.1 15.1 15.1 33.542 0 4.147 26.70846 6 1 1 2 1 0 1 2 0 1.359224415 0.615167095 -2.771230221 -2.545934 Q3TGY 1;Q9DBU Q3TGY 1; Serine/Arginine-rel Rsrc1 tr|Q3TGY 1|Q3TGY 1_MOUSE Serine/Arginine-related protein 53 OS=Mus musculus OX=10090 GN=Rsrc1 PE=1 SV=1;sp|Q9DBU6|RSRC1_MOUSE Serine/Arginine-related protein 53 OS=Mus musculus OX=10090 GN=Rsrc1 PE=1 SV=1;tr|Q8BR75|Q8BR75_MOUSE Serine/Arginine-related protei

26.08434 26.78868 26.80772 26.35176 26.96741 26.85436 27.12741 26.964 biosy nthe cell part;e Gly cerolip REACT_3 7 7 7 21.8 21.8 21.8 43.295 0 19.531 30.12349 28 4 4 4 2 4 5 6 5 1.357105363 0.615794872 0.470170498 2.5423151 Q9D517 Q9D517 1-acy l-sn-gly cerol- Agpat3 sp|Q9D517|PLCC_MOUSE 1-acy l-sn-gly cerol-3-phosphate acy ltransf erase gamma OS=Mus musculus OX=10090 GN=Agpat3 PE=1 SV=2

26.84745 26.8846 27.26383 27.06034 26.4112 26.88914 26.76724 26.46072 cell part;cy tosol 12 12 12 46 46 46 38.725 0 59.71 30.13822 54 8 7 9 8 8 11 9 10 1.35533334 0.617452685 -0.381979465 -2.53929 Q921W4;D3Z6I4 Q921W4; Quinone oxidoredu Cry zl1 sp|Q921W4|QORL1_MOUSE Quinone oxidoreductase-like protein 1 OS=Mus musculus OX=10090 GN=Cry zl1 PE=1 SV=1;tr|D3Z6I4|D3Z6I4_MOUSE Quinone oxidoreductase-like protein 1 OS=Mus musculus OX=10090 GN=Cry zl1 PE=1 SV=1;tr|D3Y ZD6|D3Y ZD6_MOUSE Quinone oxidoreductase

23.6174 24.1467 24.48505 23.72858 22.95631 22.88388 23.28842 23.84498 cellular pr cell part;cy toplasm;Golgi appar 4 4 4 16.7 16.7 16.7 42.481 0 12.758 26.9577 12 1 2 3 1 1 3 3 2 1.353495333 0.618581633 -0.75103426 -2.536152 Q9CR89;A0A0N Q9CR89; Endoplasmic reticu Ergic2 sp|Q9CR89|ERGI2_MOUSE Endoplasmic reticulum-Golgi intermediate compartment protein 2 OS=Mus musculus OX=10090 GN=Ergic2 PE=1 SV=1;tr|A0A0N4SVQ6|A0A0N4SVQ6_MOUSE Endoplasmic reticulum-Golgi intermediate compartment protein 2 (Fragment) OS=Mus musculus OX=10

26.73053 26.85614 27.1048 27.08142 27.10969 27.29255 27.12228 27.35976 alcohol m cell part;e Other ty p REACT_2 9 9 9 37.9 37.9 37.9 44.688 0 18.402 30.41609 34 7 6 7 6 6 8 8 6 1.35088195 0.622147583 0.277848244 2.5316927 Q91ZW2;A2AMC Q91ZW2; GDP-f ucose prote Pof ut1 sp|Q91ZW2|OFUT1_MOUSE GDP-f ucose protein O-f ucosy ltransf erase 1 OS=Mus musculus OX=10090 GN=Pof ut1 PE=1 SV=1;tr|A2AMC3|A2AMC3_MOUSE GDP-f ucose protein O-f ucosy ltransf erase 1 OS=Mus musculus OX=10090 GN=Pof ut1 PE=1 SV=1;tr|Q3UXG7|Q3UXG7_MOUSE GDP-f ucose pro

24.90228 25.42499 24.99205 25.33756 25.48265 25.5583 25.65796 25.37901 biological cell part;cell projectio REACT_2 10 10 10 11.4 11.4 11.4 151.86 0 36.904 28.72537 36 6 6 6 4 3 7 6 7 1.345331639 0.629167513 0.35526228 2.5222266 Q6EDY 6;D3Z03 Q6EDY 6; Leucine-rich repea Lrrc16a sp|Q6EDY 6|CARL1_MOUSE F-actin-uncapping protein LRRC16A OS=Mus musculus OX=10090 GN=Carmil1 PE=1 SV=2;tr|D3Z030|D3Z030_MOUSE F-actin-uncapping protein LRRC16A OS=Mus musculus OX=10090 GN=Carmil1 PE=1 SV=2;tr|F7AI27|F7AI27_MOUSE F-actin-uncapping protein LR

21.36634 22.51109 22.2854 21.8717 20.73137 21.79389 21.18794 20.71444 cellular metabolic process;cellular nitrogen 2 2 2 5.2 5.2 5.2 45.281 0.005925 1.6574 24.14826 2 1 1 1 1 0 1 0 0 1.345257285 0.627686076 -0.901720047 -2.5221 Q9DCL4 Q9DCL4 Probable methy ltra Mettl15 sp|Q9DCL4|MET15_MOUSE 12S rRNA N4-methy lcy tidine methy ltransf erase OS=Mus musculus OX=10090 GN=Mettl15 PE=1 SV=2

30.90512 30.79877 30.51521 31.09169 30.94984 31.22642 31.32077 31.30818 anatomica cell part;cell projectio REACT_2 29 29 1 60.4 60.4 1.6 80.646 0 288.15 34.3102 249 27 25 21 24 24 26 26 28 1.344092026 0.627686869 0.37360239 2.5201133 Q9QYC0;F8WH Q9QY C0; Alpha-adducin Add1 sp|Q9QY C0|ADDA_MOUSE Alpha-adducin OS=Mus musculus OX=10090 GN=Add1 PE=1 SV=2;tr|F8WHZ9|F8WHZ9_MOUSE Alpha-adducin OS=Mus musculus OX=10090 GN=Add1 PE=1 SV=1;tr|F8WGR0|F8WGR0_MOUSE Alpha-adducin OS=Mus musculus OX=10090 GN=Add1 PE=1 SV=1

24.78447 24.6962 20.42109 21.85863 25.96362 28.59256 24.73009 26.07545 catabolic cell part;c Drug metabolism - ot 11 11 10 27.6 27.6 26 61.787 0 52.648 29.79332 25 1 1 0 1 7 11 7 7 1.34302891 0.628241814 3.400333881 2.5183013 Q8VCT4;E9PY P Q8VCT4 Carboxy lesterase Ces1d sp|Q8VCT4|EST1D_MOUSE Carboxy lesterase 1D OS=Mus musculus OX=10090 GN=Ces1d PE=1 SV=1

28.42374 28.50032 28.84718 28.48696 28.90377 28.8087 28.94246 28.70491 biological cell part;cy tosol;intracellular me 4 4 4 96.7 96.7 96.7 9.6418 0 51.26 32.16129 68 3 3 3 3 2 3 3 4 1.342389235 0.627306533 0.275407791 2.5172111 Q9D1L9;G3UW7 Q9D1L9;G Ragulator complex Lamtor5 sp|Q9D1L9|LTOR5_MOUSE Ragulator complex protein LAMTOR5 OS=Mus musculus OX=10090 GN=Lamtor5 PE=1 SV=1;tr|G3UW70|G3UW70_MOUSE Late endosomal/ly sosomal adaptor and MAPK and MTOR activ ator 5 OS=Mus musculus OX=10090 GN=Lamtor5 PE=1 SV=1

26.73402 26.76585 26.3129 27.02506 27.08891 27.06219 27.1991 27.01744 9 9 7 32 32 26.7 53.987 0 47.569 30.13406 47 6 5 3 7 7 8 7 7 1.338237576 0.633874687 0.382452488 2.5101377 A0A571BEI2;Q6 A0A571BE Uncharacterized protein FLJ4 tr|A0A571BEI2|A0A571BEI2_MOUSE AP2-associated protein kinase 1 (Fragment) OS=Mus musculus OX=10090 GN=Aak1 PE=1 SV=1;sp|Q6PIU9|Y J005_MOUSE Uncharacterized protein FLJ45252 homolog OS=Mus musculus OX=10090 PE=1 SV=2

29.19336 29.26101 28.97624 29.16094 29.26832 29.59509 29.58003 29.26327 cell part;cy tosol 29 29 29 48.7 48.7 48.7 96.763 0 136.43 32.61535 147 21 20 19 20 19 25 24 24 1.337603885 0.63287 0.278793812 2.5090584 A0A0R4J007;P7 A0A0R4J0 Paladin Pald1 tr|A0A0R4J007|A0A0R4J007_MOUSE Paladin OS=Mus musculus OX=10090 GN=Pald1 PE=1 SV=1;sp|P70261|PALD_MOUSE Paladin OS=Mus musculus OX=10090 GN=Pald1 PE=1 SV=1

24.74899 20.23633 20.92159 22.12145 24.98246 24.16246 24.48025 24.50609 19 4 4 19.1 5.2 5.2 147.16 0 9.0295 27.33577 7 1 0 0 1 3 4 4 2 1.335921341 0.634503741 2.525729179 2.5061931 A0A286Y D76;Q6 A0A286Y D76 **Macf1** tr|A0A286Y D76|A0A286Y D76_MOUSE Microtubule-actin cross-linking f actor 1 (Fragment) OS=Mus musculus OX=10090 GN=Macf 1 PE=1 SV=1

27.0871 26.75239 27.02464 26.74817 26.35907 26.69352 26.67909 26.67438 biological cell part;e MAPK signaling path 3 3 3 50 50 50 13.553 0 11.886 29.99994 12 3 3 2 3 2 2 3 3 1.335161007 0.634129353 -0.301556587 -2.504898 O88653;A0A0G2 O88653 Ragulator complex Lamtor3 sp|O88653|LTOR3_MOUSE Ragulator complex protein LAMTOR3 OS=Mus musculus OX=10090 GN=Lamtor3 PE=1 SV=1

21.46653 21.9819 22.90228 22.10393 20.75804 21.98852 20.54408 19.96784 biological cell part;c Ly sine de REACT_2 3 3 3 2.3 2.3 2.3 186.06 0 3.1195 25.75259 4 1 1 1 1 0 1 2 1 1.334637695 0.633042184 -1.299034595 -2.504007 E9PY H6;A0A0U E9PY H6; Histone-ly sine N-mSetd1a sp|E9PY H6|SET1A_MOUSE Histone-ly sine N-methy ltransf erase SETD1A OS=Mus musculus OX=10090 GN=Setd1a PE=1 SV=1;tr|A0A0U1RNP2|A0A0U1RNP2_MOUSE Predicted gene, 49388 (Fragment) OS=Mus musculus OX=10090 GN=Gm49388 PE=4 SV=1

25.82221 26.13128 26.97432 27.28003 27.07316 27.60343 27.46669 27.95394 amine me cell part;in Aminoacy l-tRNA bio 8 8 8 17.7 17.7 17.7 101.48 0 10.761 30.52645 21 3 3 3 2 4 5 4 5 1.33211723 0.635693069 0.972342491 2.4997171 Q8VDC0;A0A1L Q8VDC0; Probable leucine--t Lars2 sp|Q8VDC0|SY LM_MOUSE Probable leucine--tRNA ligase, mitochondrial OS=Mus musculus OX=10090 GN=Lars2 PE=1 SV=1;tr|A0A1L1SSW1|A0A1L1SSW1_MOUSE Leucine--tRNA ligase OS=Mus musculus OX=10090 GN=Lars2 PE=1 SV=1

24.13985 23.51075 23.62221 24.4062 24.23191 24.64977 24.55221 24.55462 biological cell part;macromolec REACT_3 6 6 6 14.2 14.2 14.2 57.534 0 6.2982 27.5323 13 3 3 2 2 3 3 3 3 1.33135222 0.634676543 0.577374935 2.4984152 Q9WUB0;F6VII1 Q9WUB0 RanBP-ty pe and C Rbck1 sp|Q9WUB0|HOIL1_MOUSE RanBP-ty pe and C3HC4-ty pe zinc f inger-containing protein 1 OS=Mus musculus OX=10090 GN=Rbck1 PE=1 SV=2

22.34259 22.72713 20.99549 20.76489 22.90128 22.90288 22.96541 22.91911 alcohol m cell part;c Amino sug REACT_3 2 2 2 4.8 4.8 4.8 82.889 0 8.3451 25.64149 4 1 1 0 0 2 1 1 1 1.330103516 0.634541872 1.214644909 2.4962903 Q3UW64;Q91W Q3UW64; Bif unctional UDP- Gne tr|Q3UW64|Q3UW64_MOUSE Bif unctional UDP-N-acety lglucosamine 2-epimerase/N-acety lmannosamine kinase OS=Mus musculus OX=10090 GN=Gne PE=1 SV=1;sp|Q91WG8|GLCNE_MOUSE Bif unctional UDP-N-acety lglucosamine 2-epimerase/N-acety lmannosamine kinase OS=Mus musculus O

20.32889 18.47227 23.32127 19.92693 24.07259 26.10372 23.04168 22.10482 carbohy dr cell part REACT_2 6 6 6 14.1 14.1 14.1 69.062 0 25.72 27.61337 10 0 0 1 0 3 6 2 2 1.328388368 0.63431941 3.318362236 2.4933723 Q3UHK1 Q3UHK1 Proton my o-inosito Slc2a13 sp|Q3UHK1|MY CT_MOUSE Proton my o-inositol cotransporter OS=Mus musculus OX=10090 GN=Slc2a13 PE=1 SV=2

29.22073 29.18111 29.32021 29.07189 29.36089 29.47265 29.35915 29.26468 cell part;cy toplasm;extracellular 10 10 0 40.4 40.4 0 32.717 0 33.976 32.57062 69 7 7 6 7 7 6 7 8 1.32503157 0.63822549 0.165857792 2.4876632 A0A3B2W864;A0 A0A3B2W864;A0A338P731;Q Rps10 tr|A0A3B2W864|A0A3B2W864_MOUSE Predicted gene, 49804 OS=Mus musculus OX=10090 GN=Gm49804 PE=3 SV=1;tr|A0A338P731|A0A338P731_MOUSE 40S ribosomal protein S10 (Fragment) OS=Mus musculus OX=10090 GN=Rps10 PE=1 SV=1;tr|Q3UW83|Q3UW83_MOUSE 40S ribosomal protein

22.33103 23.85262 23.38535 23.84862 23.89966 24.65108 24.91187 24.17691 biological cell part;intracellular membrane- 2 2 2 8 8 8 29.524 0.005787 1.6789 27.50628 7 1 1 1 1 2 2 1 2 1.323241531 0.640146699 1.055476189 2.4846198 Q9EST4 Q9EST4 Proteasome assemPsmg2 sp|Q9EST4|PSMG2_MOUSE Proteasome assembly chaperone 2 OS=Mus musculus OX=10090 GN=Psmg2 PE=1 SV=1

20.33381 23.82435 21.45041 19.53547 23.89828 23.68185 23.57381 23.32814 cell part;cy toplasm 3 3 3 8.1 8.1 8.1 40.693 0.000165 3.0506 26.64389 5 0 1 0 0 1 3 3 2 1.322138961 0.6408 2.334510803 2.4827455 P59913;S4R2K3 P59913;S Protein-L-isoaspart Pcmtd1 sp|P59913|PCMD1_MOUSE Protein-L-isoaspartate O-methy ltransf erase domain-containing protein 1 OS=Mus musculus OX=10090 GN=Pcmtd1 PE=1 SV=1;tr|S4R2K3|S4R2K3_MOUSE Protein-L-isoaspartate O-methy ltransf erase domain-containing protein 1 (Fragment) OS=Mus muscul

27.78812 27.96928 28.20037 27.58778 27.96146 28.47445 28.49971 28.41534 anatomica cell part;cy toplasm;endoplasmi 15 15 14 29 29 29 75.127 0 59.583 31.4886 75 7 8 7 6 10 13 13 13 1.321755123 0.639941606 0.451355457 2.4820931 Q8CBM2;A2AL8 Q8CBM2; Asparty l/asparagin Asph tr|Q8CBM2|Q8CBM2_MOUSE Asparty l/asparaginy l beta-hy droxy lase OS=Mus musculus OX=10090 GN=Asph PE=1 SV=1;tr|A2AL85|A2AL85_MOUSE Asparty l/asparaginy l beta-hy droxy lase OS=Mus musculus OX=10090 GN=Asph PE=1 SV=1;sp|Q8BSY 0|ASPH_MOUSE Asparty l/asparaginy l beta-h

31.88121 31.85645 31.93438 31.92917 31.88834 31.6311 31.5845 31.78121 anatomica cell part;cell surf ace;cy toplasm 17 17 17 78.7 78.7 78.7 29.82 0 118.29 35.00926 139 12 13 15 16 14 14 13 13 1.319387444 0.641242718 -0.179017067 -2.478069 P67778;Q5SQG5 P67778;Q Prohibitin Phb sp|P67778|PHB_MOUSE Prohibitin OS=Mus musculus OX=10090 GN=Phb PE=1 SV=1;tr|Q5SQG5|Q5SQG5_MOUSE Prohibitin (Fragment) OS=Mus musculus OX=10090 GN=Phb PE=1 SV=1

24.46026 23.96358 24.06924 25.35138 23.97841 23.60975 23.51256 23.36252 cellular loc cell part;cy tosol;macromolecula 4 4 4 17.4 17.4 17.4 36.525 0 7.8135 27.43992 13 1 2 3 1 2 3 3 3 1.317458466 0.642343826 -0.845304489 -2.474792 Q9D1H7;D3Z4J Q9D1H7; Golgi to ER traf f ic Get4 sp|Q9D1H7|GET4_MOUSE Golgi to ER traf f ic protein 4 homolog OS=Mus musculus OX=10090 GN=Get4 PE=1 SV=2;tr|D3Z4J5|D3Z4J5_MOUSE Golgi to ER traf f ic protein 4 homolog (Fragment) OS=Mus musculus OX=10090 GN=Get4 PE=1 SV=1;tr|D3Z7S0|D3Z7S0_MOUSE Golgi to ER traf

26.52999 26.01933 26.33071 26.47195 26.60894 26.69803 27.04229 26.56145 cell death; cell part;c Ubiquitin mediated pr 10 10 10 34 34 34 38.368 0 27.848 29.86498 36 6 7 6 6 9 8 9 7 1.316298151 0.642241546 0.389680386 2.4728211 Q3UE37 Q3UE37 Ubiquitin-conjugati Ube2z sp|Q3UE37|UBE2Z_MOUSE Ubiquitin-conjugating enzy me E2 Z OS=Mus musculus OX=10090 GN=Ube2z PE=1 SV=2

20.97589 20.62845 20.97127 21.04427 21.07832 21.75381 21.20006 21.31335 biological cell part;centrosome;cy toplasm 1 1 1 13.7 13.7 13.7 10.478 0.004221 1.8423 24.40139 5 1 1 1 1 0 1 1 1 1.31629534 0.640693976 0.431414604 2.4728163 D3Z4D1;O88824 D3Z4D1;OProtein JTB Jtb tr|D3Z4D1|D3Z4D1_MOUSE Protein JTB OS=Mus musculus OX=10090 GN=Jtb PE=1 SV=1;sp|O88824|JTB_MOUSE Protein JTB OS=Mus musculus OX=10090 GN=Jtb PE=1 SV=1

27.75302 27.70807 27.66844 27.62805 27.67189 27.90018 27.91525 27.94754 anatomica cell cortex Acute my REACT_2 9 9 8 37 37 31.5 44.302 0 128.66 31.14017 65 7 7 8 5 7 7 7 9 1.315669108 0.639951923 0.169317245 2.4717527 Q91Y S7;Q63932 Q91Y S7;QDual specif icity m Map2k2 tr|Q91Y S7|Q91Y S7_MOUSE Dual-specif icity mitogen-activ ated protein kinase kinase 2 OS=Mus musculus OX=10090 GN=Map2k2 PE=1 SV=1;sp|Q63932|MP2K2_MOUSE Dual specif icity mitogen-activ ated protein kinase kinase 2 OS=Mus musculus OX=10090 GN=Map2k2 PE=1 SV=2

25.48222 25.06361 24.77243 24.99213 25.63846 25.31251 25.40077 25.64727 cellular lip cell part;in Fructose REACT_2 3 1 1 8.5 3.2 3.2 35.988 0.000635 2.5257 28.54735 4 1 1 1 1 1 1 1 1 1.315304537 0.638800959 0.422150612 2.4711335 P21300 P21300 Aldose reductase-r Akr1b7 sp|P21300|ALD1_MOUSE Aldo-keto reductase f amily 1 member B7 OS=Mus musculus OX=10090 GN=Akr1b7 PE=1 SV=4

25.83314 26.60017 26.38593 26.24505 26.62408 26.96356 26.57816 26.70899 biological cell part;cy toplasm;macromolec 15 15 15 18.2 18.2 18.2 154.64 0 25.916 29.9776 44 8 7 7 5 4 10 10 10 1.315268062 0.637406699 0.452627182 2.4710715 E9QKE4;A0A0A6 E9QKE4;A Rab3 GTPase-acti Rab3gap2 tr|E9QKE4|E9QKE4_MOUSE Rab3 GTPase-activ ating protein non-cataly tic subunit OS=Mus musculus OX=10090 GN=Rab3gap2 PE=1 SV=1;tr|A0A0A6Y WM5|A0A0A6Y WM5_MOUSE Rab3 GTPase-activ ating protein non-cataly tic subunit OS=Mus musculus OX=10090 GN=Rab3gap2 PE=1 SV=1;sp

19.37001 24.96429 21.30085 20.64528 24.81905 24.35394 24.13406 24.97294 cell motilit cell part;cy toskeleton;intracellul 2 2 2 2.5 2.5 2.5 88.506 0.004508 1.8035 27.37435 5 0 1 0 0 1 1 2 1 1.312636751 0.64021957 2.999891281 2.4666035 Q3USS3 Q3USS3 Dy nein regulatory Drc1 sp|Q3USS3|DRC1_MOUSE Dy nein regulatory complex protein 1 OS=Mus musculus OX=10090 GN=Drc1 PE=1 SV=1

21.78273 21.92817 22.16171 23.27085 20.49214 21.89219 21.40732 20.04673 anatomica cell part;e ECM-rece REACT_2 3 3 3 1 1 1 469.78 0 3.984 24.95934 3 1 1 1 1 1 0 0 1 1.308596548 0.645819048 -1.326270103 -2.459746 E9PZ16;B1B0C7 E9PZ16;B Basement membra Hspg2 tr|E9PZ16|E9PZ16_MOUSE Basement membrane-specif ic heparan sulf ate proteogly can core protein OS=Mus musculus OX=10090 GN=Hspg2 PE=1 SV=1;tr|B1B0C7|B1B0C7_MOUSE Basement membrane-specif ic heparan sulf ate proteogly can core protein OS=Mus musculus OX=10090 GN=

24.51971 24.53285 24.3432 25.30288 25.8715 25.8569 25.39784 24.80651 biological cell part;chromosome;intracellul 5 5 5 31 31 31 23.01 0 7.9146 28.42354 19 3 3 3 5 4 2 4 4 1.304298494 0.650821853 0.808526993 2.4524543 O54879;A2AP78 O54879;A High mobility grou Hmgb3 sp|O54879|HMGB3_MOUSE High mobility group protein B3 OS=Mus musculus OX=10090 GN=Hmgb3 PE=1 SV=3;tr|A2AP78|A2AP78_MOUSE High mobility group protein B3 (Fragment) OS=Mus musculus OX=10090 GN=Hmgb3 PE=1 SV=1

29.40393 29.71435 29.94768 29.50027 29.30424 29.16197 29.51286 29.09542 metabolic cell part;in Alzheimer' REACT_2 3 3 3 50 50 50 9.3308 0 12.21 32.65908 55 3 3 3 3 3 3 3 3 1.303661023 0.649781991 -0.372934818 -2.451373 Q9CQ91;D3Z67 Q9CQ91; NADH dehy drogen Nduf a3 sp|Q9CQ91|NDUA3_MOUSE NADH dehy drogenase [ubiquinone] 1 alpha subcomplex subunit 3 OS=Mus musculus OX=10090 GN=Nduf a3 PE=1 SV=1;tr|D3Z670|D3Z670_MOUSE Complex I-B9 OS=Mus musculus OX=10090 GN=Nduf a3 PE=1 SV=1

29.73982 29.5674 29.79348 29.89729 30.00075 29.95768 29.91085 29.86377 anatomica cell part;cy toplasm;endoplasmi 12 12 12 60.2 60.2 60.2 21.867 0 113.43 33.10004 86 11 9 10 9 12 12 12 12 1.302796563 0.648841608 0.183763504 2.4499072 P12815 P12815 Programmed cell d Pdcd6 sp|P12815|PDCD6_MOUSE Programmed cell death protein 6 OS=Mus musculus OX=10090 GN=Pdcd6 PE=1 SV=2

28.0438 28.48999 28.37799 28.0451 28.02855 27.8695 27.39877 27.90524 metabolic cell part;e Drug meta REACT_2 4 4 4 40.5 40.5 40.5 16.958 0 53.131 31.38299 62 4 4 4 3 2 2 2 2 1.298420826 0.654679245 -0.438707829 -2.442489 Q9CPU4 Q9CPU4 Microsomal glutath Mgst3 sp|Q9CPU4|MGST3_MOUSE Microsomal glutathione S-transf erase 3 OS=Mus musculus OX=10090 GN=Mgst3 PE=1 SV=1

27.81507 27.19282 27.53126 27.09527 27.76591 27.69312 28.04817 28.45105 alcohol m cell part;e PPAR sig REACT_2 9 9 9 24.6 24.6 24.6 71.275 0 53.109 31.10306 63 6 5 5 4 7 8 7 8 1.298000481 0.65376 0.580960274 2.4417766 Q60714;A0A1D5 Q60714 Long-chain f atty a Slc27a1 sp|Q60714|S27A1_MOUSE Long-chain f atty acid transport protein 1 OS=Mus musculus OX=10090 GN=Slc27a1 PE=1 SV=1

28.19103 27.91016 28.42898 28.15427 29.48844 30.04179 28.54414 28.46428 anatomica cell body ; Saliv ary secretion 6 6 6 46.4 46.4 46.4 15.531 0 47.599 32.3639 69 4 3 3 2 4 6 3 6 1.29307783 0.660460094 0.963551521 2.4334363 P21460;A2APX3 P21460;A Cy statin-C Cst3 sp|P21460|CY TC_MOUSE Cy statin-C OS=Mus musculus OX=10090 GN=Cst3 PE=1 SV=2;tr|A2APX3|A2APX3_MOUSE Cy statin-C (Fragment) OS=Mus musculus OX=10090 GN=Cst3 PE=1 SV=1

29.46892 29.69362 29.52858 29.38028 29.49526 29.98929 29.91855 30.06407 biological cell part;c Ribosome REACT_2 21 21 21 25.8 25.8 25.8 123.09 0 104.61 32.97961 100 13 14 16 11 9 16 17 17 1.292428255 0.659550351 0.348940849 2.4323361 Q6P5F9;A2AKT6 Q6P5F9 Exportin-1 Xpo1 sp|Q6P5F9|XPO1_MOUSE Exportin-1 OS=Mus musculus OX=10090 GN=Xpo1 PE=1 SV=1

26.8236 26.82639 26.85389 26.72338 26.73931 27.29272 27.33841 27.23004 autophagy cell part;cell projection;endoplas 8 8 8 23.5 23.5 23.5 46.551 0 25.603 30.31969 36 7 7 7 7 5 7 7 7 1.287275276 0.667925234 0.343309402 2.4236115 Q60766 Q60766 Immunity -related Irgm1 sp|Q60766|IRGM1_MOUSE Immunity -related GTPase f amily M protein 1 OS=Mus musculus OX=10090 GN=Irgm1 PE=1 SV=1

25.9385 26.25451 26.32184 26.84098 27.1206 27.06271 27.24018 26.4396 biological cell part;cy toplasm;intracellular 14 1 1 69.3 8.2 8.2 28.728 0 12.804 29.93874 18 1 1 1 1 1 1 1 1 1.284890856 0.670284382 0.626816273 2.4195761 A0A0R4J1L2;F6 A0A0R4J1L2;F6ZFU0;D3Y Y Eef 1d tr|A0A0R4J1L2|A0A0R4J1L2_MOUSE Elongation f actor 1-delta OS=Mus musculus OX=10090 GN=Eef 1d PE=1 SV=1;tr|F6ZFU0|F6ZFU0_MOUSE Elongation f actor 1-delta (Fragment) OS=Mus musculus OX=10090 GN=Eef 1d PE=1 SV=1;tr|D3YY 68|D3Y Y 68_MOUSE Elongation f actor 1-delta OS

23.30341 23.05451 23.317 23.46638 23.79422 23.5064 23.67001 23.37782 biological cell part;c Apoptosis REACT_2 3 3 3 10.2 10.2 10.2 50.157 0 5.5899 26.806 16 2 2 2 2 3 3 3 3 1.281906925 0.67375814 0.301790714 2.4145277 Q3USX5;Q8R4K Q3USX5;QInterleukin-1 recep Irak4 tr|Q3USX5|Q3USX5_MOUSE Interleukin-1 receptor-associated kinase 4 OS=Mus musculus OX=10090 GN=Irak4 PE=1 SV=1;sp|Q8R4K2|IRAK4_MOUSE Interleukin-1 receptor-associated kinase 4 OS=Mus musculus OX=10090 GN=Irak4 PE=1 SV=1

24.61156 24.68089 24.47988 24.70171 25.04498 25.19985 24.6804 24.7891 alcohol m cell part;c Fructose REACT_3 5 5 5 32.3 32.3 32.3 29.19 0 14.487 27.9345 11 3 2 2 2 2 3 3 3 1.280612604 0.674227378 0.31007576 2.4123384 Q8BZA9 Q8BZA9 Fructose-2,6-bisph Tigar sp|Q8BZA9|TIGAR_MOUSE Fructose-2,6-bisphosphatase TIGAR OS=Mus musculus OX=10090 GN=Tigar PE=1 SV=1

25.54112 25.60981 25.19156 25.66498 24.38555 25.24402 24.9072 25.25339 10 10 2 6.9 6.9 1.5 284.12 0 11.999 28.63166 26 5 5 4 6 3 7 5 6 1.279081894 0.675361111 -0.554327488 -2.40975 A0A2I3BRX9;A0 A0A2I3BRX9;A0A1W2P712 **Ralgapa1** tr|A0A2I3BRX9|A0A2I3BRX9_MOUSE Ral GTPase-activ ating protein subunit alpha-1 OS=Mus musculus OX=10090 GN=Ralgapa1 PE=1 SV=1;tr|A0A1W2P712|A0A1W2P712_MOUSE Ral GTPase-activ ating protein subunit alpha-1 OS=Mus musculus OX=10090 GN=Ralgapa1 PE=1 SV=1

27.86856 27.98354 27.67229 27.77348 27.87168 28.04427 28.2544 28.13389 biological AP-ty pe mLy sosome REACT_3 22 22 19 24.3 24.3 21.4 122.74 0 45.352 31.38618 89 12 13 14 10 14 19 16 18 1.278370501 0.674983834 0.251590252 2.4085467 Q9Z1T1;A0A338 Q9Z1T1 AP-3 complex sub Ap3b1 sp|Q9Z1T1|AP3B1_MOUSE AP-3 complex subunit beta-1 OS=Mus musculus OX=10090 GN=Ap3b1 PE=1 SV=2

22.05381 22.34981 22.71687 21.86861 22.28418 20.41295 21.21247 20.18087 aging;anat cell part;Golgi appara REACT_2 3 2 2 1.7 1.3 1.3 230.56 0.002613 2.0801 24.36098 2 1 1 2 1 0 0 0 0 1.274426211 0.679981567 -1.224657059 -2.401879 A0A5F8MPX1;G3 A0A5F8M Histone acety ltran Kat6a tr|A0A5F8MPX1|A0A5F8MPX1_MOUSE Histone acety ltransf erase OS=Mus musculus OX=10090 GN=Kat6a PE=1 SV=1;tr|G3X940|G3X940_MOUSE Histone acety ltransf erase OS=Mus musculus OX=10090 GN=Kat6a PE=1 SV=1;sp|Q8BZ21|KAT6A_MOUSE Histone acety ltransf erase KAT6A OS=Mus m

23.89984 24.19873 24.04101 24.65905 24.79818 24.31797 25.20672 24.83215 anatomica cell body ; Axon guid REACT_3 4 4 4 5.5 5.5 5.5 103 0 7.4121 27.99297 8 1 1 1 1 2 2 4 3 1.27191071 0.682832184 0.589094162 2.3976278 P97333 P97333 Neuropilin-1 Nrp1 sp|P97333|NRP1_MOUSE Neuropilin-1 OS=Mus musculus OX=10090 GN=Nrp1 PE=1 SV=2

30.0444 29.55225 29.57225 29.72548 30.06664 30.0879 29.88336 30.02907 anatomica cell part;e One carbo REACT_3 32 32 32 71.6 71.6 71.6 64.217 0 218.99 33.12639 206 26 28 29 25 26 27 26 27 1.26683579 0.690889908 0.293151379 2.3890552 Q9CWJ9 Q9CWJ9 Bif unctional purine Atic sp|Q9CWJ9|PUR9_MOUSE Bif unctional purine biosy nthesis protein ATIC OS=Mus musculus OX=10090 GN=Atic PE=1 SV=2

28.64495 28.59142 28.14005 28.57474 27.97213 28.06204 28.39018 28.11828 biological cell part;c Mineral ab REACT_3 14 14 14 37 37 37 54.716 0 91.78 31.53487 83 12 12 12 9 7 8 8 8 1.265568331 0.691102975 -0.352134228 -2.386915 Q60738;F6TRQ3 Q60738 Zinc transporter 1 Slc30a1 sp|Q60738|ZNT1_MOUSE Zinc transporter 1 OS=Mus musculus OX=10090 GN=Slc30a1 PE=1 SV=1

24.68309 24.85828 24.38456 23.50821 24.84828 24.87319 26.10466 25.80247 biological regulation;catabolic process;cellu 17 17 17 12.8 12.8 12.8 294.13 0 27.503 28.5177 25 3 3 2 1 4 8 11 10 1.26464417 0.690757991 1.048617363 2.3853546 E9PV45;B1AY 13 E9PV45;B Ubiquitin carboxy l- Usp24 tr|E9PV45|E9PV45_MOUSE Ubiquitiny l hy drolase 1 OS=Mus musculus OX=10090 GN=Usp24 PE=1 SV=1;sp|B1AY 13|UBP24_MOUSE Ubiquitin carboxy l-terminal hy drolase 24 OS=Mus musculus OX=10090 GN=Usp24 PE=1 SV=1

20.35332 20.66398 22.58578 19.11885 22.62509 22.74948 22.34589 22.01219 autophagy cell part;endosome;extracellular 3 3 3 11.1 11.1 11.1 36.313 0 5.7288 25.38998 5 1 0 1 0 2 3 1 2 1.262547783 0.692583144 1.752681732 2.3818156 Q9QY 73;D3Y VMQ9QY 73; Transmembrane pr Tmem59 sp|Q9QY 73|TMM59_MOUSE Transmembrane protein 59 OS=Mus musculus OX=10090 GN=Tmem59 PE=1 SV=2;tr|D3YVM2|D3YVM2_MOUSE Transmembrane protein 59 OS=Mus musculus OX=10090 GN=Tmem59 PE=1 SV=1

26.9969 26.80255 26.72624 26.74021 26.54669 26.33828 26.23032 26.8055 cell part;intracellular REACT_2 7 7 7 32.5 32.5 32.5 47.778 0 22.897 29.8464 39 6 5 7 5 5 6 4 6 1.2615764 0.692236364 -0.336280346 -2.380176 Q8BK72;A0A338 Q8BK72 28S ribosomal prot Mrps27 sp|Q8BK72|RT27_MOUSE 28S ribosomal protein S27, mitochondrial OS=Mus musculus OX=10090 GN=Mrps27 PE=1 SV=2

24.80858 23.78656 23.37544 24.159 25.52022 24.67718 24.86231 24.58984 alcohol m cell part;c Fructose REACT_2 8 4 4 34.5 19.3 19.3 36.12 0 19.598 27.77102 17 2 1 1 2 4 3 2 2 1.260910128 0.692054422 0.879992485 2.3790516 P45377 P45377 Aldose reductase-r Akr1b8 sp|P45377|ALD2_MOUSE Aldose reductase-related protein 2 OS=Mus musculus OX=10090 GN=Akr1b8 PE=1 SV=2

20.19199 19.29725 22.64361 20.6382 22.48005 22.28402 22.93497 22.03688 3 3 3 22.4 22.4 22.4 26.445 0 24.837 26.04933 6 1 0 1 1 1 2 2 1 1.259567486 0.692895928 1.741219044 2.3767859 Q8BH50;Q0VAW Q8BH50;QUncharacterized pr 8030462N sp|Q8BH50|CR025_MOUSE Uncharacterized protein C18orf 25 homolog OS=Mus musculus OX=10090 PE=1 SV=1;tr|Q0VAW6|Q0VAW6_MOUSE RIKEN cDNA 8030462N17 gene OS=Mus musculus OX=10090 GN=8030462N17Rik PE=1 SV=1

30.08601 30.28405 30.12583 30.23112 30.31851 30.55215 30.25676 30.34615 cellular m cell part;cy toplasmic membrane 24 24 10 79.3 79.3 35.5 46.284 0 165.22 33.54731 195 20 20 19 22 22 23 20 20 1.255643429 0.696848758 0.186639786 2.3701658 O88544;F6QTS1 O88544;F COP9 signalosom Cops4 sp|O88544|CSN4_MOUSE COP9 signalosome complex subunit 4 OS=Mus musculus OX=10090 GN=Cops4 PE=1 SV=1;tr|F6QTS1|F6QTS1_MOUSE COP9 signalosome complex subunit 4 (Fragment) OS=Mus musculus OX=10090 GN=Cops4 PE=1 SV=1

24.659 24.35716 24.63583 24.38291 23.99516 24.34503 23.66904 24.32814 2 2 2 15.2 15.2 15.2 26.184 0 4.7681 27.60286 16 1 1 1 1 1 2 1 1 1.254470911 0.69736036 -0.424385548 -2.368188 Q8BGZ2;A0A140 Q8BGZ2; Protein FAM168A Fam168a sp|Q8BGZ2|F168A_MOUSE Protein FAM168A OS=Mus musculus OX=10090 GN=Fam168a PE=1 SV=1;tr|A0A140LIJ1|A0A140LIJ1_MOUSE Protein FAM168A OS=Mus musculus OX=10090 GN=Fam168a PE=1 SV=1;tr|A0A140LJ81|A0A140LJ81_MOUSE Protein FAM168A OS=Mus musculus OX=10090 GN=Fam1

28.62794 28.56874 28.27511 28.39317 28.17593 28.19404 28.29658 28.34541 cell part;cy toskeleton;intracellul 12 12 12 52.1 52.1 52.1 51.827 0 86.123 31.61732 63 10 9 8 9 7 10 9 10 1.24678602 0.70807191 -0.213251591 -2.355233 Q3UFK8;D3Z5B Q3UFK8; FERM domain-con Frmd8 sp|Q3UFK8|FRMD8_MOUSE FERM domain-containing protein 8 OS=Mus musculus OX=10090 GN=Frmd8 PE=1 SV=2;tr|D3Z5B2|D3Z5B2_MOUSE FERM domain-containing protein 8 (Fragment) OS=Mus musculus OX=10090 GN=Frmd8 PE=1 SV=8

24.52073 25.24325 25.32306 25.04568 25.44354 25.76524 25.6443 25.26832 cellular m cell part;intracellular membrane- 3 3 3 25.5 25.5 25.5 21.022 0 11.625 28.79337 15 2 2 3 1 2 3 3 3 1.246047226 0.708053812 0.497168064 2.3539881 Z4Y L87;H7BWY Z4Y L87;H Pre-rRNA-processi Tsr2 tr|Z4YL87|Z4Y L87_MOUSE Pre-rRNA-processing protein TSR2 homolog OS=Mus musculus OX=10090 GN=Tsr2 PE=1 SV=1;tr|H7BWY 8|H7BWY 8_MOUSE Pre-rRNA-processing protein TSR2 homolog OS=Mus musculus OX=10090 GN=Tsr2 PE=1 SV=1;sp|Q8C8T8|TSR2_MOUSE Pre-rRNA-processing p

27.47042 27.58305 27.25043 27.37948 27.58663 27.80021 27.60052 27.54679 biological cell part;intracellular membrane- 8 8 8 35.9 35.9 35.9 33.31 0 56.132 30.88954 54 5 4 5 4 7 8 7 7 1.245013742 0.708232662 0.212695599 2.3522468 Q9D0L7;D3Z5T2 Q9D0L7;D Armadillo repeat-c Armc10 sp|Q9D0L7|ARM10_MOUSE Armadillo repeat-containing protein 10 OS=Mus musculus OX=10090 GN=Armc10 PE=1 SV=1;tr|D3Z5T2|D3Z5T2_MOUSE Armadillo repeat-containing protein 10 OS=Mus musculus OX=10090 GN=Armc10 PE=1 SV=1

26.72624 26.5842 26.28336 26.84362 26.80452 26.90575 26.91171 27.18215 amine me cell part;m Py rimidine REACT_2 8 8 7 21.2 21.2 18.8 66.682 0 33.413 30.11706 20 3 3 3 4 6 8 7 8 1.242986129 0.709705357 0.341679096 2.348831 P70698 P70698 CTP sy nthase 1 Ctps1 sp|P70698|PY RG1_MOUSE CTP sy nthase 1 OS=Mus musculus OX=10090 GN=Ctps1 PE=1 SV=2

23.09304 23.03433 23.14799 23.0912 23.31714 23.18648 23.1249 23.17872 biological cell part;c Circadian REACT_1 3 3 3 4.9 4.9 4.9 96.264 0 4.326 26.42683 5 1 1 1 1 3 3 3 3 1.241768335 0.710832962 0.110170364 2.3467798 A0A0J9Y U61;O0 A0A0J9Y Circadian locomot Clock tr|A0A0J9Y U61|A0A0J9Y U61_MOUSE Circadian locomoter output cy cles protein kaput OS=Mus musculus OX=10090 GN=Clock PE=1 SV=1;sp|O08785|CLOCK_MOUSE Circadian locomoter output cy cles protein kaput OS=Mus musculus OX=10090 GN=Clock PE=1 SV=1

25.53579 25.67357 25.30671 25.54819 25.20056 24.83244 25.49451 24.70182 cell part 7 7 7 61.8 61.8 61.8 19.457 0 19.729 28.5079 41 6 5 5 4 3 4 4 3 1.241541952 0.709635556 -0.458734512 -2.346399 Q9CZH7;Q9D1T Q9CZH7 Matrix-remodeling- Mxra7 sp|Q9CZH7|MXRA7_MOUSE Matrix-remodeling-associated protein 7 OS=Mus musculus OX=10090 GN=Mxra7 PE=1 SV=2

31.04668 31.27159 31.26007 31.05232 31.00611 30.82388 30.81391 31.10225 response cell part;in Alzheimer' REACT_2 12 12 12 54.4 54.4 54.4 29.367 0 88.015 34.2758 143 11 11 11 10 12 11 11 10 1.239379999 0.711840355 -0.221128941 -2.342758 Q9CR68 Q9CR68 Cy tochrome b-c1 Uqcrf s1 sp|Q9CR68|UCRI_MOUSE Cy tochrome b-c1 complex subunit Rieske, mitochondrial OS=Mus musculus OX=10090 GN=Uqcrf s1 PE=1 SV=1

23.96877 24.67616 24.21527 24.13061 24.56098 24.97246 24.45362 24.7967 3 3 3 23.5 23.5 23.5 22.59 0 8.6152 28.03128 13 2 2 1 2 3 3 3 3 1.238739857 0.71100885 0.448236942 2.3416798 E9Q2W8;Q9D92 E9Q2W8; Loss of heterozy g Loh12cr1 tr|E9Q2W8|E9Q2W8_MOUSE BLOC-1-related complex subunit 5 OS=Mus musculus OX=10090 GN=Borcs5 PE=1 SV=1;sp|Q9D920|BORC5_MOUSE BLOC-1-related complex subunit 5 OS=Mus musculus OX=10090 GN=Borcs5 PE=1 SV=1

29.23409 29.14326 29.44679 29.056 29.22228 29.64835 29.59909 29.7867 cellular ke cell part;e Fatty acid REACT_3 17 17 17 52.3 52.3 52.3 47.874 0 114.81 32.67915 117 15 14 15 8 13 15 17 17 1.238561723 0.709933775 0.34406805 2.3413799 Q9DBL1;E9Q5L3 Q9DBL1;E Short/branched ch Acadsb sp|Q9DBL1|ACDSB_MOUSE Short/branched chain specif ic acy l-CoA dehy drogenase, mitochondrial OS=Mus musculus OX=10090 GN=Acadsb PE=1 SV=1;tr|E9Q5L3|E9Q5L3_MOUSE Short/branched chain-specif ic acy l-CoA dehy drogenase, mitochondrial OS=Mus musculus OX=10090 GN=Ac

24.98807 24.75113 25.34969 25.33552 25.01372 24.47277 24.56145 24.61813 biological cell part;intracellular membrane- 3 3 3 17.1 17.1 17.1 19.431 0 4.1976 28.0876 19 2 2 2 3 2 2 2 2 1.237933415 0.709162996 -0.439585686 -2.340322 Q8C6I2;A0A494 Q8C6I2;A Succinate dehy dro Sdhaf 2 sp|Q8C6I2|SDHF2_MOUSE Succinate dehy drogenase assembly f actor 2, mitochondrial OS=Mus musculus OX=10090 GN=Sdhaf 2 PE=1 SV=1;tr|A0A494B9B9|A0A494B9B9_MOUSE Succinate dehy drogenase assembly f actor 2, mitochondrial OS=Mus musculus OX=10090 GN=Sdhaf 2 PE=1 SV=1

20.54839 22.03493 21.33898 22.88317 22.993 22.85905 22.67973 22.97773 autophagy cell part;cy toplasm;intracellular 1 1 1 13.3 13.3 13.3 24.278 0.002608 2.0611 25.58403 3 0 1 0 1 1 1 1 1 1.237610608 0.708140659 1.176007271 2.3397786 A0A0R4J029;Q8 A0A0R4J0 Ubiquitin-like-conju Atg10 tr|A0A0R4J029|A0A0R4J029_MOUSE Ubiquitin-like-conjugating enzy me ATG10 OS=Mus musculus OX=10090 GN=Atg10 PE=1 SV=1;sp|Q8R1P4|ATG10_MOUSE Ubiquitin-like-conjugating enzy me ATG10 OS=Mus musculus OX=10090 GN=Atg10 PE=1 SV=1

25.03023 22.72812 22.84125 23.40002 24.70604 26.17162 24.4924 24.76025 cell part;nucleoplasm 7 6 6 20.5 19 19 65.508 0 20.033 28.20149 12 2 1 1 2 2 5 3 3 1.237306575 0.706815789 1.532671452 2.3392667 A0A1L1STF0;Q6 A0A1L1ST Protein FAM63B Fam63b tr|A0A1L1STF0|A0A1L1STF0_MOUSE Ubiquitiny l hy drolase 1 OS=Mus musculus OX=10090 GN=Mindy 2 PE=1 SV=1;sp|Q6PDI6|MINY 2_MOUSE Ubiquitin carboxy l-terminal hy drolase MINDY-2 OS=Mus musculus OX=10090 GN=Mindy 2 PE=1 SV=1

25.54032 25.63633 25.86514 25.45212 26.09945 25.78506 25.78649 25.90415 biological cell part;cy toplasm;Golgi appar 8 8 8 23.5 23.5 23.5 64.802 0 43.645 29.1346 36 5 6 6 3 4 7 8 6 1.235778052 0.707597374 0.270310402 2.3366936 Q05CL8;A2AMD Q05CL8 La-related protein Larp7 sp|Q05CL8|LARP7_MOUSE La-related protein 7 OS=Mus musculus OX=10090 GN=Larp7 PE=1 SV=2

24.87333 22.71869 21.97949 23.04835 24.59298 24.77661 25.00714 24.20307 biological cell part;c Acute my REACT_2 6 6 6 15.9 15.9 15.9 84.387 0 27.773 27.44443 16 4 3 2 3 3 3 4 1 1.235266693 0.706681223 1.489984512 2.3358329 A0A0R4J0T4;Q5 A0A0R4J0 Inhibitor of nuclear Ikbkb tr|A0A0R4J0T4|A0A0R4J0T4_MOUSE I-kappa-B kinase OS=Mus musculus OX=10090 GN=Ikbkb PE=1 SV=1;tr|Q5D0E0|Q5D0E0_MOUSE I-kappa-B kinase OS=Mus musculus OX=10090 GN=Ikbkb PE=1 SV=1;sp|O88351|IKKB_MOUSE Inhibitor of nuclear f actor kappa-B kinase subunit beta OS=

25.56741 23.36038 22.57265 26.09875 24.31271 20.37073 20.72869 19.34601 cell part;cy toplasm;intracellular 3 3 3 15.9 15.9 15.9 26.184 0 13.194 26.76775 11 2 1 1 3 1 0 0 1 1.234102817 0.706623094 -3.210258961 -2.333874 A0A087WRY 3;Q A0A087W Nuclear ubiquitous Nucks1 tr|A0A087WRY 3|A0A087WRY 3_MOUSE Nuclear ubiquitous casein and cy clin-dependent kinase substrate 1 OS=Mus musculus OX=10090 GN=Nucks1 PE=1 SV=1;sp|Q80XU3|NUCKS_MOUSE Nuclear ubiquitous casein and cy clin-dependent kinase substrate 1 OS=Mus musculus OX=10090 G

27.95643 28.20163 28.29022 27.79266 27.3601 27.80833 27.66438 27.8903 cell part;cy toplasm;intracellular 11 11 11 31.4 31.4 31.4 57.543 0 58.055 31.14388 58 10 10 9 5 6 9 9 9 1.231233354 0.71066087 -0.379458427 -2.329046 Q3UKJ7 Q3UKJ7 WD40 repeat-cont Smu1 sp|Q3UKJ7|SMU1_MOUSE WD40 repeat-containing protein SMU1 OS=Mus musculus OX=10090 GN=Smu1 PE=2 SV=2

25.48517 26.09241 26.68539 25.68656 26.39854 26.94648 26.89423 26.49337 biological cell part;e Aminobenzoate degra 5 5 5 23.2 23.2 23.2 39.351 0 27.951 29.81095 35 3 4 4 3 3 5 4 4 1.230627055 0.71010846 0.695772648 2.3280255 Q62087;H3BL07 Q62087 Serum paraoxonas Pon3 sp|Q62087|PON3_MOUSE Serum paraoxonase/lactonase 3 OS=Mus musculus OX=10090 GN=Pon3 PE=1 SV=2

20.25356 21.05515 22.04327 19.02127 22.68472 22.09429 23.10465 21.38237 biological cell part;macromolec REACT_2 3 3 3 1.5 1.5 1.5 301.83 0.001565 2.295 25.68955 5 1 0 0 1 1 1 1 2 1.229945896 0.709489177 1.723194599 2.3268795 D3Z3A8;Q8C170 D3Z3A8;Q Unconv entional m My o9a tr|D3Z3A8|D3Z3A8_MOUSE Unconv entional my osin-IXa OS=Mus musculus OX=10090 GN=My o9a PE=1 SV=1;sp|Q8C170|MY O9A_MOUSE Unconv entional my osin-IXa OS=Mus musculus OX=10090 GN=My o9a PE=1 SV=2

25.13352 25.55286 25.65954 25.25631 25.37786 26.0702 26.00828 26.24552 biological cell part;c Cell cy cle REACT_2 17 1 1 75.4 4.2 4.2 35.575 0 15.658 29.06566 8 1 1 1 1 1 1 1 1 1.227859459 0.711680346 0.524907112 2.3233699 P62715 P62715 Serine/threonine-pr Ppp2cb sp|P62715|PP2AB_MOUSE Serine/threonine-protein phosphatase 2A cataly tic subunit beta isof orm OS=Mus musculus OX=10090 GN=Ppp2cb PE=1 SV=1

30.4513 30.17783 30.04101 30.54793 30.51277 30.6785 30.66076 30.53018 biological cell part;c Endocy tosis 44 44 44 61.7 61.7 61.7 96.023 0 264.77 33.73237 260 34 32 31 33 34 34 34 33 1.226941004 0.711577586 0.291034698 2.3218252 Q9WU78 Q9WU78 Programmed cell d Pdcd6ip sp|Q9WU78|PDC6I_MOUSE Programmed cell death 6-interacting protein OS=Mus musculus OX=10090 GN=Pdcd6ip PE=1 SV=3

26.96697 27.50408 28.04614 27.64045 28.48635 28.23872 28.1052 27.7929 autophagy cell part;cy toplasm;cy toskeleto 4 2 2 28.1 22.3 22.3 14.272 0.000164 3.0255 31.16932 18 1 1 1 1 2 2 2 2 1.226450143 0.711449462 0.616382599 2.3209997 Q91VR7 Q91VR7 Microtubule-associ Map1lc3a sp|Q91VR7|MLP3A_MOUSE Microtubule-associated proteins 1A/1B light chain 3A OS=Mus musculus OX=10090 GN=Map1lc3a PE=1 SV=1

23.86164 23.68977 23.55742 23.73521 24.51063 23.95766 24.69562 23.78237 cell part;centrosome;intracellula 7 7 7 9.9 9.9 9.9 89.559 0 18.955 27.29911 21 4 4 3 3 5 7 5 5 1.22425882 0.714248927 0.525558472 2.317315 E9Q6K3 E9Q6K3 Pibf 1 tr|E9Q6K3|E9Q6K3_MOUSE Progesterone immunomodulatory -binding f actor 1 OS=Mus musculus OX=10090 GN=Pibf 1 PE=1 SV=1

27.26946 27.24318 26.82274 26.81812 27.59385 27.45089 27.183 27.34299 anatomica cell part;cy toplasm;mREACT_3 11 11 11 66.8 66.8 66.8 27.385 0 36.374 30.53584 41 5 8 6 5 8 8 8 8 1.223970206 0.71351606 0.354307652 2.3168297 Q9D1E6 Q9D1E6 Tubulin-f olding cof Tbcb sp|Q9D1E6|TBCB_MOUSE Tubulin-f olding cof actor B OS=Mus musculus OX=10090 GN=Tbcb PE=1 SV=2

23.7383 24.58176 24.31534 23.94253 25.61203 24.35602 24.5686 25.26564 biological extracellular organelle;extracellu 4 4 4 26.7 26.7 26.7 37.325 0 11.877 27.9447 8 4 1 1 4 2 4 4 2 1.219383185 0.718564103 0.806089401 2.3091193 P29699;A0A338 P29699;A Alpha-2-HS-gly cop Ahsg sp|P29699|FETUA_MOUSE Alpha-2-HS-gly coprotein OS=Mus musculus OX=10090 GN=Ahsg PE=1 SV=1;tr|A0A338P703|A0A338P703_MOUSE Alpha-2-HS-gly coprotein OS=Mus musculus OX=10090 GN=Ahsg PE=1 SV=1;tr|A0A338P7G1|A0A338P7G1_MOUSE Alpha-2-HS-gly coprotein OS=Mus musculu

27.69975 27.61526 27.561 27.71339 27.90064 27.89076 27.79191 27.64554 metabolic cell part;cy toplasm REACT_3 8 8 5 57.5 57.5 40.2 20.156 0 34.817 30.98114 56 8 7 7 7 8 8 8 7 1.21747351 0.719855011 0.159860611 2.3059102 Q8R2U6 Q8R2U6 Diphosphoinositol Nudt4 sp|Q8R2U6|NUDT4_MOUSE Diphosphoinositol poly phosphate phosphohy drolase 2 OS=Mus musculus OX=10090 GN=Nudt4 PE=1 SV=1

25.40305 25.39983 24.71811 25.562 26.08527 25.46896 25.71057 25.96475 anatomica cell part;centrosome; REACT_2 7 7 7 8.8 8.8 8.8 133.99 0 18.186 28.88853 34 5 6 4 3 2 5 5 7 1.217079765 0.718782979 0.536637783 2.3052486 Q8BND3;E0CYDQ8BND3 WD repeat-contain Wdr35 sp|Q8BND3|WDR35_MOUSE WD repeat-containing protein 35 OS=Mus musculus OX=10090 GN=Wdr35 PE=1 SV=3

27.18395 27.31501 26.8776 27.48861 27.5139 27.44387 27.63291 27.51721 cell part;cy toplasm;extracellular 11 9 9 23.4 20.2 20.2 57.427 0 18.553 30.66814 42 6 6 5 6 9 9 8 9 1.216762519 0.717690021 0.310679436 2.3047156 B7ZNU9;Q61235 B7ZNU9; Beta-2-sy ntrophin Sntb2 tr|B7ZNU9|B7ZNU9_MOUSE Beta-2-sy ntrophin OS=Mus musculus OX=10090 GN=Sntb2 PE=1 SV=1;sp|Q61235|SNTB2_MOUSE Beta-2-sy ntrophin OS=Mus musculus OX=10090 GN=Sntb2 PE=1 SV=2

23.22738 19.04417 25.44446 21.81837 32.87526 34.43428 26.8023 22.95342 biological cell part;c Adipocy to REACT_2 13 13 13 58.3 58.3 58.3 26.707 0 129.15 35.70381 88 3 0 3 0 10 12 7 2 1.215559369 0.717991525 6.88271904 2.3026943 P01193;A0A1W2 P01193 Pro-opiomelanocor Pomc sp|P01193|COLI_MOUSE Pro-opiomelanocortin OS=Mus musculus OX=10090 GN=Pomc PE=1 SV=1

21.88911 22.29098 22.39312 22.76921 22.96019 22.68127 23.03528 22.5961 anatomica cell part;cell projectio REACT_2 6 6 6 5.7 5.7 5.7 131.29 0 8.0487 26.01831 15 3 3 3 3 4 3 4 4 1.214347613 0.717742072 0.482605457 2.3006587 A0A1L1SUF9;D3 A0A1L1SUCentrosomal prote Cep164 tr|A0A1L1SUF9|A0A1L1SUF9_MOUSE Centrosomal protein of 164 kDa (Fragment) OS=Mus musculus OX=10090 GN=Cep164 PE=1 SV=1;tr|D3YVU3|D3YVU3_MOUSE Centrosomal protein of 164 kDa OS=Mus musculus OX=10090 GN=Cep164 PE=1 SV=1;tr|A0A1L1SSA4|A0A1L1SSA4_MOUSE Centroso

25.51355 25.67535 26.0003 25.97384 25.56203 25.21115 25.38265 25.62313 biological cell part;intracellular membrane- 7 7 7 11.2 11.2 11.2 126.18 0 13.87 28.81115 27 4 6 4 5 4 4 5 5 1.213981707 0.716936709 -0.346021175 -2.300044 Q91Z96 Q91Z96 BMP-2-inducible pr Bmp2k sp|Q91Z96|BMP2K_MOUSE BMP-2-inducible protein kinase OS=Mus musculus OX=10090 GN=Bmp2k PE=1 SV=1

24.65992 24.60428 24.44979 24.50367 25.46289 24.5041 25.06266 25.04798 anatomica cell part;cell projectio REACT_3 10 10 10 15.2 15.2 15.2 79.284 0 15.956 28.05092 26 5 5 2 3 6 6 5 8 1.211760261 0.720050526 0.464990616 2.296313 O35594;D3Z5A8 O35594 Intraf lagellar trans If t81 sp|O35594|IFT81_MOUSE Intraf lagellar transport protein 81 homolog OS=Mus musculus OX=10090 GN=If t81 PE=1 SV=4

26.45497 26.234 26.47537 25.37749 24.84675 25.56602 25.68896 25.50443 anatomica cell part;cy toskeleton;intracellul 13 13 13 35.8 35.8 35.8 71.051 0 36.654 29.15726 33 9 9 7 3 4 6 6 5 1.209476879 0.723268908 -0.733918667 -2.292479 Q6ZPU9;H3BIY Q6ZPU9; KIF1-binding prote Kbp;25100 sp|Q6ZPU9|KBP_MOUSE KIF-binding protein OS=Mus musculus OX=10090 GN=Kif bp PE=1 SV=2;tr|H3BIY 2|H3BIY 2_MOUSE KIF-binding protein OS=Mus musculus OX=10090 GN=Kif bp PE=1 SV=1

25.27519 25.44057 25.89152 25.38476 25.67944 25.79571 25.9623 25.93616 cellular m cell part;in Butanoate REACT_3 8 8 8 22.8 22.8 22.8 50.898 0 31.098 28.90607 38 4 4 6 5 6 6 6 7 1.209140696 0.722658281 0.345392227 2.2919144 Q91Y P0 Q91Y P0 L-2-hy droxy glutara L2hgdh sp|Q91Y P0|L2HDH_MOUSE L-2-hy droxy glutarate dehy drogenase, mitochondrial OS=Mus musculus OX=10090 GN=L2hgdh PE=1 SV=1

25.44044 25.33323 26.16446 25.70213 25.88103 26.12183 26.25102 26.2703 amine me cell part;e Sphingolip REACT_3 1 1 1 4.8 4.8 4.8 35.955 0 13.936 29.20783 15 1 1 1 1 1 1 1 1 1.207967896 0.72232636 0.470978737 2.2899454 Q6GV12 Q6GV12 3-ketodihy drosphin Kdsr sp|Q6GV12|KDSR_MOUSE 3-ketodihy drosphingosine reductase OS=Mus musculus OX=10090 GN=Kdsr PE=1 SV=1

27.138 26.96334 27.06404 27.58764 27.509 27.62261 27.79947 27.3517 anatomica cell part;c RNA trans REACT_2 4 4 4 43.6 43.6 43.6 11.557 0 18.346 30.57777 41 4 4 4 4 4 4 4 4 1.207678008 0.721453027 0.38244009 2.2894587 P63166;A0A087 P63166;A Small ubiquitin-rela Sumo1 sp|P63166|SUMO1_MOUSE Small ubiquitin-related modif ier 1 OS=Mus musculus OX=10090 GN=Sumo1 PE=1 SV=1;tr|A0A087WRQ4|A0A087WRQ4_MOUSE Small ubiquitin-related modif ier 1 OS=Mus musculus OX=10090 GN=Sumo1 PE=1 SV=1

26.28113 25.82355 25.6852 26.00045 26.93101 26.66384 26.32682 26.02441 cellular m cell part;membrane 7 7 7 14.1 14.1 14.1 64.697 0 9.5452 29.94977 14 3 3 3 1 3 4 3 4 1.206261179 0.72185 0.538936138 2.2870804 Q61576;A2A4H9 Q61576;A Peptidy l-proly l cis- Fkbp10 sp|Q61576|FKB10_MOUSE Peptidy l-proly l cis-trans isomerase FKBP10 OS=Mus musculus OX=10090 GN=Fkbp10 PE=1 SV=2;tr|A2A4H9|A2A4H9_MOUSE Peptidy lproly l isomerase OS=Mus musculus OX=10090 GN=Fkbp10 PE=1 SV=1

30.51738 30.52384 30.26941 30.8359 30.78503 30.81948 30.99766 30.72899 metabolic cell part;c Neurotrop REACT_2 12 12 12 73.5 73.5 73.5 22.851 0 174.71 33.91403 178 11 12 11 10 10 10 10 10 1.203890856 0.72429106 0.296156406 2.2831021 Q61599;D3Y WL Q61599 Rho GDP-dissocia Arhgdib sp|Q61599|GDIR2_MOUSE Rho GDP-dissociation inhibitor 2 OS=Mus musculus OX=10090 GN=Arhgdib PE=1 SV=3

28.38648 28.325 28.3871 28.18929 28.49499 28.51047 28.41844 28.37737 cellular co cell body ;cell part;cell projection 10 10 10 63.5 63.5 63.5 30.972 0 76.692 31.68954 58 8 7 8 8 8 8 8 8 1.203080449 0.72473029 0.128349781 2.2817422 Q8K4Z3 Q8K4Z3 NAD(P)H-hy drate Apoa1bp sp|Q8K4Z3|NNRE_MOUSE NAD(P)H-hy drate epimerase OS=Mus musculus OX=10090 GN=Naxe PE=1 SV=1

22.79761 22.98355 23.24288 23.18628 23.39859 23.93157 24.04193 23.08706 biosy nthe cell part;intracellular membrane- 9 9 9 7.4 7.4 7.4 225.54 0 15.517 26.52859 19 5 3 4 2 3 4 3 5 1.199921878 0.727817805 0.562205791 2.2764427 B2RQG2 B2RQG2 Phf 3 tr|B2RQG2|B2RQG2_MOUSE PHD f inger protein 3 OS=Mus musculus OX=10090 GN=Phf 3 PE=1 SV=1

27.37393 27.51194 27.42575 27.75996 27.84356 27.85341 27.63644 27.66986 anatomica cell part;e Ly sine de REACT_2 15 15 15 32.8 32.8 32.8 84.921 0 67.524 30.85846 73 12 13 13 11 10 8 11 10 1.194668962 0.737 0.232923985 2.2676325 Q9R0E1;F6W3Q Q9R0E1 Procollagen-ly sine Plod3 sp|Q9R0E1|PLOD3_MOUSE Multif unctional procollagen ly sine hy droxy lase and gly cosy ltransf erase LH3 OS=Mus musculus OX=10090 GN=Plod3 PE=1 SV=1

28.50271 28.4149 29.00852 28.50896 28.22366 28.09431 28.50089 28.14253 5 5 5 77.2 77.2 77.2 6.954 0 16.421 31.61973 42 4 3 5 3 3 3 3 3 1.192771781 0.738820619 -0.368423462 -2.264452 P0DN34 P0DN34 **Ndufb1** sp|P0DN34|NDUB1_MOUSE NADH dehy drogenase [ubiquinone] 1 beta subcomplex subunit 1 OS=Mus musculus OX=10090 GN=Nduf b1 PE=1 SV=1

27.67432 27.32714 28.10415 27.80452 26.96235 27.55954 27.33543 26.49621 biological cell part;c mRNA su REACT_2 7 7 7 50.6 50.6 50.6 19.586 0 22.114 30.85646 46 5 5 6 5 5 6 6 6 1.189618367 0.742814815 -0.639151096 -2.259165 E9Q317;O55128 E9Q317;O Histone deacety la Gm10094; tr|E9Q317|E9Q317_MOUSE 18 kDa Sin3-associated poly peptide OS=Mus musculus OX=10090 GN=Sap18 PE=1 SV=1;sp|O55128|SAP18_MOUSE Histone deacety lase complex subunit SAP18 OS=Mus musculus OX=10090 GN=Sap18 PE=1 SV=1;tr|D3Z2N9|D3Z2N9_MOUSE Histone deacety lase com

25.24372 25.57398 24.89444 25.35951 26.06556 25.91075 25.81965 25.27828 anatomica cell part;cell projection;cy toplas 6 6 6 16.3 16.3 16.3 69.558 0 14.953 28.74663 25 3 3 3 4 3 4 4 3 1.184796552 0.75123614 0.500645638 2.2510853 Q9Z2C5;B1AW2 Q9Z2C5;B My otubularin Mtm1 sp|Q9Z2C5|MTM1_MOUSE My otubularin OS=Mus musculus OX=10090 GN=Mtm1 PE=1 SV=2;tr|B1AW21|B1AW21_MOUSE My otubularin OS=Mus musculus OX=10090 GN=Mtm1 PE=1 SV=1

27.95931 27.0592 27.71142 27.24862 28.57975 28.6973 27.82578 27.76269 anatomica cell part;cy toplasm;c REACT_3 14 14 14 35.9 35.9 35.9 51.655 0 49.482 31.28797 54 7 5 6 5 11 11 8 9 1.184138177 0.750336066 0.721741199 2.2499823 Q06890;E9PUU2 Q06890;E Clusterin;Clusterin Clu sp|Q06890|CLUS_MOUSE Clusterin OS=Mus musculus OX=10090 GN=Clu PE=1 SV=1;tr|E9PUU2|E9PUU2_MOUSE Clusterin (Fragment) OS=Mus musculus OX=10090 GN=Clu PE=1 SV=1;tr|E9PXG5|E9PXG5_MOUSE Clusterin (Fragment) OS=Mus musculus OX=10090 GN=Clu PE=1 SV=1;tr|E9Q8Y 5|E

25.0739 25.22391 25.20605 25.34411 25.65685 25.44761 25.27405 25.35984 cell part;cy toplasm;i REACT_2 8 8 8 10.9 10.9 10.9 99.229 0 12.377 28.72107 25 3 3 3 3 3 5 6 5 1.183114358 0.750118609 0.222595215 2.2482672 Q7TSQ8;D6RD0 Q7TSQ8;DPy ruv ate dehy dro Pdpr sp|Q7TSQ8|PDPR_MOUSE Py ruv ate dehy drogenase phosphatase regulatory subunit, mitochondrial OS=Mus musculus OX=10090 GN=Pdpr PE=1 SV=1;tr|D6RD07|D6RD07_MOUSE Py ruv ate dehy drogenase phosphatase regulatory subunit, mitochondrial OS=Mus musculus OX=10090 GN=Pdp

23.02066 23.01388 22.37406 22.91816 24.12904 23.06614 23.82658 23.07633 anatomica cell body ;cell part;cell projection 9 1 1 26.1 2.6 2.6 52.675 0.000642 2.6911 26.46165 6 1 1 1 1 1 1 1 1 1.174994565 0.763118367 0.692831516 2.2346695 Q3TLQ0;G3UW Q3TLQ0 Microtubule-associ Map2 tr|Q3TLQ0|Q3TLQ0_MOUSE Microtubule-associated protein OS=Mus musculus OX=10090 GN=Map2 PE=1 SV=1

29.04698 28.48385 29.10074 28.55322 28.03575 28.50423 28.40532 28.49743 biological cell body ;cell part;ce REACT_3 5 5 5 53.1 53.1 53.1 14.099 0 61.687 31.87847 56 5 4 5 4 4 5 5 5 1.170145292 0.771095723 -0.435515881 -2.226553 G3UZY 2;P97493 G3UZY 2; Thioredoxin, mitoc Txn2 tr|G3UZY 2|G3UZY 2_MOUSE Thioredoxin, mitochondrial (Fragment) OS=Mus musculus OX=10090 GN=Txn2 PE=1 SV=1;sp|P97493|THIOM_MOUSE Thioredoxin, mitochondrial OS=Mus musculus OX=10090 GN=Txn2 PE=1 SV=1

26.07778 26.7557 26.8125 26.21655 26.98663 26.95327 26.72507 26.94 establishmcell part;in Proximal t REACT_2 6 6 6 26.8 26.8 26.8 31.715 0 37.514 30.04492 30 5 5 5 3 3 5 5 5 1.169938023 0.769739837 0.435614109 2.2262062 Q9QZD8 Q9QZD8 Mitochondrial dicar Slc25a10 sp|Q9QZD8|DIC_MOUSE Mitochondrial dicarboxy late carrier OS=Mus musculus OX=10090 GN=Slc25a10 PE=1 SV=2

20.30392 22.44846 20.05066 21.36461 23.26413 22.36594 21.74763 22.40304 anatomica cell part;c Axon guid REACT_2 3 2 2 4.1 3 3 108.85 0.007196 1.5598 25.31983 3 0 1 0 0 1 1 1 1 1.168959502 0.769476673 1.40327549 2.2245687 Q03145 Q03145 Ephrin ty pe-A rece Epha2 sp|Q03145|EPHA2_MOUSE Ephrin ty pe-A receptor 2 OS=Mus musculus OX=10090 GN=Epha2 PE=1 SV=3

27.02136 27.42013 27.56493 27.58169 28.57521 28.02083 27.488 27.82826 cellular pr cell part;e Collecting REACT_2 5 5 5 72.3 72.3 72.3 13.37 0 90.869 31.00062 30 4 3 4 3 5 4 5 5 1.163886692 0.776518219 0.581048489 2.2160821 Q9D1K2;A0A0N Q9D1K2;A V-ty pe proton ATP Atp6v 1f sp|Q9D1K2|VATF_MOUSE V-ty pe proton ATPase subunit F OS=Mus musculus OX=10090 GN=Atp6v 1f PE=1 SV=2;tr|A0A0N4SVE1|A0A0N4SVE1_MOUSE V-ATPase 14 kDa subunit OS=Mus musculus OX=10090 GN=Atp6v 1f PE=1 SV=1;tr|F7B2B4|F7B2B4_MOUSE V-ATPase 14 kDa subunit (Fragment)

25.24989 25.00488 25.05292 25.83641 26.32716 25.9616 25.75537 25.44903 anatomica cell part;chromatin re REACT_3 9 5 4 26.2 15.6 13 51.96 0 18.609 28.86806 21 2 3 2 2 4 4 3 3 1.161847663 0.77849697 0.587267399 2.2126718 A0A0G2JG60;Q6 A0A0G2J SWI/SNF-related Smarcd3 tr|A0A0G2JG60|A0A0G2JG60_MOUSE SWI/SNF-related matrix-associated actin-dependent regulator of chromatin subf amily D member 3 OS=Mus musculus OX=10090 GN=Smarcd3 PE=1 SV=1;sp|Q6P9Z1|SMRD3_MOUSE SWI/SNF-related matrix-associated actin-dependent regulator of

23.15677 22.94705 23.0295 23.13268 23.30855 23.08541 23.35151 23.19943 cell part;membrane 2 2 2 5.2 5.2 5.2 41.739 0 4.9028 26.34084 9 2 2 2 2 2 2 1 2 1.160154139 0.779483871 0.169726849 2.2098398 Q3TBU7;Q3U2K Q3TBU7;QArf -GAP domain a Agf g2 tr|Q3TBU7|Q3TBU7_MOUSE Arf -GAP domain and FG repeat-containing protein 2 (Fragment) OS=Mus musculus OX=10090 GN=Agf g2 PE=1 SV=1;tr|Q3U2K8|Q3U2K8_MOUSE Arf -GAP domain and FG repeat-containing protein 2 OS=Mus musculus OX=10090 GN=Agf g2 PE=1 SV=1;sp|Q80WC7|A

24.71303 24.70213 25.31206 24.51503 24.47698 24.26234 23.83403 24.56744 cell part;intracellular REACT_2 4 4 4 11.3 11.3 11.3 45.029 0 3.6883 27.97837 14 2 2 2 1 2 2 3 2 1.157838877 0.782124748 -0.525365353 -2.205969 Q8K2M0 Q8K2M0 39S ribosomal prot Mrpl38 sp|Q8K2M0|RM38_MOUSE 39S ribosomal protein L38, mitochondrial OS=Mus musculus OX=10090 GN=Mrpl38 PE=1 SV=2

29.81224 29.55028 29.40937 30.02683 30.05556 29.81912 30.18139 30.15436 29 29 2 44.9 44.9 2 78.317 0 164.33 33.1566 242 25 24 24 23 26 25 28 28 1.156534874 0.783413655 0.352929592 2.2037885 A0A494B9K4;A0 A0A494B9K4 **Add3** tr|A0A494B9K4|A0A494B9K4_MOUSE Gamma-adducin OS=Mus musculus OX=10090 GN=Add3 PE=1 SV=1

31.71092 32.32654 31.87595 31.79169 32.3954 32.40357 32.38113 32.00357 alcohol m cell part;c Amino sug REACT_2 22 22 22 77.1 77.1 77.1 34.127 0 150.68 35.43359 234 16 16 18 15 18 18 18 17 1.149075795 0.796440882 0.369643211 2.1913223 Q9DCN2;F2Z45 Q9DCN2; NADH-cy tochrom Cy b5r3 sp|Q9DCN2|NB5R3_MOUSE NADH-cy tochrome b5 reductase 3 OS=Mus musculus OX=10090 GN=Cy b5r3 PE=1 SV=3;tr|F2Z456|F2Z456_MOUSE NADH-cy tochrome b5 reductase OS=Mus musculus OX=10090 GN=Cy b5r3 PE=1 SV=1

30.5601 30.46217 30.66425 30.51098 29.77731 30.22331 30.34499 30.47296 anatomica cell part;c Nitrogen mREACT_3 13 13 13 52.1 52.1 52.1 34.351 0 86.334 33.66321 125 13 12 13 11 11 12 11 12 1.148955414 0.795056 -0.344733238 -2.191121 Q64444;F6ST32 Q64444;F Carbonic anhy dras Ca4;Car4 sp|Q64444|CAH4_MOUSE Carbonic anhy drase 4 OS=Mus musculus OX=10090 GN=Ca4 PE=1 SV=1;tr|F6ST32|F6ST32_MOUSE Carbonate dehy dratase IV (Fragment) OS=Mus musculus OX=10090 GN=Car4 PE=1 SV=1

26.20576 26.1816 25.97655 26.31593 26.576 26.28909 26.36651 26.29995 cellular m cell part;cy toplasm;intracellular 12 12 12 29.9 29.9 29.9 66.031 0 35.573 29.59124 44 7 8 7 5 6 11 8 9 1.14869125 0.79401996 0.212928772 2.1906798 Q8N7N5;A0A0A Q8N7N5 DDB1- and CUL4- Dcaf 8 sp|Q8N7N5|DCAF8_MOUSE DDB1- and CUL4-associated f actor 8 OS=Mus musculus OX=10090 GN=Dcaf 8 PE=1 SV=1

23.86051 24.1035 23.56767 23.46986 23.466 20.15133 20.30784 23.16251 cell part 1 1 1 8 8 8 44.807 0 3.7978 26.17037 5 1 1 1 1 1 0 0 1 1.146371078 0.796756972 -1.978461742 -2.186804 Q8BGN5 Q8BGN5 NIPA-like protein 3 Nipal3 sp|Q8BGN5|NPAL3_MOUSE NIPA-like protein 3 OS=Mus musculus OX=10090 GN=Nipal3 PE=2 SV=1

26.20883 26.38037 26.10105 26.37615 26.39046 26.40467 27.26562 27.19826 biological cell part;c Adherens REACT_2 48 1 1 99.2 4.5 4.5 41.792 0 26.295 30.02828 10 1 1 1 1 1 1 1 1 1.146154463 0.795483101 0.548152447 2.1864417 P63260;B1ATY 1 P63260 Actin, cy toplasmic Actg1 sp|P63260|ACTG_MOUSE Actin, cy toplasmic 2 OS=Mus musculus OX=10090 GN=Actg1 PE=1 SV=1

30.4181 30.30465 30.7144 30.78986 30.19237 30.12497 30.43569 30.29822 anatomica cell part;cell surf ace; REACT_3 94 86 86 54.8 52 52 268.09 0 323.31 33.6208 443 69 71 73 65 42 58 60 55 1.145577247 0.794634921 -0.293941021 -2.185478 Q3UGX2;E9Q39 Q3UGX2; Spectrin beta chai Sptb tr|Q3UGX2|Q3UGX2_MOUSE Spectrin beta chain OS=Mus musculus OX=10090 GN=Sptb PE=1 SV=1;tr|E9Q397|E9Q397_MOUSE Spectrin beta chain OS=Mus musculus OX=10090 GN=Sptb PE=1 SV=1;sp|P15508|SPTB1_MOUSE Spectrin beta chain, ery throcy tic OS=Mus musculus OX=10090 GN=

24.60637 25.0068 24.46756 24.81807 25.46731 26.12957 24.94405 24.98351 alcohol m cell part;e N-Gly can REACT_2 8 8 8 10.9 10.9 10.9 131.63 0 17.753 28.51258 24 3 3 3 2 5 6 5 6 1.143986274 0.796807921 0.656408787 2.1828201 P27046;F6QMB7 P27046 Alpha-mannosidas Man2a1 sp|P27046|MA2A1_MOUSE Alpha-mannosidase 2 OS=Mus musculus OX=10090 GN=Man2a1 PE=1 SV=2

24.65048 24.25753 24.89352 24.99373 25.06032 25.07839 25.04181 25.04556 cell part;in RNA degr REACT_3 4 4 4 13 13 13 55.957 0 8.9946 28.00008 21 3 4 3 4 1 3 3 3 1.142900344 0.797320158 0.357702732 2.1810063 Q8K2D3 Q8K2D3 Enhancer of mRN Edc3 sp|Q8K2D3|EDC3_MOUSE Enhancer of mRNA-decapping protein 3 OS=Mus musculus OX=10090 GN=Edc3 PE=1 SV=1

24.09168 24.63683 24.20531 25.48373 23.74755 23.92624 23.83952 24.08318 2 2 2 84.6 84.6 84.6 5.3856 0 5.8711 27.41296 11 2 2 2 1 1 1 1 1 1.141457904 0.798311637 -0.705264091 -2.178597 A0A1B0GSF9 A0A1B0GSF9 **Gm19935** tr|A0A1B0GSF9|A0A1B0GSF9_MOUSE Predicted gene, 19935 OS=Mus musculus OX=10090 GN=Gm19935 PE=4 SV=1

28.37522 28.39517 28.19465 28.40361 28.17164 28.26808 28.0804 28.26674 catabolic cell part;c Protein pr REACT_3 22 22 22 54.8 54.8 54.8 68.016 0 72.259 31.53982 101 16 14 11 14 16 14 15 15 1.140190817 0.799519685 -0.145449162 -2.176482 P60670 P60670 Nuclear protein loc Nploc4 sp|P60670|NPL4_MOUSE Nuclear protein localization protein 4 homolog OS=Mus musculus OX=10090 GN=Nploc4 PE=1 SV=3

# Table S.:` List of differentially expressed proteins between control and *Pvalb-atg5KO-TdT* cells (p < 0.05).

| LFQ intensity | LFQ intensity | LFQ | LFQ | LFQ | LFQ | LFQ | LFQ | Student's T-test | Student's T- | GOBP | GOCC | KEGG | Reactom | Peptides | Razor + | Unique | Sequence | Unique + razor sequence | Unique sequence cov erage [%] | Mol. weight [kDa] | Q-v alue | Score | Intensity | **MS/MS** | Razor + | Razor + | Razor + | Razor + | Razor + | Razor + | Razor + | Razor + | -Log Student's | Student's T-test | Student's T-test | Student's T-test Test | Protein | Majority | **Protein names** | Gene names | Fasta |
| --- | --- | --- | --- | --- | --- | --- | --- | --- | --- | --- | --- | --- | --- | --- | --- | --- | --- | --- | --- | --- | --- | --- | --- | --- | --- | --- | --- | --- | --- | --- | --- | --- | --- | --- | --- | --- | --- | --- | --- | --- | --- |
| 13983-ctrl | 13984-ctrl | intensity 13985-  ctrl | intensity 13986-  ctrl | intensity 13987- KO | intensity 13988- KO | intensity 13989- KO | intensity 13990- KO | Signif icant KO_Ctrl | test  signif icant | slim name | slim name | name | e |  | unique peptides | peptides | cov erage [%] | cov erage [%] |  |  |  |  |  | **count** | unique peptides 13983-ctrl | unique peptides 13984-ctrl | unique peptides 13985-ctrl | unique peptides 13986-ctrl | unique peptides 13987-KO | unique peptides 13988-KO | unique peptides 13989-KO | unique peptides 13990-KO | T-test p-v alue KO_Ctrl | q-v alue KO_Ctrl | Dif f erence KO_Ctrl | statistic KO_Ctrl | IDs | protein IDs |  |  | headers |

23.77062035 23.47456932 24.00949 23.55039 20.42706 20.22727 20.23676 20.23835 + KO_Ctrl biological r cell part;intracellular membrane-bo 1 1 1 3.7 3.7 3.7 34.943 0 4.0677 25.73771 1 1 1 1 1 0 0 0 0 6.705578967 0 -3.418909073 -26.35396121 F6V6B6;Q8 F6V6B6;Q8 Protein BANP **Banp** tr|F6V6B6|F6V6B6_MOUSE Protein BANP (Fragment) OS=Mus musculus OX=10090 GN=Banp PE=1 SV=2;sp|Q8VBU8|BANP_MOUSE Protein BANP OS=Mus musculus OX=10090 GN=Banp PE=1 SV=1

19.24876213 18.87222481 19.43807 19.85525 23.656 24.03591 23.82852 24.02935 + KO_Ctrl biological r cell part;cyt ErbB signal REACT_288 2 2 2 8.4 8.4 8.4 59.312 0 5.4187 26.38306 5 0 0 0 0 2 1 2 2 6.027577032 0 4.533864975 20.263918 Q8CE90 Q8CE90 Dual specificity mitogen-activated protein kinase k **Map2k7** sp|Q8CE90|MP2K7_MOUSE Dual specificity mitogen-activated protein kinase kinase 7 OS=Mus musculus OX=10090 GN=Map2k7 PE=1 SV=1

23.61762047 23.97613716 23.84316 23.74622 19.47362 19.87977 20.43262 19.88661 + KO_Ctrl cell part;cytoplasm;intracellular me 2 2 2 7.3 7.3 7.3 35.274 0.004644 1.774 25.7985 2 1 1 1 1 0 0 1 0 5.777751583 0 -3.877631187 -18.386505 Q9D0D4 Q9D0D4 Probable dimethyladenosine transferase **Dimt1** sp|Q9D0D4|DIM1_MOUSE Probable dimethyladenosine transferase OS=Mus musculus OX=10090 GN=Dimt1 PE=2 SV=1

20.45552635 20.22158623 20.32584 20.57549 22.41596 22.43604 22.11092 22.30694 + KO_Ctrl biological r cell part;cytoplasm;cytoskeleton;int 3 3 3 7 7 7 94.316 0 4.3306 24.89601 9 2 1 0 0 3 2 2 2 5.716823681 0 1.9228549 17.95491373 Q5SSG4;F2 Q5SSG4 GAS2-like protein 2 **Gas2l2** sp|Q5SSG4|GA2L2_MOUSE GAS2-like protein 2 OS=Mus musculus OX=10090 GN=Gas2l2 PE=1 SV=1

30.4924736 30.46168137 32.38193 30.76793 20.1866 20.58055 20.79382 21.83113 + KO_Ctrl 4 4 4 5.2 5.2 5.2 136.23 0.008305 1.468 33.39777 3 2 2 2 1 1 2 1 0 5.674096748 0 -10.17798042 -17.65812007 Q3U3C9 Q3U3C9 Genetic suppressor element 1 **Gse1** sp|Q3U3C9|GSE1_MOUSE Genetic suppressor element 1 OS=Mus musculus OX=10090 GN=Gse1 PE=1 SV=2

19.43297005 20.14211845 19.44708 20.59836 24.34699 24.20284 24.24743 24.10981 + KO_Ctrl biological r cell part;intracellular m REACT_282 4 4 4 18.4 18.4 18.4 42.554 0 10.609 26.70372 8 0 2 0 0 3 3 3 2 5.25403581 0 4.321633816 14.9810431 P19426;G3 P19426;G3 Negative elongation factor E **Nelfe** sp|P19426|NELFE_MOUSE Negative elongation factor E OS=Mus musculus OX=10090 GN=Nelfe PE=1 SV=2;tr|G3UY39|G3UY39_MOUSE Negative elongation factor E (Fragment) OS=Mus musculus OX=10090 GN=Nelfe PE=1 SV=1

20.23049927 21.60172653 20.94029 20.54033 25.09889 25.3772 25.38215 25.32776 + KO_Ctrl cell part;extracellular organelle;mem 7 7 7 29 29 29 46.201 0 15.131 28.34075 6 4 4 3 3 2 3 4 2 5.210242084 0 4.468287468 14.72549345 Q8K2Q7;A Q8K2Q7 BRO1 domain-containing protein BROX **Brox** sp|Q8K2Q7|BROX_MOUSE BRO1 domain-containing protein BROX OS=Mus musculus OX=10090 GN=Brox PE=1 SV=1

25.7899971 25.57905579 25.1563 24.85548 20.36623 20.44236 19.64469 20.94786 + KO_Ctrl anatomical cell part;cel Adherens junction;Bact 4 3 3 14.7 12.3 12.3 61.508 0 6.8994 27.5451 4 2 1 3 2 1 0 0 1 5.200115315 0 -4.994924545 -14.66699515 Q8R5H6 Q8R5H6 Wiskott-Aldrich syndrome protein family member **Wasf1** sp|Q8R5H6|WASF1_MOUSE Wiskott-Aldrich syndrome protein family member 1 OS=Mus musculus OX=10090 GN=Wasf1 PE=1 SV=2

25.44268799 25.41044426 25.6725 25.83615 21.24265 19.80773 19.71467 19.94933 + KO_Ctrl 4 4 4 61.9 61.9 61.9 11.546 0 10.094 27.52425 6 3 4 3 4 0 0 0 0 5.180714305 0 -5.411850929 -14.55554095 Q9CRC6 Q9CRC6 UPF0693 protein C10orf32 homolog sp|Q9CRC6|BORC7_MOUSE BLOC-1-related complex subunit 7 OS=Mus musculus OX=10090 GN=Borcs7 PE=1 SV=1

23.76212883 24.3448925 24.53249 23.70725 20.04198 20.67945 19.73948 19.98771 + KO_Ctrl biological r cell part;cytoplasmic m REACT_343 4 4 4 8.1 8.1 8.1 82.79 0 15.184 26.37171 5 1 2 3 1 0 1 1 0 5.04478679 0 -3.974534988 -13.79694736 Q80WG7;E Q80WG7;E E3 ubiquitin-protein ligase Trim36 **Trim36** sp|Q80WG7|TRI36_MOUSE E3 ubiquitin-protein ligase Trim36 OS=Mus musculus OX=10090 GN=Trim36 PE=1 SV=2;tr|E9Q3A0|E9Q3A0_MOUSE E3 ubiquitin-protein ligase Trim36 OS=Mus musculus OX=10090 GN=Trim36 PE=1 SV=1;tr|A0A494B9M7|A0A494B9M7_MOUSE E3 ubiquitin-protein

24.07144737 23.7875576 23.78616 23.48431 20.55931 20.70005 21.07911 20.10386 + KO_Ctrl anatomical cell part;cel Histidine metabolism 2 2 2 11.9 11.9 11.9 33.664 0 6.985 26.90185 3 1 1 1 1 0 1 1 2 4.995452395 0 -3.171787262 -13.53100645 Q91VF2 Q91VF2 Histamine N-methyltransferase **Hnmt** sp|Q91VF2|HNMT_MOUSE Histamine N-methyltransferase OS=Mus musculus OX=10090 GN=Hnmt PE=1 SV=1

23.17910194 23.64339447 23.58795 23.71461 21.12309 20.53921 20.43202 20.24417 + KO_Ctrl carbohydra cell part;me Glycosphin REACT_310 4 4 4 23.9 23.9 23.9 35.414 0 5.6812 26.20631 6 3 2 1 2 1 1 2 1 4.918779923 0 -2.946641922 -13.12728371 Q9WUV2;A Q9WUV2;A Alpha-N-acetylgalactosaminide alpha-2,6-sialyltran **St6galnac3** sp|Q9WUV2|SIA7C_MOUSE Alpha-N-acetylgalactosaminide alpha-2,6-sialyltransferase 3 OS=Mus musculus OX=10090 GN=St6galnac3 PE=1 SV=2;tr|A0A0H2UKC4|A0A0H2UKC4_MOUSE Alpha-N-acetylgalactosaminide alpha-2,6-sialyltransferase 3 (Fragment) OS=Mus musculus OX=1009

21.46049309 21.50611877 19.96153 20.80542 25.91775 25.50127 25.75901 25.91239 + KO_Ctrl biological r cell part;intracellular m REACT_277 4 4 4 8.2 8.2 8.2 92.632 0 23.604 28.26548 3 1 2 1 0 2 1 1 1 4.880280558 0 4.839215755 12.92886839 Q8CIG3;F6 Q8CIG3;F6 Lysine-specific histone demethylase 1B **Kdm1b** sp|Q8CIG3|KDM1B_MOUSE Lysine-specific histone demethylase 1B OS=Mus musculus OX=10090 GN=Kdm1b PE=1 SV=1;tr|F6V3V2|F6V3V2_MOUSE Lysine-specific histone demethylase 1B (Fragment) OS=Mus musculus OX=10090 GN=Kdm1b PE=1 SV=1

20.16018677 20.31419945 20.96852 19.76841 23.87173 24.78616 24.23579 24.36833 + KO_Ctrl anatomical cell part;cytoplasm;intr REACT_291 5 5 5 14.3 14.3 14.3 63.053 0 7.3383 27.15991 7 1 1 1 1 3 4 2 2 4.85765267 0 4.012675762 12.81356902 A0A286YD A0A286YD Elongator complex protein 3 **Elp3** tr|A0A286YDB8|A0A286YDB8_MOUSE Elongator complex protein 3 OS=Mus musculus OX=10090 GN=Elp3 PE=1 SV=1;sp|Q9CZX0|ELP3_MOUSE Elongator complex protein 3 OS=Mus musculus OX=10090 GN=Elp3 PE=1 SV=1

20.8896637 21.50396538 20.50443 20.9035 24.05167 23.67691 23.83798 23.65578 + KO_Ctrl biosynthet cell part;intracellular membrane-bo 2 2 2 3.3 3.3 3.3 96.382 0 3.973 26.44444 3 0 0 0 0 1 2 1 2 4.82421093 0 2.855196953 12.64493352 V9GWX3;Q V9GWX3;Q RNA polymerase II-associated protein 1 **Rpap1** tr|V9GWX3|V9GWX3_MOUSE RNA polymerase II-associated protein 1 OS=Mus musculus OX=10090 GN=Rpap1 PE=1 SV=1;sp|Q80TE0|RPAP1_MOUSE RNA polymerase II-associated protein 1 OS=Mus musculus OX=10090 GN=Rpap1 PE=1 SV=2

20.71244431 21.19105721 20.2771 20.68534 23.32882 23.10074 23.19191 23.46538 + KO_Ctrl biological r cell part;membrane 3 3 3 7.9 7.9 7.9 67.487 0 5.8282 25.77992 3 0 0 0 0 1 1 3 1 4.809823812 0 2.555227757 12.57302593 A0A2R8W6 A0A2R8W6 La-related protein 4 **Larp4** tr|A0A2R8W6Y5|A0A2R8W6Y5_MOUSE La-related protein 4 OS=Mus musculus OX=10090 GN=Larp4 PE=1 SV=1;tr|A0A2R8VKL5|A0A2R8VKL5_MOUSE La-related protein 4 OS=Mus musculus OX=10090 GN=Larp4 PE=1 SV=1;tr|E9Q066|E9Q066_MOUSE La-related protein 4 OS=Mus musculus OX=1

20.34271049 20.1870327 20.70218 19.75355 25.30281 24.111 24.18382 24.31679 + KO_Ctrl biological r cell part;en p53 signali REACT_290 2 2 2 5.9 5.9 5.9 54.747 0.004235 1.875 26.68672 2 0 0 0 0 2 2 1 2 4.781149192 0 4.232235909 12.43084873 A0A0R4J1GA0A0R4J1GMetalloreductase STEAP3 **Steap3** tr|A0A0R4J1G9|A0A0R4J1G9_MOUSE Metalloreductase STEAP3 OS=Mus musculus OX=10090 GN=Steap3 PE=1 SV=1;tr|E9QN92|E9QN92_MOUSE Metalloreductase STEAP3 OS=Mus musculus OX=10090 GN=Steap3 PE=1 SV=1;sp|Q8CI59|STEA3_MOUSE Metalloreductase STEAP3 OS=Mus musculus OX

20.34236908 20.74874115 19.34951 20.53098 24.49546 24.3319 24.01095 24.61807 + KO_Ctrl cell part;endoplasmic reticulum;intr 2 2 2 12.2 12.2 12.2 58.507 0 4.0991 26.90736 2 0 1 0 0 1 1 1 1 4.747717865 0.002444444 4.121191978 12.26698413 Q99J23 Q99J23 GH3 domain-containing protein **Ghdc** sp|Q99J23|GHDC_MOUSE GH3 domain-containing protein OS=Mus musculus OX=10090 GN=Ghdc PE=2 SV=2

23.50142479 23.81860542 23.92823 23.94878 20.79448 19.39289 19.7955 20.06021 + KO_Ctrl cell part;cell surface;extracellular or 2 2 2 7 7 7 50.555 0 3.7937 25.75929 2 1 2 1 1 0 0 0 0 4.717008911 0.002315789 -3.788486481 -12.11824156 Q80V42 Q80V42 Carboxypeptidase M **Cpm** sp|Q80V42|CBPM_MOUSE Carboxypeptidase M OS=Mus musculus OX=10090 GN=Cpm PE=1 SV=2

21.65784836 20.45513916 19.90482 19.76704 25.70749 25.69365 25.6876 25.5616 + KO_Ctrl biological r cell part;cytoplasm;endosome;intra 3 3 3 4.2 4.2 4.2 115.77 0 6.5397 28.25156 7 1 1 1 0 1 2 2 2 4.709776163 0.0022 5.216374397 12.08345427 E9QMX4;Q E9QMX4;Q Ribosomal protein S6 kinase delta-1 **Rps6kc1** tr|E9QMX4|E9QMX4_MOUSE Non-specific serine/threonine protein kinase OS=Mus musculus OX=10090 GN=Rps6kc1 PE=1 SV=1;sp|Q8BLK9|KS6C1_MOUSE Ribosomal protein S6 kinase delta-1 OS=Mus musculus OX=10090 GN=Rps6kc1 PE=1 SV=2

26.0237751 24.99261093 25.02378 25.55256 20.19153 21.08622 19.66109 20.9935 + KO_Ctrl anatomical cell part;cyt Regulation REACT_289 4 4 4 32.4 32.4 32.4 32.402 0 13.874 27.42037 13 4 4 3 3 0 0 0 0 4.636409863 0.002095238 -4.91509676 -11.73579549 Q99J83 Q99J83 Autophagy protein 5 **Atg5** sp|Q99J83|ATG5_MOUSE Autophagy protein 5 OS=Mus musculus OX=10090 GN=Atg5 PE=1 SV=1

20.2451725 20.10064507 20.8374 20.73649 26.22597 25.82607 25.70844 24.34469 + KO_Ctrl biological r cell part;cyt mRNA surv REACT_279 1 1 1 3.2 3.2 3.2 75.769 0.004501 1.7871 28.11515 3 0 0 0 0 1 1 1 1 4.537294151 0.002 5.046363831 11.28082713 Q8K3W3 Q8K3W3 Protein CASC3 **Casc3** sp|Q8K3W3|CASC3_MOUSE Protein CASC3 OS=Mus musculus OX=10090 GN=Casc3 PE=1 SV=3

20.39159203 19.80899811 20.63519 19.52756 24.60947 23.796 24.6903 23.67336 + KO_Ctrl biological r extracellular organelle;extracellular s 11 3 3 35.2 10.5 10.5 46.879 0 7.5086 26.70359 5 0 0 0 0 2 3 3 3 4.501830503 0.001913043 4.101448536 11.12203285 P07759 P07759 Serine protease inhibitor A3K **Serpina3k** sp|P07759|SPA3K_MOUSE Serine protease inhibitor A3K OS=Mus musculus OX=10090 GN=Serpina3k PE=1 SV=2

20.27791214 19.93434715 20.49174 21.17364 23.60128 23.72775 23.28107 23.56871 + KO_Ctrl metabolic p cell part;cytoplasm;extracellular org 4 4 4 22.5 22.5 22.5 24.443 0 3.7943 26.19126 7 0 1 0 1 2 3 2 2 4.48999472 0.001833333 3.075294018 11.06949552 Q9D142;A Q9D142;A Uridine diphosphate glucose pyrophosphatase **Nudt14** sp|Q9D142|NUD14_MOUSE Uridine diphosphate glucose pyrophosphatase NUDT14 OS=Mus musculus OX=10090 GN=Nudt14 PE=1 SV=1;tr|A0A217FL49|A0A217FL49_MOUSE Uridine diphosphate glucose pyrophosphatase NUDT14 OS=Mus musculus OX=10090 GN=Nudt14 PE=1 SV=1

22.45948982 22.88082314 22.89567 22.36469 20.12964 19.69584 20.19645 20.57897 + KO_Ctrl biological r cell part;intracellular membrane-bo 2 2 2 2 2 2 246.7 0.000164 3.0005 24.74945 3 2 1 1 1 0 0 0 1 4.465493874 0.00176 -2.499941826 -10.96146327 E9Q507;K4 E9Q507;K4 GON-4-like protein **Gon4l** tr|E9Q507|E9Q507_MOUSE GON-4-like protein OS=Mus musculus OX=10090 GN=Gon4l PE=1 SV=1;tr|K4DI71|K4DI71_MOUSE GON-4-like protein OS=Mus musculus OX=10090 GN=Gon4l PE=1 SV=1;sp|Q9DB00|GON4L_MOUSE GON-4-like protein OS=Mus musculus OX=10090 GN=Gon4l PE=1 SV=3

20.77942848 21.20188332 20.33858 20.58886 22.78257 22.93885 22.69534 22.65417 + KO_Ctrl anatomical cell part;cyt Calcium sig REACT_321 1 1 1 3.2 3.2 3.2 52.377 0.00693 1.5966 25.26116 2 0 0 0 0 1 1 1 1 4.378619607 0.003384615 2.040545464 10.58614007 F7AAE0;Q8 F7AAE0;Q8 Phosphoinositide phospholipase C;1-phosphatid **Plcd3** tr|F7AAE0|F7AAE0_MOUSE Phosphoinositide phospholipase C (Fragment) OS=Mus musculus OX=10090 GN=Plcd3 PE=1 SV=1;sp|Q8K2J0|PLCD3_MOUSE 1-phosphatidylinositol 4,5-bisphosphate phosphodiesterase delta-3 OS=Mus musculus OX=10090 GN=Plcd3 PE=1 SV=2

23.60738373 23.74949837 24.98007 24.30744 20.55938 20.97726 20.92064 20.88424 + KO_Ctrl biological a cell part;cel Arrhythmo REACT_307 3 3 3 4.5 4.5 4.5 112.68 0 8.5893 26.34099 5 2 2 3 2 0 0 0 0 4.287824665 0.008148148 -3.325715065 -10.20642711 Q9QUM0 Q9QUM0 Integrin alpha-IIb;Integrin alpha-IIb heavy chain;Int **Itga2b** sp|Q9QUM0|ITA2B_MOUSE Integrin alpha-IIb OS=Mus musculus OX=10090 GN=Itga2b PE=1 SV=2

19.30158615 19.76436615 20.6989 19.83308 24.30459 24.33429 24.18359 23.17072 + KO_Ctrl anatomical cell body;cell part;cell projection;m 9 9 9 8.3 8.3 8.3 200.06 0 4.271 27.17874 12 4 2 0 0 2 4 2 3 4.281544937 0.007857143 4.098815441 10.18062726 E9QAF9;Q0 E9QAF9;Q0 Protein TANC1 **Tanc1** tr|E9QAF9|E9QAF9_MOUSE Protein TANC1 OS=Mus musculus OX=10090 GN=Tanc1 PE=1 SV=1;sp|Q0VGY8|TANC1_MOUSE Protein TANC1 OS=Mus musculus OX=10090 GN=Tanc1 PE=1 SV=2

25.50445747 25.87734032 26.39632 25.50624 21.5926 20.44213 19.42048 21.05728 + KO_Ctrl biosynthet cell part;me Glyceropho REACT_292 1 1 1 4.2 4.2 4.2 55.603 0.002322 2.1657 27.97842 1 1 1 1 1 0 0 0 0 4.274179055 0.007586207 -5.192968369 -10.15044021 Q99LH2 Q99LH2 Phosphatidylserine synthase 1 **Ptdss1** sp|Q99LH2|PTSS1_MOUSE Phosphatidylserine synthase 1 OS=Mus musculus OX=10090 GN=Ptdss1 PE=1 SV=1

20.34203339 19.50029564 20.45849 19.43405 23.05051 22.5906 22.98557 23.19187 + KO_Ctrl anatomical cell part;cel Allograft re REACT_300 4 1 0 14.1 4.3 0 37.41 0.005068 1.7206 25.38479 1 0 0 0 0 1 1 1 1 4.256018593 0.007333333 3.020916939 10.07635984 P14430;E9 P14430;E9 H-2 class I histocompatibility antigen, Q8 alpha ch **H2-Q8;H2-Q7;H2-Q6;H2-Q9** sp|P14430|HA18_MOUSE H-2 class I histocompatibility antigen, Q8 alpha chain OS=Mus musculus OX=10090 GN=H2-Q8 PE=3 SV=1;tr|E9PWT4|E9PWT4_MOUSE H-2 class I histocompatibility antigen, Q7 alpha chain OS=Mus musculus OX=10090 GN=H2-Q7 PE=3 SV=2;tr|E9QJR9|E9QJ

19.40817261 21.02241898 20.10306 20.22212 25.0471 24.11791 24.11593 24.00941 + KO_Ctrl response t cell part Arachidoni REACT_308 3 3 3 23.9 23.9 23.9 24.148 0.000635 2.5206 26.76395 5 1 0 0 0 2 1 2 1 4.255478022 0.007096774 4.133644104 10.07416224 Q9D7B7 Q9D7B7 Probable glutathione peroxidase 8 **Gpx8** sp|Q9D7B7|GPX8_MOUSE Probable glutathione peroxidase 8 OS=Mus musculus OX=10090 GN=Gpx8 PE=1 SV=1

22.61178017 22.79110146 22.48734 22.09381 20.84937 20.47259 20.74079 20.91656 + KO_Ctrl biological r cell part;cyt Bladder can REACT_313 2 1 1 6.7 3.5 3.5 56.135 0.004229 1.8681 24.52307 1 1 1 1 1 0 0 0 0 4.207678356 0.0085 -1.751177788 -9.881540535 E9JGN0;Q8 E9JGN0;Q8 Death-associated protein kinase 2 **Dapk2** tr|E9JGN0|E9JGN0_MOUSE DAP-kinase-related protein 1 beta isoform OS=Mus musculus OX=10090 GN=Dapk2 PE=1 SV=1;sp|Q8VDF3|DAPK2_MOUSE Death-associated protein kinase 2 OS=Mus musculus OX=10090 GN=Dapk2 PE=1 SV=1

19.7934494 19.96064186 21.37589 20.1433 24.4025 23.7543 24.15599 24.43841 + KO_Ctrl anatomical cell part;centrosome;in REACT_286 5 5 5 9.1 9.1 9.1 110.09 0 12.749 26.79501 6 1 1 0 1 1 4 3 2 4.200793478 0.008242424 3.869479179 9.854070941 Q80SY4;F6 Q80SY4;F6 E3 ubiquitin-protein ligase MIB1 **Mib1** sp|Q80SY4|MIB1_MOUSE E3 ubiquitin-protein ligase MIB1 OS=Mus musculus OX=10090 GN=Mib1 PE=1 SV=1;tr|F6ZBL2|F6ZBL2_MOUSE RING-type E3 ubiquitin transferase (Fragment) OS=Mus musculus OX=10090 GN=Mib1 PE=1 SV=1

18.6766758 20.20793152 19.38173 19.23711 22.73136 23.05623 22.37984 22.87455 + KO_Ctrl biological r cell part;macromolecular complex;p 3 3 3 13.8 13.8 13.8 42.354 0.006643 1.606 25.97695 3 2 1 1 0 1 1 1 1 4.174025527 0.008 3.384632111 9.747921436 Q8BUY9;A0 Q8BUY9;A0 Geranylgeranyl transferase type-1 subunit beta **Pggt1b** sp|Q8BUY9|PGTB1_MOUSE Geranylgeranyl transferase type-1 subunit beta OS=Mus musculus OX=10090 GN=Pggt1b PE=1 SV=1;tr|A0A494BA07|A0A494BA07_MOUSE Geranylgeranyl transferase type-1 subunit beta OS=Mus musculus OX=10090 GN=Pggt1b PE=1 SV=1

25.02453613 24.52569962 24.92873 24.86429 18.99915 20.34118 19.5888 21.22073 + KO_Ctrl biological regulation;cellular process;response t 2 2 2 6.1 6.1 6.1 116.24 0 10.43 26.83714 4 2 2 1 2 0 0 0 0 4.171759688 0.007771429 -4.79834938 -9.738983421 Q99MR1;A Q99MR1;A PERQ amino acid-rich with GYF domain-containing **Gigyf1** sp|Q99MR1|GGYF1_MOUSE GRB10-interacting GYF protein 1 OS=Mus musculus OX=10090 GN=Gigyf1 PE=1 SV=2;tr|A0A0G2JGR7|A0A0G2JGR7_MOUSE GRB10-interacting GYF protein 1 (Fragment) OS=Mus musculus OX=10090 GN=Gigyf1 PE=1 SV=1

24.82590103 24.92605782 24.86835 25.05109 21.0721 21.69309 19.6595 20.72869 + KO_Ctrl cellular comcell part;intracellular membrane-bo 7 7 7 18.8 18.8 18.8 68.171 0 47.942 26.7497 15 4 6 3 4 0 1 0 0 4.144504496 0.007555556 -4.129500866 -9.632044084 Q8C0J2;D3 Q8C0J2;D3 Autophagy-related protein 16-1 **Atg16l1** sp|Q8C0J2|A16L1_MOUSE Autophagy-related protein 16-1 OS=Mus musculus OX=10090 GN=Atg16l1 PE=1 SV=1;tr|D3YZW7|D3YZW7_MOUSE Autophagy-related protein 16-1 (Fragment) OS=Mus musculus OX=10090 GN=Atg16l1 PE=1 SV=1

20.40773201 20.97622108 20.18011 19.79838 23.38337 24.14585 23.1965 23.2832 + KO_Ctrl anatomical cell part;cell projection; REACT_272 4 4 4 2.4 2.4 2.4 289.07 0.001866 2.2273 26.29957 4 0 0 0 0 2 2 1 2 4.139544691 0.007351351 3.16161871 9.612697036 Q6A078;E9 Q6A078;E9 Centrosomal protein of 290 kDa **Cep290** sp|Q6A078|CE290_MOUSE Centrosomal protein of 290 kDa OS=Mus musculus OX=10090 GN=Cep290 PE=1 SV=2;tr|E9Q9M0|E9Q9M0_MOUSE Centrosomal protein of 290 kDa OS=Mus musculus OX=10090 GN=Cep290 PE=1 SV=1

24.13547516 23.57600212 24.8597 23.71177 20.07889 20.99465 20.59611 19.87459 + KO_Ctrl cell death;c cell part;macromolecular complex;p 2 2 2 13.7 13.7 13.7 20.116 0.006213 1.6382 26.48932 5 2 2 2 1 0 0 1 1 4.134255687 0.007157895 -3.684676647 -9.592104135 Q9CZX9 Q9CZX9 ER membrane protein complex subunit 4 **Emc4** sp|Q9CZX9|EMC4_MOUSE ER membrane protein complex subunit 4 OS=Mus musculus OX=10090 GN=Emc4 PE=1 SV=1

23.42713547 23.85386086 23.76091 23.92533 21.15326 19.73049 20.37249 19.61962 + KO_Ctrl anatomical cell part;cyt Basal trans REACT_279 2 2 2 9.7 9.7 9.7 35.848 0.001262 2.4553 25.66457 2 1 1 2 2 0 0 0 1 4.118135789 0.006974359 -3.522845268 -9.529583616 P51949;A0 P51949;A0 CDK-activating kinase assembly factor MAT1 **Mnat1** sp|P51949|MAT1_MOUSE CDK-activating kinase assembly factor MAT1 OS=Mus musculus OX=10090 GN=Mnat1 PE=1 SV=2;tr|A0A087WSQ7|A0A087WSQ7_MOUSE CDK-activating kinase assembly factor MAT1 OS=Mus musculus OX=10090 GN=Mnat1 PE=1 SV=1;tr|A0A087WSG5|A0A087WSG5_MOUSE

20.29174423 19.34304428 20.14296 19.84513 22.76967 22.32405 22.52597 23.29936 + KO_Ctrl alcohol me cell part REACT_294 2 2 2 4 4 4 79.582 0.007464 1.5214 25.08582 2 0 0 0 0 1 1 2 1 4.113594102 0.0068 2.824043751 9.512034614 Q9JKZ2 Q9JKZ2 Sodium/myo-inositol cotransporter **Slc5a3** sp|Q9JKZ2|SC5A3_MOUSE Sodium/myo-inositol cotransporter OS=Mus musculus OX=10090 GN=Slc5a3 PE=1 SV=2

20.3611908 19.94425774 20.97083 20.29634 24.28926 23.01995 23.45438 23.72265 + KO_Ctrl biological r cell part;cel Inositol ph REACT_272 2 2 2 5.8 5.8 5.8 54.158 0.009867 1.4163 27.42037 2 1 1 1 1 1 1 1 1 4.105402113 0.006634146 3.228402615 9.480453747 Q8C5L6;Q5 Q8C5L6;Q5 Inositol polyphosphate 5-phosphatase K **Inpp5k** sp|Q8C5L6|INP5K_MOUSE Inositol polyphosphate 5-phosphatase K OS=Mus musculus OX=10090 GN=Inpp5k PE=1 SV=2;tr|Q5ND44|Q5ND44_MOUSE Inositol polyphosphate 5-phosphatase K (Fragment) OS=Mus musculus OX=10090 GN=Inpp5k PE=1 SV=1

23.54498291 23.29572105 23.53112 24.04469 21.0698 20.84446 19.82096 20.16593 + KO_Ctrl alcohol me cell part;cyt Glycolysis / REACT_314 2 1 1 5.5 3.5 3.5 58.82 0.000163 2.9653 25.54984 2 1 1 1 1 0 0 0 0 4.101113111 0.00647619 -3.128841877 -9.46395655 E9Q509;G3 E9Q509;G3 Pyruvate kinase;Pyruvate kinase PKLR **Pklr** tr|E9Q509|E9Q509_MOUSE Pyruvate kinase OS=Mus musculus OX=10090 GN=Pklr PE=1 SV=1;tr|G3X925|G3X925_MOUSE Pyruvate kinase OS=Mus musculus OX=10090 GN=Pklr PE=1 SV=1;sp|P53657|KPYR_MOUSE Pyruvate kinase PKLR OS=Mus musculus OX=10090 GN=Pklr PE=1 SV=1;tr|D3Z2

23.43529892 23.51376343 22.79269 23.79501 21.11765 20.90062 20.42377 20.32163 + KO_Ctrl behavior;bi cell part;cel ABC transp REACT_346 1 1 1 1.1 1.1 1.1 237.75 0.000164 3.0021 25.28132 1 1 1 1 1 0 0 0 0 4.101083546 0.006325581 -2.693272114 -9.463842918 E9Q6G4;Q E9Q6G4;Q ATP-binding cassette sub-family A member 7 **Abca7** tr|E9Q6G4|E9Q6G4_MOUSE ATP-binding cassette sub-family A member 7 OS=Mus musculus OX=10090 GN=Abca7 PE=1 SV=1;sp|Q91V24|ABCA7_MOUSE ATP-binding cassette sub-family A member 7 OS=Mus musculus OX=10090 GN=Abca7 PE=1 SV=1

21.05871391 19.37294197 20.47064 20.27798 23.69424 24.18162 23.58187 24.1795 + KO_Ctrl cellular metabolic process;cellular process;meta 5 5 5 11.8 11.8 11.8 63.858 0 6.3921 27.01829 9 1 1 1 1 1 2 4 3 4.090546522 0.006181818 3.614237785 9.423422286 A0A2I3BR8 A0A2I3BR81;Q91X76;A0A2I3BQR1;A0A2I3BRL0 **Nt5dc2** tr|A0A2I3BR81|A0A2I3BR81_MOUSE 5-nucleotidase domain-containing 2 OS=Mus musculus OX=10090 GN=Nt5dc2 PE=1 SV=1;tr|Q91X76|Q91X76_MOUSE 5-nucleotidase domain-containing 2 OS=Mus musculus OX=10090 GN=Nt5dc2 PE=1 SV=1;tr|A0A2I3BQR1|A0A2I3BQR1_MOUSE 5-nucleo

24.02647972 23.90784073 23.60784 23.89717 20.71085 21.66061 20.3113 20.25468 + KO_Ctrl biological r cell part;intracellular membrane-bo 3 3 3 9 9 9 58.867 0 4.5184 25.9162 2 1 1 1 1 0 1 1 0 4.05161128 0.006044444 -3.125471115 -9.275392395 Q91W36;E Q91W36;E Ubiquitin carboxyl-terminal hydrolase 3;Ubiquitin **Usp3** sp|Q91W36|UBP3_MOUSE Ubiquitin carboxyl-terminal hydrolase 3 OS=Mus musculus OX=10090 GN=Usp3 PE=2 SV=1;tr|E9Q8W9|E9Q8W9_MOUSE Ubiquitin carboxyl-terminal hydrolase OS=Mus musculus OX=10090 GN=Usp3 PE=1 SV=1;tr|G3UZF0|G3UZF0_MOUSE Ubiquitin carboxyl-termin

23.31617165 23.55484962 23.56023 23.52552 20.56948 20.44231 18.89794 20.12268 + KO_Ctrl anatomical cell part;cyt MAPK sign REACT_301 6 3 3 14.2 6.4 6.4 55.541 0.00202 2.2179 26.12524 3 1 1 2 1 0 1 1 1 3.98136648 0.010173913 -3.481090546 -9.013526501 Q9JI11;Q8 Q9JI11 Serine/threonine-protein kinase 4;Serine/threonin **Stk4** sp|Q9JI11|STK4_MOUSE Serine/threonine-protein kinase 4 OS=Mus musculus OX=10090 GN=Stk4 PE=1 SV=1

23.76830101 23.21127701 23.59685 23.54851 21.7713 21.28005 20.66037 21.21014 + KO_Ctrl biological r cell part;cytoplasm;intracellular me 3 3 3 35.7 35.7 35.7 22.656 0 7.4909 26.60428 5 1 1 2 1 0 1 1 0 3.978745537 0.009957447 -2.300767899 -9.003883424 O88843 O88843 Death domain-containing protein CRADD **Cradd** sp|O88843|CRADD_MOUSE Death domain-containing protein CRADD OS=Mus musculus OX=10090 GN=Cradd PE=1 SV=2

23.53824806 24.02216721 24.1888 22.90514 21.02552 20.77086 21.17044 20.81687 + KO_Ctrl cellular met cell part;cytoplasm;intracellular me 3 3 3 6.6 6.6 6.6 74.331 0 4.5618 27.11734 4 1 2 2 2 1 1 1 1 3.971832558 0.00975 -2.717667103 -8.978492446 Q8VEG4;A0 Q8VEG4;A0 Exonuclease 3-5 domain-containing protein 2 **Exd2** sp|Q8VEG4|EXD2_MOUSE Exonuclease 3-5 domain-containing protein 2 OS=Mus musculus OX=10090 GN=Exd2 PE=1 SV=2;tr|A0A1W2P7Q1|A0A1W2P7Q1_MOUSE Exonuclease 3-5 domain-containing protein 2 (Fragment) OS=Mus musculus OX=10090 GN=Exd2 PE=1 SV=1

21.42704201 21.44332695 20.71926 19.76252 24.98542 24.30932 24.7434 24.48763 + KO_Ctrl biological a cell part;cell projection;cytoplasm;i 2 2 2 8.3 8.3 8.3 43.478 0 10.768 27.19657 4 0 0 0 0 1 2 2 2 3.969346609 0.00955102 3.793403149 8.969377105 Q99N69 Q99N69 Leupaxin **Lpxn** sp|Q99N69|LPXN_MOUSE Leupaxin OS=Mus musculus OX=10090 GN=Lpxn PE=1 SV=2

23.30799866 23.41854095 23.3254 24.35844 20.86574 19.46901 20.23508 20.26803 + KO_Ctrl cell part;intracellular membrane-bo 2 2 2 14.9 14.9 14.9 22.178 0 3.9892 25.39382 6 2 1 1 2 0 0 1 0 3.945021488 0.0104 -3.393128395 -8.880611182 Q9CR10;H Q9CR10;H Oxidoreductase-like domain-containing protein 1 **Oxld1** sp|Q9CR10|OXLD1_MOUSE Oxidoreductase-like domain-containing protein 1 OS=Mus musculus OX=10090 GN=Oxld1 PE=1 SV=1;tr|H3BK04|H3BK04_MOUSE Oxidoreductase-like domain-containing protein 1 (Fragment) OS=Mus musculus OX=10090 GN=Oxld1 PE=1 SV=8

23.12026978 24.26806641 24.08278 23.17579 20.11472 20.73951 19.98362 20.84016 + KO_Ctrl establishm cell part;macromolecul REACT_292 2 2 2 13.7 13.7 13.7 20.749 0 4.7269 25.85017 2 2 2 2 2 0 0 0 0 3.91709071 0.012470588 -3.242221355 -8.779638513 A0A2I3BS4 A0A2I3BS4 Mitochondrial import inner membrane translocas  **Timm23** tr|A0A2I3BS49|A0A2I3BS49_MOUSE Mitochondrial import inner membrane translocase subunit Tim23 OS=Mus musculus OX=10090 GN=Timm23 PE=1 SV=1;tr|Q9CXU4|Q9CXU4_MOUSE Mitochondrial import inner membrane translocase subunit TIM23 OS=Mus musculus OX=10090 GN=Timm2

21.14826393 22.27550316 21.29392 21.68699 23.735 24.16967 24.15182 23.85576 + KO_Ctrl cellular pro cell part;Golgi apparatus;intracellul 9 9 9 16.4 16.4 16.4 87.337 0 39.002 27.06928 13 3 2 2 3 2 5 5 4 3.885570836 0.013307692 2.376896381 8.666897569 Q9CW79;A Q9CW79 Golgin subfamily A member 1 **Golga1** sp|Q9CW79|GOGA1_MOUSE Golgin subfamily A member 1 OS=Mus musculus OX=10090 GN=Golga1 PE=1 SV=2

19.76926041 20.12755394 21.65599 20.58333 23.89541 24.34442 24.15182 24.34807 + KO_Ctrl biological r cell part;en Amyotrophic lateral scl 1 1 1 4.4 4.4 4.4 28.834 0.000636 2.5471 26.72299 2 0 0 0 0 1 1 1 1 3.874801278 0.013056604 3.650896072 8.628667227 Q99J56 Q99J56 Derlin-1 **Derl1** sp|Q99J56|DERL1_MOUSE Derlin-1 OS=Mus musculus OX=10090 GN=Derl1 PE=1 SV=1

20.29405212 19.73694801 20.95164 18.97851 23.5354 23.48296 23.73965 23.66405 + KO_Ctrl 3 3 3 9.2 9.2 9.2 49.401 0.000164 3.026 26.02333 5 0 0 0 0 1 2 2 3 3.852376073 0.012814815 3.61523056 8.54953152 D3Z286 D3Z286 **Tmppe** tr|D3Z286|D3Z286_MOUSE Transmembrane protein with metallophosphoesterase domain OS=Mus musculus OX=10090 GN=Tmppe PE=1 SV=2

22.26407623 22.24394417 21.88937 21.83688 20.4614 19.55907 19.73886 20.31719 + KO_Ctrl biological r cell part;cytoplasm;intracellular me 2 2 2 10.1 10.1 10.1 26.02 0.002459 2.0822 24.09015 3 2 2 1 1 0 0 0 0 3.772976545 0.013672727 -2.039439678 -8.274371027 Q9CWV0 Q9CWV0 Mitochondrial assembly of ribosomal large subun **Malsu1** sp|Q9CWV0|MASU1_MOUSE Mitochondrial assembly of ribosomal large subunit protein 1 OS=Mus musculus OX=10090 GN=Malsu1 PE=1 SV=1

23.62466431 24.02462196 24.34442 25.03322 19.82641 20.02488 21.20256 20.99221 + KO_Ctrl anatomical cell part;cyt Pathways i REACT_303 3 3 3 7.2 7.2 7.2 83.601 0 7.7247 26.95759 4 1 1 1 1 0 2 2 1 3.763005618 0.013428571 -3.745216846 -8.240362463 Q8CEC2;E9 Q8CEC2;E9 Aryl hydrocarbon receptor nuclear translocator **Arnt** tr|Q8CEC2|Q8CEC2_MOUSE Aryl hydrocarbon receptor nuclear translocator OS=Mus musculus OX=10090 GN=Arnt PE=1 SV=1;tr|E9QLT6|E9QLT6_MOUSE Aryl hydrocarbon receptor nuclear translocator OS=Mus musculus OX=10090 GN=Arnt PE=1 SV=2;tr|Q3ULM2|Q3ULM2_MOUSE Aryl hy

24.37451172 24.20067596 24.71009 24.33702 21.22102 21.11243 19.21026 20.48319 + KO_Ctrl cell part;cytoskeleton;cytosol;Golgi 5 5 5 12.3 12.3 12.3 83.193 0 11.471 27.07521 10 1 2 3 3 2 1 1 1 3.757383787 0.013192982 -3.898848057 -8.221240659 Q8C167;A0 Q8C167 Prolyl endopeptidase-like **Prepl** sp|Q8C167|PPCEL_MOUSE Prolyl endopeptidase-like OS=Mus musculus OX=10090 GN=Prepl PE=1 SV=1

19.95231056 20.38956451 22.13735 18.37037 26.74227 26.41796 26.69352 26.50685 + KO_Ctrl 3 3 3 9 9 9 70.046 0.001712 2.2388 29.06108 3 2 2 2 0 1 1 1 1 3.749665978 0.012965517 6.377750397 8.195051737 Q8BMC4 Q8BMC4 Nucleolar protein 9 **Nop9** sp|Q8BMC4|NOP9_MOUSE Nucleolar protein 9 OS=Mus musculus OX=10090 GN=Nop9 PE=1 SV=1

20.55824089 20.40461922 21.89583 19.92751 24.14172 24.77394 24.14888 24.31417 + KO_Ctrl biological r cell part;endoplasmic r REACT_338 4 3 3 5.4 4.8 4.8 133.53 0.009871 1.4209 27.07684 5 2 0 0 0 1 1 1 1 3.739160994 0.012745763 3.648127079 8.15952001 Q6DFW5;A Q6DFW5;A Phospholipid-transporting ATPase **Atp11b** tr|Q6DFW5|Q6DFW5_MOUSE Phospholipid-transporting ATPase OS=Mus musculus OX=10090 GN=Atp11b PE=1 SV=1;tr|A0A0G2JE89|A0A0G2JE89_MOUSE Phospholipid-transporting ATPase (Fragment) OS=Mus musculus OX=10090 GN=Atp11b PE=1 SV=1

19.7307663 19.33987617 20.67715 19.40971 22.14286 22.81282 22.7777 22.69053 + KO_Ctrl metabolic p cell part;intracellular membrane-bo 2 2 2 25.2 25.2 25.2 13.43 0 3.1548 25.28701 7 0 0 0 0 1 2 2 2 3.737648871 0.012533333 2.816601753 8.154416337 Q9CWB7 Q9CWB7 Glutaredoxin-like protein C5orf63 homolog sp|Q9CWB7|YD286_MOUSE Glutaredoxin-like protein C5orf63 homolog OS=Mus musculus OX=10090 PE=1 SV=1

26.97486877 26.9903183 27.01903 27.06517 22.11159 21.10642 19.71679 18.33058 + KO_Ctrl biological r cell body;c Fc gamma R REACT_344 2 2 2 3.1 3.1 3.1 151.55 0 13.975 28.8039 6 2 2 2 2 0 0 0 0 3.728263328 0.013377049 -6.696001053 -8.122799573 E9Q5G1;F8 E9Q5G1;F8 Unconventional myosin-X **Myo10** tr|E9Q5G1|E9Q5G1_MOUSE Unconventional myosin-X OS=Mus musculus OX=10090 GN=Myo10 PE=1 SV=1;sp|F8VQB6|MYO10_MOUSE Unconventional myosin-X OS=Mus musculus OX=10090 GN=Myo10 PE=1 SV=1

19.36965752 20.19112015 19.64166 21.65739 24.35016 24.39585 24.97439 24.31693 + KO_Ctrl cellular met cell part;ma mRNA surveillance path 3 3 3 14.4 14.4 14.4 27.419 0.00126 2.407 27.22867 6 1 1 1 0 2 3 1 2 3.70621307 0.01316129 4.294373035 8.048931535 B2LVG5;B2 B2LVG5;B2 Cleavage and polyadenylation specificity factor su **Cpsf4** tr|B2LVG5|B2LVG5_MOUSE Cleavage and polyadenylation specificity factor subunit 4 OS=Mus musculus OX=10090 GN=Cpsf4 PE=1 SV=1;tr|B2LVG6|B2LVG6_MOUSE Cleavage and polyadenylation specificity factor subunit 4 OS=Mus musculus OX=10090 GN=Cpsf4 PE=1 SV=1;tr|E0C

19.50460625 19.78818703 21.12996 20.43719 23.44594 23.53801 23.03465 23.11913 + KO_Ctrl 3 3 3 17.8 17.8 17.8 20.146 0 3.4172 25.82243 3 0 1 0 0 1 2 1 1 3.700469487 0.012952381 3.069447994 8.029784988 C0HK80;C0 C0HK80;C0HK79 sp|C0HK80|ARXS2_MOUSE Adipocyte-related X-chromosome expressed sequence 2 OS=Mus musculus OX=10090 GN=Arxes2 PE=1 SV=1;sp|C0HK79|ARXS1_MOUSE Adipocyte-related X-chromosome expressed sequence 1 OS=Mus musculus OX=10090 GN=Arxes1 PE=1 SV=1

20.08302307 21.72648621 19.79976 20.32181 24.1168 23.83519 24.16269 23.8292 + KO_Ctrl anatomical cell part;membrane;plasma membra 3 3 3 2.1 2.1 2.1 181.72 0.005071 1.7254 26.27341 3 0 0 0 0 2 2 3 2 3.69485467 0.01275 3.503199577 8.011105212 Q3LAC4 Q3LAC4 Phosphatidylinositol 3,4,5-trisphosphate-depend **Prex2** sp|Q3LAC4|PREX2_MOUSE Phosphatidylinositol 3,4,5-trisphosphate-dependent Rac exchanger 2 protein OS=Mus musculus OX=10090 GN=Prex2 PE=1 SV=2

19.29830551 20.54047585 21.29059 20.14291 23.56256 23.70546 23.44342 23.78367 + KO_Ctrl biological r cell part;cytoplasm;intracellular me 9 1 1 8.4 1.8 1.8 234.41 0 3.3916 26.09925 2 0 0 0 0 1 1 1 1 3.64235884 0.013353846 3.305704117 7.838237136 A0A1W2P8 A0A1W2P8 Ral GTPase-activating protein subunit alpha-1 **Ralgapa1** tr|A0A1W2P832|A0A1W2P832_MOUSE Ral GTPase-activating protein subunit alpha-1 OS=Mus musculus OX=10090 GN=Ralgapa1 PE=1 SV=1;sp|Q6GYP7|RGPA1_MOUSE Ral GTPase-activating protein subunit alpha-1 OS=Mus musculus OX=10090 GN=Ralgapa1 PE=1 SV=1

20.89674187 19.35231209 20.66953 20.14085 24.94695 24.45212 23.91752 23.25652 + KO_Ctrl biological r cell part;endoplasmic reticulum;intr 4 4 4 10.1 10.1 10.1 90.093 0.000165 3.0861 30.04817 7 1 1 2 0 2 2 3 3 3.621416176 0.013151515 3.878420353 7.770160079 Q8BZ36 Q8BZ36 RAD50-interacting protein 1 **Rint1** sp|Q8BZ36|RINT1_MOUSE RAD50-interacting protein 1 OS=Mus musculus OX=10090 GN=Rint1 PE=1 SV=2

22.93883514 22.47256279 22.603 22.54985 19.58689 20.88874 20.61611 20.14018 + KO_Ctrl biological r cell part;ext Cytokine-c REACT_336 1 1 1 1.9 1.9 1.9 56.578 0.007879 1.4836 24.64136 1 1 1 1 1 0 0 0 0 3.593720582 0.013910448 -2.333082199 -7.680897367 O35664 O35664 Interferon alpha/beta receptor 2 **Ifnar2** sp|O35664|INAR2_MOUSE Interferon alpha/beta receptor 2 OS=Mus musculus OX=10090 GN=Ifnar2 PE=1 SV=2

24.05821037 24.13743019 24.62906 23.62545 20.7578 19.4994 20.58697 18.53138 + KO_Ctrl biological r cell part;ma Glycosylph REACT_273 1 1 1 3.9 3.9 3.9 49.951 0 6.6963 26.24932 1 1 1 1 1 0 0 0 0 3.584033491 0.013705882 -4.268649101 -7.649880077 Q3TAA8;Q8 Q3TAA8;Q8 Phosphatidylinositol glycan anchor biosynthesis c **Pigu** tr|Q3TAA8|Q3TAA8_MOUSE Phosphatidylinositol glycan anchor biosynthesis class U protein OS=Mus musculus OX=10090 GN=Pigu PE=1 SV=1;sp|Q8K358|PIGU_MOUSE Phosphatidylinositol glycan anchor biosynthesis class U protein OS=Mus musculus OX=10090 GN=Pigu PE=1 SV=

21.29693794 21.60608673 19.24849 20.25642 24.66481 24.80055 24.57086 24.97018 + KO_Ctrl alcohol me cell part;intracellular m REACT_281 3 3 3 20.7 20.7 20.7 20.123 0 3.739 27.28542 8 0 0 1 0 2 2 3 3 3.583355348 0.013507246 4.149619102 7.647712657 P46656;A0 P46656 Adrenodoxin, mitochondrial  **Fdx1** sp|P46656|ADX_MOUSE Adrenodoxin, mitochondrial OS=Mus musculus OX=10090 GN=Fdx1 PE=1 SV=1

20.71434784 21.01074028 21.1228 20.38202 24.01206 23.63323 22.92607 22.78535 + KO_Ctrl anatomical cell part;cyt Huntington REACT_302 4 4 4 10 10 10 80.443 0 8.4375 26.07457 6 2 0 0 1 2 1 2 2 3.554614073 0.0152 2.531698227 7.556323864 G3X8Q0;O G3X8Q0;O Transcription factor Sp1 **Sp1** tr|G3X8Q0|G3X8Q0_MOUSE Transcription factor Sp1 OS=Mus musculus OX=10090 GN=Sp1 PE=1 SV=1;sp|O89090|SP1_MOUSE Transcription factor Sp1 OS=Mus musculus OX=10090 GN=Sp1 PE=1 SV=2

20.21366119 19.5349102 21.15477 20.51848 23.61908 22.81796 23.04388 23.58612 + KO_Ctrl biological r cell part;centrosome;intracellular n 10 10 10 6.2 6.2 6.2 243.72 0 27.321 26.12248 13 3 2 0 1 3 3 2 6 3.518479161 0.016676056 2.911305904 7.442720367 Q61043;A0 Q61043;A0 Ninein **Nin** sp|Q61043|NIN_MOUSE Ninein OS=Mus musculus OX=10090 GN=Nin PE=1 SV=4;tr|A0A1Y7VNC5|A0A1Y7VNC5_MOUSE Ninein OS=Mus musculus OX=10090 GN=Nin PE=1 SV=1

20.66013908 20.75572014 21.36814 21.0338 22.55253 23.65655 23.15103 22.87136 + KO_Ctrl cell part;nucleoplasm 3 3 3 5 5 5 96.062 0 7.738 26.69126 6 1 1 1 1 1 2 3 3 3.515571807 0.017333333 2.10341835 7.433642113 A0A2I3BQJ A0A2I3BQJ Serine/threonine-protein phosphatase 6 regulator **Ankrd28** tr|A0A2I3BQJ1|A0A2I3BQJ1_MOUSE Serine/threonine-protein phosphatase 6 regulatory ankyrin repeat subunit A OS=Mus musculus OX=10090 GN=Ankrd28 PE=1 SV=1;tr|A0A2I3BQ07|A0A2I3BQ07_MOUSE Serine/threonine-protein phosphatase 6 regulatory ankyrin repeat subunit

22.61865234 23.56743813 23.94601 22.92011 20.60164 20.75045 21.20814 20.5743 + KO_Ctrl biosynthet cell part;intracellular m REACT_292 2 2 2 5.2 5.2 5.2 49.939 0.001712 2.2392 25.56552 4 1 2 2 1 0 0 1 0 3.502089089 0.018027397 -2.479419708 -7.391662109 Q9D0G0 Q9D0G0 28S ribosomal protein S30, mitochondrial  **Mrps30** sp|Q9D0G0|RT30_MOUSE 28S ribosomal protein S30, mitochondrial OS=Mus musculus OX=10090 GN=Mrps30 PE=1 SV=1

20.54628563 19.48986816 21.71489 19.50413 24.46905 24.3694 24.02749 24.26184 + KO_Ctrl biological r cell cortex;cell part;cell REACT_297 5 5 5 3.5 3.5 3.5 229.72 0 4.4414 27.04781 7 0 0 1 0 3 4 3 3 3.501764907 0.017783784 3.968152046 7.390655155 Q811P8;S4 Q811P8 Rho GTPase-activating protein 32 **Arhgap32** sp|Q811P8|RHG32_MOUSE Rho GTPase-activating protein 32 OS=Mus musculus OX=10090 GN=Arhgap32 PE=1 SV=2

21.49198914 19.31735611 20.20984 20.10621 26.25618 24.49124 24.50094 24.43695 + KO_Ctrl biological a cell part;membrane;plasma membra 37 1 1 54.3 1.5 1.5 72.403 0.008307 1.4687 27.22656 2 0 0 0 0 1 1 1 1 3.482065858 0.018133333 4.639980793 7.329679792 B2RXQ9;D B2RXQ9 **Sorbs2** tr|B2RXQ9|B2RXQ9_MOUSE Sorbin and SH3 domain-containing protein 2 OS=Mus musculus OX=10090 GN=Sorbs2 PE=1 SV=1

24.01979446 23.93391037 25.0135 25.01052 18.25184 20.15163 19.9229 20.93952 + KO_Ctrl cell division cell part;intracellular membrane-bo 4 4 4 10 10 10 55.196 0 3.3518 27.1379 6 2 2 2 1 0 2 2 1 3.477142434 0.017894737 -4.677959442 -7.314505201 Q80YV2;H3 Q80YV2;H3 Nuclear-interacting partner of ALK **Zc3hc1** sp|Q80YV2|NIPA_MOUSE Nuclear-interacting partner of ALK OS=Mus musculus OX=10090 GN=Zc3hc1 PE=1 SV=1;tr|H3BKM2|H3BKM2_MOUSE Nuclear-interacting partner of ALK OS=Mus musculus OX=10090 GN=Zc3hc1 PE=1 SV=1;tr|D3Z3D0|D3Z3D0_MOUSE Nuclear-interacting partner o

23.94511986 24.26570702 24.99865 24.07692 19.16678 20.55674 19.72766 21.50282 + KO_Ctrl cell part;macromolecular complex;m 2 2 2 5.1 5.1 5.1 59.007 0 3.9001 26.47888 2 2 2 2 1 0 0 0 0 3.46666565 0.017662338 -4.083095074 -7.282300692 Q3TH73 Q3TH73 Protein tweety homolog 2 **Ttyh2** sp|Q3TH73|TTYH2_MOUSE Protein tweety homolog 2 OS=Mus musculus OX=10090 GN=Ttyh2 PE=1 SV=1

19.48200989 21.92944527 20.02202 20.61882 25.11668 25.04118 24.37862 24.20836 + KO_Ctrl 3 3 3 22.3 22.3 22.3 22.769 0 6.3708 27.20834 7 1 0 1 0 1 2 3 2 3.462123991 0.017435897 4.173138142 7.268376463 G5E8Q5;G G5E8Q5;G DCN1-like protein;DCN1-like protein 2 **Dcun1d2** tr|G5E8Q5|G5E8Q5_MOUSE DCN1-like protein 2 OS=Mus musculus OX=10090 GN=Dcun1d2 PE=1 SV=1;tr|G5E8Q6|G5E8Q6_MOUSE DCN1-like protein 2 OS=Mus musculus OX=10090 GN=Dcun1d2 PE=1 SV=1;tr|A0A0N4SW97|A0A0N4SW97_MOUSE DCN1-like protein 2 OS=Mus musculus OX=10090 GN

19.70107269 20.14513969 21.17771 21.70404 24.42515 24.1397 24.0937 23.85557 + KO_Ctrl cellular pro cell part;intracellular m REACT_292 2 2 2 22.1 22.1 22.1 16.284 0 3.717 27.52051 1 1 1 1 1 1 1 1 1 3.459326039 0.018025316 3.446538925 7.259809164 Q9DCC8 Q9DCC8 Mitochondrial import receptor subunit TOM20 ho **Tomm20** sp|Q9DCC8|TOM20_MOUSE Mitochondrial import receptor subunit TOM20 homolog OS=Mus musculus OX=10090 GN=Tomm20 PE=1 SV=1

27.537714 27.68852425 27.48915 27.53081 27.85733 27.89232 27.93528 27.90782 + KO_Ctrl biological r cell part;intracellular membrane-bo 13 13 13 20.3 20.3 20.3 127.77 0 64.564 31.27236 55 10 9 7 9 8 10 10 9 3.447999711 0.0194 0.336639881 7.22521285 A6PWC3;A A6PWC3;A Nardilysin **Nrd1** tr|A6PWC3|A6PWC3_MOUSE Nardilysin, N-arginine dibasic convertase, NRD convertase 1 OS=Mus musculus OX=10090 GN=Nrd1 PE=1 SV=1;tr|A2A9Q2|A2A9Q2_MOUSE Nardilysin, N-arginine dibasic convertase, NRD convertase 1 OS=Mus musculus OX=10090 GN=Nrd1 PE=1 SV=1;sp|Q

20.81474686 20.3051548 20.99749 21.28004 18.82056 19.14962 18.85319 18.16776 + KO_Ctrl cellular pro cell part;membrane 2 1 1 7.4 4.2 4.2 47.244 0.001264 2.4627 21.33336 4 0 0 0 0 1 1 1 1 3.443523641 0.019160494 -2.101574898 -7.211578066 Q3UED7 Q3UED7 **Gm4951** tr|Q3UED7|Q3UED7_MOUSE Interferon-gamma-inducible GTPase Ifgga2 protein OS=Mus musculus OX=10090 GN=Gm4951 PE=1 SV=1

26.74765587 25.91458511 25.42518 26.02897 21.26189 18.82785 22.04556 19.70416 + KO_Ctrl biological r cell part;cytoplasm;intracellular me 15 3 3 41.9 14.1 14.1 49.067 0 9.9849 28.04661 2 3 3 2 2 0 0 0 0 3.422257822 0.018926829 -5.569231987 -7.147086985 Q8VDW0;D Q8VDW0 ATP-dependent RNA helicase DDX39A **Ddx39a** sp|Q8VDW0|DX39A_MOUSE ATP-dependent RNA helicase DDX39A OS=Mus musculus OX=10090 GN=Ddx39a PE=1 SV=1

23.2008419 25.31409836 24.04034 24.04718 20.18293 19.5644 20.55808 21.06248 + KO_Ctrl anatomical cell part;cytoplasm;cytosol;intracell 3 3 3 1.8 1.8 1.8 182.35 0.009453 1.4359 26.3658 3 2 1 3 1 0 0 0 0 3.399366624 0.021012048 -3.808644295 -7.078194455 Q9ESK9;F7 Q9ESK9;F7 RB1-inducible coiled-coil protein 1 **Rb1cc1** sp|Q9ESK9|RBCC1_MOUSE RB1-inducible coiled-coil protein 1 OS=Mus musculus OX=10090 GN=Rb1cc1 PE=1 SV=3;tr|F7CC56|F7CC56_MOUSE RB1-inducible coiled-coil protein 1 (Fragment) OS=Mus musculus OX=10090 GN=Rb1cc1 PE=1 SV=1;tr|F7CCJ3|F7CCJ3_MOUSE RB1-inducible c

25.38906479 24.50772858 24.65009 24.92556 21.76154 18.75289 20.5045 19.53758 + KO_Ctrl biological r cell part;cytoplasm;intracellular me 3 3 3 17.5 17.5 17.5 22.517 0 5.8738 26.86207 10 3 2 2 3 1 0 0 0 3.367883556 0.02152381 -4.72898531 -6.984328064 Q9CY97 Q9CY97 RNA polymerase II subunit A C-terminal domain ph **Ssu72** sp|Q9CY97|SSU72_MOUSE RNA polymerase II subunit A C-terminal domain phosphatase SSU72 OS=Mus musculus OX=10090 GN=Ssu72 PE=1 SV=1

24.28757095 25.18918228 24.23674 24.32231 19.87368 22.07882 20.91246 20.22359 + KO_Ctrl biological r cell body;c Mineral abs REACT_313 1 1 1 14.6 14.6 14.6 14.486 0.002173 2.2061 26.5451 1 1 1 1 1 0 0 0 0 3.360766314 0.022023529 -3.736813068 -6.963248679 A8Y5P1;Q8 A8Y5P1;Q8 High affinity copper uptake protein 1 **Slc31a1** tr|A8Y5P1|A8Y5P1_MOUSE Copper transporter OS=Mus musculus OX=10090 GN=Slc31a1 PE=1 SV=1;sp|Q8K211|COPT1_MOUSE High affinity copper uptake protein 1 OS=Mus musculus OX=10090 GN=Slc31a1 PE=2 SV=1

21.23576355 20.14349747 20.6037 22.13903 24.43148 23.76213 24.35743 24.6525 + KO_Ctrl biological r cell part;cyt Basal trans REACT_282 1 1 1 18.1 18.1 18.1 14.09 0.000164 2.9868 26.66397 1 0 0 0 0 1 1 1 1 3.348806663 0.021767442 3.270386219 6.927943256 A0A087WS A0A087WS General transcription factor IIE subunit 2 **Gtf2e2** tr|A0A087WSE8|A0A087WSE8_MOUSE General transcription factor IIE subunit 2 (Fragment) OS=Mus musculus OX=10090 GN=Gtf2e2 PE=1 SV=1;sp|Q9D902|T2EB_MOUSE General transcription factor IIE subunit 2 OS=Mus musculus OX=10090 GN=Gtf2e2 PE=1 SV=2

20.03981209 19.76958084 21.13637 18.07202 23.88865 24.65992 24.96974 24.23125 + KO_Ctrl cellular comcell part;centrosome;en REACT_272 2 2 2 6.9 6.9 6.9 71.309 0 7.195 27.00303 4 0 0 0 0 2 2 2 2 3.345477419 0.021517241 4.682944775 6.918140951 Q8BKL6;Q8 Q8BKL6;Q8 Gamma-tubulin complex component 4 **Tubgcp4** tr|Q8BKL6|Q8BKL6_MOUSE Gamma-tubulin complex component OS=Mus musculus OX=10090 GN=Tubgcp4 PE=1 SV=1;tr|Q8BYN2|Q8BYN2_MOUSE Gamma-tubulin complex component OS=Mus musculus OX=10090 GN=Tubgcp4 PE=1 SV=1;sp|Q9D4F8|GCP4_MOUSE Gamma-tubulin complex component 4

23.26913643 24.05862427 24.20814 23.84479 19.91763 21.00533 21.78177 20.78546 + KO_Ctrl anatomical cell part;ma Long-term REACT_275 5 5 5 17.9 17.9 17.9 44.095 0 3.4366 26.36511 5 1 3 3 1 1 1 1 1 3.315482471 0.022 -2.972628593 -6.830329428 P27600;A0 P27600;A0 Guanine nucleotide-binding protein subunit alpha **Gna12** sp|P27600|GNA12_MOUSE Guanine nucleotide-binding protein subunit alpha-12 OS=Mus musculus OX=10090 GN=Gna12 PE=1 SV=3;tr|A0A0G2JG40|A0A0G2JG40_MOUSE Guanine nucleotide-binding protein subunit alpha-12 (Fragment) OS=Mus musculus OX=10090 GN=Gna12 PE=1 SV=1

21.11304474 20.77113342 19.8479 19.36487 23.46326 22.96229 22.93647 23.45362 + KO_Ctrl anatomical cell part;intracellular membrane-bo 1 1 1 12.3 12.3 12.3 9.1541 0.001715 2.2552 25.6248 3 0 0 0 0 1 1 1 1 3.309628476 0.022247191 2.929673195 6.813296543 A0A494BA A0A494BA Protein DPCD **Dpcd** tr|A0A494BAZ3|A0A494BAZ3_MOUSE Predicted gene 17018 OS=Mus musculus OX=10090 GN=Gm17018 PE=1 SV=1;sp|Q8BPA8|DPCD_MOUSE Protein DPCD OS=Mus musculus OX=10090 GN=Dpcd PE=1 SV=1

20.52184105 18.49047089 20.832 20.01613 23.48148 23.31202 23.71188 23.71513 + KO_Ctrl catabolic p cell part;cyt Arachidoni REACT_295 3 3 3 10.8 10.8 10.8 57.935 0 4.5647 26.06495 5 0 0 0 0 1 1 3 2 3.302037026 0.022 3.590016365 6.791259043 Q924D1;B Q924D1 **Cyp2j9** tr|Q924D1|Q924D1_MOUSE Cytochrome P450 CYP2J9 OS=Mus musculus OX=10090 GN=Cyp2j9 PE=1 SV=1

22.99132156 23.11021996 24.14981 22.83459 20.30303 18.6013 20.23234 20.20235 + KO_Ctrl cellular met cell part;cyt Ubiquitin mediated pro 3 3 3 27 27 27 21.11 0 9.5381 26.6679 6 2 2 1 2 0 1 1 0 3.291546712 0.022857143 -3.436730385 -6.760900237 Q9CY34;Q3 Q9CY34 NEDD8-conjugating enzyme UBE2F **Ube2f** sp|Q9CY34|UB2FA_MOUSE NEDD8-conjugating enzyme UBE2F OS=Mus musculus OX=10090 GN=Ube2f PE=1 SV=1

19.47533035 21.27954674 21.39393 18.92633 24.95136 24.43981 24.37159 24.59679 + KO_Ctrl establishm cell part;membrane 3 3 3 4.8 4.8 4.8 101.7 0.007194 1.5547 27.16816 3 0 0 1 1 1 1 1 2 3.287173241 0.022608696 4.321103096 6.748275559 G5E833;Q9 G5E833;Q9 Oxysterol-binding protein;Oxysterol-binding prote **Osbpl5** tr|G5E833|G5E833_MOUSE Oxysterol-binding protein OS=Mus musculus OX=10090 GN=Osbpl5 PE=1 SV=1;sp|Q9ER64|OSBL5_MOUSE Oxysterol-binding protein-related protein 5 OS=Mus musculus OX=10090 GN=Osbpl5 PE=1 SV=3

20.31515884 21.07089996 20.55417 21.28647 23.59651 22.83648 23.03444 22.48458 + KO_Ctrl biological r cell body;cell part;DNA-directed RN 3 3 3 14.5 14.5 14.5 41.236 0 9.0763 25.66725 5 0 0 0 0 1 3 2 3 3.286833836 0.022365591 2.181328773 6.747296602 Q6P6I6 Q6P6I6 DNA-directed RNA polymerase II subunit GRINL1A **Polr2m** sp|Q6P6I6|GRL1A_MOUSE DNA-directed RNA polymerase II subunit GRINL1A OS=Mus musculus OX=10090 GN=Polr2m PE=2 SV=2

22.93801117 23.1901474 23.04308 23.75032 21.58367 20.70386 20.61311 20.00548 + KO_Ctrl biological r cell part;cyt RNA degrad REACT_330 2 2 2 7.6 7.6 7.6 62.693 0 3.7421 25.10173 2 2 1 1 1 0 0 0 0 3.281195106 0.02212766 -2.50385952 -6.731049221 B9EIX0;Q3 B9EIX0;Q3 mRNA-decapping enzyme 1B **Dcp1b** tr|B9EIX0|B9EIX0_MOUSE DCP1 decapping enzyme homolog b (S. cerevisiae) OS=Mus musculus OX=10090 GN=Dcp1b PE=1 SV=1;sp|Q3U564|DCP1B_MOUSE mRNA-decapping enzyme 1B OS=Mus musculus OX=10090 GN=Dcp1b PE=1 SV=1

23.40327644 22.90045166 23.18306 23.61279 21.19422 19.46309 20.46977 19.29071 + KO_Ctrl 2 2 2 23 23 23 13.331 0.00231 2.1032 25.20262 2 2 2 1 1 0 0 0 0 3.276118605 0.022442105 -3.17044735 -6.716448541 Q9CZH3 Q9CZH3 Proteasome assembly chaperone 3 **Psmg3** sp|Q9CZH3|PSMG3_MOUSE Proteasome assembly chaperone 3 OS=Mus musculus OX=10090 GN=Psmg3 PE=1 SV=1

27.90959167 27.56957054 28.24921 24.66904 20.59453 20.7 20.18752 22.13869 + KO_Ctrl extracellula Alzheimer's disease;Cal 7 1 1 49.7 24.8 24.8 16.701 0 4.7922 29.78032 3 1 1 1 1 0 0 0 0 3.267557069 0.02275 -6.194168091 -6.691881582 Q9D6P8 Q9D6P8 Calmodulin-like protein 3 **Calml3** sp|Q9D6P8|CALL3_MOUSE Calmodulin-like protein 3 OS=Mus musculus OX=10090 GN=Calml3 PE=2 SV=1

22.4497509 23.88474083 23.99602 22.99381 20.64778 21.09369 20.48093 20.52178 + KO_Ctrl biological r cell part;cyt Jak-STAT signaling path 2 2 2 5.4 5.4 5.4 50.664 0 3.1769 25.61869 2 2 2 2 1 0 0 0 0 3.266869832 0.022515464 -2.645036221 -6.689912691 Q924S8 Q924S8 Sprouty-related, EVH1 domain-containing protein **Spred1** sp|Q924S8|SPRE1_MOUSE Sprouty-related, EVH1 domain-containing protein 1 OS=Mus musculus OX=10090 GN=Spred1 PE=1 SV=1

22.53476143 23.09148788 22.70683 22.67947 21.2057 19.56149 20.17021 19.73619 + KO_Ctrl developme cell part Wnt signaling pathway 3 3 3 13.2 13.2 13.2 54.365 0 11.94 25.09716 6 3 1 1 1 1 0 1 1 3.26066414 0.022816327 -2.584741116 -6.672154597 Q3UXU7;Q Q3UXU7;Q Vang-like protein 1 **Vangl1** tr|Q3UXU7|Q3UXU7_MOUSE Vang-like protein OS=Mus musculus OX=10090 GN=Vangl1 PE=1 SV=1;sp|Q80Z96|VANG1_MOUSE Vang-like protein 1 OS=Mus musculus OX=10090 GN=Vangl1 PE=1 SV=2;tr|A0A0H2UH25|A0A0H2UH25_MOUSE Vang-like protein 1 OS=Mus musculus OX=10090 GN=Vang

24.17394257 24.78551483 25.20292 24.7638 20.43305 21.60408 19.36021 21.72981 + KO_Ctrl biological r cell body;cell part;cell projection;cy 7 7 7 25 25 25 39.835 0 7.821 27.3955 8 2 3 5 1 1 1 2 1 3.247084857 0.023232323 -3.949756622 -6.633426884 Q8VCM5 Q8VCM5 Mitochondrial ubiquitin ligase activator of NFKB 1 **Mul1** sp|Q8VCM5|MUL1_MOUSE Mitochondrial ubiquitin ligase activator of NFKB 1 OS=Mus musculus OX=10090 GN=Mul1 PE=1 SV=2

23.78206825 23.11364174 23.48505 22.97706 21.59026 19.96582 20.13239 19.82557 + KO_Ctrl biosynthet cell part;ext Pyrimidine REACT_353 3 3 3 31.5 31.5 31.5 17.384 0.000639 2.6413 25.61661 4 2 1 1 1 0 0 1 0 3.240541295 0.023 -2.96094656 -6.614828551 Q9CQ43;Q Q9CQ43;Q8VCG1 **Dut** tr|Q9CQ43|Q9CQ43_MOUSE Deoxyuridine 5-triphosphate nucleotidohydrolase OS=Mus musculus OX=10090 GN=Dut PE=1 SV=1;tr|Q8VCG1|Q8VCG1_MOUSE Deoxyuridine 5-triphosphate nucleotidohydrolase OS=Mus musculus OX=10090 GN=Dut PE=1 SV=1

19.96138191 19.19254494 21.23183 21.26181 23.68752 24.02563 24.08448 23.55918 + KO_Ctrl cell part;intracellular m REACT_297 3 3 3 11.3 11.3 11.3 49.299 0 6.0241 26.55986 7 0 0 0 0 1 3 3 2 3.219309 0.023287129 3.427313328 6.554765014 Q5NCE8 Q5NCE8 Magnesium transporter MRS2 homolog, mitochon **Mrs2** sp|Q5NCE8|MRS2_MOUSE Magnesium transporter MRS2 homolog, mitochondrial OS=Mus musculus OX=10090 GN=Mrs2 PE=2 SV=2

20.12452507 20.36079025 21.15759 20.36275 24.13281 22.47813 23.12166 23.0418 + KO_Ctrl anatomical cell part;cell projection;cytoskeleto 4 3 3 4.4 3.3 3.3 108.29 0.00464 1.7572 25.67777 5 1 1 0 0 1 2 2 1 3.213843091 0.023058824 2.692185879 6.539372465 Q3TN34;E9 Q3TN34;E9 MICAL-like protein 2 **Micall2** sp|Q3TN34|MILK2_MOUSE MICAL-like protein 2 OS=Mus musculus OX=10090 GN=Micall2 PE=1 SV=1;tr|E9PZD2|E9PZD2_MOUSE MICAL-like protein 2 OS=Mus musculus OX=10090 GN=Micall2 PE=1 SV=1

24.48615837 24.70408821 24.16461 24.70915 18.50408 20.14365 19.94853 21.75834 + KO_Ctrl cell death;cellular process;death 2 2 2 16.2 16.2 16.2 26.353 0.000638 2.6261 26.45527 6 2 2 2 2 0 0 0 1 3.210048967 0.024737864 -4.427349567 -6.528704565 A0A0U1RN A0A0U1RN B-cell CLL/lymphoma 7 protein family member C **Bcl7c** tr|A0A0U1RNX8|A0A0U1RNX8_MOUSE B-cell CLL/lymphoma 7 protein family member C OS=Mus musculus OX=10090 GN=Bcl7c PE=1 SV=1;sp|O08664|BCL7C_MOUSE B-cell CLL/lymphoma 7 protein family member C OS=Mus musculus OX=10090 GN=Bcl7c PE=1 SV=1

22.02433205 20.73705864 19.07306 20.79528 25.65363 24.77188 24.57762 24.69376 + KO_Ctrl biological r apical part Calcium signaling path 4 4 4 20.1 20.1 20.1 38.139 0 5.298 27.33986 4 0 1 1 1 2 2 1 3 3.208449898 0.0245 4.266787052 6.524212579 D3YYR5;Q9 D3YYR5;Q9 P2X purinoceptor;P2X purinoceptor 4 **P2rx4** tr|D3YYR5|D3YYR5_MOUSE ATP receptor OS=Mus musculus OX=10090 GN=P2rx4 PE=1 SV=2;tr|Q9Z256|Q9Z256_MOUSE P2X purinoceptor OS=Mus musculus OX=10090 GN=P2rx4 PE=1 SV=1;tr|D3Z5U5|D3Z5U5_MOUSE P2X purinoceptor OS=Mus musculus OX=10090 GN=P2rx4 PE=1 SV=2;tr|Q9Z25

23.67325211 23.5952549 23.79054 23.68635 21.78112 19.96698 20.98163 19.76506 + KO_Ctrl biological r cell part;ext Protein processing in e 2 2 2 32.5 32.5 32.5 8.3635 0 7.5319 26.07087 2 1 1 1 1 1 1 0 0 3.20352154 0.024266667 -3.062650681 -6.510383481 Q3UZP4 Q3UZP4 Small VCP/p97-interacting protein **Svip** sp|Q3UZP4|SVIP_MOUSE Small VCP/p97-interacting protein OS=Mus musculus OX=10090 GN=Svip PE=3 SV=1

23.46026421 23.33224297 23.29151 23.46849 19.89363 20.5975 20.4897 21.85017 + KO_Ctrl cell differen cell part;macromolecul REACT_271 1 1 1 17.1 17.1 17.1 16.461 0.002604 2.0342 25.3502 3 1 1 1 1 0 0 0 0 3.193474905 0.024037736 -2.680379391 -6.482263625 G3UVU6;Q G3UVU6;Q Mediator of RNA polymerase II transcription subu **Gm20517;Med20** tr|G3UVU6|G3UVU6_MOUSE Mediator of RNA polymerase II transcription subunit 20 OS=Mus musculus OX=10090 GN=Gm20517 PE=3 SV=1;sp|Q9R0X0|MED20_MOUSE Mediator of RNA polymerase II transcription subunit 20 OS=Mus musculus OX=10090 GN=Med20 PE=1 SV=1

22.77227402 22.49818802 22.93359 23.17175 20.66028 20.08971 21.47304 20.17769 + KO_Ctrl 1 1 1 9.6 9.6 9.6 12.336 0.007192 1.5536 24.7761 2 1 1 1 1 0 0 0 0 3.187843316 0.023813084 -2.243768692 -6.466542877 A0A571BE A0A571BE82;A0A571BEH6;A0A571BGB9;A8WHU1;A0A571B **Ccdc30** tr|A0A571BE82|A0A571BE82_MOUSE Coiled-coil domain-containing protein 30 OS=Mus musculus OX=10090 GN=Ccdc30 PE=4 SV=1;tr|A0A571BEH6|A0A571BEH6_MOUSE Coiled-coil domain-containing protein 30 OS=Mus musculus OX=10090 GN=Ccdc30 PE=4 SV=1;tr|A0A571BGB9|A0A571BG

18.95648575 19.67010117 20.51289 20.87499 22.81372 22.76701 22.62605 22.9828 + KO_Ctrl anatomical cell part;intracellular non-membran 1 1 1 7.3 7.3 7.3 28.543 0 3.3278 25.2367 1 0 0 0 0 1 1 1 1 3.161557109 0.025962963 2.793778419 6.393557825 Q9D8M4 Q9D8M4 60S ribosomal protein L7-like 1 **Rpl7l1** sp|Q9D8M4|RL7L_MOUSE 60S ribosomal protein L7-like 1 OS=Mus musculus OX=10090 GN=Rpl7l1 PE=1 SV=1

23.54792595 24.84416962 24.64411 23.1495 20.4849 20.88021 19.94432 21.47647 + KO_Ctrl biological r cell part;cell projection;cytoplasm;c 19 1 1 52.2 3.9 3.9 63.138 0.001567 2.327 26.36471 2 1 1 1 1 0 0 0 0 3.159845855 0.025724771 -3.349951267 -6.388828805 F8SLP9;F8S F8SLP9;F8S PEX5-related protein **Pex5l** tr|F8SLP9|F8SLP9_MOUSE PEX5-related protein OS=Mus musculus OX=10090 GN=Pex5l PE=1 SV=1;tr|F8SLQ3|F8SLQ3_MOUSE PEX5-related protein OS=Mus musculus OX=10090 GN=Pex5l PE=1 SV=1;tr|F8SLQ1|F8SLQ1_MOUSE PEX5-related protein OS=Mus musculus OX=10090 GN=Pex5l PE

22.65920639 23.14443398 22.60173 23.0851 20.9749 19.83923 18.69889 19.49942 + KO_Ctrl biological r cell part;membrane;plasma membra 1 1 1 6.5 6.5 6.5 33.221 0 6.0428 24.78676 1 1 1 1 1 0 0 0 0 3.138675873 0.026836364 -3.119507313 -6.330550399 Q6PGA2;O Q6PGA2;O GTP-binding protein RAD **Rrad** tr|Q6PGA2|Q6PGA2_MOUSE GTP-binding protein OS=Mus musculus OX=10090 GN=Rrad PE=1 SV=1;sp|O88667|RAD_MOUSE GTP-binding protein RAD OS=Mus musculus OX=10090 GN=Rrad PE=1 SV=1

22.66711235 21.91746902 21.98256 22.32696 20.45063 20.99509 20.93156 21.0055 + KO_Ctrl anatomical cell part;cytoplasm 2 2 2 6 6 6 40.32 0.004509 1.8044 24.5131 2 1 1 1 1 0 1 1 1 3.134501852 0.026594595 -1.377830029 -6.319108644 Q3UCV8;A Q3UCV8;A Ubiquitin thioesterase otulin **Otulin** sp|Q3UCV8|OTUL_MOUSE Ubiquitin thioesterase otulin OS=Mus musculus OX=10090 GN=Otulin PE=1 SV=1;tr|A0A2I3BRS5|A0A2I3BRS5_MOUSE Ubiquitin thioesterase otulin (Fragment) OS=Mus musculus OX=10090 GN=Otulin PE=1 SV=1

19.5263958 20.49160576 20.456 20.82121 25.57868 25.55066 23.51256 23.39532 + KO_Ctrl alcohol me extracellular space;insu REACT_331 4 4 4 16.6 16.6 16.6 30.372 0 15.998 27.62038 6 0 0 0 0 3 4 1 1 3.107096501 0.027178571 4.185503483 6.244381783 Q07079 Q07079 Insulin-like growth factor-binding protein 5 **Igfbp5** sp|Q07079|IBP5_MOUSE Insulin-like growth factor-binding protein 5 OS=Mus musculus OX=10090 GN=Igfbp5 PE=1 SV=1

21.25717545 20.6448288 20.7667 19.27925 28.37447 30.106 26.01604 25.60334 + KO_Ctrl cell part;extracellular re REACT_307 19 19 19 48 48 48 53.326 0 84.483 31.42852 41 0 0 0 0 9 18 8 5 3.087374576 0.028707965 7.037973404 6.191027731 P47867;A0 P47867 Secretogranin-3 **Scg3** sp|P47867|SCG3_MOUSE Secretogranin-3 OS=Mus musculus OX=10090 GN=Scg3 PE=1 SV=1

23.78436661 23.67895508 23.67411 23.24016 21.91159 19.91368 21.04928 20.40303 + KO_Ctrl 2 2 2 7.8 7.8 7.8 47.416 0 3.1517 25.65968 3 2 2 1 1 0 0 0 0 3.085679421 0.02845614 -2.775003433 -6.186458171 Q922R1 Q922R1 UPF0183 protein C16orf70 homolog sp|Q922R1|CP070_MOUSE UPF0183 protein C16orf70 homolog OS=Mus musculus OX=10090 PE=1 SV=2

24.30910873 23.50748634 24.0694 23.4148 19.93372 20.51172 21.37051 21.82294 + KO_Ctrl anatomical cell part;intracellular membrane-bo 4 4 4 22.5 22.5 22.5 22.03 0 4.3536 25.79835 6 3 2 1 2 0 0 1 0 3.062117611 0.029669565 -2.915478706 -6.123210032 Q08024 Q08024 Core-binding factor subunit beta **Cbfb** sp|Q08024|PEBB_MOUSE Core-binding factor subunit beta OS=Mus musculus OX=10090 GN=Cbfb PE=1 SV=1

19.7954216 19.92663383 20.84907 22.38997 26.14137 27.98212 25.25947 25.04552 + KO_Ctrl biological r cell part;cyt Tight junction 4 3 3 21.9 18.3 18.3 24.757 0 13.66 29.41818 5 0 0 0 0 1 3 1 1 3.009835413 0.033896552 5.366847038 5.984620985 Q9CZT8;A2 Q9CZT8;A2 Ras-related protein Rab-3B **Rab3b** sp|Q9CZT8|RAB3B_MOUSE Ras-related protein Rab-3B OS=Mus musculus OX=10090 GN=Rab3b PE=1 SV=1;tr|A2A7Z6|A2A7Z6_MOUSE Ras-related protein Rab-3B OS=Mus musculus OX=10090 GN=Rab3b PE=1 SV=1

20.15105057 21.20955849 21.16768 19.63963 23.82813 23.51075 22.59585 23.40419 + KO_Ctrl biological r cell part;cytoplasm;intr REACT_275 5 3 3 6.4 5.4 5.4 105.38 0 3.3714 25.52109 3 0 0 1 0 1 1 2 1 3.002787337 0.034700855 2.792748928 5.96612071 Q99K46 Q99K46 Ubiquitin carboxyl-terminal hydrolase 11 **Usp11** sp|Q99K46|UBP11_MOUSE Ubiquitin carboxyl-terminal hydrolase 11 OS=Mus musculus OX=10090 GN=Usp11 PE=1 SV=4

20.75382614 21.94248962 19.48253 20.42555 23.92379 23.36078 24.16385 23.90003 + KO_Ctrl 3 3 3 8.8 8.8 8.8 46.338 0.00157 2.347 26.48373 3 1 1 0 1 2 2 2 2 2.996925122 0.03440678 3.186012268 5.950765895 Q80UY1;A0 Q80UY1 UPF0586 protein C9orf41 homolog sp|Q80UY1|CARME_MOUSE Carnosine N-methyltransferase OS=Mus musculus OX=10090 GN=Carnmt1 PE=1 SV=1

26.10197449 25.94911194 26.05354 25.95607 26.81054 26.54261 26.39751 26.5401 + KO_Ctrl cell part;cytoplasm;intracellular me 14 14 14 23.8 23.8 23.8 81.163 0 27.216 29.59527 37 9 6 4 6 8 9 9 9 2.98873462 0.036840336 0.557516098 5.929362245 Q3TVI8;D3 Q3TVI8 Pre-B-cell leukemia transcription factor-interacting **Pbxip1** sp|Q3TVI8|PBIP1_MOUSE Pre-B-cell leukemia transcription factor-interacting protein 1 OS=Mus musculus OX=10090 GN=Pbxip1 PE=1 SV=2

22.49567795 22.95397186 23.92958 22.2199 20.31834 20.81177 20.62706 19.99957 + KO_Ctrl cellular comcell part;endoplasmic reticulum;intr 1 1 1 6.6 6.6 6.6 21.102 0.000163 2.9377 25.20568 1 1 1 1 1 0 0 0 0 2.986323781 0.0369 -2.460598946 -5.923073161 Q5XKN4 Q5XKN4 Protein jagunal homolog 1 **Jagn1** sp|Q5XKN4|JAGN1_MOUSE Protein jagunal homolog 1 OS=Mus musculus OX=10090 GN=Jagn1 PE=1 SV=2

22.07675743 22.82452202 22.44316 23.41015 19.97921 19.93821 20.59233 21.05106 + KO_Ctrl anatomical cell part;cell projection;intracellular 1 1 1 1.7 1.7 1.7 73.373 0.000635 2.5217 24.62355 3 1 1 1 1 0 0 0 0 2.975373422 0.037652893 -2.298445702 -5.894569982 P0C7T6 P0C7T6 Ataxin-1-like **Atxn1l** sp|P0C7T6|ATX1L_MOUSE Ataxin-1-like OS=Mus musculus OX=10090 GN=Atxn1l PE=1 SV=1

20.35529709 19.93436813 21.69164 20.67227 22.71915 22.90879 23.17185 22.945 + KO_Ctrl biological r cell part;cytoplasm;macromolecular 3 3 3 6.2 6.2 6.2 83.305 0.000642 2.7008 25.53851 5 0 0 0 0 1 3 3 1 2.973328338 0.037344262 2.27280426 5.889258102 Q6PHQ8;E Q6PHQ8;E N-alpha-acetyltransferase 35, NatC auxiliary subun **Naa35** sp|Q6PHQ8|NAA35_MOUSE N-alpha-acetyltransferase 35, NatC auxiliary subunit OS=Mus musculus OX=10090 GN=Naa35 PE=1 SV=1;tr|E9Q2U4|E9Q2U4_MOUSE N-alpha-acetyltransferase 35, NatC auxiliary subunit (Fragment) OS=Mus musculus OX=10090 GN=Naa35 PE=1 SV=1;tr|E9Q

20.65440941 19.29964256 21.21075 21.33265 24.04993 23.45186 23.95819 23.10933 + KO_Ctrl biological r cell part;cytoplasm;intr REACT_359 3 3 3 5.1 5.1 5.1 76.956 0 3.9669 25.92286 4 0 0 0 0 3 1 2 3 2.960355155 0.037560976 3.017965794 5.855644637 Q8BWR8 Q8BWR8 Rhophilin-2 **Rhpn2** sp|Q8BWR8|RHPN2_MOUSE Rhophilin-2 OS=Mus musculus OX=10090 GN=Rhpn2 PE=1 SV=2

19.48609352 19.91101837 21.51694 20.23512 22.56412 23.7167 23.43275 24.04527 + KO_Ctrl cell motility cell part;endosome;intracellular me 3 3 3 5.5 5.5 5.5 72.185 0 7.3998 26.30095 7 0 0 0 0 2 3 3 1 2.950473836 0.037258065 3.152414322 5.830137951 Q80U56 Q80U56 Late secretory pathway protein AVL9 homolog **Avl9** sp|Q80U56|AVL9_MOUSE Late secretory pathway protein AVL9 homolog OS=Mus musculus OX=10090 GN=Avl9 PE=1 SV=2

21.98427773 20.05706787 21.22872 21.16691 23.32704 23.45739 23.40847 23.82085 + KO_Ctrl biosynthet cell part;cytoplasm;intracellular me 4 4 4 10 10 10 74.575 0 5.184 26.89076 5 1 1 1 0 2 2 2 1 2.945074416 0.03696 2.394190788 5.816235241 H9KV04;Q H9KV04;Q Mini-chromosome maintenance complex-binding **Mcmbp** tr|H9KV04|H9KV04_MOUSE Mini-chromosome maintenance complex-binding protein OS=Mus musculus OX=10090 GN=Mcmbp PE=1 SV=1;sp|Q8R3C0|MCMBP_MOUSE Mini-chromosome maintenance complex-binding protein OS=Mus musculus OX=10090 GN=Mcmbp PE=1 SV=1

20.84100533 21.3706913 21.63391 19.96215 22.89139 23.36653 23.2014 23.16759 + KO_Ctrl biosynthet cell part;intracellular m REACT_292 1 1 1 4.3 4.3 4.3 23.366 0.002607 2.0537 25.71618 1 0 0 0 0 1 1 1 1 2.929086272 0.03815873 2.204789162 5.775211788 Q9D1N9 Q9D1N9 39S ribosomal protein L21, mitochondrial  **Mrpl21** sp|Q9D1N9|RM21_MOUSE 39S ribosomal protein L21, mitochondrial OS=Mus musculus OX=10090 GN=Mrpl21 PE=1 SV=1

23.25666618 23.22130013 23.54227 23.27355 23.84699 23.97745 23.72983 24.06521 biological r cell part;cytoskeleton;intracellular n 2 2 2 10.1 10.1 10.1 22.345 0 3.4963 26.82736 8 2 2 2 1 2 1 1 2 2.852293979 0.05215748 0.581423283 5.581120159 A0A1B0GR A0A1B0GR ELMO domain-containing protein 2 **Elmod2** tr|A0A1B0GRZ8|A0A1B0GRZ8_MOUSE ELMO domain-containing protein 2 (Fragment) OS=Mus musculus OX=10090 GN=Elmod2 PE=1 SV=1;sp|Q8BGF6|ELMD2_MOUSE ELMO domain-containing protein 2 OS=Mus musculus OX=10090 GN=Elmod2 PE=1 SV=1

20.44190216 19.9446106 21.52575 20.44479 22.72167 22.52464 23.88642 23.28362 amine meta cell part;cytosol 1 1 1 47.1 47.1 47.1 5.9886 0.000163 2.8884 25.70841 5 0 0 0 0 1 1 1 1 2.840885732 0.052625 2.51482439 5.552695304 H3BKG8;H H3BKG8;H3BLA5 **Ahcyl2** tr|H3BKG8|H3BKG8_MOUSE Putative adenosylhomocysteinase 3 (Fragment) OS=Mus musculus OX=10090 GN=Ahcyl2 PE=1 SV=1;tr|H3BLA5|H3BLA5_MOUSE Putative adenosylhomocysteinase 3 (Fragment) OS=Mus musculus OX=10090 GN=Ahcyl2 PE=1 SV=1

21.91194534 20.23079681 19.36429 20.16104 23.26785 23.51424 23.3199 23.49179 establishment of localization;protein transport 2 2 2 1.3 1.3 1.3 269.25 0 4.2643 25.93924 4 0 0 0 0 1 2 2 2 2.831567599 0.054108527 2.981428623 5.529555538 Q8BL99;H7 Q8BL99;H7 Protein dopey-1 **Dopey1** sp|Q8BL99|DOP1_MOUSE Protein dopey-1 OS=Mus musculus OX=10090 GN=Dop1a PE=1 SV=2;tr|H7BWZ9|H7BWZ9_MOUSE Protein dopey-1 OS=Mus musculus OX=10090 GN=Dop1a PE=1 SV=1;tr|A0A087WQ48|A0A087WQ48_MOUSE Protein dopey-1 OS=Mus musculus OX=10090 GN=Dop1a PE=1 SV=1

24.26728058 23.63333893 24.91789 23.64042 21.01472 22.24778 21.3211 20.14887 developme cell part;cyt Cardiac muscle contrac 46 2 0 82.9 6.4 0 32.495 0 5.6822 27.36645 4 1 2 2 1 1 1 1 1 2.83069502 0.053692308 -2.931612492 -5.527392206 E9Q452;Q8 E9Q452;Q8BSH3;E9Q454 **Tpm1** tr|E9Q452|E9Q452_MOUSE Tropomyosin alpha-1 chain OS=Mus musculus OX=10090 GN=Tpm1 PE=1 SV=1;tr|Q8BSH3|Q8BSH3_MOUSE Tropomyosin alpha-1 chain OS=Mus musculus OX=10090 GN=Tpm1 PE=1 SV=1;tr|E9Q454|E9Q454_MOUSE Tropomyosin alpha-1 chain OS=Mus musculus OX=1009

21.823843 22.73167038 22.51965 22.63314 20.20796 21.17346 20.89573 20.10908 localization cell part;cytoplasm;intracellular me 1 1 1 1.4 1.4 1.4 118 0.000641 2.6774 24.42284 2 1 1 1 1 0 0 0 0 2.828524865 0.053770992 -1.830520153 -5.522014505 Q3TAA7 Q3TAA7 Serine/threonine-protein kinase 11-interacting pro **Stk11ip** sp|Q3TAA7|S11IP_MOUSE Serine/threonine-protein kinase 11-interacting protein OS=Mus musculus OX=10090 GN=Stk11ip PE=1 SV=1

26.6486969 26.70714569 26.77769 26.94503 27.17274 27.15634 27.13917 27.08608 catabolic p cell part;cyt Purine metabolism 10 10 10 31.9 31.9 31.9 50.239 0 33.111 30.36797 43 7 7 4 4 6 10 9 8 2.827903113 0.053363636 0.368943691 5.520474479 Q8BIW1 Q8BIW1 Protein prune homolog **Prune** sp|Q8BIW1|PRUN1_MOUSE Exopolyphosphatase PRUNE1 OS=Mus musculus OX=10090 GN=Prune1 PE=1 SV=1

22.32676888 22.56022644 22.51944 22.94312 20.17047 21.61696 20.65792 20.66895 carbohydra cell part;membrane REACT_327 1 1 1 9 9 9 18.725 0.003198 1.9574 24.52987 2 1 1 1 1 0 0 0 0 2.818623648 0.053774436 -1.808812141 -5.497526535 A0A0G2JD A0A0G2JD UDP-N-acetylglucosamine transporter **Slc35a3** tr|A0A0G2JDH8|A0A0G2JDH8_MOUSE UDP-N-acetylglucosamine transporter (Fragment) OS=Mus musculus OX=10090 GN=Slc35a3 PE=1 SV=1;tr|D3YXZ7|D3YXZ7_MOUSE UDP-N-acetylglucosamine transporter (Fragment) OS=Mus musculus OX=10090 GN=Slc35a3 PE=1 SV=1;sp|Q8R1T4|S35A3_

20.4918499 19.66106796 19.96988 19.60486 23.87718 22.35216 22.02982 22.06526 biological r cell part;cyt Axon guidance 4 4 4 4.7 4.7 4.7 106.22 0 9.6748 26.09909 7 1 1 1 1 1 3 3 3 2.814267187 0.053373134 2.649188042 5.486776632 Q9DC04;F6 Q9DC04;F6 Regulator of G-protein signaling 3 **Rgs3** sp|Q9DC04|RGS3_MOUSE Regulator of G-protein signaling 3 OS=Mus musculus OX=10090 GN=Rgs3 PE=1 SV=2;tr|F6X4A2|F6X4A2_MOUSE Regulator of G-protein-signaling 3 (Fragment) OS=Mus musculus OX=10090 GN=Rgs3 PE=1 SV=1;tr|Q542M0|Q542M0_MOUSE Regulator of G-protein

21.07425499 18.86930847 19.00991 22.65107 25.84859 25.91707 24.91237 25.38106 biological r cell part;ext Chemokine REACT_272 4 4 4 66.7 66.7 66.7 8.4047 0 12.468 28.33858 5 1 1 1 1 2 3 2 2 2.811284938 0.053451852 5.113639355 5.479426357 P50153 P50153 Guanine nucleotide-binding protein G(I)/G(S)/G(O **Gng4** sp|P50153|GBG4_MOUSE Guanine nucleotide-binding protein G(I)/G(S)/G(O) subunit gamma-4 OS=Mus musculus OX=10090 GN=Gng4 PE=1 SV=1

21.22636032 18.65542221 20.7474 19.99605 23.89245 24.1332 23.10449 23.03263 biological r cell part;intracellular membrane-bo 3 3 3 29.2 29.2 29.2 18.128 0 10 26.22944 4 2 1 2 1 1 1 1 1 2.783034051 0.057352941 3.384383678 5.410143851 A0A1B0GT A0A1B0GT Inhibitor of growth protein 1 **Ing1** tr|A0A1B0GT89|A0A1B0GT89_MOUSE Inhibitor of growth protein 1 (Fragment) OS=Mus musculus OX=10090 GN=Ing1 PE=1 SV=1;sp|Q9QXV3|ING1_MOUSE Inhibitor of growth protein 1 OS=Mus musculus OX=10090 GN=Ing1 PE=2 SV=1

24.38251495 24.6216507 24.22964 25.81683 20.67764 21.9119 22.03672 19.44147 biological r cell part;cel Alzheimer's REACT_282 4 4 4 4.9 4.9 4.9 160.05 0 5.4661 26.80021 4 2 3 2 3 1 0 0 0 2.736836539 0.062773723 -3.745727062 -5.298183666 S4R255;F8 S4R255;F8 Nitric oxide synthase;Nitric oxide synthase, brain **Nos1** tr|S4R255|S4R255_MOUSE Nitric oxide synthase OS=Mus musculus OX=10090 GN=Nos1 PE=1 SV=1;tr|F8WGF2|F8WGF2_MOUSE Constitutive NOS OS=Mus musculus OX=10090 GN=Nos1 PE=1 SV=1;sp|Q9Z0J4|NOS1_MOUSE Nitric oxide synthase, brain OS=Mus musculus OX=10090 GN=Nos1 PE

25.30372047 26.16691208 25.74496 26.41232 24.53688 24.28546 24.67584 23.93382 anatomical cell part;endosome;Golgi apparatus 3 3 3 5.5 5.5 5.5 92.403 0.006788 1.6034 28.37397 3 2 3 3 1 1 1 2 1 2.730824654 0.063536232 -1.548979759 -5.28373399 A6H6A9 A6H6A9 Rab GTPase-activating protein 1-like **Rabgap1l** sp|A6H6A9|RBG1L_MOUSE Rab GTPase-activating protein 1-like OS=Mus musculus OX=10090 GN=Rabgap1l PE=1 SV=1

22.96243477 23.03042603 23.38574 23.12807 21.95412 20.44781 21.34937 20.27094 biological r cell body;c Retinol met REACT_280 3 3 3 17.3 17.3 17.3 34.826 0 3.2119 25.28577 3 1 1 1 1 0 0 1 2 2.709323987 0.065323741 -2.121110439 -5.232280497 O55240;D O55240;D 11-cis retinol dehydrogenase **Rdh5** sp|O55240|RDH5_MOUSE Retinol dehydrogenase 5 OS=Mus musculus OX=10090 GN=Rdh5 PE=1 SV=1;tr|D3Z0N7|D3Z0N7_MOUSE Retinol dehydrogenase 5 (Fragment) OS=Mus musculus OX=10090 GN=Rdh5 PE=1 SV=1

21.26861191 20.73513412 19.64873 20.91442 22.3769 23.24457 22.8358 23.87145 anatomical cell part;cytosol;intracellular memb 2 2 2 6 6 6 48.343 0 3.8767 25.70972 6 0 1 0 0 1 2 2 1 2.684866409 0.068885714 2.440455914 5.174172288 P97346 P97346 Nucleoredoxin **Nxn** sp|P97346|NXN_MOUSE Nucleoredoxin OS=Mus musculus OX=10090 GN=Nxn PE=1 SV=1

19.94651604 21.305233 18.53086 19.99499 22.79846 22.69051 23.11777 23.04203 biological r cell part;nucleoplasm 1 1 1 11.5 11.5 11.5 19.312 0.000485 2.7776 25.39614 2 0 0 0 0 1 1 1 1 2.677744036 0.069446809 2.96779108 5.157333995 Q921P9 Q921P9 Transcription elongation factor A protein-like 1 **Tceal1** sp|Q921P9|TCAL1_MOUSE Transcription elongation factor A protein-like 1 OS=Mus musculus OX=10090 GN=Tceal1 PE=2 SV=1

20.53536797 20.15522003 21.88434 21.04237 22.98633 23.47643 23.68228 24.93544 brush border;cell corte REACT_297 28 1 1 44.5 2 2 78.776 0.001567 2.3227 26.4356 2 0 0 0 0 1 1 1 1 2.666677821 0.072140845 2.865793705 5.131246176 Q9QYB5 Q9QYB5 Gamma-adducin **Add3** sp|Q9QYB5|ADDG_MOUSE Gamma-adducin OS=Mus musculus OX=10090 GN=Add3 PE=1 SV=2

20.10696793 22.57741165 21.94415 19.64944 25.03779 24.50936 25.17607 24.48155 2 2 2 10.1 10.1 10.1 21.137 0.005495 1.6853 27.30904 2 0 0 0 0 1 2 1 1 2.663421479 0.072391608 3.73169899 5.123586727 Q9CPR1;H Q9CPR1;H RWD domain-containing protein 4 **Rwdd4;Rwdd4a** sp|Q9CPR1|RWDD4_MOUSE RWD domain-containing protein 4 OS=Mus musculus OX=10090 GN=Rwdd4 PE=2 SV=1;tr|H3BJI4|H3BJI4_MOUSE RWD domain-containing 4A OS=Mus musculus OX=10090 GN=Rwdd4a PE=1 SV=1;tr|H3BJN8|H3BJN8_MOUSE RWD domain-containing 4A (Fragment) OS=Mus

22.06177521 20.1671505 21.30695 20.50147 23.99265 23.70028 23.36813 22.89628 anatomical apical part Basal cell c REACT_274 1 1 1 2 2 2 78.86 0.001561 2.2717 25.90458 1 0 0 0 0 1 1 1 1 2.657185634 0.07225 2.480000496 5.108940684 Q60838 Q60838 Segment polarity protein dishevelled homolog DVL **Dvl2** sp|Q60838|DVL2_MOUSE Segment polarity protein dishevelled homolog DVL-2 OS=Mus musculus OX=10090 GN=Dvl2 PE=1 SV=2

19.27231407 20.7406559 19.55768 21.08192 22.25069 22.41748 22.70946 22.35893 biological r cell part;cytoplasm;intracellular me 2 2 2 5.5 5.5 5.5 48.795 0.005067 1.7205 24.9725 2 0 0 1 0 1 1 1 1 2.620229456 0.078896552 2.270998955 5.022722396 Q99LG4 Q99LG4 Tetratricopeptide repeat protein 5 **Ttc5** sp|Q99LG4|TTC5_MOUSE Tetratricopeptide repeat protein 5 OS=Mus musculus OX=10090 GN=Ttc5 PE=1 SV=2

28.56873703 28.67320442 28.57395 28.76787 28.94843 28.92076 29.05559 28.87569 biological r cell part;cyt Tight juncti REACT_293 31 31 31 47.2 47.2 47.2 99.226 0 134.34 32.08046 174 23 22 19 20 25 26 26 27 2.613617166 0.079123288 0.304179668 5.007399829 A0A067XG A0A067XG Peripheral plasma membrane protein CASK **Cask** tr|A0A067XG53|A0A067XG53_MOUSE Peripheral plasma membrane protein CASK (Fragment) OS=Mus musculus OX=10090 GN=Cask PE=1 SV=1;sp|O70589|CSKP_MOUSE Peripheral plasma membrane protein CASK OS=Mus musculus OX=10090 GN=Cask PE=1 SV=2

19.31262589 21.21330833 21.09294 21.64139 23.16875 23.67777 23.39008 23.55215 anatomical cell part;cyt Chagas dis REACT_281 2 2 2 4.8 4.8 4.8 117.51 0.004643 1.7696 26.20963 2 0 1 0 1 1 1 1 1 2.611901736 0.078911565 2.632124424 5.003429795 F6QCP8;P0 F6QCP8;P0 Angiotensin-converting enzyme;Angiotensin-conv **Ace** tr|F6QCP8|F6QCP8_MOUSE Angiotensin-converting enzyme (Fragment) OS=Mus musculus OX=10090 GN=Ace PE=1 SV=1;sp|P09470|ACE_MOUSE Angiotensin-converting enzyme OS=Mus musculus OX=10090 GN=Ace PE=1 SV=3

20.13819695 18.95127678 19.34222 21.87197 23.53077 23.09162 23.18829 23.88176 anatomical cell part Retinol met REACT_280 2 2 2 9.9 9.9 9.9 33.652 0 3.9221 25.80944 2 0 0 0 0 1 1 2 2 2.602008512 0.080540541 3.34719038 4.980574762 O88876;G O88876;G Short-chain dehydrogenase/reductase 3 **Dhrs3** sp|O88876|DHRS3_MOUSE Short-chain dehydrogenase/reductase 3 OS=Mus musculus OX=10090 GN=Dhrs3 PE=1 SV=2;tr|G5E8W9|G5E8W9_MOUSE Short-chain dehydrogenase/reductase 3 OS=Mus musculus OX=10090 GN=Dhrs3 PE=1 SV=1;tr|B1ARS9|B1ARS9_MOUSE Short-chain dehydrogenas

27.67283249 27.77643204 27.77436 27.85252 28.00737 28.24509 28.0177 28.14686 establishm cell part;cytoplasm;cytosol;endoso 15 15 10 49.4 49.4 36.4 54.325 0 63.853 31.17039 69 11 11 11 11 11 10 10 10 2.592648451 0.082040268 0.335219383 4.959015429 O88746;Q O88746;Q Target of Myb protein 1 **Tom1** sp|O88746|TOM1_MOUSE Target of Myb protein 1 OS=Mus musculus OX=10090 GN=Tom1 PE=1 SV=1;tr|Q3UDC3|Q3UDC3_MOUSE Target of Myb protein 1 OS=Mus musculus OX=10090 GN=Tom1 PE=1 SV=1

23.48406792 23.6198616 23.3668 23.10594 21.92352 21.1014 19.42138 20.35488 behavior;bi cell part;cytoplasm 1 1 1 15.3 15.3 15.3 14.7 0 5.0106 25.4406 1 1 1 1 1 0 0 0 0 2.588459579 0.08184 -2.693872929 -4.949387138 Q9D6V8 Q9D6V8 Polyadenylate-binding protein-interacting protein **Paip2** sp|Q9D6V8|PAIP2_MOUSE Polyadenylate-binding protein-interacting protein 2 OS=Mus musculus OX=10090 GN=Paip2 PE=1 SV=1

23.112957 23.74026299 22.75079 23.01985 19.90671 20.82913 21.68542 19.41062 biological a cell part;ext Cell adhesion molecule 2 2 2 12.8 12.8 12.8 23.054 0 3.4349 25.1828 3 2 1 1 1 0 0 0 0 2.586844604 0.081695364 -2.697994232 -4.945678358 O54942 O54942 Claudin-5 **Cldn5** sp|O54942|CLD5_MOUSE Claudin-5 OS=Mus musculus OX=10090 GN=Cldn5 PE=1 SV=2

26.64237595 26.91889 26.69458 26.62617 27.03622 27.44016 27.26204 27.25052 biological r cell part;cyt Nucleotide REACT_290 10 10 9 27.2 27.2 27.2 43.512 0 52.043 30.32002 40 5 6 5 7 8 8 8 8 2.579806633 0.084157895 0.526728153 4.929537143 P54728 P54728 UV excision repair protein RAD23 homolog B **Rad23b** sp|P54728|RD23B_MOUSE UV excision repair protein RAD23 homolog B OS=Mus musculus OX=10090 GN=Rad23b PE=1 SV=2

22.65700531 22.56367302 22.58284 22.91351 20.31013 21.57689 19.28383 20.05654 anatomical cell part;centrosome;intracellular n 1 1 1 4.3 4.3 4.3 39.316 0.005934 1.6737 25.66161 1 1 1 1 1 0 1 1 1 2.571757774 0.085986928 -2.372410774 -4.911120127 A0A1W2P6 A0A1W2P6 POC1 centriolar protein homolog B **Poc1b** tr|A0A1W2P6E5|A0A1W2P6E5_MOUSE POC1 centriolar protein homolog B OS=Mus musculus OX=10090 GN=Poc1b PE=1 SV=1;tr|Q9D3W6|Q9D3W6_MOUSE POC1 centriolar protein homolog B OS=Mus musculus OX=10090 GN=Poc1b PE=1 SV=1;tr|A6H699|A6H699_MOUSE POC1 centriolar protein

20.86061287 19.90746117 19.63859 20.90513 21.98381 21.94135 22.04517 21.78113 cell part;cytoplasm 3 2 2 5.2 3.7 3.7 70.612 0.004505 1.7997 24.45168 3 0 0 0 0 1 2 2 1 2.557217513 0.087974026 1.609915733 4.877964465 B9EI38;A2AB9EI38;A2ARalBP1-associated Eps domain-containing protein **Reps2** tr|B9EI38|B9EI38_MOUSE RalBP1-associated Eps domain-containing protein 2 OS=Mus musculus OX=10090 GN=Reps2 PE=1 SV=1;tr|A2AFI8|A2AFI8_MOUSE RalBP1-associated Eps domain-containing protein 2 OS=Mus musculus OX=10090 GN=Reps2 PE=1 SV=1;sp|Q80XA6|REPS2_MOUSE

21.06653976 20.10677338 22.05288 20.78642 25.06919 23.27611 23.78826 23.26342 cell part;nucleoplasm 2 2 2 6.5 6.5 6.5 59.241 0.007195 1.5559 26.04233 2 0 0 0 0 2 2 1 2 2.548078165 0.091716129 2.846091747 4.857199455 Q80ZU5 Q80ZU5 Coiled-coil domain-containing protein 181 **Ccdc181** sp|Q80ZU5|CC181_MOUSE Coiled-coil domain-containing protein 181 OS=Mus musculus OX=10090 GN=Ccdc181 PE=1 SV=1

24.01077652 24.1483345 24.21897 24.03699 24.59861 24.33552 24.45432 24.43879 establishm cell part;cytoplasm;cytoskeleton;en 6 6 6 7.2 7.2 7.2 147.62 0 24.847 27.83533 20 3 2 3 1 2 5 6 4 2.546098137 0.091128205 0.353040695 4.852708352 E9PYJ7;Q6 E9PYJ7;Q6 Membrane-associated phosphatidylinositol transf **Pitpnm2** tr|E9PYJ7|E9PYJ7_MOUSE Membrane-associated phosphatidylinositol transfer protein 2 OS=Mus musculus OX=10090 GN=Pitpnm2 PE=1 SV=1;sp|Q6ZPQ6|PITM2_MOUSE Membrane-associated phosphatidylinositol transfer protein 2 OS=Mus musculus OX=10090 GN=Pitpnm2 PE=1 SV=2

23.58554649 23.51231766 23.51316 23.40367 20.34479 21.56345 20.61306 22.40286 cell part;extracellular organelle;mem 2 2 2 12.4 12.4 12.4 35.384 0 4.222 26.64155 2 1 1 1 1 0 1 1 1 2.531596076 0.094751592 -2.272631168 -4.819896995 Q9D136 Q9D136 2-oxoglutarate and iron-dependent oxygenase do **Ogfod3** sp|Q9D136|OGFD3_MOUSE 2-oxoglutarate and iron-dependent oxygenase domain-containing protein 3 OS=Mus musculus OX=10090 GN=Ogfod3 PE=2 SV=1

23.02162552 23.66351128 24.52259 22.96397 21.6083 20.67167 21.90498 20.66647 cell part;ma Tight junction;Viral myo 4 3 3 1.9 1.5 1.5 221.84 0.003644 1.9226 25.93526 3 2 2 1 1 1 0 0 0 2.53021377 0.095240506 -2.330069065 -4.816777017 E9Q264 E9Q264 **Myh15** tr|E9Q264|E9Q264_MOUSE Myosin, heavy chain 15 OS=Mus musculus OX=10090 GN=Myh15 PE=1 SV=1

27.0489521 27.31155396 27.4827 27.56449 27.77944 28.03826 28.15248 28.1053 cell motility cell part;cyt Gap junctio REACT_278 32 2 2 80.4 3.6 3.6 49.953 0 6.9667 31.14625 14 2 2 2 2 2 2 2 2 2.490867049 0.102415094 0.666944504 4.72851238 Q9CWF2 Q9CWF2 Tubulin beta-2B chain **Tubb2b** sp|Q9CWF2|TBB2B_MOUSE Tubulin beta-2B chain OS=Mus musculus OX=10090 GN=Tubb2b PE=1 SV=1

19.40408325 20.50213242 20.18166 21.44801 23.53576 23.01856 22.78642 22.05732 amine meta cell part;en Glycosamin REACT_324 2 2 2 4.7 4.7 4.7 82.063 0.000637 2.6029 25.34601 5 1 0 1 0 1 1 1 1 2.485762287 0.1029 2.465542793 4.717137533 P70428;F6 P70428;F6 Exostosin-2 **Ext2** sp|P70428|EXT2_MOUSE Exostosin-2 OS=Mus musculus OX=10090 GN=Ext2 PE=1 SV=2;tr|F6WHC9|F6WHC9_MOUSE Exostosin-2 (Fragment) OS=Mus musculus OX=10090 GN=Ext2 PE=1 SV=1;tr|A2AIG3|A2AIG3_MOUSE Exostosin-2 (Fragment) OS=Mus musculus OX=10090 GN=Ext2 PE=1 SV=1;tr

24.66329575 24.4187336 27.15422 23.55391 21.64286 20.33587 19.67593 21.29771 biological r cell part;intracellular 7 3 3 41.9 26.7 26.7 22.215 0 7.5424 27.94425 4 2 2 3 1 0 0 0 0 2.481576726 0.103826087 -4.20944643 -4.707823904 Q8BGZ1;F2 Q8BGZ1;F2 Hippocalcin-like protein 4 **Hpcal4** sp|Q8BGZ1|HPCL4_MOUSE Hippocalcin-like protein 4 OS=Mus musculus OX=10090 GN=Hpcal4 PE=1 SV=3;tr|F2Z3Z1|F2Z3Z1_MOUSE Hippocalcin-like protein 4 (Fragment) OS=Mus musculus OX=10090 GN=Hpcal4 PE=1 SV=1

23.60896492 23.52647591 24.15892 22.34649 20.53648 19.81886 21.64228 19.03615 biological r cell part;intracellular m REACT_271 4 4 4 12.7 12.7 12.7 77.864 0 6.2985 27.16385 4 3 3 3 1 1 1 1 1 2.472516015 0.104617284 -3.151773453 -4.687702123 A0A140LH A0A140LH Mediator of RNA polymerase II transcription subu **Med25** tr|A0A140LHQ0|A0A140LHQ0_MOUSE Mediator complex subunit 25 OS=Mus musculus OX=10090 GN=Med25 PE=1 SV=1;tr|A0A140LHG7|A0A140LHG7_MOUSE Mediator complex subunit 25 OS=Mus musculus OX=10090 GN=Med25 PE=1 SV=1;sp|Q8VCB2|MED25_MOUSE Mediator of RNA polymerase I

22.16306305 22.07561493 21.72925 22.85732 21.04882 20.29428 21.13142 20.67163 anatomical cell part;cell surface;ext REACT_315 4 3 3 7.5 6.4 6.4 98.19 0.004242 1.8872 25.31479 4 1 1 1 1 0 1 2 1 2.460266649 0.107705521 -1.419774055 -4.660585514 Q00993;Q Q00993;Q Tyrosine-protein kinase receptor UFO;Receptor pr **Axl** sp|Q00993|UFO_MOUSE Tyrosine-protein kinase receptor UFO OS=Mus musculus OX=10090 GN=Axl PE=1 SV=2;tr|Q6PE80|Q6PE80_MOUSE Receptor protein-tyrosine kinase OS=Mus musculus OX=10090 GN=Axl PE=1 SV=1

20.36487389 20.80998039 21.64421 21.62541 22.90421 22.60779 22.44187 22.62638 biosynthet cell part;intracellular m REACT_292 2 2 2 18.9 18.9 18.9 15.944 0 7.7583 25.03616 2 0 1 0 0 1 1 1 1 2.455013064 0.107341463 1.533944607 4.648985877 Q99N92 Q99N92 39S ribosomal protein L27, mitochondrial  **Mrpl27** sp|Q99N92|RM27_MOUSE 39S ribosomal protein L27, mitochondrial OS=Mus musculus OX=10090 GN=Mrpl27 PE=1 SV=1

22.75429916 22.73937607 23.49607 22.99193 19.971 20.83818 21.82765 19.57914 biological r cell part;cytoplasm;Golgi apparatus 3 3 3 5.4 5.4 5.4 103.68 0 5.1447 24.99929 4 1 1 1 2 0 0 1 0 2.436699488 0.113260606 -2.441424847 -4.608691784 Q8CIE4;A0 Q8CIE4 **Parp10** sp|Q8CIE4|PAR10_MOUSE Protein mono-ADP-ribosyltransferase PARP10 OS=Mus musculus OX=10090 GN=Parp10 PE=2 SV=1

22.89897728 23.55109978 22.98134 22.91386 19.63466 19.51184 22.02599 19.64104 anatomical cell part;cytoplasm;cytoskeleton;int 2 2 2 3.9 3.9 3.9 77.009 0.000485 2.7683 25.12605 3 2 2 1 2 0 0 0 0 2.428674351 0.115204819 -2.882936954 -4.591103431 A0A5F8MP A0A5F8MP Tectonic-2 **Tctn2** tr|A0A5F8MPG0|A0A5F8MPG0_MOUSE Tectonic-2 OS=Mus musculus OX=10090 GN=Tctn2 PE=1 SV=1;sp|Q2MV57|TECT2_MOUSE Tectonic-2 OS=Mus musculus OX=10090 GN=Tctn2 PE=1 SV=2

22.37416649 22.24754143 22.6781 21.95943 18.4706 19.50467 20.3765 20.88145 biological r cell part;ch Colorectal c REACT_331 2 2 2 3.3 3.3 3.3 123.28 0.000637 2.6 24.4332 4 1 2 2 1 0 0 0 0 2.415357641 0.117628743 -2.506503105 -4.562009589 E9QPY6;A0 E9QPY6;A0 DNA mismatch repair protein Msh3 **Msh3** tr|E9QPY6|E9QPY6_MOUSE DNA mismatch repair protein OS=Mus musculus OX=10090 GN=Msh3 PE=1 SV=2;tr|A0A087WQ16|A0A087WQ16_MOUSE DNA mismatch repair protein OS=Mus musculus OX=10090 GN=Msh3 PE=1 SV=1;tr|A0A087WP43|A0A087WP43_MOUSE DNA mismatch repair protein M

22.5261879 22.90354347 23.46151 22.19755 19.96915 21.24515 21.20283 21.17715 biological a cell part;cytoplasm;cytoplasmic me 2 2 2 8.4 8.4 8.4 37.829 0 3.3407 24.86717 4 1 1 1 2 0 0 0 0 2.408591696 0.119452381 -1.873628616 -4.547271283 Q9ER39 Q9ER39 Torsin-1A **Tor1a** sp|Q9ER39|TOR1A_MOUSE Torsin-1A OS=Mus musculus OX=10090 GN=Tor1a PE=1 SV=1

26.8436203 26.92206955 26.92468 27.0591 27.61996 27.21697 27.28445 27.43825 autophagy; cell part;cyt Osteoclast REACT_295 10 10 10 44.1 44.1 44.1 48.162 0 76.907 30.39039 62 9 9 7 7 8 6 9 8 2.396004454 0.122035503 0.452541828 4.519930224 Q64337;D Q64337;D Sequestosome-1 **Sqstm1** sp|Q64337|SQSTM_MOUSE Sequestosome-1 OS=Mus musculus OX=10090 GN=Sqstm1 PE=1 SV=1;tr|D3YZJ1|D3YZJ1_MOUSE Sequestosome-1 OS=Mus musculus OX=10090 GN=Sqstm1 PE=1 SV=1

24.04067802 22.81644058 23.56999 23.19188 21.89561 21.90341 20.70979 20.25833 cell part Lysine degr REACT_285 1 1 1 4.9 4.9 4.9 55.051 0.008739 1.4609 25.51848 1 1 1 1 1 0 0 0 0 2.377339059 0.125082353 -2.212958813 -4.479571669 D6RCH2;Q D6RCH2;Q Procollagen galactosyltransferase 2 **Colgalt2** tr|D6RCH2|D6RCH2_MOUSE Procollagen galactosyltransferase 2 OS=Mus musculus OX=10090 GN=Colgalt2 PE=1 SV=1;sp|Q6NVG7|GT252_MOUSE Procollagen galactosyltransferase 2 OS=Mus musculus OX=10090 GN=Colgalt2 PE=2 SV=2

24.02267647 24.27696419 24.4393 25.11231 22.27274 22.36798 20.9743 19.50352 biological r cell part;cytoplasm;intracellular me 2 2 2 6.8 6.8 6.8 61.159 0 13.941 25.92198 2 2 1 1 2 0 0 0 0 2.375658544 0.124350877 -3.183177948 -4.475948816 D3YTU2;Q3 D3YTU2;Q3 Prolyl 3-hydroxylase OGFOD1 **Ogfod1** tr|D3YTU2|D3YTU2_MOUSE 2-oxoglutarate and iron-dependent oxygenase domain-containing protein 1 OS=Mus musculus OX=10090 GN=Ogfod1 PE=1 SV=1;sp|Q3U0K8|OGFD1_MOUSE Prolyl 3-hydroxylase OGFOD1 OS=Mus musculus OX=10090 GN=Ogfod1 PE=1 SV=1

22.44354248 22.32621956 22.74544 23.43288 21.26747 20.08023 20.7283 18.95408 cell cycle;cellular process 1 1 1 14.9 14.9 14.9 9.6308 0.007059 1.574 24.72993 1 1 1 1 1 0 0 0 0 2.367601521 0.125976744 -2.479502678 -4.458604081 F2Z4B3;O3 F2Z4B3;O3 Cyclin-dependent kinase 2-associated protein 1 **Cdk2ap1** tr|F2Z4B3|F2Z4B3_MOUSE Cyclin-dependent kinase 2-associated protein 1 OS=Mus musculus OX=10090 GN=Cdk2ap1 PE=1 SV=1;sp|O35207|CDKA1_MOUSE Cyclin-dependent kinase 2-associated protein 1 OS=Mus musculus OX=10090 GN=Cdk2ap1 PE=1 SV=2

20.2335434 21.51531219 19.89028 20.74542 22.38582 22.52981 23.2627 22.01662 catabolic process;cellular metabolic process;cel 7 7 7 5.4 5.4 5.4 176.66 0 7.6467 26.0897 8 1 2 2 0 2 5 3 1 2.360794892 0.127375723 1.952600956 4.443982743 Q8BL06 Q8BL06 Inactive ubiquitin carboxyl-terminal hydrolase 54 **Usp54** sp|Q8BL06|UBP54_MOUSE Inactive ubiquitin carboxyl-terminal hydrolase 54 OS=Mus musculus OX=10090 GN=Usp54 PE=1 SV=2

20.26055527 19.44755363 21.09068 18.85697 21.69655 23.08698 23.16659 24.15954 biological a cell part;cel Adherens j REACT_297 6 2 2 33.3 12 12 21.441 0.006501 1.6208 26.32944 2 0 1 1 0 1 1 1 1 2.356374546 0.126643678 3.113476753 4.434502835 Q05144;A0 Q05144;A0 Ras-related C3 botulinum toxin substrate 2 **Rac2** sp|Q05144|RAC2_MOUSE Ras-related C3 botulinum toxin substrate 2 OS=Mus musculus OX=10090 GN=Rac2 PE=1 SV=1;tr|A0A2R8VHH0|A0A2R8VHH0_MOUSE Ras-related C3 botulinum toxin substrate 2 (Fragment) OS=Mus musculus OX=10090 GN=Rac2 PE=1 SV=1

26.44925308 26.36833 25.87319 26.42594 27.27622 26.96444 27.26428 26.79823 biological r cell part;cytoplasm;extr REACT_274 6 6 6 83.2 83.2 83.2 11.871 0 120.44 29.94391 37 5 4 4 5 3 5 5 4 2.353366919 0.126148571 0.79661417 4.428059593 Q9QUH0;A Q9QUH0;A Glutaredoxin-1 **Glrx** sp|Q9QUH0|GLRX1_MOUSE Glutaredoxin-1 OS=Mus musculus OX=10090 GN=Glrx PE=1 SV=3;tr|A0A1Y7VM65|A0A1Y7VM65_MOUSE Glutaredoxin-1 OS=Mus musculus OX=10090 GN=Glrx PE=1 SV=1

21.43634224 21.3997345 21.65158 22.45287 20.12275 19.74987 20.82582 20.36003 biological r cell part;intracellular membrane-bo 2 2 2 4.6 4.6 4.6 98.1 0 6.2838 25.20896 4 1 1 1 1 1 0 2 1 2.345874243 0.128545455 -1.470515251 -4.412032381 A0A1B0GR A0A1B0GR MHC class II regulatory factor RFX1 **Rfx1** tr|A0A1B0GRV3|A0A1B0GRV3_MOUSE MHC class II regulatory factor RFX1 OS=Mus musculus OX=10090 GN=Rfx1 PE=1 SV=1;sp|P48377|RFX1_MOUSE MHC class II regulatory factor RFX1 OS=Mus musculus OX=10090 GN=Rfx1 PE=1 SV=2

29.92238235 29.99158859 29.9592 29.99873 30.20758 30.15424 30.14528 30.04349 biological r cell part;cytoplasm;intracellular me 22 22 3 59.4 59.4 9.7 51.596 0 201.97 33.31959 194 21 21 21 21 19 22 22 22 2.345732832 0.127819209 0.169671535 4.411730229 P61202 P61202 COP9 signalosome complex subunit 2 **Cops2** sp|P61202|CSN2_MOUSE COP9 signalosome complex subunit 2 OS=Mus musculus OX=10090 GN=Cops2 PE=1 SV=1

28.07851982 28.30007553 28.23959 28.10944 28.45678 28.38286 28.4311 28.4182 biosynthet cell part;cyt Fatty acid biosynthesis 11 11 11 47.7 47.7 47.7 48.627 0 79.511 31.56606 77 9 8 9 8 8 10 10 11 2.332503913 0.132359551 0.240328789 4.383518529 Q9D404 Q9D404 3-oxoacyl-[acyl-carrier-protein] synthase, mitocho **Oxsm** sp|Q9D404|OXSM_MOUSE 3-oxoacyl-[acyl-carrier-protein] synthase, mitochondrial OS=Mus musculus OX=10090 GN=Oxsm PE=1 SV=1

21.23706436 20.5929985 23.30433 20.97197 26.24538 27.97569 23.8447 26.3652 2 2 1 52.5 52.5 41 6.912 0 9.4266 29.69029 3 0 0 0 0 1 2 1 1 2.331499363 0.131977654 4.581148148 4.381380636 E9Q161 E9Q161 **Alg13** tr|E9Q161|E9Q161_MOUSE N-acetylglucosaminyldiphosphodolichol N-acetylglucosaminyltransferase OS=Mus musculus OX=10090 GN=Alg13 PE=1 SV=2

19.64272308 21.04343796 21.1756 19.20917 22.14096 22.32292 22.95883 22.82067 anatomical cell part;cyt Acute myel REACT_272 2 2 2 5.5 5.5 5.5 60.539 0.006921 1.5835 25.27569 3 1 0 1 0 1 1 2 1 2.298068372 0.144466667 2.293115139 4.310582409 S4R1S1;Q8 S4R1S1;Q8 Phosphatidylinositol 4,5-bisphosphate 3-kinase c **Pik3cb** tr|S4R1S1|S4R1S1_MOUSE Phosphatidylinositol-4,5-bisphosphate 3-kinase OS=Mus musculus OX=10090 GN=Pik3cb PE=1 SV=1;sp|Q8BTI9|PK3CB_MOUSE Phosphatidylinositol 4,5-bisphosphate 3-kinase catalytic subunit beta isoform OS=Mus musculus OX=10090 GN=Pik3cb PE=1 S

25.17827988 25.32981682 25.4519 25.44919 25.70203 25.81885 26.03802 26.26617 biological r cell part;cytoplasm;intracellular me 2 2 2 21.2 21.2 21.2 20.29 0 12.63 28.98777 10 2 2 2 2 2 2 2 2 2.294779107 0.143955801 0.603971958 4.303653039 A0A494BB A0A494BB Steroid receptor RNA activator 1 **Sra1** tr|A0A494BBA2|A0A494BBA2_MOUSE Steroid receptor RNA activator 1 (Fragment) OS=Mus musculus OX=10090 GN=Sra1 PE=1 SV=1;tr|G3X8R2|G3X8R2_MOUSE Steroid receptor RNA activator 1 (Fragment) OS=Mus musculus OX=10090 GN=Sra1 PE=1 SV=1;sp|Q80VJ2|SRA1_MOUSE Steroid

32.05727386 32.0479126 32.25901 32.16036 32.34763 32.34776 32.32507 32.3714 anatomical cell part;ext Benzoate d REACT_286 28 28 28 79.7 79.7 79.7 44.816 0 323.31 35.50462 401 24 24 23 24 27 27 27 26 2.288031009 0.144835165 0.216823578 4.289457335 Q8QZT1 Q8QZT1 Acetyl-CoA acetyltransferase, mitochondrial **Acat1** sp|Q8QZT1|THIL_MOUSE Acetyl-CoA acetyltransferase, mitochondrial OS=Mus musculus OX=10090 GN=Acat1 PE=1 SV=1

20.38409615 20.77994919 20.30179 20.41691 18.95506 19.16435 20.0454 19.05309 anatomical cell part;intracellular membrane-bo 2 2 2 2.2 2.2 2.2 109.98 0.000164 2.9924 21.93762 2 0 0 0 0 1 1 2 2 2.286382794 0.145289617 -1.166211128 -4.285994182 V9GXA5;G V9GXA5;G Zinc finger transcription factor Trps1 **Trps1** tr|V9GXA5|V9GXA5_MOUSE Zinc finger transcription factor Trps1 OS=Mus musculus OX=10090 GN=Trps1 PE=1 SV=1;tr|G3UW90|G3UW90_MOUSE Zinc finger transcription factor Trps1 OS=Mus musculus OX=10090 GN=Trps1 PE=1 SV=1;tr|V9GX74|V9GX74_MOUSE Zinc finger transcrip

22.83859444 23.38047028 23.07589 23.25854 18.80583 21.24904 21.57472 20.01299 anatomical cell part;cell projection; REACT_278 6 6 6 5.3 5.3 5.3 197.55 0 10.919 26.28757 8 1 2 2 2 1 2 2 3 2.266089196 0.151804348 -2.727729321 -4.243486334 Q6VH22;A Q6VH22 Intraflagellar transport protein 172 homolog **Ift172** sp|Q6VH22|IF172_MOUSE Intraflagellar transport protein 172 homolog OS=Mus musculus OX=10090 GN=Ift172 PE=1 SV=1

23.50069427 22.80905724 25.01958 24.05895 21.59397 19.27324 21.3806 21.1789 alcohol me cell part;cytoplasm;cyto REACT_316 3 3 2 2.6 2.6 1.7 159.92 0 3.2154 26.26331 6 2 1 2 2 1 1 2 0 2.251377275 0.155827027 -2.990395546 -4.212821801 A2ARP1;E9 A2ARP1 Inositol hexakisphosphate and diphosphoinositol **Ppip5k1** sp|A2ARP1|VIP1_MOUSE Inositol hexakisphosphate and diphosphoinositol-pentakisphosphate kinase 1 OS=Mus musculus OX=10090 GN=Ppip5k1 PE=1 SV=1

32.01962662 32.02058792 32.08819 32.20763 32.28273 32.27772 32.25721 32.26791 anatomical cell part;cel Regulation REACT_307 13 13 13 95 95 95 14.957 0 134.05 35.46322 206 13 13 13 13 12 12 12 12 2.246736675 0.155698925 0.187382698 4.203175488 P62962;Q5 P62962;Q5 Profilin-1;Profilin **Pfn1** sp|P62962|PROF1_MOUSE Profilin-1 OS=Mus musculus OX=10090 GN=Pfn1 PE=1 SV=2;tr|Q5SX49|Q5SX49_MOUSE Profilin OS=Mus musculus OX=10090 GN=Pfn1 PE=1 SV=1

19.52672958 18.87616539 20.66379 19.98592 22.03379 21.03651 21.47105 21.78684 biosynthet cell part;ext Alzheimer's REACT_287 1 1 1 16.4 16.4 16.4 25.095 0 4.5946 24.14554 4 0 0 1 0 1 1 1 1 2.241278845 0.156898396 1.818896294 4.191846438 P00848 P00848 ATP synthase subunit a **Mtatp6** sp|P00848|ATP6_MOUSE ATP synthase subunit a OS=Mus musculus OX=10090 GN=Mtatp6 PE=1 SV=1

28.284935 28.22425842 28.25246 27.99189 28.76424 28.73483 28.57888 28.41768 catabolic p cell part;cyt Nicotinate REACT_277 7 7 7 59 59 59 23.076 0 142.2 31.61824 54 6 5 5 6 5 5 5 5 2.238606709 0.157212766 0.435521603 4.186306071 Q9JM14;A Q9JM14;A 5(3)-deoxyribonucleotidase, cytosolic type **Nt5c** sp|Q9JM14|NT5C_MOUSE 5(3)-deoxyribonucleotidase, cytosolic type OS=Mus musculus OX=10090 GN=Nt5c PE=1 SV=1;tr|A2A9X5|A2A9X5_MOUSE 5(3)-deoxyribonucleotidase, cytosolic type OS=Mus musculus OX=10090 GN=Nt5c PE=1 SV=1

23.66589928 23.19160843 23.60082 23.36292 19.87945 21.10474 22.1881 19.05948 biological r cell part;intracellular membrane-bo 2 2 2 21.7 21.7 21.7 16.837 0 3.6314 26.58992 4 1 1 1 1 1 1 1 1 2.2285472 0.159174603 -2.897371292 -4.165485831 A0A2R8W7 A0A2R8W7 Zinc finger protein 740 **Zfp740;Znf740** tr|A0A2R8W747|A0A2R8W747_MOUSE Zinc finger protein 740 OS=Mus musculus OX=10090 GN=Zfp740 PE=1 SV=1;tr|D3Z4A3|D3Z4A3_MOUSE Zinc finger protein 740 (Fragment) OS=Mus musculus OX=10090 GN=Zfp740 PE=1 SV=9;sp|Q6NZQ6|ZN740_MOUSE Zinc finger protein 740 OS=Mus

24.36339188 24.26470566 24.53035 24.44164 24.57126 24.91242 24.92823 24.92379 biological r cell part;Golgi apparatus;intracellul 2 2 2 11.8 11.8 11.8 34.79 0 9.1577 28.05165 11 1 1 1 1 1 2 2 2 2.226351959 0.158610526 0.43390274 4.160950073 P52875;D3 P52875;D3 Transmembrane protein 165 **Tmem165** sp|P52875|TM165_MOUSE Transmembrane protein 165 OS=Mus musculus OX=10090 GN=Tmem165 PE=1 SV=2;tr|D3YV67|D3YV67_MOUSE GDT1 family protein (Fragment) OS=Mus musculus OX=10090 GN=Tmem165 PE=1 SV=1

33.69322205 33.68676758 33.65662 33.68708 33.73459 33.77225 33.7597 33.83479 biological r cell part;cyt Protein pro REACT_329 59 59 59 75.3 75.3 75.3 89.321 0 323.31 36.96935 762 55 56 57 53 50 54 55 55 2.212526687 0.160774869 0.09441185 4.132448034 Q01853 Q01853 Transitional endoplasmic reticulum ATPase **Vcp** sp|Q01853|TERA_MOUSE Transitional endoplasmic reticulum ATPase OS=Mus musculus OX=10090 GN=Vcp PE=1 SV=4

21.38825226 20.85860634 20.66386 18.68436 23.66958 22.56188 22.92928 22.7978 anatomical cell part;Golgi apparatu REACT_328 4 4 4 7.4 7.4 7.4 81.997 0.002605 2.045 25.28302 4 0 0 0 0 3 2 1 2 2.177345795 0.171666667 2.590865612 4.060408018 Q9D5R3 Q9D5R3 Centrosomal protein of 83 kDa **Cep83** sp|Q9D5R3|CEP83_MOUSE Centrosomal protein of 83 kDa OS=Mus musculus OX=10090 GN=Cep83 PE=1 SV=2

28.81558609 29.15978622 28.63996 29.10652 29.87439 29.83032 29.27793 29.98454 anatomical cell part;cytoplasm;cytoskeleton;dy 32 25 12 56.3 48.4 27.6 54.565 0 124.18 32.72588 192 17 20 18 18 20 22 24 22 2.17407404 0.171875648 0.811332226 4.053743694 P11679 P11679 Keratin, type II cytoskeletal 8 **Krt8** sp|P11679|K2C8_MOUSE Keratin, type II cytoskeletal 8 OS=Mus musculus OX=10090 GN=Krt8 PE=1 SV=4

26.50639725 26.23460007 26.61105 26.20732 26.77618 26.98555 26.77781 26.82542 biological r cell part;cyt Drug metab REACT_353 10 10 10 33.9 33.9 33.9 52.292 0 53.188 29.99469 33 6 5 7 6 5 8 7 7 2.170950748 0.173257732 0.451397419 4.047387326 P13439;D6 P13439 Uridine 5-monophosphate synthase;Orotate phos **Umps** sp|P13439|UMPS_MOUSE Uridine 5-monophosphate synthase OS=Mus musculus OX=10090 GN=Umps PE=1 SV=3

20.38799667 20.77876663 20.78659 20.20251 21.09165 22.07002 21.66071 21.41282 biological r cell part;int Ribosome biogenesis in 1 1 1 2 2 2 76.908 0.0076 1.5033 24.22634 1 0 0 0 0 1 1 1 1 2.165476934 0.174564103 1.019833565 4.036260333 Q8R2N2 Q8R2N2 Cirhin **Cirh1a** sp|Q8R2N2|UTP4_MOUSE U3 small nucleolar RNA-associated protein 4 homolog OS=Mus musculus OX=10090 GN=Utp4 PE=2 SV=3

21.27388954 19.38748932 20.84843 22.06364 23.68966 22.73948 23.43593 23.3009 biological a cell part;cell surface;ext REACT_286 1 1 1 2.5 2.5 2.5 128.41 0 3.7427 25.6919 6 0 0 0 0 1 1 1 1 2.157976553 0.176081633 2.398127556 4.021040667 A0A286YC A0A286YC Nidogen-2 **Nid2** tr|A0A286YCQ5|A0A286YCQ5_MOUSE Nidogen-2 (Fragment) OS=Mus musculus OX=10090 GN=Nid2 PE=1 SV=1;sp|O88322|NID2_MOUSE Nidogen-2 OS=Mus musculus OX=10090 GN=Nid2 PE=1 SV=2

23.26999283 21.94113159 22.51415 22.28198 21.1663 21.31735 19.71718 20.38867 anatomical cell part;cell projection;cilium;cytos 3 3 3 4.4 4.4 4.4 96.955 0.005933 1.6714 27.58248 4 1 1 1 1 1 1 2 1 2.138399387 0.183208122 -1.854440689 -3.981460302 A9Q751;G A9Q751 Cilia- and flagella-associated protein 221 **Cfap221** sp|A9Q751|PCDP1_MOUSE Cilia- and flagella-associated protein 221 OS=Mus musculus OX=10090 GN=Cfap221 PE=1 SV=1

19.51105499 19.25483894 22.70837 21.02434 25.66441 24.6142 24.55912 23.01637 anatomical cell part;Golgi apparatus;intracellul 1 1 1 8.4 8.4 8.4 17.127 0.009596 1.4338 26.79092 1 0 0 0 0 1 1 1 1 2.135704842 0.183353535 3.838870049 3.976028919 E9PZK7;Q8 E9PZK7;Q8 ADP-ribosylation factor-related protein 1 **Arfrp1** tr|E9PZK7|E9PZK7_MOUSE ADP-ribosylation factor-related protein 1 OS=Mus musculus OX=10090 GN=Arfrp1 PE=1 SV=1;sp|Q8BXL7|ARFRP_MOUSE ADP-ribosylation factor-related protein 1 OS=Mus musculus OX=10090 GN=Arfrp1 PE=1 SV=2

23.34190941 23.8912468 23.86041 23.58497 18.51622 22.28601 19.95869 21.03288 4 4 4 49.3 49.3 49.3 16.277 0 4.1711 26.89816 5 3 1 2 3 1 1 1 1 2.13376895 0.182653266 -3.221186161 -3.972129182 A2AA85;Q6 A2AA85;Q6 SUZ domain-containing protein 1 **Szrd1** tr|A2AA85|A2AA85_MOUSE SUZ domain-containing protein 1 (Fragment) OS=Mus musculus OX=10090 GN=Szrd1 PE=1 SV=1;sp|Q6NXN1|SZRD1_MOUSE SUZ domain-containing protein 1 OS=Mus musculus OX=10090 GN=Szrd1 PE=1 SV=1

23.5666256 24.16744804 25.26628 23.46526 19.39536 19.7708 21.2399 22.64475 biological a cell part;ext Peroxisome 5 5 5 18.4 18.4 18.4 27.003 0 5.5134 26.9257 7 3 3 3 1 1 1 1 2 2.12003693 0.18756 -3.353699684 -3.944524905 E9PXK7;D6 E9PXK7;D6 Peroxisomal membrane protein 11B **Pex11b** tr|E9PXK7|E9PXK7_MOUSE Peroxisomal membrane protein 11B OS=Mus musculus OX=10090 GN=Pex11b PE=1 SV=1;tr|D6RFQ2|D6RFQ2_MOUSE Predicted gene 42957 OS=Mus musculus OX=10090 GN=Gm42957 PE=1 SV=1;sp|Q9Z210|PX11B_MOUSE Peroxisomal membrane protein 11B OS=Mus mus

24.44978905 22.54747963 22.25237 23.14041 21.15927 21.43147 20.21274 19.79025 biological r cell part;cel Calcium signaling path 2 2 2 14.1 14.1 14.1 34.407 0 5.7487 25.11696 3 2 2 1 2 0 0 0 0 2.115420515 0.189651741 -2.449081898 -3.935267684 P56528 P56528 ADP-ribosyl cyclase/cyclic ADP-ribose hydrolase 1 **Cd38** sp|P56528|CD38_MOUSE ADP-ribosyl cyclase/cyclic ADP-ribose hydrolase 1 OS=Mus musculus OX=10090 GN=Cd38 PE=1 SV=2

27.54406929 27.54649925 27.36369 27.88518 26.73402 27.056 26.9914 27.22242 cell part;intracellular m REACT_278 5 5 5 26.3 26.3 26.3 32.118 0 23 30.52561 18 3 3 3 3 4 4 4 4 2.109951885 0.191069307 -0.583902359 -3.924316272 Q5SUD5;Q Q5SUD5;Q Protein SCO1 homolog, mitochondrial  **Sco1** tr|Q5SUD5|Q5SUD5_MOUSE Protein SCO1 homolog, mitochondrial OS=Mus musculus OX=10090 GN=Sco1 PE=1 SV=1;sp|Q5SUC9|SCO1_MOUSE Protein SCO1 homolog, mitochondrial OS=Mus musculus OX=10090 GN=Sco1 PE=1 SV=1

19.11296463 21.37883949 20.53806 20.07166 22.04677 22.02476 22.17048 22.37281 biosynthet cell part;ce Cytosolic D REACT_274 2 2 2 21.6 21.6 21.6 22.948 0 7.4256 25.12479 4 0 0 1 0 1 1 1 1 2.107612705 0.191014778 1.878323078 3.919636725 Q9D2C6;A Q9D2C6;A DNA-directed RNA polymerase III subunit RPC8 **Polr3h** sp|Q9D2C6|RPC8_MOUSE DNA-directed RNA polymerase III subunit RPC8 OS=Mus musculus OX=10090 GN=Polr3h PE=1 SV=2;tr|A0A2R8VK29|A0A2R8VK29_MOUSE DNA-directed RNA polymerase III subunit RPC8 OS=Mus musculus OX=10090 GN=Polr3h PE=1 SV=1

23.51026535 23.66383743 22.81605 22.57062 21.05892 22.3602 20.38872 21.10792 anatomical cell part;cell projection; REACT_361 3 3 3 10.2 10.2 10.2 47.952 0 5.7197 25.42085 3 2 1 1 1 1 1 0 0 2.101877082 0.191843137 -1.911253452 -3.908174871 Q8R3P7;D Q8R3P7;D Clusterin-associated protein 1 **Cluap1** sp|Q8R3P7|CLUA1_MOUSE Clusterin-associated protein 1 OS=Mus musculus OX=10090 GN=Cluap1 PE=1 SV=1;tr|D3Z1F2|D3Z1F2_MOUSE Clusterin-associated protein 1 (Fragment) OS=Mus musculus OX=10090 GN=Cluap1 PE=1 SV=9;tr|E9Q8M4|E9Q8M4_MOUSE Clusterin-associated prot

22.69398117 22.83477974 25.34895 22.95312 21.74056 20.28467 20.11638 19.86242 biological r cell part;cell projection;centrosome 5 5 5 25.8 25.8 25.8 28.378 0 4.9075 26.88332 5 1 1 3 2 2 2 1 1 2.091476658 0.194926829 -2.956697464 -3.887435457 Q8QZT2 Q8QZT2 Centriole, cilia and spindle-associated protein **Ccsap** sp|Q8QZT2|CCSAP_MOUSE Centriole, cilia and spindle-associated protein OS=Mus musculus OX=10090 GN=Ccsap PE=1 SV=1

26.36601257 26.34597397 26.57542 26.39761 26.6088 27.31147 27.242 27.35548 autophagy; cell part;cyt Regulation of autophag 6 6 5 56.4 56.4 50.4 13.667 0 7.6017 30.21037 27 4 4 4 3 5 6 6 5 2.084700018 0.196718447 0.708180904 3.873952842 P60521 P60521 Gamma-aminobutyric acid receptor-associated pr **Gabarapl2** sp|P60521|GBRL2_MOUSE Gamma-aminobutyric acid receptor-associated protein-like 2 OS=Mus musculus OX=10090 GN=Gabarapl2 PE=1 SV=1

24.99308586 24.85723495 25.09648 24.88054 25.30205 25.89301 25.64491 25.32701 catabolic process;cellul Inositol ph REACT_272 7 7 7 15.1 15.1 15.1 99.971 0 20.087 28.78251 27 3 3 3 3 2 6 5 5 2.082592105 0.196850242 0.584907532 3.869763905 Q3UEQ1;E9 Q3UEQ1;E9 Type I inositol 3,4-bisphosphate 4-phosphatase **Inpp4a** tr|Q3UEQ1|Q3UEQ1_MOUSE Phosphatidylinositol-3,4-bisphosphate 4-phosphatase OS=Mus musculus OX=10090 GN=Inpp4a PE=1 SV=1;tr|E9Q9A0|E9Q9A0_MOUSE Phosphatidylinositol-3,4-bisphosphate 4-phosphatase OS=Mus musculus OX=10090 GN=Inpp4a PE=1 SV=2;tr|F6V2U0|F6V2U0

27.57014847 27.96184921 27.57304 27.40422 28.08096 28.06384 28.10099 28.09482 cell cycle;ce cell part;cytoplasmic membrane-bo 17 9 9 60.8 43.5 43.5 49.619 0 72.192 31.16273 69 7 7 6 6 9 8 8 9 2.078288588 0.198615385 0.457836628 3.861218974 Q9R1T4;A2 Q9R1T4 Septin-6 **6-Sep** sp|Q9R1T4|SEPT6_MOUSE Septin-6 OS=Mus musculus OX=10090 GN=Septin6 PE=1 SV=4

20.84256744 20.05608749 19.36762 20.31221 21.15128 21.95088 22.00103 21.28811 1 1 1 3.4 3.4 3.4 66.798 0 4.6631 24.23462 3 0 0 0 0 1 1 1 1 2.072818928 0.199827751 1.453201771 3.850372514 Q8C318;A2 Q8C318;A2 UHRF1-binding protein 1-like **Uhrf1bp1l** tr|Q8C318|Q8C318_MOUSE UHRF1-binding protein 1-like (Fragment) OS=Mus musculus OX=10090 GN=Uhrf1bp1l PE=1 SV=1;sp|A2RSJ4|UH1BL_MOUSE UHRF1-binding protein 1-like OS=Mus musculus OX=10090 GN=Uhrf1bp1l PE=1 SV=2

21.78536415 21.42910385 22.57607 22.07503 20.87997 20.96432 21.10156 20.29664 biological r cell part;cyt Glycerolipi REACT_348 2 2 2 3.3 3.3 3.3 135.21 0 3.7076 24.09362 4 1 2 1 1 0 0 1 0 2.071938906 0.199466667 -1.155769348 -3.848628864 E9PUQ8 E9PUQ8 Diacylglycerol kinase **Dgkd** sp|E9PUQ8|DGKD_MOUSE Diacylglycerol kinase delta OS=Mus musculus OX=10090 GN=Dgkd PE=1 SV=1

30.49676514 30.68059921 30.76659 30.66704 30.88344 30.88016 30.83598 30.91371 biological r cell part;ext Carbon fixa REACT_333 34 34 34 55.8 55.8 55.8 79.921 0 219.67 34.02622 266 27 28 26 28 27 28 29 28 2.069034719 0.19943128 0.225569725 3.842877437 Q91ZA3;H3 Q91ZA3 Propionyl-CoA carboxylase alpha chain, mitochon  **Pcca** sp|Q91ZA3|PCCA_MOUSE Propionyl-CoA carboxylase alpha chain, mitochondrial OS=Mus musculus OX=10090 GN=Pcca PE=1 SV=2

20.75918007 19.60961914 21.96017 20.99217 23.28785 22.71708 22.96012 22.36188 cell part REACT_291 1 1 1 9.1 9.1 9.1 23.528 0 3.6824 25.30556 3 0 0 0 0 1 1 1 1 2.068641754 0.198698113 2.001448631 3.84209955 Q9CY24 Q9CY24 Transmembrane protein 179B **Tmem179b** sp|Q9CY24|T179B_MOUSE Transmembrane protein 179B OS=Mus musculus OX=10090 GN=Tmem179b PE=1 SV=1

20.25522041 22.12033081 19.16615 21.31925 23.17711 23.16782 24.52199 23.13411 biological r cell part;cytoplasm;intr REACT_291 2 2 2 16.7 16.7 16.7 26.529 0 12.586 26.10653 2 0 0 0 0 2 1 2 2 2.062796278 0.200037559 2.785019875 3.830537644 Q9CR11 Q9CR11 YEATS domain-containing protein 4 **Yeats4** sp|Q9CR11|YETS4_MOUSE YEATS domain-containing protein 4 OS=Mus musculus OX=10090 GN=Yeats4 PE=2 SV=1

21.18400192 19.51076508 20.6726 26.25519 28.33471 27.6013 27.09114 27.70418 cellular lipi cell part;cyt Inositol ph REACT_272 5 4 4 5.5 5 5 154.71 0.000165 3.0759 30.63019 8 1 1 1 1 2 3 3 1 2.056378246 0.20271028 5.777192593 3.817863557 D3YZB2;F8 D3YZB2;F8 Synaptojanin-2 **Synj2** tr|D3YZB2|D3YZB2_MOUSE Phosphoinositide 5-phosphatase OS=Mus musculus OX=10090 GN=Synj2 PE=1 SV=1;tr|F8WHD8|F8WHD8_MOUSE Phosphoinositide 5-phosphatase OS=Mus musculus OX=10090 GN=Synj2 PE=1 SV=1;sp|Q9D2G5|SYNJ2_MOUSE Synaptojanin-2 OS=Mus musculus OX=1009

23.75378799 22.7901268 24.27916 24.04009 19.12133 19.92273 21.46889 22.29077 amine meta cell part;cytoplasm;extracellular org 2 2 2 25.8 25.8 25.8 20.489 0 23.77 25.8087 6 2 1 1 1 0 0 0 0 2.05431973 0.202288372 -3.014863014 -3.813802961 O88593 O88593 Peptidoglycan recognition protein 1 **Pglyrp1** sp|O88593|PGRP1_MOUSE Peptidoglycan recognition protein 1 OS=Mus musculus OX=10090 GN=Pglyrp1 PE=1 SV=1

23.88063622 23.37145805 23.70683 23.10034 18.6945 21.86107 21.72483 20.12407 catabolic p cell part;en Parkinson's disease;Pro 2 2 2 16.8 16.8 16.8 25.848 0 4.1202 25.64955 3 1 2 2 1 0 0 0 0 2.044460331 0.206425926 -2.913696289 -3.794384527 B0QZM1;B B0QZM1;B Ubiquitin-conjugating enzyme E2 J2 **Ube2j2** tr|B0QZM1|B0QZM1_MOUSE Ubiquitin-conjugating enzyme E2 J2 OS=Mus musculus OX=10090 GN=Ube2j2 PE=1 SV=1;tr|B1ASK8|B1ASK8_MOUSE Ubiquitin-conjugating enzyme E2 J2 OS=Mus musculus OX=10090 GN=Ube2j2 PE=1 SV=1;sp|Q6P073|UB2J2_MOUSE Ubiquitin-conjugating enzyme

24.94454002 25.22762299 24.99123 24.76086 24.4899 24.60196 24.69503 24.42944 biological r cell part;cyt Insulin sign REACT_289 3 3 3 42.2 42.2 42.2 14.703 0 8.9529 28.15258 13 3 2 3 2 2 3 2 3 2.040799751 0.20875576 -0.426981926 -3.787187501 M0QWD1; M0QWD1; Eukaryotic translation initiation factor 4E type 2 **Eif4e2** tr|M0QWD1|M0QWD1_MOUSE Eukaryotic translation initiation factor 4E type 2 (Fragment) OS=Mus musculus OX=10090 GN=Eif4e2 PE=1 SV=1;tr|D3Z730|D3Z730_MOUSE Eukaryotic translation initiation factor 4E type 2 OS=Mus musculus OX=10090 GN=Eif4e2 PE=1 SV=1;tr|Q0P6

27.24472427 27.6102047 27.20834 27.5786 27.09154 26.05681 26.15493 26.46256 cell part;cell projection;cytoplasm;G 2 2 2 4.9 4.9 4.9 81.332 0.007605 1.5089 30.3742 6 1 1 1 1 1 2 2 2 2.035916089 0.210678899 -0.969006538 -3.777596378 Q9DBQ7 Q9DBQ7 Protein-associating with the carboxyl-terminal do **Scyl3** sp|Q9DBQ7|PACE1_MOUSE Protein-associating with the carboxyl-terminal domain of ezrin OS=Mus musculus OX=10090 GN=Scyl3 PE=1 SV=3

28.98533249 28.98880196 28.8079 28.95191 28.67815 28.72745 28.79634 28.77621 biological r cell part;cytoplasm;extracellular org 8 8 3 53.1 53.1 26.9 16.367 0 45.322 31.94084 83 8 7 7 7 7 7 7 8 2.02879326 0.214063927 -0.188949108 -3.763629333 Q9D2M8;A Q9D2M8;A Ubiquitin-conjugating enzyme E2 variant 2 **Ube2v2;Ube2v1** sp|Q9D2M8|UB2V2_MOUSE Ubiquitin-conjugating enzyme E2 variant 2 OS=Mus musculus OX=10090 GN=Ube2v2 PE=1 SV=4;tr|A6X925|A6X925_MOUSE Ubiquitin-conjugating enzyme E2 variant 2 OS=Mus musculus OX=10090 GN=Ube2v2 PE=1 SV=1;tr|B7ZBY6|B7ZBY6_MOUSE Ubiquitin-conj

28.64578056 28.87622261 28.75232 28.91271 29.09718 29.16796 28.98818 29.02316 biological r cell part;ext Pathways i REACT_276 8 8 8 81.4 81.4 81.4 13.17 0 13.42 32.21011 84 7 7 7 7 7 8 8 7 2.01508257 0.221072727 0.272363186 3.736816036 P62869;A0 P62869;A0 Transcription elongation factor B polypeptide 2 **Tceb2** sp|P62869|ELOB_MOUSE Elongin-B OS=Mus musculus OX=10090 GN=Elob PE=1 SV=1;tr|A0A3B2WBM3|A0A3B2WBM3_MOUSE Elongin-B OS=Mus musculus OX=10090 GN=Elob PE=1 SV=1

34.7339325 34.72735596 34.56041 34.75658 34.86701 34.89681 34.83262 34.88667 anatomical cell part;cel Antigen pro REACT_278 64 64 36 79.6 79.6 48.8 83.28 0 323.31 38.06803 991 60 58 60 59 62 60 59 61 2.008492156 0.223746606 0.17620945 3.723960902 P11499;E9 P11499 Heat shock protein HSP 90-beta **Hsp90ab1** sp|P11499|HS90B_MOUSE Heat shock protein HSP 90-beta OS=Mus musculus OX=10090 GN=Hsp90ab1 PE=1 SV=3

20.29895592 20.53107452 21.36397 20.31501 19.39452 18.61465 19.73332 19.54231 biosynthet cell part;ma Glycosylph REACT_336 1 1 1 4.4 4.4 4.4 25.679 0.004506 1.8018 21.88815 3 0 0 0 0 1 1 1 1 2.005531051 0.225945946 -1.306052685 -3.718192049 F7BU94;A0 F7BU94;A0 Phosphatidylinositol N-acetylglucosaminyltransfe **Pigq** tr|F7BU94|F7BU94_MOUSE Phosphatidylinositol N-acetylglucosaminyltransferase subunit Q (Fragment) OS=Mus musculus OX=10090 GN=Pigq PE=1 SV=2;tr|A0A140LIE2|A0A140LIE2_MOUSE Phosphatidylinositol N-acetylglucosaminyltransferase subunit Q OS=Mus musculus OX=100

24.62120247 24.78061676 24.71476 24.72546 25.25879 25.11625 25.37368 24.84824 biological r cell part;cyt Glycerolipi REACT_348 6 6 6 9.2 9.2 9.2 130.1 0 11.953 28.39881 11 1 1 2 1 2 3 4 5 2.004804915 0.22561435 0.438726425 3.716778046 A0A2I3BQ4 A0A2I3BQ4 Diacylglycerol kinase **Dgkh** tr|A0A2I3BQ48|A0A2I3BQ48_MOUSE Diacylglycerol kinase OS=Mus musculus OX=10090 GN=Dgkh PE=1 SV=1;tr|A0A2I3BQ43|A0A2I3BQ43_MOUSE Diacylglycerol kinase OS=Mus musculus OX=10090 GN=Dgkh PE=1 SV=1;tr|A0JP53|A0JP53_MOUSE Diacylglycerol kinase OS=Mus musculus OX=

24.05001068 21.22600555 20.64447 20.65352 24.86806 24.60987 24.70483 24.43078 3 3 3 4.8 4.8 4.8 109.95 0.008308 1.4698 27.52731 4 1 0 0 0 2 3 3 2 1.983942873 0.238982143 3.009881973 3.676264328 Q3U0J8 Q3U0J8 TBC1 domain family member 2B **Tbc1d2b** sp|Q3U0J8|TBD2B_MOUSE TBC1 domain family member 2B OS=Mus musculus OX=10090 GN=Tbc1d2b PE=1 SV=2

23.80901909 23.72619247 26.32605 23.18394 19.88866 19.6552 22.1312 21.62528 cell part;int Glycosamin REACT_294 3 3 3 11.8 11.8 11.8 62.185 0 10.419 27.49503 5 1 2 2 1 0 0 1 1 1.981907583 0.239093333 -3.436214447 -3.672323235 Q08890;F6 Q08890;F6 Iduronate 2-sulfatase **Ids** sp|Q08890|IDS_MOUSE Iduronate 2-sulfatase OS=Mus musculus OX=10090 GN=Ids PE=2 SV=3;tr|F6X9C5|F6X9C5_MOUSE Iduronate 2-sulfatase (Fragment) OS=Mus musculus OX=10090 GN=Ids PE=1 SV=8

27.29780388 27.13192749 27.36469 27.14783 27.07867 26.59349 26.65444 26.8169 anatomical cell part;cytoplasm;cytoplasmic me 2 2 2 36.6 36.6 36.6 12.605 0 8.7342 30.27398 17 2 2 2 2 1 2 1 1 1.981758517 0.238336283 -0.449687958 -3.672034666 Q9D8T7;F8 Q9D8T7;F8 SRA stem-loop-interacting RNA-binding protein, m **Slirp** sp|Q9D8T7|SLIRP_MOUSE SRA stem-loop-interacting RNA-binding protein, mitochondrial OS=Mus musculus OX=10090 GN=Slirp PE=1 SV=2;tr|F8WHU8|F8WHU8_MOUSE SRA stem-loop-interacting RNA-binding protein, mitochondrial (Fragment) OS=Mus musculus OX=10090 GN=Slirp

24.05001068 20.62570572 19.51472 19.92542 24.82934 24.45782 25.07055 25.0426 anatomical cell part;int Axon guida REACT_280 6 6 6 4.3 4.3 4.3 211.53 0 8.8608 27.7377 5 2 0 1 2 3 3 4 3 1.978415788 0.240123348 3.82111454 3.66556649 P70207;Q8 P70207 Plexin-A2 **Plxna2** sp|P70207|PLXA2_MOUSE Plexin-A2 OS=Mus musculus OX=10090 GN=Plxna2 PE=1 SV=2

21.119133 20.38406181 20.18854 20.82582 21.41787 21.65508 21.71243 21.22835 biological r cell part;ma Basal trans REACT_279 1 1 1 1.9 1.9 1.9 89.125 0.0072 1.5652 24.02884 3 0 0 0 0 1 1 1 1 1.977931136 0.239807018 0.874043465 3.664629139 P49135 P49135 TFIIH basal transcription factor complex helicase X **Ercc3** sp|P49135|ERCC3_MOUSE General transcription and DNA repair factor IIH helicase subunit XPB OS=Mus musculus OX=10090 GN=Ercc3 PE=2 SV=1

27.45057487 27.31656837 27.25467 27.4753 27.6526 27.68264 27.5974 27.51299 biological a cell part;cytoplasm;intr REACT_311 11 11 11 50.8 50.8 50.8 43.221 0 99.763 30.70775 77 9 8 7 10 9 9 10 7 1.976637232 0.239406114 0.237133026 3.662127195 Q8BP48;A0 Q8BP48 Methionine aminopeptidase 1 **Metap1** sp|Q8BP48|MAP11_MOUSE Methionine aminopeptidase 1 OS=Mus musculus OX=10090 GN=Metap1 PE=1 SV=1

20.1269474 21.20508575 20.18066 22.15529 22.80849 22.88291 22.4437 22.71354 biological r cell part;cytoplasm;intracellular me 2 2 2 3.8 3.8 3.8 104.48 0 5.6272 25.35468 2 1 0 0 0 1 1 1 1 1.973444375 0.241165217 1.795162678 3.655956833 A0A1Y7VJH A0A1Y7VJH Apoptosis-stimulating of p53 protein 1 **Ppp1r13b** tr|A0A1Y7VJH3|A0A1Y7VJH3_MOUSE Apoptosis-stimulating of p53 protein 1 (Fragment) OS=Mus musculus OX=10090 GN=Ppp1r13b PE=1 SV=1;sp|Q62415|ASPP1_MOUSE Apoptosis-stimulating of p53 protein 1 OS=Mus musculus OX=10090 GN=Ppp1r13b PE=1 SV=2

20.58339691 20.07523346 20.15775 19.65017 20.90345 21.04707 21.89401 20.98801 biological r cell part;intracellular;synapse 3 3 3 4.9 4.9 4.9 53.837 0 5.4205 23.92922 4 0 0 0 0 2 3 2 1 1.967365767 0.244727273 1.091499329 3.644223216 Q3V2R3 Q3V2R3 **Chn2** tr|Q3V2R3|Q3V2R3_MOUSE Chimaerin OS=Mus musculus OX=10090 GN=Chn2 PE=1 SV=1

22.77742004 23.03474808 22.48525 23.55391 19.30008 19.13947 21.70117 21.37242 biological r cell part;cytoplasm 5 5 1 8 8 1.4 70.273 0 3.1528 25.89546 7 2 2 1 2 2 5 2 1 1.964766108 0.244086207 -2.584547043 -3.639210494 E9Q1U6 E9Q1U6 **Ctif** tr|E9Q1U6|E9Q1U6_MOUSE CBP80/20-dependent translation initiation factor OS=Mus musculus OX=10090 GN=Ctif PE=1 SV=1

23.44480705 23.25024605 23.1325 23.39257 24.42892 23.54498 24.41493 24.02859 biological r cell part REACT_301 2 2 2 4.2 4.2 4.2 73.649 0 3.4104 27.1032 13 2 2 2 2 1 2 2 2 1.962331451 0.244085837 0.799326897 3.634518873 E9QJY0;P1 E9QJY0;P1 Cationic amino acid transporter 2 **Slc7a2** tr|E9QJY0|E9QJY0_MOUSE Cationic amino acid transporter 2 OS=Mus musculus OX=10090 GN=Slc7a2 PE=1 SV=1;sp|P18581|CTR2_MOUSE Cationic amino acid transporter 2 OS=Mus musculus OX=10090 GN=Slc7a2 PE=1 SV=3

25.19449043 25.86985397 26.16173 25.89668 27.04906 26.98902 26.90231 26.23564 cellular pro cell part;cytoplasm;cytoskeleton;int 4 4 4 5.5 5.5 5.5 115.45 0 6.0079 29.35111 8 3 2 3 1 2 2 2 2 1.958262835 0.245965812 1.013315678 3.626684929 H3BIZ7;Q3 H3BIZ7;Q3 FERM domain-containing protein 4A **Frmd4a** tr|H3BIZ7|H3BIZ7_MOUSE FERM domain-containing protein 4A OS=Mus musculus OX=10090 GN=Frmd4a PE=1 SV=1;tr|Q3TB04|Q3TB04_MOUSE FERM domain-containing protein 4A OS=Mus musculus OX=10090 GN=Frmd4a PE=1 SV=1;sp|Q8BIE6|FRM4A_MOUSE FERM domain-containing protein

32.239254 32.25414276 32.30756 32.41815 32.64229 32.59531 32.59257 32.39077 anatomical cell part;cytoplasm;cyto REACT_271 29 29 28 68.6 68.6 66.9 51.564 0 323.31 35.69675 385 27 27 24 28 26 26 26 25 1.95637018 0.245480851 0.250455856 3.623043401 P40124;B1 P40124 Adenylyl cyclase-associated protein 1 **Cap1** sp|P40124|CAP1_MOUSE Adenylyl cyclase-associated protein 1 OS=Mus musculus OX=10090 GN=Cap1 PE=1 SV=4

23.06766129 22.9575901 22.13166 26.4821 20.24778 20.058 19.96436 20.3586 3 3 3 1.8 1.8 1.8 211.54 0.009593 1.4297 26.34009 3 1 2 1 1 0 0 1 1 1.954284438 0.245627119 -3.502569675 -3.619032346 E9Q5M6 E9Q5M6 **Cfap44** sp|E9Q5M6|CFA44_MOUSE Cilia- and flagella-associated protein 44 OS=Mus musculus OX=10090 GN=Cfap44 PE=2 SV=1

22.84272385 20.36962891 21.29665 20.76986 23.80705 23.00508 23.12888 23.68271 anatomical cell part;cytoplasm;cyto REACT_319 2 2 2 8.4 8.4 8.4 47.976 0 4.6045 26.01984 5 1 0 0 0 1 2 2 2 1.951362394 0.245907173 2.086217403 3.613416499 E0CZ81;Q8 E0CZ81;Q8 Tubulin-specific chaperone E **Tbce** tr|E0CZ81|E0CZ81_MOUSE Tubulin-folding cofactor E OS=Mus musculus OX=10090 GN=Tbce PE=1 SV=1;sp|Q8CIV8|TBCE_MOUSE Tubulin-specific chaperone E OS=Mus musculus OX=10090 GN=Tbce PE=1 SV=1

22.96782494 23.1395092 23.07028 23.17681 22.55913 21.10336 21.01531 19.95797 biosynthet cell part;ma Folate bios REACT_298 3 3 3 9.7 9.7 9.7 69.858 0 10.716 25.64232 5 3 2 3 1 1 1 1 0 1.943262654 0.248941176 -1.929663181 -3.597870877 Q5RKZ7;G3 Q5RKZ7 Molybdenum cofactor biosynthesis protein 1;Cyc **Mocs1** sp|Q5RKZ7|MOCS1_MOUSE Molybdenum cofactor biosynthesis protein 1 OS=Mus musculus OX=10090 GN=Mocs1 PE=1 SV=2

24.27419472 24.89157104 24.47017 24.39015 21.57344 21.0838 23.90003 20.37237 catabolic p cell part;int DNA replication 4 4 4 50 50 50 17.883 0 9.4324 27.4847 9 3 2 2 3 0 1 1 0 1.933169506 0.255280335 -2.77410984 -3.578542702 A0A494BB A0A494BB Ribonuclease H2 subunit C **Rnaseh2c** tr|A0A494BBB6|A0A494BBB6_MOUSE Ribonuclease H2 subunit C (Fragment) OS=Mus musculus OX=10090 GN=Rnaseh2c PE=1 SV=1;tr|A0A494B9C7|A0A494B9C7_MOUSE Ribonuclease H2 subunit C (Fragment) OS=Mus musculus OX=10090 GN=Rnaseh2c PE=1 SV=1;sp|Q9CQ18|RNH2C_MOUSE Ribo

21.5749321 21.98628616 19.47392 20.67959 23.92597 22.72422 22.96261 22.9344 1 1 1 4.7 4.7 4.7 37.574 0.001571 2.3533 25.53169 1 0 0 0 0 1 1 1 1 1.931133557 0.255366667 2.208114624 3.574649699 Q7TSF4 Q7TSF4 Leucine-rich repeat-containing protein 75A **Lrrc75a** sp|Q7TSF4|LR75A_MOUSE Leucine-rich repeat-containing protein 75A OS=Mus musculus OX=10090 GN=Lrrc75a PE=1 SV=1

21.26258659 21.78364754 22.93043 22.9767 20.18748 20.70833 20.99049 20.54698 cell part;Golgi apparatus;intracellul 3 3 3 7.1 7.1 7.1 62.055 0.000164 2.9771 25.74312 5 2 1 1 1 2 1 1 1 1.921914509 0.259419087 -1.630019188 -3.557045896 Q8BUV8 Q8BUV8 Protein GPR107 **Gpr107** sp|Q8BUV8|GP107_MOUSE Protein GPR107 OS=Mus musculus OX=10090 GN=Gpr107 PE=1 SV=2

25.69553757 24.90118027 24.82953 24.89717 24.34509 23.67023 24.29299 24.30208 amine meta cell part;intracellular membrane-bo 1 1 1 16.8 16.8 16.8 16.667 0 29.75 27.86378 8 1 1 1 1 1 1 1 1 1.920103863 0.25953719 -0.928257465 -3.553593108 Q8CBY0 Q8CBY0 Glutamyl-tRNA(Gln) amidotransferase subunit C,  **Gatc** sp|Q8CBY0|GATC_MOUSE Glutamyl-tRNA(Gln) amidotransferase subunit C, mitochondrial OS=Mus musculus OX=10090 GN=Gatc PE=1 SV=1

27.07561493 27.15199471 26.99237 26.93225 27.24 27.19178 27.44008 27.30591 cellular pro cell part;cyt Toxoplasmosis 12 12 10 44 44 35.2 48.493 0 78.82 30.36223 46 7 8 7 8 6 7 7 7 1.91927542 0.259407407 0.256385326 3.552013828 Q9DCE9 Q9DCE9 **Igtp** tr|Q9DCE9|Q9DCE9_MOUSE Interferon gamma-induced GTPase OS=Mus musculus OX=10090 GN=Igtp PE=1 SV=1

24.24038124 25.08768272 24.48677 24.42496 25.35595 25.42021 25.31648 25.02559 cellular met cell part;cytoplasm 10 10 10 21.8 21.8 21.8 67.969 0 80.027 28.37642 26 3 6 3 2 3 7 6 4 1.913321466 0.262852459 0.719611645 3.540673006 O35239;A0 O35239 Tyrosine-protein phosphatase non-receptor type **Ptpn9** sp|O35239|PTN9_MOUSE Tyrosine-protein phosphatase non-receptor type 9 OS=Mus musculus OX=10090 GN=Ptpn9 PE=1 SV=2

28.43125534 28.71969986 28.34769 28.63989 28.85234 29.06733 28.95676 28.77806 anatomical cell part;cyt Chemokine REACT_306 20 20 20 62 62 62 50.716 0 78.603 32.04928 121 16 17 17 14 14 18 17 20 1.905943903 0.266612245 0.378988743 3.526643239 P41241;A0 P41241 Tyrosine-protein kinase CSK **Csk** sp|P41241|CSK_MOUSE Tyrosine-protein kinase CSK OS=Mus musculus OX=10090 GN=Csk PE=1 SV=2

26.19865417 26.37577248 25.74229 26.2661 26.63699 26.57658 26.6979 26.64113 cell death;c cell part;cytoplasm;cyto REACT_315 20 13 13 29.9 23.6 23.6 83.886 0 48.458 29.76512 29 6 7 4 4 5 10 10 10 1.881131625 0.284276423 0.492445469 3.479640529 Q8BHL5;F6 Q8BHL5 Engulfment and cell motility protein 2 **Elmo2** sp|Q8BHL5|ELMO2_MOUSE Engulfment and cell motility protein 2 OS=Mus musculus OX=10090 GN=Elmo2 PE=1 SV=1

22.03761482 22.04129601 23.13597 21.77872 20.03193 18.75956 20.71704 21.14767 4 4 4 5.6 5.6 5.6 137.32 0.004639 1.757 26.37146 3 2 2 1 1 1 2 2 0 1.869829251 0.293117409 -2.084352493 -3.458322148 Q9CXK9;D Q9CXK9;D RNA-binding protein 33 **Rbm33** sp|Q9CXK9|RBM33_MOUSE RNA-binding protein 33 OS=Mus musculus OX=10090 GN=Rbm33 PE=1 SV=2;tr|D3Z5I9|D3Z5I9_MOUSE RNA-binding protein 33 OS=Mus musculus OX=10090 GN=Rbm33 PE=1 SV=1;tr|F6RNF9|F6RNF9_MOUSE RNA-binding protein 33 (Fragment) OS=Mus musculus OX=1

20.73051071 19.45271301 21.98203 19.87015 22.61856 21.96169 22.88084 22.826 anatomical cell part;membrane 3 3 3 16.9 16.9 16.9 39.968 0 8.0511 25.74684 5 1 1 2 0 1 1 1 1 1.86824049 0.293693548 2.062923908 3.455330023 Q3TUA9 Q3TUA9 Protein O-mannose kinase **Pomk** sp|Q3TUA9|SG196_MOUSE Protein O-mannose kinase OS=Mus musculus OX=10090 GN=Pomk PE=1 SV=2

28.7850647 29.00996017 28.80391 28.92895 29.02704 29.09695 29.10011 29.07584 biological r cell part;cell projection;cytoplasm;c 17 15 15 52.9 48.9 48.9 40.079 0 98.692 32.17102 78 11 12 12 13 11 10 10 10 1.867262261 0.293301205 0.193019867 3.453488275 Q91YR1;D3 Q91YR1 Twinfilin-1 **Twf1** sp|Q91YR1|TWF1_MOUSE Twinfilin-1 OS=Mus musculus OX=10090 GN=Twf1 PE=1 SV=2

21.00343132 19.24804306 19.8352 22.07083 18.45411 18.36995 18.39435 18.25382 anatomical cell part;cel Basal cell c REACT_278 1 1 1 2.9 2.9 2.9 65.85 0.008594 1.4613 20.92622 2 0 0 0 0 1 1 1 1 1.866951017 0.292384 -2.171318054 -3.452902373 Q3UMJ8;Q Q3UMJ8;Q Zinc finger protein GLI2;Transcriptional activator G **Gli3;Gli2** tr|Q3UMJ8|Q3UMJ8_MOUSE Transcriptional activator GLI3 (Fragment) OS=Mus musculus OX=10090 GN=Gli3 PE=1 SV=1;sp|Q0VGT2|GLI2_MOUSE Zinc finger protein GLI2 OS=Mus musculus OX=10090 GN=Gli2 PE=1 SV=2;sp|Q61602|GLI3_MOUSE Transcriptional activator GLI3 OS=Mus

22.8190155 20.5504837 20.06985 20.38921 23.40263 23.21827 22.75896 23.35917 anatomical cell part;cell projection; REACT_362 2 2 2 3.5 3.5 3.5 79.931 0 4.5126 25.79082 8 1 0 0 0 2 2 2 2 1.86556339 0.291681275 2.227619648 3.450290756 Q9CWF6 Q9CWF6 Bardet-Biedl syndrome 2 protein homolog **Bbs2** sp|Q9CWF6|BBS2_MOUSE Bardet-Biedl syndrome 2 protein homolog OS=Mus musculus OX=10090 GN=Bbs2 PE=1 SV=1

24.29740143 24.46138954 24.6221 24.43301 24.93975 25.27491 25.18048 24.6184 establishm cell part;intracellular membrane-bo 8 8 8 35.7 35.7 35.7 32.63 0 20.139 28.14014 30 4 4 4 4 4 6 7 6 1.848354474 0.303428571 0.549911976 3.417972998 Q5SWT3;A Q5SWT3;A Solute carrier family 25 member 35 **Slc25a35** sp|Q5SWT3|S2535_MOUSE Solute carrier family 25 member 35 OS=Mus musculus OX=10090 GN=Slc25a35 PE=1 SV=2;tr|A0A0A0MQ70|A0A0A0MQ70_MOUSE Solute carrier family 25 member 35 OS=Mus musculus OX=10090 GN=Slc25a35 PE=1 SV=1

29.28881264 29.39611435 29.19779 29.3441 29.43446 29.60072 29.65378 29.46221 anatomical cell part;ce Axon guida REACT_280 42 42 36 39.6 39.6 34.9 160.61 0 229.67 32.74047 222 32 30 29 27 31 35 37 31 1.839692945 0.310545455 0.231088161 3.401756055 F8VPK5;A0 F8VPK5;A0 Rho-associated protein kinase;Rho-associated pro **Rock2** tr|F8VPK5|F8VPK5_MOUSE Rho-associated protein kinase OS=Mus musculus OX=10090 GN=Rock2 PE=1 SV=1;tr|A0A1Y7VMN0|A0A1Y7VMN0_MOUSE Rho-associated protein kinase 2 (Fragment) OS=Mus musculus OX=10090 GN=Rock2 PE=1 SV=1;sp|P70336|ROCK2_MOUSE Rho-associated prot

21.07978439 20.7147274 21.03432 21.51424 21.47041 22.36975 22.35877 21.85064 anatomical cell part;cytoplasm;nucleoplasm 1 1 1 2.1 2.1 2.1 100.78 0 3.7732 24.68736 2 0 0 0 0 1 1 1 1 1.838519107 0.309905512 0.926623821 3.399560793 Q8VBW5 Q8VBW5 HMG box transcription factor BBX **Bbx** sp|Q8VBW5|BBX_MOUSE HMG box transcription factor BBX OS=Mus musculus OX=10090 GN=Bbx PE=1 SV=2

31.83684349 31.81615829 31.8671 31.83176 31.65527 31.66831 31.44973 31.73283 biosynthet cell part;ext Ribosome REACT_279 11 11 11 80.9 80.9 80.9 11.651 0 107.76 34.98749 88 10 10 6 9 8 9 11 9 1.836989104 0.309568627 -0.211429596 -3.396700345 P99027;A0 P99027;A0 60S acidic ribosomal protein P2 **Rplp2** sp|P99027|RLA2_MOUSE 60S acidic ribosomal protein P2 OS=Mus musculus OX=10090 GN=Rplp2 PE=1 SV=3;tr|A0A5F8MPY2|A0A5F8MPY2_MOUSE 60S acidic ribosomal protein P2 OS=Mus musculus OX=10090 GN=Rplp2-ps1 PE=3 SV=1

22.35517311 22.04076195 22.04856 21.76203 20.64615 21.72103 21.09249 20.31178 cellular met cell part;ma Ribosome biogenesis in 1 1 1 7.8 7.8 7.8 29.473 0.004778 1.7337 24.1055 1 1 1 1 1 0 0 0 0 1.828697855 0.313984375 -1.108768463 -3.38121687 O88796 O88796 Ribonuclease P protein subunit p30 **Rpp30** sp|O88796|RPP30_MOUSE Ribonuclease P protein subunit p30 OS=Mus musculus OX=10090 GN=Rpp30 PE=1 SV=1

31.560606 31.46509361 31.4747 31.57326 31.61601 31.77992 32.00919 31.82297 anatomical cell part;cyt Cell cycle;N REACT_291 24 22 21 78.5 78.5 78.5 28.211 0 178.67 35.02015 249 20 19 19 19 18 20 20 19 1.823956093 0.315642023 0.288607597 3.372375175 P68510 P68510 14-3-3 protein eta **Ywhah** sp|P68510|1433F_MOUSE 14-3-3 protein eta OS=Mus musculus OX=10090 GN=Ywhah PE=1 SV=2

28.14447784 28.24240875 28.29518 28.0876 28.36861 28.46447 28.31147 28.52223 cellular comcell part;en Cell cycle - Caulobacter 8 8 8 37.1 37.1 37.1 29.8 0 27.324 31.6168 75 6 6 6 6 8 8 8 7 1.822690224 0.315426357 0.22427845 3.37001641 O88696 O88696 ATP-dependent Clp protease proteolytic subunit,  **Clpp** sp|O88696|CLPP_MOUSE ATP-dependent Clp protease proteolytic subunit, mitochondrial OS=Mus musculus OX=10090 GN=Clpp PE=1 SV=1

25.06394005 24.39832687 23.72484 25.00749 21.42676 21.504 19.68197 23.70588 response t cell part;intracellular or REACT_346 2 2 2 11.8 11.8 11.8 43.001 0 5.449 27.30635 8 1 1 1 1 1 1 1 2 1.821288851 0.315196911 -2.968995094 -3.367405953 A0A3B2W4 A0A3B2W4 Chloride channel protein;H(+)/Cl(-) exchange trans **Clcn7** tr|A0A3B2W4I8|A0A3B2W4I8_MOUSE H(+)/Cl(-) exchange transporter 7 OS=Mus musculus OX=10090 GN=Clcn7 PE=1 SV=1;tr|E9PYL4|E9PYL4_MOUSE Chloride channel protein OS=Mus musculus OX=10090 GN=Clcn7 PE=1 SV=1;tr|F6SUM2|F6SUM2_MOUSE Chloride channel protein OS=Mus

23.51629066 23.81928825 24.01733 23.56326 23.96164 24.93333 24.54481 24.62014 anatomical cell body;c Acute myel REACT_275 5 5 5 9.4 9.4 9.4 67.581 0 5.5595 27.61119 11 3 3 2 2 4 3 4 3 1.816500879 0.317261538 0.785935879 3.358493311 P04627;Q8 P04627;Q8 Serine/threonine-protein kinase A-Raf **Araf** sp|P04627|ARAF_MOUSE Serine/threonine-protein kinase A-Raf OS=Mus musculus OX=10090 GN=Araf PE=1 SV=2;tr|Q8CAD1|Q8CAD1_MOUSE Non-specific serine/threonine protein kinase OS=Mus musculus OX=10090 GN=Araf PE=1 SV=1;tr|B1AUN8|B1AUN8_MOUSE Non-specific serine/

31.10717964 30.99738884 30.96334 30.86681 31.42547 31.32367 31.07508 31.30177 catabolic p cell part;ext Aminobenz REACT_279 18 18 18 71 71 71 31.474 0 164.55 34.46057 177 16 16 16 15 15 13 16 16 1.81004101 0.319785441 0.297818661 3.346483936 Q8BH95 Q8BH95 Enoyl-CoA hydratase, mitochondrial  **Echs1** sp|Q8BH95|ECHM_MOUSE Enoyl-CoA hydratase, mitochondrial OS=Mus musculus OX=10090 GN=Echs1 PE=1 SV=1

26.49185562 26.04963684 25.88807 26.16262 26.5123 26.54271 26.69392 26.86715 biological regulation;bi Purine met REACT_345 12 12 12 24.8 24.8 24.8 89.567 0 43.432 29.80466 45 4 3 5 3 5 11 9 8 1.807237724 0.320549618 0.505975246 3.341277916 A0A1L1SRX A0A1L1SRX AMP deaminase 3 **Ampd3** tr|A0A1L1SRX2|A0A1L1SRX2_MOUSE AMP deaminase OS=Mus musculus OX=10090 GN=Ampd3 PE=1 SV=1;sp|O08739|AMPD3_MOUSE AMP deaminase 3 OS=Mus musculus OX=10090 GN=Ampd3 PE=1 SV=2

27.28947258 27.44663811 27.31086 27.55105 28.11148 27.88379 27.76338 27.56107 amine meta extracellula Alanine, as REACT_315 17 17 17 52.8 52.8 52.8 51.739 0 82.323 30.91933 66 12 11 11 11 8 11 12 14 1.785433562 0.333536122 0.430422783 3.30089765 Q91YI0;E0C Q91YI0;E0C Argininosuccinate lyase **Asl** sp|Q91YI0|ARLY_MOUSE Argininosuccinate lyase OS=Mus musculus OX=10090 GN=Asl PE=1 SV=1;tr|E0CY49|E0CY49_MOUSE Argininosuccinate lyase (Fragment) OS=Mus musculus OX=10090 GN=Asl PE=1 SV=1

21.43781471 20.25296974 19.08348 22.53248 23.53528 23.68603 23.74632 22.65907 anatomical cell;cell par Adherens j REACT_294 1 1 1 3.6 3.6 3.6 47.575 0.006926 1.5872 26.1616 2 1 0 0 1 1 1 1 1 1.77465491 0.342045455 2.579993248 3.281008972 Q9D5H8;E Q9D5H8;E Receptor protein serine/threonine kinase;TGF-beta **Tgfbr1** tr|Q9D5H8|Q9D5H8_MOUSE Receptor protein serine/threonine kinase OS=Mus musculus OX=10090 GN=Tgfbr1 PE=1 SV=1;tr|E9Q418|E9Q418_MOUSE Receptor protein serine/threonine kinase OS=Mus musculus OX=10090 GN=Tgfbr1 PE=1 SV=1;sp|Q64729|TGFR1_MOUSE TGF-beta recepto

27.62198639 27.43952179 27.28842 27.74631 27.77693 28.15914 27.96681 27.88174 cellular comcell part;endosome;intracellular me 26 26 26 25.6 25.6 25.6 162.33 0 133.53 31.09005 116 11 15 13 12 15 19 20 18 1.767401911 0.343833962 0.422096729 3.267652604 Q8VDC1;A Q8VDC1;A FYVE and coiled-coil domain-containing protein 1 **Fyco1** sp|Q8VDC1|FYCO1_MOUSE FYVE and coiled-coil domain-containing protein 1 OS=Mus musculus OX=10090 GN=Fyco1 PE=1 SV=1;tr|A0A140T8V9|A0A140T8V9_MOUSE FYVE and coiled-coil domain-containing protein 1 (Fragment) OS=Mus musculus OX=10090 GN=Fyco1 PE=1 SV=3

26.18285179 25.64253998 25.95105 25.98311 25.4406 25.15731 25.41735 25.69928 cell part 5 5 5 46.5 46.5 46.5 25.222 0 23.029 28.91929 25 4 5 4 3 2 3 3 4 1.758927811 0.349428571 -0.511255741 -3.252074675 Q8BTZ5 Q8BTZ5 Ankyrin repeat domain-containing protein 46 **Ankrd46** sp|Q8BTZ5|ANR46_MOUSE Ankyrin repeat domain-containing protein 46 OS=Mus musculus OX=10090 GN=Ankrd46 PE=1 SV=1

28.81363106 28.9781189 29.50472 29.14156 28.53971 28.7081 28.61101 28.60703 biological r cell part;ma Spliceosom REACT_271 5 5 5 19.6 19.6 19.6 44.355 0 40.913 32.14105 42 4 4 4 3 4 4 5 4 1.756968036 0.350277154 -0.493043423 -3.248476156 Q8QZY9 Q8QZY9 Splicing factor 3B subunit 4 **Sf3b4** sp|Q8QZY9|SF3B4_MOUSE Splicing factor 3B subunit 4 OS=Mus musculus OX=10090 GN=Sf3b4 PE=1 SV=1

25.25634384 25.17192078 25.07451 25.44247 25.90412 25.57066 25.42822 25.70253 cell part;macromolecular complex;p 4 4 3 14 14 11.9 37.271 0 15.769 28.6567 16 4 4 4 3 3 3 3 3 1.756417299 0.350059701 0.415071964 3.247465177 E9PYD1;F6 E9PYD1;F6ZXW2;D3Z100;F6S0H0 **Fam98c** tr|E9PYD1|E9PYD1_MOUSE Family with sequence similarity 98, member C OS=Mus musculus OX=10090 GN=Fam98c PE=1 SV=1;tr|F6ZXW2|F6ZXW2_MOUSE Family with sequence similarity 98, member C (Fragment) OS=Mus musculus OX=10090 GN=Fam98c PE=1 SV=1;tr|D3Z100|D3Z100_MO

24.82434845 25.16817665 25.06011 25.05883 25.81836 25.3433 25.37971 25.34452 biological r cell part;cell projection;cytoplasm;c 8 8 8 9 9 9 135.62 0 29.003 28.55677 21 4 3 4 2 3 6 7 5 1.754497273 0.350111524 0.443606377 3.24394157 F8VQK5;P5 F8VQK5;P5 SAM and SH3 domain-containing protein 1 **Sash1** tr|F8VQK5|F8VQK5_MOUSE SAM and SH3 domain-containing protein 1 OS=Mus musculus OX=10090 GN=Sash1 PE=1 SV=1;sp|P59808|SASH1_MOUSE SAM and SH3 domain-containing protein 1 OS=Mus musculus OX=10090 GN=Sash1 PE=1 SV=1

21.77489281 23.45375061 20.6177 19.97877 24.15638 24.14227 23.64515 23.85053 biological r cell part;intracellular membrane-bo 4 4 4 10.5 10.5 10.5 71.035 0.000792 2.5099 26.67479 5 0 1 0 0 2 3 4 4 1.744990762 0.357896296 2.492305756 3.226517112 A0A1D5RMA0A1D5RMTranscription factor 25 **Tcf25** tr|A0A1D5RM90|A0A1D5RM90_MOUSE Transcription factor 25 OS=Mus musculus OX=10090 GN=Tcf25 PE=1 SV=1;tr|B2ZAC8|B2ZAC8_MOUSE Nuclear localized protein-1 isoform d (Fragment) OS=Mus musculus OX=10090 GN=Tcf25 PE=1 SV=1;sp|Q8R3L2|TCF25_MOUSE Transcription facto

20.77689362 21.80443001 23.34868 19.96504 23.65042 23.95189 24.12171 23.69019 autophagy; cell part;cytoplasm;endomembrane 7 7 7 3.8 3.8 3.8 394.29 0 22.686 26.83774 11 0 1 2 0 1 5 5 3 1.743929218 0.357653137 2.379793644 3.22457365 G3UYW1;A G3UYW1;A WD repeat and FYVE domain-containing protein 3 **Wdfy3** tr|G3UYW1|G3UYW1_MOUSE WD repeat and FYVE domain-containing protein 3 OS=Mus musculus OX=10090 GN=Wdfy3 PE=1 SV=1;tr|A0A1D5RLV7|A0A1D5RLV7_MOUSE WD repeat and FYVE domain-containing protein 3 OS=Mus musculus OX=10090 GN=Wdfy3 PE=1 SV=1;sp|Q6VNB8|WDFY3_MOUS

20.82894707 19.86487198 21.76876 20.70451 19.36444 19.19316 19.6554 19.69743 biological r cell part;cyt Nucleotide REACT_279 1 1 1 0.9 0.9 0.9 130.81 0.004093 1.9006 21.97865 1 0 0 0 0 1 1 1 1 1.742534714 0.357323529 -1.314166546 -3.222021288 E9QM61;P E9QM61;P DNA repair protein complementing XP-G cells hom **Ercc5** tr|E9QM61|E9QM61_MOUSE DNA repair protein-complementing XP-G cells homolog OS=Mus musculus OX=10090 GN=Ercc5 PE=1 SV=1;sp|P35689|ERCC5_MOUSE DNA repair protein complementing XP-G cells homolog OS=Mus musculus OX=10090 GN=Ercc5 PE=1 SV=4

23.58794975 24.67567635 25.08974 24.18101 20.28497 19.83577 20.76608 23.94235 cell part;cytoplasm;Golgi apparatus 2 2 2 2.6 2.6 2.6 111.71 0.003196 1.9512 27.02707 7 2 1 1 1 1 0 1 1 1.742299157 0.356483516 -3.176304817 -3.221590226 Q8BWZ3;G Q8BWZ3;G N-alpha-acetyltransferase 25, NatB auxiliary subun **Naa25** sp|Q8BWZ3|NAA25_MOUSE N-alpha-acetyltransferase 25, NatB auxiliary subunit OS=Mus musculus OX=10090 GN=Naa25 PE=1 SV=1;tr|G3UZ51|G3UZ51_MOUSE N-alpha-acetyltransferase 25, NatB auxiliary subunit (Fragment) OS=Mus musculus OX=10090 GN=Naa25 PE=1 SV=1

30.42071342 30.41810226 30.58916 30.71645 30.29143 30.3434 30.16087 30.2922 biosynthet cell part;cytoplasm;mac REACT_347 9 9 9 61.8 61.8 61.8 24.693 0 143.15 33.65811 132 9 9 8 8 9 9 9 9 1.740706398 0.356510949 -0.2641325 -3.218676092 O70251;A0 O70251;A0 Elongation factor 1-beta **Eef1b;Eef1b2** sp|O70251|EF1B_MOUSE Elongation factor 1-beta OS=Mus musculus OX=10090 GN=Eef1b PE=1 SV=5;tr|A0A087WS46|A0A087WS46_MOUSE Eukaryotic translation elongation factor 1 beta 2 OS=Mus musculus OX=10090 GN=Eef1b2 PE=1 SV=1

19.35315132 20.60333443 22.98815 19.85159 23.06955 23.26699 23.32773 23.5285 biosynthet cell part;intracellular m REACT_292 4 4 4 21.4 21.4 21.4 20.68 0.002311 2.1076 26.31902 4 1 1 1 1 1 2 2 2 1.733890063 0.360683636 2.599137783 3.206216179 Q9CQF0;A0 Q9CQF0;A0 39S ribosomal protein L11, mitochondrial  **Mrpl11** sp|Q9CQF0|RM11_MOUSE 39S ribosomal protein L11, mitochondrial OS=Mus musculus OX=10090 GN=Mrpl11 PE=1 SV=1;tr|A0A494B9H5|A0A494B9H5_MOUSE 39S ribosomal protein L11, mitochondrial (Fragment) OS=Mus musculus OX=10090 GN=Mrpl11 PE=1 SV=1;tr|A0A494B9R3|A0A494B

28.2185421 28.18588829 28.11202 27.88215 28.22871 28.73767 28.50945 28.65564 amine meta cell part;cyt One carbon REACT_344 27 27 27 46.1 46.1 46.1 107.5 0 161.12 31.65864 118 21 18 16 17 16 21 21 19 1.733798716 0.359521739 0.433215618 3.206049325 Q64737;D Q64737 Trifunctional purine biosynthetic protein adenosin **Gart** sp|Q64737|PUR2_MOUSE Trifunctional purine biosynthetic protein adenosine-3 OS=Mus musculus OX=10090 GN=Gart PE=1 SV=3

28.69355202 28.79420662 28.89371 29.08715 29.67857 29.16684 29.12952 29.47798 biological r cell part;intracellular membrane-bo 12 11 11 54 48.1 48.1 35.649 0 37.092 32.36403 74 10 10 10 9 8 8 9 9 1.724525148 0.365776173 0.4960742 3.189127402 Q99JR1 Q99JR1 Sideroflexin-1 **Sfxn1** sp|Q99JR1|SFXN1_MOUSE Sideroflexin-1 OS=Mus musculus OX=10090 GN=Sfxn1 PE=1 SV=3

23.81597137 23.67195702 22.56246 24.78696 22.44133 18.1436 20.64227 20.89824 biological r cell part;ma Calcium sig REACT_327 41 2 2 39.2 1.9 1.9 128.56 0.003493 1.9247 25.6949 3 1 1 1 1 1 0 1 0 1.719450805 0.369482014 -3.177976131 -3.179882228 F7AAP4;E9 F7AAP4;E9 Calcium-transporting ATPase **Atp2b4** tr|F7AAP4|F7AAP4_MOUSE Calcium-transporting ATPase OS=Mus musculus OX=10090 GN=Atp2b4 PE=1 SV=1;tr|E9Q828|E9Q828_MOUSE Calcium-transporting ATPase OS=Mus musculus OX=10090 GN=Atp2b4 PE=1 SV=1

20.73595428 18.79472733 21.31014 21.65467 22.79931 22.88972 22.5956 22.41684 biological r cell part;cytoplasm;intr REACT_346 3 3 2 40.7 40.7 31 12.562 0.00424 1.8812 26.55497 2 1 1 1 2 1 1 1 1 1.713919145 0.37311828 2.051493168 3.169815233 B1AVF2;Q9 B1AVF2;Q9 TSC22 domain family protein 3 **Tsc22d3** tr|B1AVF2|B1AVF2_MOUSE TSC22 domain family protein 3 OS=Mus musculus OX=10090 GN=Tsc22d3 PE=1 SV=1;sp|Q9Z2S7|T22D3_MOUSE TSC22 domain family protein 3 OS=Mus musculus OX=10090 GN=Tsc22d3 PE=1 SV=2

26.58161545 26.97738266 26.32074 26.86089 27.47769 27.27719 27.22426 26.99938 catabolic p cell part;cyt Alzheimer's REACT_275 15 15 15 28 28 28 80.362 0 25.907 30.30965 40 6 5 5 4 10 12 10 9 1.709489186 0.376957143 0.559475422 3.16176173 A0A494BB A0A494BB Calpain-1 catalytic subunit **Capn1** tr|A0A494BBB0|A0A494BBB0_MOUSE Calcium-activated neutral proteinase 1 (Fragment) OS=Mus musculus OX=10090 GN=Capn1 PE=1 SV=1;sp|O35350|CAN1_MOUSE Calpain-1 catalytic subunit OS=Mus musculus OX=10090 GN=Capn1 PE=1 SV=1

23.31520462 23.16154099 23.2798 23.56209 23.70567 23.73975 23.9208 23.49057 establishm cell part;membrane;plasma membra 5 5 4 8.3 8.3 7.2 76.278 0 4.0627 26.92875 10 1 1 1 2 2 3 4 4 1.705089339 0.378533808 0.384537697 3.153770434 Q32NY4 Q32NY4 Metal transporter CNNM3 **Cnnm3** sp|Q32NY4|CNNM3_MOUSE Metal transporter CNNM3 OS=Mus musculus OX=10090 GN=Cnnm3 PE=1 SV=2

22.89644814 23.38903236 23.66893 23.18376 23.61628 24.18495 23.85386 24.25162 biological r cell part;cytoplasm;intracellular me 6 3 0 59.4 34.6 0 14.659 0 28.638 27.14073 13 2 2 1 1 2 2 3 3 1.696986506 0.383106383 0.692135334 3.139072882 A0A0N4SU A0A0N4SUX1;Q8CII5;G5E8L2;Q80ZW7;A0A0N4SVR8 **Tia1** tr|A0A0N4SUX1|A0A0N4SUX1_MOUSE Nucleolysin TIA-1 OS=Mus musculus OX=10090 GN=Tia1 PE=1 SV=1;tr|Q8CII5|Q8CII5_MOUSE Nucleolysin TIA-1 OS=Mus musculus OX=10090 GN=Tia1 PE=1 SV=1;tr|G5E8L2|G5E8L2_MOUSE Nucleolysin TIA-1 OS=Mus musculus OX=10090 GN=Tia1 PE=1 S

23.95898819 22.20871925 22.17703 22.12909 21.24652 19.40115 21.36674 20.51578 cell activati cell part;int Non-homologous end- 1 1 1 8.8 8.8 8.8 32.739 0 15.719 24.85576 1 1 1 1 1 0 0 0 0 1.689384544 0.38780212 -1.985909462 -3.125306499 Q3KNJ2 Q3KNJ2 Non-homologous end-joining factor 1 **Nhej1** sp|Q3KNJ2|NHEJ1_MOUSE Non-homologous end-joining factor 1 OS=Mus musculus OX=10090 GN=Nhej1 PE=1 SV=1

28.02991676 28.24222755 27.8527 28.10689 28.35699 28.29605 28.27298 28.35405 biological r macromolecular complex;protein c 16 16 15 53.2 53.2 49.6 42.467 0 88.237 31.43674 80 12 11 10 10 13 11 13 12 1.687408162 0.387647887 0.262085915 3.12173104 B1AT82;Q9 B1AT82;Q9 Phosphoribosyl pyrophosphate synthase-associa **Prpsap1** tr|B1AT82|B1AT82_MOUSE Phosphoribosyl pyrophosphate synthase-associated protein 1 OS=Mus musculus OX=10090 GN=Prpsap1 PE=1 SV=1;sp|Q9D0M1|KPRA_MOUSE Phosphoribosyl pyrophosphate synthase-associated protein 1 OS=Mus musculus OX=10090 GN=Prpsap1 PE=1 SV=1

25.96547699 26.28772926 26.25979 26.13809 26.52634 26.57946 26.41171 26.31164 lipid metab cell part;me Steroid hormone biosy 7 7 7 17 17 17 66.59 0 17.711 29.6406 39 6 6 7 4 5 6 7 7 1.681451319 0.390498246 0.294512272 3.110963423 P50427 P50427 Steryl-sulfatase **Sts** sp|P50427|STS_MOUSE Steryl-sulfatase OS=Mus musculus OX=10090 GN=Sts PE=1 SV=1

26.59875107 26.53507614 26.63727 26.69205 26.19385 26.38359 26.54743 26.17722 11 11 11 34 34 34 54.044 0 24.234 29.80286 44 6 7 7 3 7 7 8 5 1.67686696 0.392671329 -0.290267944 -3.102685737 Q8VE88 Q8VE88 Protein FAM114A2 **Fam114a2** sp|Q8VE88|F1142_MOUSE Protein FAM114A2 OS=Mus musculus OX=10090 GN=Fam114a2 PE=1 SV=2

21.54472351 21.85285187 21.94913 21.91838 20.92956 19.7763 19.33405 21.31646 cellular comcell body;cell part;cell projection;cy 6 3 3 6.2 2.8 2.8 190.97 0.002321 2.155 23.80607 3 2 1 1 1 0 0 0 1 1.67249875 0.394522648 -1.477180004 -3.0948056 E9QAN4;G E9QAN4;G Kinesin-like protein;Kinesin-like protein KIF1A **Kif1a** tr|E9QAN4|E9QAN4_MOUSE Kinesin-like protein KIF1A OS=Mus musculus OX=10090 GN=Kif1a PE=1 SV=1;tr|G3UW47|G3UW47_MOUSE Kinesin-like protein KIF1A OS=Mus musculus OX=10090 GN=Kif1a PE=1 SV=1;tr|Q6TA13|Q6TA13_MOUSE Kinesin-like protein KIF1A OS=Mus musculus OX

21.65119171 21.61105728 24.27455 20.27668 24.08562 25.05957 25.18465 24.29936 biological r cell part;intracellular membrane-bo 4 4 4 10.1 10.1 10.1 63.538 0 4.4119 27.63582 8 0 0 1 0 2 4 3 3 1.659302716 0.406472222 2.703929901 3.071043009 Q9JHW4;A Q9JHW4;A Selenocysteine-specific elongation factor **Eefsec** sp|Q9JHW4|SELB_MOUSE Selenocysteine-specific elongation factor OS=Mus musculus OX=10090 GN=Eefsec PE=1 SV=2;tr|A0A0N4SUV6|A0A0N4SUV6_MOUSE Selenocysteine-specific elongation factor OS=Mus musculus OX=10090 GN=Eefsec PE=1 SV=1;tr|A0A0N4SVA4|A0A0N4SVA4_MOUSE

25.12813568 25.29203796 25.1102 25.53917 26.17575 25.90104 25.39398 25.97821 biological r cell part;cell projection; REACT_272 14 14 14 18 18 18 120.23 0 30.748 28.88951 41 8 6 5 7 10 11 10 12 1.657702259 0.406768166 0.594860554 3.068165338 B1AXI9;J3QB1AXI9;J3QCentrosomal protein of 131 kDa **Cep131** tr|B1AXI9|B1AXI9_MOUSE 5-azacytidine induced gene 1 OS=Mus musculus OX=10090 GN=Cep131 PE=1 SV=1;tr|J3QMP9|J3QMP9_MOUSE Centrosomal protein of 131 kDa OS=Mus musculus OX=10090 GN=Cep131 PE=1 SV=1;sp|Q62036|CP131_MOUSE Centrosomal protein of 131 kDa OS=Mus

24.62611389 22.13693047 23.66709 22.10604 20.0628 20.00161 22.25751 20.10626 anatomical apical part Vasopressi REACT_316 5 5 5 30.2 30.2 30.2 39.469 0 6.3035 28.36093 5 3 2 2 2 3 1 2 3 1.651562668 0.413089655 -2.526998043 -3.0571348 Q8K0T2 Q8K0T2 Cytoplasmic dynein 2 light intermediate chain 1 **Dync2li1** sp|Q8K0T2|DC2L1_MOUSE Cytoplasmic dynein 2 light intermediate chain 1 OS=Mus musculus OX=10090 GN=Dync2li1 PE=1 SV=1

24.35339737 23.72993088 23.82114 24.16323 24.55374 24.52576 25.56491 24.87526 anatomical cell part;endosome;intracellular me 8 8 8 6.9 6.9 6.9 180.2 0 36.845 27.9989 29 4 2 2 1 3 3 6 6 1.651435635 0.411670103 0.862991333 3.056906714 Q69Z37;E9 Q69Z37;E9 Sterile alpha motif domain-containing protein 9-lik **Samd9l** sp|Q69Z37|SAM9L_MOUSE Sterile alpha motif domain-containing protein 9-like OS=Mus musculus OX=10090 GN=Samd9l PE=1 SV=2;tr|E9PX59|E9PX59_MOUSE Sterile alpha motif domain-containing protein 9-like OS=Mus musculus OX=10090 GN=Samd9l PE=1 SV=1

26.77504921 26.85448074 26.69883 27.04562 27.47507 27.08598 27.10629 27.14734 biological r cell part;cel Chemokine REACT_272 7 7 7 34.4 34.4 34.4 43.565 0 78.073 30.25878 52 6 5 4 4 5 5 7 5 1.650610045 0.411273973 0.360176563 3.055424517 P62881 P62881 Guanine nucleotide-binding protein subunit beta- **Gnb5** sp|P62881|GNB5_MOUSE Guanine nucleotide-binding protein subunit beta-5 OS=Mus musculus OX=10090 GN=Gnb5 PE=1 SV=1

21.67968559 21.9214344 22.41929 22.4139 20.97756 19.2652 21.51091 20.39078 anatomical cell body;cell part;cell projection;cy 1 1 1 3.5 3.5 3.5 36.28 0.006504 1.6287 24.33518 2 1 1 1 1 0 0 0 0 1.647446861 0.412081911 -1.572464943 -3.049747874 A0A2R8VH A0A2R8VH Zinc finger protein 385A **Znf385a** tr|A0A2R8VHX5|A0A2R8VHX5_MOUSE Zinc finger protein 385A OS=Mus musculus OX=10090 GN=Zfp385a PE=1 SV=1;sp|Q8VD12|Z385A_MOUSE Zinc finger protein 385A OS=Mus musculus OX=10090 GN=Znf385a PE=1 SV=2

25.542593 25.75465584 25.3947 25.57228 25.94606 26.03576 25.86802 25.68752 biological r cell part;cytoplasm;intracellular me 6 6 6 30.5 30.5 30.5 30.232 0 28.472 29.01289 28 5 4 4 4 5 5 6 5 1.643817475 0.414068027 0.318283081 3.043239017 A0A1B0GR A0A1B0GR MOB kinase activator 2 **Mob2** tr|A0A1B0GR18|A0A1B0GR18_MOUSE MOB kinase activator 2 OS=Mus musculus OX=10090 GN=Mob2 PE=1 SV=1;tr|A0A1B0GRX6|A0A1B0GRX6_MOUSE MOB kinase activator 2 (Fragment) OS=Mus musculus OX=10090 GN=Mob2 PE=1 SV=1;sp|Q8VI63|MOB2_MOUSE MOB kinase activator 2 OS=Mus

22.31282425 22.29443169 22.97215 23.44088 20.73953 19.39068 22.3192 19.2754 biological r cell part;cyt Axon guida REACT_295 3 3 3 6.8 6.8 6.8 67.285 0 3.1333 25.90366 4 2 1 3 1 0 1 1 1 1.64308251 0.413098305 -2.323868752 -3.041921522 E9Q0W6;E E9Q0W6;E Actin-binding LIM protein 2 **Ablim2** tr|E9Q0W6|E9Q0W6_MOUSE Actin-binding LIM protein 2 OS=Mus musculus OX=10090 GN=Ablim2 PE=1 SV=1;tr|E9Q4K0|E9Q4K0_MOUSE Actin-binding LIM protein 2 OS=Mus musculus OX=10090 GN=Ablim2 PE=1 SV=1;sp|Q8BL65|ABLM2_MOUSE Actin-binding LIM protein 2 OS=Mus musculu

19.62844086 20.95433044 21.29149 18.50654 21.76276 23.48407 22.43886 21.8418 behavior;bi cell part;membrane REACT_301 2 1 1 24.4 13.7 13.7 18.513 0.004231 1.87 25.27142 1 0 0 0 0 1 1 1 1 1.640210727 0.413256757 2.286669731 3.036775431 F6RT95;B1 F6RT95;B1AXF2;Q8BUN9 **Slc24a2** tr|F6RT95|F6RT95_MOUSE Solute carrier family 24 (sodium/potassium/calcium exchanger), member 2 (Fragment) OS=Mus musculus OX=10090 GN=Slc24a2 PE=1 SV=8;tr|B1AXF2|B1AXF2_MOUSE Solute carrier family 24 (sodium/potassium/calcium exchanger), member 2 OS=Mus mu

24.78301811 24.67066002 24.89865 24.68694 20.13773 20.04244 24.6692 19.30807 biological r cell part;endoplasmic reticulum;intr 3 3 3 7 7 7 72.799 0.004239 1.8805 27.20023 6 2 3 3 1 0 0 1 1 1.640138085 0.41186532 -3.72045517 -3.036645298 Q8BKS9;A0 Q8BKS9;A0 Pumilio domain-containing protein KIAA0020 **Kiaa0020** sp|Q8BKS9|PUM3_MOUSE Pumilio homolog 3 OS=Mus musculus OX=10090 GN=Pum3 PE=1 SV=2;tr|A0A0N4SUH4|A0A0N4SUH4_MOUSE Pumilio homolog 3 OS=Mus musculus OX=10090 GN=Pum3 PE=1 SV=1

38.27950287 37.68146515 38.13542 38.52697 37.60967 37.24458 37.5433 37.73132 cell part;macromolecul REACT_280 27 27 9 97.3 97.3 38.1 15.748 0 323.31 41.10583 1878 27 26 27 26 24 23 25 25 1.638670025 0.41138255 -0.623621941 -3.034015784 A8DUK4;E9 A8DUK4;E9Q223 **Hbbt1;Hbb-bs** tr|A8DUK4|A8DUK4_MOUSE Beta-globin OS=Mus musculus OX=10090 GN=Hbb-bs PE=1 SV=1;tr|E9Q223|E9Q223_MOUSE Hemoglobin, beta adult s chain (Fragment) OS=Mus musculus OX=10090 GN=Hbb-bs PE=1 SV=1

24.78735924 24.48953056 24.81948 24.47871 24.0573 24.34171 24.34232 23.71963 cell death;c cell part;cytoplasm;endoplasmic ret 7 7 7 11.7 11.7 11.7 71.302 0 13 27.67566 16 4 5 5 3 2 4 4 4 1.63614652 0.412521739 -0.528532505 -3.029497603 D3Z6S1;Q8 D3Z6S1;Q8 Transmembrane protein 214 **Tmem214** tr|D3Z6S1|D3Z6S1_MOUSE Transmembrane protein 214 OS=Mus musculus OX=10090 GN=Tmem214 PE=1 SV=3;sp|Q8BM55|TM214_MOUSE Transmembrane protein 214 OS=Mus musculus OX=10090 GN=Tmem214 PE=1 SV=1

22.80088806 22.43883133 22.57071 22.74273 22.90277 22.80126 23.13591 23.13242 cellular comcell part;centrosome;in REACT_275 5 2 2 4.5 2.4 2.4 195.7 0 3.6084 26.06363 9 2 2 1 1 1 1 1 1 1.626601404 0.42196 0.354801178 3.012428058 F8VQ75;Q9 F8VQ75;Q9 Kinesin-like protein;Kinesin-like protein KIF13A **Kif13a** tr|F8VQ75|F8VQ75_MOUSE Kinesin-like protein KIF13A OS=Mus musculus OX=10090 GN=Kif13a PE=1 SV=1;sp|Q9EQW7|KI13A_MOUSE Kinesin-like protein KIF13A OS=Mus musculus OX=10090 GN=Kif13a PE=1 SV=1

21.12922668 22.08300018 19.60153 19.73146 22.70881 22.30042 22.28825 22.47912 cellular component organization;cellular memb 3 3 3 7.4 7.4 7.4 70.083 0 4.0557 25.15221 3 1 0 0 0 2 1 1 1 1.62275533 0.423774086 1.807843208 3.005559183 D3YWC9;Q D3YWC9;Q MTSS1-like protein **Mtss1l** tr|D3YWC9|D3YWC9_MOUSE Protein MTSS 2 OS=Mus musculus OX=10090 GN=Mtss2 PE=1 SV=1;sp|Q6P9S0|MTSS2_MOUSE Protein MTSS 2 OS=Mus musculus OX=10090 GN=Mtss2 PE=1 SV=1;tr|F7D291|F7D291_MOUSE Protein MTSS 2 (Fragment) OS=Mus musculus OX=10090 GN=Mtss2 PE=1 SV=1

21.75135994 22.79447556 22.45413 22.81029 19.99566 20.48455 22.23701 20.47223 2 2 2 6.8 6.8 6.8 40.433 0.004095 1.9109 24.4128 2 1 1 1 1 0 1 0 0 1.619057624 0.427311258 -1.655198574 -2.998960162 Q91W67;A Q91W67;A Ubiquitin-like protein 7 **Ubl7** sp|Q91W67|UBL7_MOUSE Ubiquitin-like protein 7 OS=Mus musculus OX=10090 GN=Ubl7 PE=1 SV=2;tr|A0A1L1SRW2|A0A1L1SRW2_MOUSE Ubiquitin-like protein 7 (Fragment) OS=Mus musculus OX=10090 GN=Ubl7 PE=1 SV=1;tr|A0A1L1STF2|A0A1L1STF2_MOUSE Ubiquitin-like protein 7 (

20.48976517 23.70851517 21.8035 18.83183 24.75466 25.19577 24.00496 23.83316 cell part;cytoplasm 9 1 1 52.5 6 6 21.706 0 3.6712 27.28472 5 1 1 0 0 1 1 1 1 1.618987274 0.42590099 3.238733768 2.99883466 Q8BKP1;Q Q8BKP1;Q3TUJ9;F6VQ81 **Tpd52l2** tr|Q8BKP1|Q8BKP1_MOUSE Tumor protein D54 OS=Mus musculus OX=10090 GN=Tpd52l2 PE=1 SV=1;tr|Q3TUJ9|Q3TUJ9_MOUSE Tumor protein D54 OS=Mus musculus OX=10090 GN=Tpd52l2 PE=1 SV=1;tr|F6VQ81|F6VQ81_MOUSE Tumor protein D54 (Fragment) OS=Mus musculus OX=10090 GN=Tp

25.7204628 25.9591217 25.74258 25.92159 25.923 26.13825 26.22131 26.10821 6 6 6 36.7 36.7 36.7 24.33 0 14.162 29.31786 28 5 4 5 4 4 5 5 5 1.61266211 0.430697368 0.261754513 2.987557788 Q8VE95;Q9 Q8VE95 UPF0598 protein C8orf82 homolog sp|Q8VE95|CH082_MOUSE UPF0598 protein C8orf82 homolog OS=Mus musculus OX=10090 PE=1 SV=1

19.58298111 20.7926712 22.58097 19.10726 22.58987 22.79687 22.70428 23.8399 biosynthet cell part;cyt mRNA surv REACT_291 4 4 4 32.1 32.1 32.1 18.934 0.000637 2.5872 26.45513 4 2 2 2 0 1 1 2 1 1.611030163 0.430937705 2.466760635 2.984650506 E9QAY4;A0 E9QAY4;A0 Poly(A) polymerase alpha **Papola** tr|E9QAY4|E9QAY4_MOUSE Polynucleotide adenylyltransferase OS=Mus musculus OX=10090 GN=Papola PE=1 SV=1;tr|A0A0R4J244|A0A0R4J244_MOUSE Polynucleotide adenylyltransferase OS=Mus musculus OX=10090 GN=Papola PE=1 SV=1;tr|E9PWC8|E9PWC8_MOUSE Poly(A) polymerase

22.72673225 20.86763191 23.36706 22.22858 19.70801 20.34104 19.87539 21.44197 extracellular organelle;organelle;ves 1 1 1 3.2 3.2 3.2 91.921 0 4.617 24.87591 2 1 1 1 1 0 0 0 1 1.602351232 0.438287582 -1.9559021 -2.969204544 Q9D2I5 Q9D2I5 LisH domain-containing protein ARMC9 **Armc9** sp|Q9D2I5|ARMC9_MOUSE LisH domain-containing protein ARMC9 OS=Mus musculus OX=10090 GN=Armc9 PE=1 SV=1

23.21689606 23.01792717 22.68945 23.08049 22.37359 20.91469 21.60291 19.34328 biological r cell part;cell projection; REACT_289 15 1 1 21.1 1.4 1.4 123.69 0.004496 1.7809 24.92007 1 1 1 1 1 0 0 0 0 1.601950904 0.437798046 -1.942567825 -2.9684927 E9PY13;E9 E9PY13;E9 Guanine nucleotide exchange factor DBS **Mcf2l** tr|E9PY13|E9PY13_MOUSE Guanine nucleotide exchange factor DBS OS=Mus musculus OX=10090 GN=Mcf2l PE=1 SV=1;tr|E9PY12|E9PY12_MOUSE Guanine nucleotide exchange factor DBS OS=Mus musculus OX=10090 GN=Mcf2l PE=1 SV=1;tr|G3UX72|G3UX72_MOUSE Guanine nucleotide ex

19.66499519 21.44098854 18.55232 24.16791 25.09366 24.53629 24.88362 24.07594 biosynthet cell part;int Pyruvate m REACT_292 3 3 3 12 12 12 51.847 0 7.3456 27.10089 5 0 0 0 1 3 2 2 2 1.600187098 0.439298701 3.690825462 2.965357034 Q7TNG8 Q7TNG8 Probable D-lactate dehydrogenase, mitochondrial **Ldhd** sp|Q7TNG8|LDHD_MOUSE Probable D-lactate dehydrogenase, mitochondrial OS=Mus musculus OX=10090 GN=Ldhd PE=1 SV=1

20.75008965 20.15924835 21.34692 19.73868 19.3483 19.27232 19.76268 19.20641 biological r cell part;cytoplasm;intracellular me 3 3 3 3.6 3.6 3.6 130.66 0.007479 1.5377 23.18057 3 0 0 1 0 1 2 1 1 1.59599951 0.441294498 -1.101304531 -2.957916643 E9QKD1;Q E9QKD1;Q Nucleolar protein 8 **Nol8** tr|E9QKD1|E9QKD1_MOUSE Nucleolar protein 8 OS=Mus musculus OX=10090 GN=Nol8 PE=1 SV=1;sp|Q3UHX0|NOL8_MOUSE Nucleolar protein 8 OS=Mus musculus OX=10090 GN=Nol8 PE=1 SV=2

29.95240974 29.93999672 29.8935 30.09119 30.12325 30.24252 30.03983 30.23866 alcohol me cell part;cyt Inositol ph REACT_293 16 16 16 61 61 61 30.429 0 108.27 33.27017 150 15 15 15 15 13 16 15 15 1.59467933 0.440954839 0.191790581 2.955572216 Q924B0;O Q924B0;O Inositol monophosphatase 1 **Impa1** tr|Q924B0|Q924B0_MOUSE Inositol-1-monophosphatase OS=Mus musculus OX=10090 GN=Impa1 PE=1 SV=1;sp|O55023|IMPA1_MOUSE Inositol monophosphatase 1 OS=Mus musculus OX=10090 GN=Impa1 PE=1 SV=1;tr|Q80ZJ2|Q80ZJ2_MOUSE Inositol-1-monophosphatase OS=Mus musculus OX=

31.73839951 31.74346542 31.71338 31.71514 31.72777 31.97333 31.94465 31.98142 cell recogni cell body;cell part;centr REACT_272 35 35 35 81.3 81.3 81.3 59.623 0 310.47 35.14843 350 30 30 30 32 29 32 32 31 1.592934833 0.440578778 0.179194927 2.952475176 P80316;E0 P80316 T-complex protein 1 subunit epsilon **Cct5** sp|P80316|TCPE_MOUSE T-complex protein 1 subunit epsilon OS=Mus musculus OX=10090 GN=Cct5 PE=1 SV=1

27.09637451 26.79352379 26.75875 26.57888 27.01808 27.31034 27.21364 27.60406 cell part;extracellular organelle;intra 7 7 7 33.7 33.7 33.7 32.032 0 23.92 30.33405 37 5 5 4 5 6 6 6 6 1.591994764 0.439730769 0.479646683 2.950806676 Q91V64;A0 Q91V64;A0 Isochorismatase domain-containing protein 1 **Isoc1** sp|Q91V64|ISOC1_MOUSE Isochorismatase domain-containing protein 1 OS=Mus musculus OX=10090 GN=Isoc1 PE=1 SV=1;tr|A0A494B952|A0A494B952_MOUSE Isochorismatase domain-containing protein 1 OS=Mus musculus OX=10090 GN=Isoc1 PE=1 SV=1

25.09992981 24.62555695 24.94659 24.78756 25.35121 25.17303 25.05953 25.32347 7 7 7 30.3 30.3 30.3 43.126 0 22.433 28.31363 27 4 6 5 5 2 3 3 3 1.590284123 0.439884984 0.361900806 2.947771279 Q8K0G5;A Q8K0G5;A Protein TSSC1 **Tssc1** sp|Q8K0G5|EIPR1_MOUSE EARP and GARP complex-interacting protein 1 OS=Mus musculus OX=10090 GN=Eipr1 PE=1 SV=2;tr|A0A1Y7VN36|A0A1Y7VN36_MOUSE EARP and GARP complex-interacting protein 1 (Fragment) OS=Mus musculus OX=10090 GN=Eipr1 PE=1 SV=1

27.48277664 27.58183098 27.24943 27.51518 27.07826 27.33952 27.17655 27.17055 anatomical cell part;Golgi apparatu REACT_361 32 32 32 25.7 25.7 25.7 226.45 0 102.5 30.59272 88 13 14 17 16 17 22 22 18 1.587474831 0.441312102 -0.266086102 -2.942788547 E9Q512;H3 E9Q512;H3BJG4 **Trip11** tr|E9Q512|E9Q512_MOUSE Thyroid hormone receptor interactor 11 OS=Mus musculus OX=10090 GN=Trip11 PE=1 SV=1;tr|H3BJG4|H3BJG4_MOUSE Thyroid hormone receptor interactor 11 (Fragment) OS=Mus musculus OX=10090 GN=Trip11 PE=1 SV=1

26.24178123 26.1018734 25.89885 25.99688 26.44993 26.33441 26.39997 26.17307 anatomical cell part;cytoplasm;cyto REACT_319 6 6 6 15.6 15.6 15.6 65.853 0 22.419 29.44908 26 5 4 4 5 3 5 5 5 1.58685751 0.440165079 0.279497147 2.941693984 Q9WVG6;D Q9WVG6;D Histone-arginine methyltransferase CARM1 **Carm1** sp|Q9WVG6|CARM1_MOUSE Histone-arginine methyltransferase CARM1 OS=Mus musculus OX=10090 GN=Carm1 PE=1 SV=2;tr|D3YUP1|D3YUP1_MOUSE Coactivator-associated arginine methyltransferase 1 OS=Mus musculus OX=10090 GN=Carm1 PE=1 SV=1

21.29513168 19.79761696 20.66761 18.41202 21.51167 22.15001 22.30031 21.83434 anatomical cell part;histone methy REACT_332 4 4 4 3.9 3.9 3.9 219.96 0.00064 2.6612 26.38652 10 1 2 0 3 1 1 1 1 1.585394013 0.440227848 1.905984879 2.939099587 Q5XJV5;A2 Q5XJV5;A2 Nuclear receptor coactivator 6 **Ncoa6** tr|Q5XJV5|Q5XJV5_MOUSE Nuclear receptor coactivator 6 OS=Mus musculus OX=10090 GN=Ncoa6 PE=1 SV=1;tr|A2AQM9|A2AQM9_MOUSE Nuclear receptor coactivator 6 (Fragment) OS=Mus musculus OX=10090 GN=Ncoa6 PE=1 SV=8;sp|Q9JL19|NCOA6_MOUSE Nuclear receptor coactivato

21.64829636 21.13288879 22.10287 21.85003 22.6446 22.87923 22.53129 21.97179 anatomical cell part;cytoplasm;extr REACT_278 2 2 2 4.3 4.3 4.3 74.609 0 4.0711 25.60685 13 1 1 1 1 1 2 2 2 1.575654253 0.44859306 0.823209286 2.921851719 Q61592 Q61592 Growth arrest-specific protein 6 **Gas6** sp|Q61592|GAS6_MOUSE Growth arrest-specific protein 6 OS=Mus musculus OX=10090 GN=Gas6 PE=2 SV=2

30.64108276 30.69701958 31.00243 30.73295 29.93255 30.32967 30.58118 30.3872 biological r cell part;cyt Collecting d REACT_282 27 27 27 45.1 45.1 45.1 103.13 0 151.81 33.82781 198 20 24 22 17 19 23 23 22 1.572050294 0.450515723 -0.460717678 -2.915477531 P04919 P04919 Band 3 anion transport protein **Slc4a1** sp|P04919|B3AT_MOUSE Band 3 anion transport protein OS=Mus musculus OX=10090 GN=Slc4a1 PE=1 SV=1

24.93418121 24.43179703 24.85861 24.88962 25.5263 25.27199 25.0466 25.11164 anatomical cell part;intracellular m REACT_285 5 3 3 18.6 13.4 13.4 54.161 0 11.695 28.33803 13 2 1 1 3 1 2 2 1 1.570425375 0.45015674 0.460577965 2.912605013 E9PUH7;Q E9PUH7;Q Nuclear factor 1;Nuclear factor 1 X-type **Nfix** tr|E9PUH7|E9PUH7_MOUSE Nuclear factor 1 OS=Mus musculus OX=10090 GN=Nfix PE=1 SV=1;tr|Q3TYK3|Q3TYK3_MOUSE Nuclear factor 1 OS=Mus musculus OX=10090 GN=Nfix PE=1 SV=1;tr|D3YZ00|D3YZ00_MOUSE Nuclear factor 1 (Fragment) OS=Mus musculus OX=10090 GN=Nfix PE=1 S

23.37901497 24.22044373 24.16285 23.8399 23.34733 22.71697 22.9749 23.50397 biological regulation;ca Protein processing in e 1 1 1 4.4 4.4 4.4 37.485 0 4.2758 26.7618 8 1 1 1 1 1 1 1 1 1.569328627 0.44985 -0.764759541 -2.910666668 Q8CB27 Q8CB27 Ubiquitin thioesterase OTU1 **Yod1** sp|Q8CB27|OTU1_MOUSE Ubiquitin thioesterase OTU1 OS=Mus musculus OX=10090 GN=Yod1 PE=1 SV=1

22.25853729 23.91861725 20.67608 24.06751 26.09794 25.1736 24.32855 25.55286 digestion;m extracellular organelle;e REACT_319 1 1 1 8.1 8.1 8.1 26.274 0.004363 1.8157 28.48984 3 1 1 0 1 1 1 1 1 1.566333648 0.450990654 2.558049202 2.90537548 Q9R0T7;Q9 Q9R0T7;Q9QUK9 **Try4;Try5** tr|Q9R0T7|Q9R0T7_MOUSE Pancreatic trypsin OS=Mus musculus OX=10090 GN=Try4 PE=1 SV=1;tr|Q9QUK9|Q9QUK9_MOUSE TESP4 OS=Mus musculus OX=10090 GN=Try5 PE=1 SV=1

27.35984421 27.43777657 27.35026 27.25332 27.84416 27.78126 27.42101 27.56841 amine meta cell part;en Arginine and proline m 13 13 13 38.4 38.4 38.4 60.909 0 70.617 30.87247 72 10 11 10 5 9 12 11 10 1.564815582 0.451118012 0.303409576 2.902694652 Q60715;E9 Q60715;E9 Prolyl 4-hydroxylase subunit alpha-1 **P4ha1** sp|Q60715|P4HA1_MOUSE Prolyl 4-hydroxylase subunit alpha-1 OS=Mus musculus OX=10090 GN=P4ha1 PE=1 SV=2;tr|E9Q7B0|E9Q7B0_MOUSE Procollagen-proline 4-dioxygenase OS=Mus musculus OX=10090 GN=P4ha1 PE=1 SV=1

25.37970924 25.7993412 26.21764 25.7233 25.40882 25.21638 25.26721 25.10009 cell part;int Spliceosome 6 6 6 30.2 30.2 30.2 23.612 0 7.3073 28.83747 25 4 4 5 3 3 3 3 4 1.56163921 0.452668731 -0.531872272 -2.897087764 Q9CPW7 Q9CPW7 Zinc finger matrin-type protein 2 **Zmat2** sp|Q9CPW7|ZMAT2_MOUSE Zinc finger matrin-type protein 2 OS=Mus musculus OX=10090 GN=Zmat2 PE=2 SV=1

23.03751564 23.27113342 26.02789 23.16886 20.1572 23.04298 20.42153 20.42072 cell part;membrane;plasma membra 3 3 3 14.3 14.3 14.3 32.506 0 9.4661 26.94682 4 1 1 3 1 0 1 0 0 1.560820438 0.452037037 -2.865743637 -2.895643011 B1AXV0;F6 B1AXV0 DOMON domain-containing protein FRRS1L **Frrs1l** sp|B1AXV0|FRS1L_MOUSE DOMON domain-containing protein FRRS1L OS=Mus musculus OX=10090 GN=Frrs1l PE=1 SV=1

25.86398506 25.35924149 25.09023 25.06669 24.57346 24.6381 24.98694 24.78835 cellular comcell part;cytoplasm;cytoplasmic me 9 9 9 20.3 20.3 20.3 70.525 0 11.39 28.31203 18 4 4 4 4 4 4 4 6 1.55950656 0.451852308 -0.598323345 -2.893325079 P59016;S4 P59016 Vacuolar protein sorting-associated protein 33B **Vps33b** sp|P59016|VP33B_MOUSE Vacuolar protein sorting-associated protein 33B OS=Mus musculus OX=10090 GN=Vps33b PE=1 SV=1

20.65860176 22.06485367 21.75193 20.05681 22.87527 22.62299 22.12417 22.60806 cell part;membrane 3 3 3 51.2 51.2 51.2 9.2858 0.000164 3.0064 25.08205 6 1 0 1 0 1 1 1 3 1.553567158 0.455730061 1.424571991 2.882853786 Q8BTE5 Q8BTE5 Protein CEBPZOS **Cebpzos** sp|Q8BTE5|CEBOS_MOUSE Protein CEBPZOS OS=Mus musculus OX=10090 GN=Cebpzos PE=3 SV=2

25.06282806 25.05262566 24.6392 25.26112 24.72562 24.15792 24.10773 24.6418 biological r cell part;intracellular membrane-bo 2 2 2 19.2 19.2 19.2 17.307 0 4.8264 28.04046 9 1 1 1 1 2 2 2 2 1.552105992 0.455437309 -0.595677853 -2.880279459 Q923X4;B7 Q923X4;B7 Glutaredoxin-2, mitochondrial **Glrx2** sp|Q923X4|GLRX2_MOUSE Glutaredoxin-2, mitochondrial OS=Mus musculus OX=10090 GN=Glrx2 PE=1 SV=1;tr|B7ZC40|B7ZC40_MOUSE Glutaredoxin-2, mitochondrial OS=Mus musculus OX=10090 GN=Glrx2 PE=1 SV=1

32.00948715 31.90687943 32.01152 31.86515 31.71955 31.87665 31.84206 31.71387 anatomical cell part;cel Endocytosis;Vasopress 15 15 4 62.8 62.8 15.6 24.489 0 165.44 35.09805 124 14 14 12 15 13 12 12 11 1.547785574 0.45952439 -0.160225868 -2.872671606 P46638;G3 P46638;G3 Ras-related protein Rab-11B **Rab11b;Rab11a** sp|P46638|RB11B_MOUSE Ras-related protein Rab-11B OS=Mus musculus OX=10090 GN=Rab11b PE=1 SV=3;tr|G3UZD3|G3UZD3_MOUSE Ras-related protein Rab-11B OS=Mus musculus OX=10090 GN=Rab11b PE=1 SV=1;tr|G3UY29|G3UY29_MOUSE Ras-related protein Rab-11B OS=Mus musculu

21.92368507 20.55194473 22.56132 21.73331 22.90327 22.94867 22.85516 22.86797 biological r cell part;cytoplasm;intracellular me 2 2 2 9.1 9.1 9.1 31.139 0 4.4418 25.87692 9 1 1 1 1 1 2 1 2 1.540858376 0.467890578 1.201202393 2.860485851 Q9DCX1 Q9DCX1 MAD2L1-binding protein **Mad2l1bp** sp|Q9DCX1|MD2BP_MOUSE MAD2L1-binding protein OS=Mus musculus OX=10090 GN=Mad2l1bp PE=1 SV=2

19.88583374 20.68232155 21.60699 21.85434 23.18456 22.15168 22.16435 22.38572 anatomical cell part;cytoplasm;intracellular me 6 3 3 8.9 5.1 5.1 122.46 0 13.352 26.52973 10 1 1 1 1 1 2 2 1 1.538618437 0.468751515 1.464205742 2.856548781 Q64318;E9 Q64318;E9 Zinc finger E-box-binding homeobox 1 **Zeb1** sp|Q64318|ZEB1_MOUSE Zinc finger E-box-binding homeobox 1 OS=Mus musculus OX=10090 GN=Zeb1 PE=1 SV=1;tr|E9PXY5|E9PXY5_MOUSE Zinc finger E-box-binding homeobox 1 (Fragment) OS=Mus musculus OX=10090 GN=Zeb1 PE=1 SV=8

22.91307449 21.00749016 17.3131 19.8201 23.51689 25.94313 23.40626 23.37425 catabolic p cell part;cyt MetabolismREACT_274 5 5 5 13.8 13.8 13.8 55.948 0 10.705 27.42021 13 1 0 0 0 2 4 1 3 1.538030961 0.468507553 3.796691895 2.855516454 P33267 P33267 Cytochrome P450 2F2 **Cyp2f2** sp|P33267|CP2F2_MOUSE Cytochrome P450 2F2 OS=Mus musculus OX=10090 GN=Cyp2f2 PE=1 SV=1

23.36879539 22.3794384 22.73018 23.17335 19.72678 21.81511 22.32376 20.76027 biological r cell part;cytoplasm;intracellular me 3 3 3 21 21 21 17.521 0 6.123 25.76812 3 1 1 1 1 2 2 1 0 1.529029573 0.475698795 -1.756458759 -2.839712519 Q5EBG6 Q5EBG6 Heat shock protein beta-6 **Hspb6** sp|Q5EBG6|HSPB6_MOUSE Heat shock protein beta-6 OS=Mus musculus OX=10090 GN=Hspb6 PE=1 SV=1

24.88725281 24.65031433 24.19873 24.86292 24.18722 23.87549 24.36165 24.02597 aging;anato cell part;cyt Adherens j REACT_294 6 6 6 12 12 12 67.121 0 10.69 27.61288 15 4 3 4 4 3 3 4 1 1.515772363 0.487435435 -0.537220478 -2.81648226 Q62312 Q62312 TGF-beta receptor type-2 **Tgfbr2** sp|Q62312|TGFR2_MOUSE TGF-beta receptor type-2 OS=Mus musculus OX=10090 GN=Tgfbr2 PE=1 SV=1

22.47258759 23.56022644 23.48591 21.35138 20.99569 18.72721 20.69472 21.48161 biological r cell part;cytoplasm;endoplasmic ret 3 3 3 35.1 35.1 35.1 15.278 0.007878 1.4816 26.00434 3 2 1 2 1 1 1 1 1 1.515186874 0.486814371 -2.242717743 -2.815457564 Q9ER81 Q9ER81 Torsin-1A-interacting protein 2, isoform IFRG15 **Tor1aip2** sp|Q9ER81|IFG15_MOUSE Torsin-1A-interacting protein 2, isoform IFRG15 OS=Mus musculus OX=10090 GN=Tor1aip2 PE=1 SV=1

28.60378265 28.66197014 29.04035 28.53438 28.8909 29.17453 29.13521 29.09713 establishm cell part;ext Calcium sig REACT_327 37 24 20 41.1 31.5 28 134.75 0 101.07 32.21749 141 17 15 19 16 19 18 20 18 1.513016303 0.487797015 0.364321232 2.811659646 G5E829;A0 G5E829 Plasma membrane calcium-transporting ATPase 1 **Atp2b1** sp|G5E829|AT2B1_MOUSE Plasma membrane calcium-transporting ATPase 1 OS=Mus musculus OX=10090 GN=Atp2b1 PE=1 SV=1

25.64880562 25.67470741 24.8768 25.68223 26.82372 26.28884 26.62296 25.69458 biological r cell part;cytoplasm;intracellular me 10 10 10 24 24 24 60.785 0 27.867 29.37084 38 5 4 3 3 7 8 7 8 1.507103674 0.494166667 0.886889458 2.801321367 A0A2R8VH A0A2R8VH Calcium-binding and coiled-coil domain-containin **Calcoco1** tr|A0A2R8VHU8|A0A2R8VHU8_MOUSE Calcium-binding and coiled-coil domain-containing protein 1 (Fragment) OS=Mus musculus OX=10090 GN=Calcoco1 PE=1 SV=1;tr|E9Q7U2|E9Q7U2_MOUSE Calcium-binding and coiled-coil domain-containing protein 1 OS=Mus musculus OX=10090

26.75773621 26.42075729 26.46456 26.85555 26.31869 25.94085 26.30661 26.33528 anatomical cell part;centrosome;cy REACT_272 10 10 7 40.4 40.4 33.4 38.522 0 34.976 29.66756 39 6 6 6 6 7 5 6 5 1.498080125 0.504047478 -0.399292946 -2.785563867 Q9CZA6;F6 Q9CZA6 Nuclear distribution protein nudE homolog 1 **Nde1** sp|Q9CZA6|NDE1_MOUSE Nuclear distribution protein nudE homolog 1 OS=Mus musculus OX=10090 GN=Nde1 PE=1 SV=1

29.15904045 29.10671806 28.70945 29.06381 29.30367 29.46428 29.2135 29.32075 cellular comcell part;macromolecul REACT_274 21 21 21 69.4 69.4 69.4 55.971 0 210.34 32.51388 120 19 14 18 14 13 16 17 16 1.483420644 0.522508876 0.315793991 2.760015957 Q8BJY1;F7 Q8BJY1 26S proteasome non-ATPase regulatory subunit 5 **Psmd5** sp|Q8BJY1|PSMD5_MOUSE 26S proteasome non-ATPase regulatory subunit 5 OS=Mus musculus OX=10090 GN=Psmd5 PE=1 SV=4

24.92025375 25.0832653 25.03687 24.64927 24.22435 24.51045 24.15399 24.81719 anatomical cell part;extracellular or REACT_320 1 1 1 3.4 3.4 3.4 101.16 0 9.4515 27.9678 13 1 1 1 1 1 1 1 1 1.481423103 0.523575221 -0.495921612 -2.756539592 Q03146 Q03146 Epithelial discoidin domain-containing receptor 1 **Ddr1** sp|Q03146|DDR1_MOUSE Epithelial discoidin domain-containing receptor 1 OS=Mus musculus OX=10090 GN=Ddr1 PE=2 SV=2

25.3761692 25.3239212 25.69368 25.63948 25.82663 25.86483 25.99393 25.6437 biological r cell part;cel SNARE interactions in v 8 7 7 46.2 43.8 43.8 33.203 0 40.983 29.03981 26 4 3 4 4 5 5 5 4 1.481087005 0.522376471 0.323959351 2.755954786 Q3TJ55;Q8 Q3TJ55;Q8 Syntaxin-2 **Stx2** tr|Q3TJ55|Q3TJ55_MOUSE Syntaxin-2 OS=Mus musculus OX=10090 GN=Stx2 PE=1 SV=1;tr|Q80W45|Q80W45_MOUSE Syntaxin-2 OS=Mus musculus OX=10090 GN=Stx2 PE=1 SV=1;sp|Q00262|STX2_MOUSE Syntaxin-2 OS=Mus musculus OX=10090 GN=Stx2 PE=1 SV=1

20.35557938 20.46127701 23.18797 18.84089 22.74974 23.19711 23.60399 23.4665 biological r cell part;en Protein processing in e 2 2 2 4.7 4.7 4.7 63.869 0 4.5084 26.21191 5 0 0 1 0 1 2 1 1 1.478753615 0.523002933 2.542908192 2.751895628 Q61712;F6 Q61712;F6 DnaJ homolog subfamily C member 1 **Dnajc1** sp|Q61712|DNJC1_MOUSE DnaJ homolog subfamily C member 1 OS=Mus musculus OX=10090 GN=Dnajc1 PE=1 SV=1;tr|F6ZL86|F6ZL86_MOUSE DnaJ homolog subfamily C member 1 (Fragment) OS=Mus musculus OX=10090 GN=Dnajc1 PE=1 SV=1;tr|F6WEH1|F6WEH1_MOUSE DnaJ homolog subfam

25.95282555 26.18057823 26.54097 26.18956 26.17392 25.23542 25.25061 25.41983 biological r cell part;cytoplasm;extracellular org 20 10 10 30.6 20.1 20.1 111.84 0 31.219 29.25462 45 5 7 5 4 5 5 5 5 1.47840217 0.521473684 -0.696038246 -2.751284392 Q80YR5;D6 Q80YR5 Scaffold attachment factor B2 **Safb2** sp|Q80YR5|SAFB2_MOUSE Scaffold attachment factor B2 OS=Mus musculus OX=10090 GN=Safb2 PE=1 SV=2

21.20372963 20.69266319 20.2365 21.06271 31.00658 32.39433 24.08318 22.65452 anatomical cell part;cytoplasmic membrane-bo 37 37 37 74.6 74.6 74.6 70.643 0 251.26 33.63526 95 0 0 0 0 29 37 4 2 1.478020549 0.519953353 6.735751629 2.750620713 Q03517;Q Q03517;Q Secretogranin-2;Secretoneurin;Manserin **Scg2** sp|Q03517|SCG2_MOUSE Secretogranin-2 OS=Mus musculus OX=10090 GN=Scg2 PE=1 SV=1;tr|Q4W8U9|Q4W8U9_MOUSE Manserin OS=Mus musculus OX=10090 GN=Scg2 PE=1 SV=1

24.75353432 24.18563271 24.68442 24.62817 25.24978 25.01937 24.83586 24.88674 amine metabolic proces Pyruvate metabolism 5 5 5 26.9 26.9 26.9 31.49 0 9.5044 28.10154 11 3 3 4 3 3 4 4 3 1.477757175 0.51905814 0.434996128 2.750162702 Q9DB32;D Q9DB32;D Hydroxyacylglutathione hydrolase-like protein **Haghl** sp|Q9DB32|HAGHL_MOUSE Hydroxyacylglutathione hydrolase-like protein OS=Mus musculus OX=10090 GN=Haghl PE=1 SV=1;tr|D3YZU6|D3YZU6_MOUSE Hydroxyacylglutathione hydrolase-like protein OS=Mus musculus OX=10090 GN=Haghl PE=1 SV=1

22.41573334 22.59133339 24.4883 22.54439 19.90209 21.47896 19.26742 22.18769 biological r cell part 2 2 2 3.1 3.1 3.1 89.135 0 5.2754 25.46345 2 2 2 2 2 0 0 0 0 1.474042116 0.521101449 -2.300901413 -2.743704298 Q80TN4 Q80TN4 DnaJ homolog subfamily C member 16 **Dnajc16** sp|Q80TN4|DJC16_MOUSE DnaJ homolog subfamily C member 16 OS=Mus musculus OX=10090 GN=Dnajc16 PE=1 SV=2

28.65686607 27.84122086 28.24291 28.53092 28.95155 28.74224 28.82745 28.7943 cellular ket cell part;cyt Pentose and glucurona 15 15 15 66.8 66.8 66.8 35.208 0 105.06 31.91267 101 13 12 11 12 12 14 15 14 1.472543979 0.521676301 0.510902405 2.741100996 Q99KP3 Q99KP3 Lambda-crystallin homolog **Cryl1** sp|Q99KP3|CRYL1_MOUSE Lambda-crystallin homolog OS=Mus musculus OX=10090 GN=Cryl1 PE=1 SV=3

23.5525074 24.88585854 24.70625 24.38034 25.05875 25.83028 25.91668 24.96243 biological r cell part;cyt RNA degrad REACT_297 9 9 9 15.4 15.4 15.4 113.88 0 28.816 28.51326 28 3 6 4 3 4 7 5 4 1.471307811 0.522270893 1.060798645 2.7389534 Q8VHK9 Q8VHK9 ATP-dependent RNA helicase DHX36 **Dhx36** sp|Q8VHK9|DHX36_MOUSE ATP-dependent DNA/RNA helicase DHX36 OS=Mus musculus OX=10090 GN=Dhx36 PE=1 SV=2

27.64450836 27.53274536 27.76945 27.67013 28.02818 27.97564 27.85365 27.70668 catabolic p cell part;cyt RNA degrad REACT_330 4 4 4 65 65 65 9.1275 0 74.029 31.0717 29 4 4 4 4 4 4 4 4 1.471192887 0.521103448 0.236827374 2.738753764 P62313 P62313 U6 snRNA-associated Sm-like protein LSm6 **Lsm6** sp|P62313|LSM6_MOUSE U6 snRNA-associated Sm-like protein LSm6 OS=Mus musculus OX=10090 GN=Lsm6 PE=1 SV=1

20.62388229 19.62415123 21.09442 21.94903 22.07248 23.96702 21.92528 22.6756 biosynthet cell part;ext One carbon pool by fol 4 4 4 20.9 20.9 20.9 37.863 0 5.386 26.24102 4 1 1 2 0 1 2 1 2 1.469450738 0.522578797 1.837224007 2.735727924 P18155 P18155 Bifunctional methylenetetrahydrofolate dehydrog **Mthfd2** sp|P18155|MTDC_MOUSE Bifunctional methylenetetrahydrofolate dehydrogenase/cyclohydrolase, mitochondrial OS=Mus musculus OX=10090 GN=Mthfd2 PE=1 SV=1

20.46172333 23.08833694 20.67713 19.6939 23.09288 22.70246 23.25523 22.99056 biological r cell part;centrosome;cytoplasm;intr 2 2 2 4.4 4.4 4.4 60.642 0 4.4342 25.75791 5 0 1 0 0 1 1 2 2 1.467780562 0.522777143 2.030008316 2.732827897 Q3TVW5;F Q3TVW5;F Trichoplein keratin filament-binding protein **Tchp** sp|Q3TVW5|TCHP_MOUSE Trichoplein keratin filament-binding protein OS=Mus musculus OX=10090 GN=Tchp PE=1 SV=2;tr|F6Q720|F6Q720_MOUSE Trichoplein keratin filament-binding protein (Fragment) OS=Mus musculus OX=10090 GN=Tchp PE=1 SV=1

23.90416908 24.47654915 24.40594 24.45444 24.54887 24.8275 24.68121 24.88171 carbohydra cell part;en N-Glycan b REACT_320 2 2 2 7.1 7.1 7.1 54.417 0 13.828 27.9219 11 2 2 1 2 2 2 2 2 1.466376594 0.522564103 0.424547195 2.730390717 Q921Q3 Q921Q3 Chitobiosyldiphosphodolichol beta-mannosyltran **Alg1** sp|Q921Q3|ALG1_MOUSE Chitobiosyldiphosphodolichol beta-mannosyltransferase OS=Mus musculus OX=10090 GN=Alg1 PE=1 SV=3

23.83277321 23.64636421 23.79956 22.99595 20.91756 20.91872 21.18145 23.66622 2 2 2 19.8 19.8 19.8 32.191 0 3.5283 26.00103 2 2 2 1 1 0 0 0 1 1.464391721 0.523909091 -1.897674561 -2.726946081 B1APT1;Q9 B1APT1;Q9 Metallophosphoesterase MPPED2 **Mpped2** tr|B1APT1|B1APT1_MOUSE Metallophosphoesterase MPPED2 (Fragment) OS=Mus musculus OX=10090 GN=Mpped2 PE=1 SV=1;sp|Q9CZJ0|MPPD2_MOUSE Metallophosphoesterase MPPED2 OS=Mus musculus OX=10090 GN=Mpped2 PE=2 SV=1

34.53826141 34.54737091 34.3442 34.17199 34.63855 34.54927 34.80188 34.83879 behavior;bi cell part;cel Aldosteron REACT_338 65 65 43 64.6 64.6 42.6 103.58 0 323.31 37.89301 985 59 61 59 60 64 61 61 62 1.463811546 0.522878187 0.306666374 2.72593943 D3YYN7;Q6 D3YYN7;Q6 Sodium/potassium-transporting ATPase subunit a **Atp1a2** tr|D3YYN7|D3YYN7_MOUSE Sodium/potassium-transporting ATPase subunit alpha OS=Mus musculus OX=10090 GN=Atp1a2 PE=1 SV=1;sp|Q6PIE5|AT1A2_MOUSE Sodium/potassium-transporting ATPase subunit alpha-2 OS=Mus musculus OX=10090 GN=Atp1a2 PE=1 SV=1

24.00752831 23.71732903 23.46438 23.70049 23.58932 23.32553 23.16037 23.02739 2 2 2 8.7 8.7 8.7 31.593 0.000485 2.771 26.84518 7 1 1 1 1 1 2 1 1 1.459390774 0.527231638 -0.446779251 -2.718272135 Q9CZT6 Q9CZT6 Protein CMSS1 **Cmss1** sp|Q9CZT6|CMS1_MOUSE Protein CMSS1 OS=Mus musculus OX=10090 GN=Cmss1 PE=2 SV=1

25.68605614 26.08342743 26.04156 26.03465 26.18745 26.37731 26.20245 26.19661 8 2 0 50.6 12.9 0 28.687 0 11.165 29.39842 23 2 2 2 2 2 2 2 2 1.456314427 0.53063662 0.279533863 2.712939812 A0A1B0GS A0A1B0GSH8 tr|A0A1B0GSH8|A0A1B0GSH8_MOUSE Predicted gene 45808 (Fragment) OS=Mus musculus OX=10090 GN=Gm45808 PE=4 SV=1

27.38541603 27.50876999 27.57087 27.44947 27.53668 28.02923 27.8242 27.71967 biological r extracellula Protein processing in e 18 18 18 34.1 34.1 34.1 87.22 0 67.565 30.95303 96 15 15 12 11 13 17 16 13 1.454875238 0.531202247 0.298811436 2.710446128 P27612;F7 P27612 Phospholipase A-2-activating protein **Plaa** sp|P27612|PLAP_MOUSE Phospholipase A-2-activating protein OS=Mus musculus OX=10090 GN=Plaa PE=1 SV=4

29.79326057 30.05827332 29.98482 29.65222 29.95712 30.4175 30.23797 30.39734 biological r cell part;cyt Neuroactiv REACT_284 40 40 40 46.2 46.2 46.2 138.43 0 179.33 33.44369 220 29 26 23 19 23 34 32 35 1.450590344 0.537221289 0.380339622 2.703025091 Q8CIE6;F8 Q8CIE6;F8 Coatomer subunit alpha;Xenin;Proxenin;Coatomer **Copa** sp|Q8CIE6|COPA_MOUSE Coatomer subunit alpha OS=Mus musculus OX=10090 GN=Copa PE=1 SV=2;tr|F8WHL2|F8WHL2_MOUSE Coatomer subunit alpha OS=Mus musculus OX=10090 GN=Copa PE=1 SV=1

28.77812386 29.11577415 28.93013 28.65075 29.02292 29.32571 29.15617 29.44107 amine meta cell part;int Valine, leuc REACT_305 22 22 22 64.1 64.1 64.1 61.378 0 174.29 32.34369 165 18 18 19 15 16 17 16 19 1.449714216 0.536346369 0.36777401 2.701508343 Q3ULD5;Q Q3ULD5;Q Methylcrotonoyl-CoA carboxylase beta chain, mito **Mccc2** sp|Q3ULD5|MCCB_MOUSE Methylcrotonoyl-CoA carboxylase beta chain, mitochondrial OS=Mus musculus OX=10090 GN=Mccc2 PE=1 SV=1;tr|Q6PD20|Q6PD20_MOUSE Mccc2 protein (Fragment) OS=Mus musculus OX=10090 GN=Mccc2 PE=1 SV=1

28.30691147 28.7143383 28.37997 28.29728 28.73331 28.71803 28.90182 28.60038 biological r cell body;cell part;cytoplasmic mem 17 17 17 44.2 44.2 44.2 67.789 0 87.299 31.87635 100 14 14 12 12 14 14 15 14 1.448139798 0.538038997 0.313760281 2.698783252 Q8C788;Q Q8C788;Q Sorting nexin;Sorting nexin-18 **Snx18** tr|Q8C788|Q8C788_MOUSE Sorting nexin OS=Mus musculus OX=10090 GN=Snx18 PE=1 SV=1;sp|Q91ZR2|SNX18_MOUSE Sorting nexin-18 OS=Mus musculus OX=10090 GN=Snx18 PE=1 SV=1

26.99183273 26.90403175 26.93236 27.52021 27.84637 27.51382 27.44632 27.43102 biological r cell body;c Insulin sign REACT_288 5 5 5 23.4 23.4 23.4 20.451 0 20.686 30.61737 33 3 4 3 3 3 4 4 4 1.447426802 0.537344444 0.472275257 2.697549382 Q921J2 Q921J2 GTP-binding protein Rheb **Rheb** sp|Q921J2|RHEB_MOUSE GTP-binding protein Rheb OS=Mus musculus OX=10090 GN=Rheb PE=1 SV=1

34.21052551 34.06919861 33.99167 34.34023 34.3901 34.26689 34.50894 34.44289 amine trans cell leading Cell cycle;N REACT_291 28 28 23 83.7 83.7 71.8 27.771 0 323.31 37.55185 466 26 26 24 28 24 25 26 26 1.444182729 0.54033241 0.249298096 2.691937135 P63101;A0 P63101;A0 14-3-3 protein zeta/delta **Ywhaz** sp|P63101|1433Z_MOUSE 14-3-3 protein zeta/delta OS=Mus musculus OX=10090 GN=Ywhaz PE=1 SV=1;tr|A0A2I3BQ03|A0A2I3BQ03_MOUSE 14-3-3 protein zeta/delta (Fragment) OS=Mus musculus OX=10090 GN=Ywhaz PE=1 SV=1

22.28282928 22.31777191 22.86537 22.0001 22.13057 20.2246 20.45363 21.48239 autophagy; cell part;cyt Regulation of autophag 1 1 1 3.7 3.7 3.7 52.055 0.00275 1.9937 24.47599 1 1 1 1 1 0 0 0 0 1.441289169 0.542950276 -1.293721676 -2.686933699 Q811C2 Q811C2 Cysteine protease ATG4C **Atg4c** sp|Q811C2|ATG4C_MOUSE Cysteine protease ATG4C OS=Mus musculus OX=10090 GN=Atg4c PE=1 SV=2

22.36212158 23.01860619 23.21693 22.50074 21.35019 20.14021 22.57881 21.24553 2 2 2 24.1 24.1 24.1 12.835 0.002462 2.0861 25.32876 3 1 2 2 1 0 0 1 0 1.43949761 0.543592287 -1.445913315 -2.683836941 P0DP60 P0DP60 sp|P0DP60|LYNX1_MOUSE Ly-6/neurotoxin-like protein 1 OS=Mus musculus OX=10090 GN=Lynx1 PE=1 SV=1

27.90426064 28.06383705 27.96692 27.92649 28.11391 28.06758 28.18248 28.02322 cellular met cell part;cyt mRNA surv REACT_291 12 12 12 51.7 51.7 51.7 48.381 0 85.861 31.26361 88 9 9 7 8 9 10 10 10 1.437562546 0.544549451 0.13142395 2.680493107 Q99LC2;A2 Q99LC2 Cleavage stimulation factor subunit 1 **Cstf1** sp|Q99LC2|CSTF1_MOUSE Cleavage stimulation factor subunit 1 OS=Mus musculus OX=10090 GN=Cstf1 PE=1 SV=1

28.90020752 28.95651436 29.09494 29.00571 29.20163 29.3869 29.38636 29.02057 cellular lipi cell part;Golgi apparatu REACT_325 25 25 25 49.2 49.2 49.2 66.943 0 157.85 32.45547 119 19 22 19 18 21 20 20 23 1.435737379 0.545764384 0.259520531 2.677340104 Q9EP69;A0 Q9EP69;A0 Phosphatidylinositide phosphatase SAC1 **Sacm1l** sp|Q9EP69|SAC1_MOUSE Phosphatidylinositol-3-phosphatase SAC1 OS=Mus musculus OX=10090 GN=Sacm1l PE=1 SV=1;tr|A0A5F8MPK9|A0A5F8MPK9_MOUSE Phosphatidylinositol-3-phosphatase SAC1 OS=Mus musculus OX=10090 GN=Sacm1l PE=1 SV=1

26.51249886 26.69032478 26.95527 25.98958 26.98479 27.11386 27.03957 27.39803 cell part;cytoplasm;intr REACT_293 7 7 7 17.5 17.5 17.5 67.314 0 34.935 30.17997 42 7 7 7 6 3 5 5 4 1.43175655 0.549245902 0.597145557 2.670466268 P61222 P61222 ATP-binding cassette sub-family E member 1 **Abce1** sp|P61222|ABCE1_MOUSE ATP-binding cassette sub-family E member 1 OS=Mus musculus OX=10090 GN=Abce1 PE=1 SV=1

28.21687889 28.06507111 28.06332 27.87625 27.35733 27.72585 27.76623 27.91182 cell part;endosome;intracellular me 20 20 20 47.6 47.6 47.6 67.177 0 86.621 31.17099 80 13 14 13 13 12 18 17 15 1.428210595 0.553340599 -0.365071297 -2.664346912 Q80UP5;F7 Q80UP5;F7 Ankyrin repeat domain-containing protein 13A **Ankrd13a** sp|Q80UP5|AN13A_MOUSE Ankyrin repeat domain-containing protein 13A OS=Mus musculus OX=10090 GN=Ankrd13a PE=1 SV=2;tr|F7B209|F7B209_MOUSE Ankyrin repeat domain-containing protein 13A (Fragment) OS=Mus musculus OX=10090 GN=Ankrd13a PE=1 SV=1

23.88818359 24.90916824 25.45764 25.50803 26.20927 25.86361 26.16406 25.71984 anatomical cell part;ma African trypanosomiasi 8 1 1 44.9 8.8 8.8 15.878 0 4.7194 29.3258 10 1 1 1 1 1 1 1 1 1.425292809 0.554902174 1.048438549 2.659314107 P02089 P02089 Hemoglobin subunit beta-2 **Hbb-b2** sp|P02089|HBB2_MOUSE Hemoglobin subunit beta-2 OS=Mus musculus OX=10090 GN=Hbb-b2 PE=1 SV=2

23.22933197 23.43096733 23.44291 23.80469 23.72088 23.7967 24.23286 23.90298 anatomical cell part;cyt Axon guida REACT_331 3 3 3 10.8 10.8 10.8 76.691 0 16.347 26.91878 14 2 2 1 2 2 2 2 1 1.421180475 0.558363144 0.436378479 2.652224677 Q9DBQ6;B Q9DBQ6;B Nuclear factor of activated T-cells, cytoplasmic 1 **Nfatc1** tr|Q9DBQ6|Q9DBQ6_MOUSE Nuclear factor of activated T-cells c1 isoform IB-VIII OS=Mus musculus OX=10090 GN=Nfatc1 PE=1 SV=1;tr|B5B2N5|B5B2N5_MOUSE Nuclear factor of activated T-cells c1 isoform IA-VIII OS=Mus musculus OX=10090 GN=Nfatc1 PE=1 SV=1;tr|B5B2N4|

25.97276688 25.88231659 25.95096 26.29854 26.26496 26.41914 26.4803 26.17331 biological r cell part;extracellular or REACT_271 8 8 8 19.3 19.3 19.3 62.643 0 7.6879 29.47018 29 6 6 6 7 3 4 4 4 1.418822604 0.560962162 0.308280945 2.648161845 A0A1L1SVGA0A1L1SVGCholine transporter-like protein 2 **Slc44a2** tr|A0A1L1SVG6|A0A1L1SVG6_MOUSE Choline transporter-like protein 2 OS=Mus musculus OX=10090 GN=Slc44a2 PE=1 SV=1;sp|Q8BY89|CTL2_MOUSE Choline transporter-like protein 2 OS=Mus musculus OX=10090 GN=Slc44a2 PE=1 SV=2

26.40641022 25.77497482 26.2394 25.50791 25.75855 25.04976 25.24949 25.09736 biological r cell part;cel Regulation REACT_358 7 5 5 7.6 6.5 6.5 180.53 0 8.7477 28.93239 15 5 2 4 1 1 3 3 3 1.412044098 0.568808625 -0.693383217 -2.636489909 Q3UQ44 Q3UQ44 Ras GTPase-activating-like protein IQGAP2 **Iqgap2** sp|Q3UQ44|IQGA2_MOUSE Ras GTPase-activating-like protein IQGAP2 OS=Mus musculus OX=10090 GN=Iqgap2 PE=1 SV=2

27.81836319 27.39272118 27.1037 27.59619 27.89643 27.69869 28.10159 28.19282 cellular met cell part;extracellular organelle;mem 13 12 12 30.7 28.6 28.6 62.63 0 47.14 31.08151 64 9 9 7 9 10 11 11 11 1.410650859 0.569139785 0.494639874 2.634092359 Q9JLB0;E9 Q9JLB0 MAGUK p55 subfamily member 6 **Mpp6** sp|Q9JLB0|MPP6_MOUSE MAGUK p55 subfamily member 6 OS=Mus musculus OX=10090 GN=Mpp6 PE=1 SV=1

32.9601326 32.84987259 32.74707 32.70642 33.38934 33.13441 32.92788 33.02062 alcohol me cell part;cyt Glycolysis / REACT_308 17 17 15 69.7 69.7 61.4 28.832 0 323.31 36.21796 295 16 16 15 15 16 16 15 16 1.409367156 0.569297587 0.302189827 2.631883745 Q9DBJ1 Q9DBJ1 Phosphoglycerate mutase 1 **Pgam1** sp|Q9DBJ1|PGAM1_MOUSE Phosphoglycerate mutase 1 OS=Mus musculus OX=10090 GN=Pgam1 PE=1 SV=3

23.73231316 23.97333717 23.58302 23.80577 24.12888 24.14717 23.89883 24.52636 cell part;endoplasmic reticulum;Go 1 1 1 4.5 4.5 4.5 27.283 0.000637 2.583 27.2683 2 1 1 1 1 1 1 1 1 1.403824136 0.575860963 0.401697159 2.622351814 Q8C407 Q8C407 Protein YIPF4 **Yipf4** sp|Q8C407|YIPF4_MOUSE Protein YIPF4 OS=Mus musculus OX=10090 GN=Yipf4 PE=1 SV=1
[truncated: 23,782 more chars]
